# DCAF13 is essential for mouse uterine function and fertility

**DOI:** 10.1038/s41420-025-02583-w

**Published:** 2025-08-01

**Authors:** Qianhui Zhou, Xiaohui Li, Ningjing Wang, Liang Zhang, Enhui Jiang, Kaixuan Wang, Xingyu Yan, Cong Zhang

**Affiliations:** 1 Department of Reproductive Medicine, Ren Ji Hospital, Shanghai Jiao Tong University School of Medicine, Shanghai, China; 2 Shanghai Key Laboratory for Assisted Reproduction and Reproductive Genetics, Shanghai, China; 3 Shandong Provincial Key Laboratory of Animal Resistance Biology, College of Life Sciences, Shandong Normal University, Jinan, Shandong China; 4 Shandong Laboratory of Advanced Materials and Green Manufacturing at Yantai, Yantai, China; 5 Research Center of Translational Medicine, Jinan Central Hospital Affiliated to Shandong First Medical University, Jinan, Shandong China

**Keywords:** Developmental biology, Cell biology

## Abstract

The incidence of female infertility is a growing worldwide concern and a leading cause of population decline. Therefore, understanding the pathogenesis of infertility is of utmost importance. DDB1 and CUL4 Associated Factor 13 (DCAF13) is a significant component of the CRL4 E3 ubiquitin ligase complex responsible for recognizing substrates and degrading them after polyubiquitylation. DCAF13 has been implicated in oocyte and embryo development, but its role in the uterus remains elusive. To investigate its function, we generated *Dcaf13* conditional knockout (cKO) mice and discovered that the uteri of cKO mice became smaller and thinner as they mature, and the embryos were unable to implant, leading to infertility. Mechanistically, we detected aberrant expression of estrogen and progesterone receptors, along with dysregulation of estrogen- and progesterone-responsive genes in the endometrium. This led to insufficient proliferation of endometrial cells in mice. RNAseq analysis revealed an overall increase in transcription of methylation-related genes, including *SUV39H2*, leading to higher H3K9me3 levels and consequently hindered cell proliferation in the uterus. Furthermore, DCAF13 knockdown resulted in elevated intracellular H3K9me3 levels. In conclusion, these findings suggest that DCAF13 is essential for maintaining the structure of the uterus and fertility. This study potentially contributes to the development of new strategies aimed at improving female reproductive health.

## Introduction

The uterus is a vital organ for mammalian reproduction [1]. It is composed of two primary components: the endometrium and the myometrium [2]. The endometrium consists of a single layer of tubular epithelium and an endometrial stroma consisting of fibroblasts and glands coiled within it. The myometrium, which mainly comprises smooth muscle cells, provide the mechanical force required for contraction of the uterus [3–7]. The uterus has unique reproductive functions, including facilitating the rapid transport of sperm to the fallopian tubes where fertilization occurs, guiding the blastocyst to the optimal site for implantation, forming the placenta around implantation, providing nutrients for the developing embryos, assisting in the timely and safe delivery of the baby, and expelling the placenta after childbirth. Additionally, it constricts blood vessels and compresses the placental area to prevent bleeding following pregnancy [8–11]. A healthy uterus is essential for a successful pregnancy [11–13]. Abnormalities in the structure of the uterus can lead to partial or total irregularities, impacting the pregnancy process and leading to infertility [14–16]. Approximately 8–12% of women in their childbearing years suffer from infertility, making it a significant contributing factor to low birth rates in the population [17–21]. Therefore, understanding the regulatory mechanisms within the uterus is crucial for ensuring female fertility.

DDB1 and CUL4 Associated Factor 13 (DCAF13) is a member of the DCAF protein family and comprises 445 amino acids [22]. It serves as a substrate recognition protein of the CRL4 (Cullin 4-RING Ubiquitin Ligase) E3 ubiquitin ligase complex, which identifies and recruits various substrates, and is an essential component in the development of female reproductive system [22–25]. Recent investigations on DCAF13 in reproduction have focused on both early embryonic development and oocyte growth and maturation [26]. DCAF13 functions as a substrate recognition protein for the CRL4 E3 ubiquitin ligase complex, which polyubiquitinates and degrades SUV39H1, thereby keeping H3K9me3 at a low level and thus promoting early embryonic development [22, 27, 28]. In addition, DCAF13 also mediates the polyubiquitination of PTEN followed by its degradation through the CRL4 E3 ubiquitin ligase complex, maintaining low levels of PTEN and thus activating the PI3K signaling pathway, which facilitates oocyte growth and maturation [25, 27, 29]. While these studies indicate the crucial role of DCAF13 in the regulation of oocytes and early embryonic development, its function in the uterus is still unclear.

Upon conducting a thorough search on the Human Protein Atlas website (https://www.proteinatlas.org/), a noteworthy discovery emerged. Specifically, it was observed that the expression of DCAF13 is significantly enriched in the uterus. This observation hints at a potential irreplaceable role of DCAF13 in this organ. Consequently, a novel avenue for research is presented: investigating the functions of DCAF13 in the uterus, with a particular emphasis on the pathogenesis of infertility.

## Results

### cKO female mice are infertile but have normal ovarian function

**Fig. 1:**
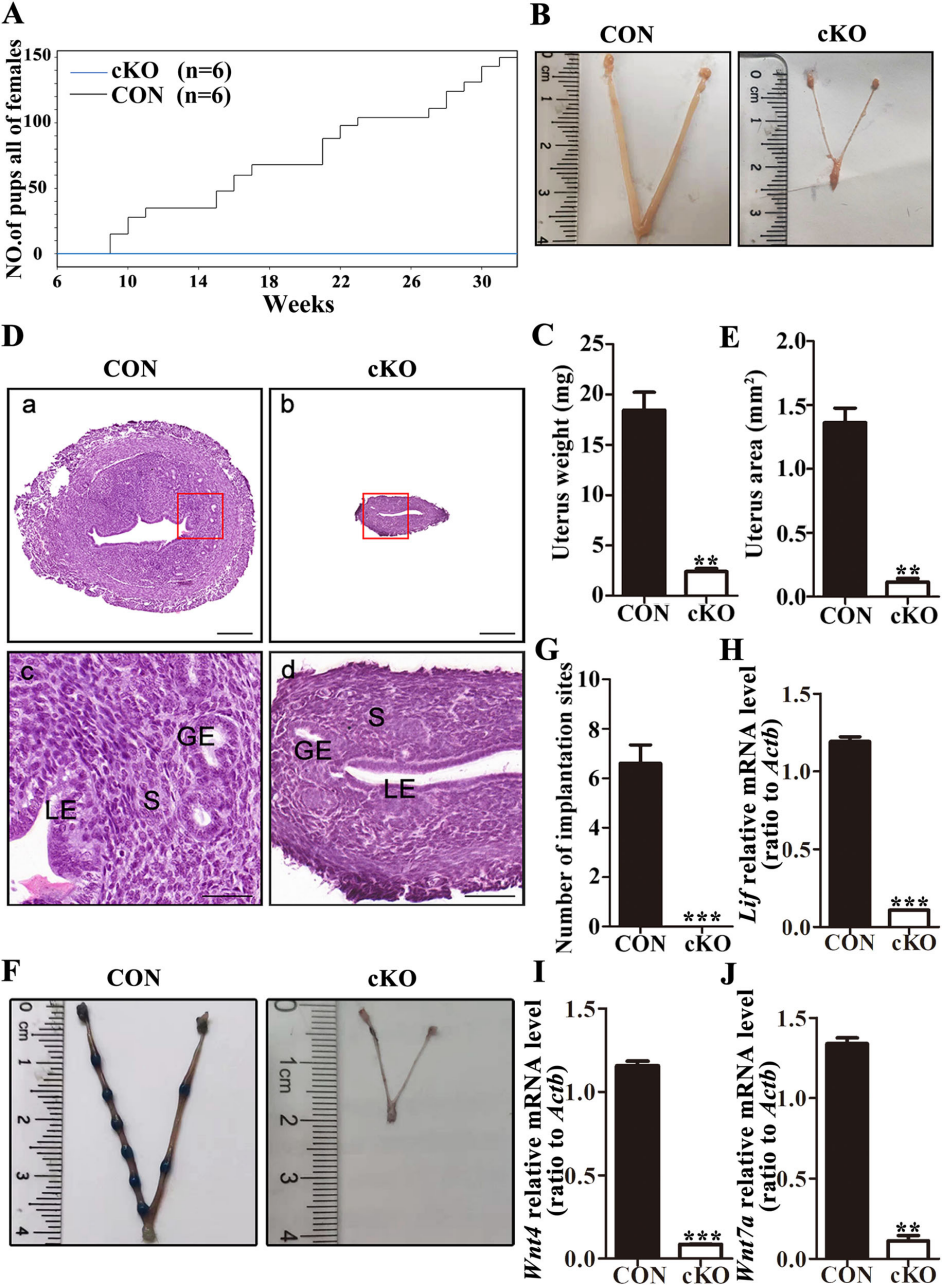
The abnormal morphological structure and function of the uterus in *Dcaf13* cKO mice. **A** The fertility of adult control (*Dcaf13*^*f/f*^) mice and *Dcaf13* cKO mice was detected. **B** Comparison of uterine length between adult non-pregnant control mice and *Dcaf13* cKO mice. **C** The uterine weight of non-pregnant adult *Dcaf13* cKO mice was lower than that of control mice. **D** HE staining reveals the uterine structure in adult non-pregnant control mice and *Dcaf13* cKO mice. highlighting the luminal epithelium (LE), stroma (S), and glandular epithelium (GE). The enlarged images in c and d correspond to the red boxes in images a and b, with scale measurements of 200 μm for a and b, and 50 μm for c and d. **E** The uterine cross-sectional area of adult non-pregnant *Dcaf13* cKO mice was found to be significantly smaller than that of control mice. **F** Comparison of implantation sites between control mice and *Dcaf13* cKO mice. **G** Compared to control mice, the number of implantation sites in *Dcaf13* cKO mice was significantly reduced. **H**–**J** The expression of implantation marker molecules. The mRNA levels were lower in *Dcaf13* cKO mice than those of control mice. *P < 0.05; **P < 0.01; ***P < 0.001.

Previous studies have reported that complete loss of DCAF13 results in early embryo developmental arrest, leading to the inability of morula to develop into blastocysts [22]. To study the role of DCAF13 in the adult uterus while avoiding the harmful effects of its deletion, we generated a *Dcaf13* cKO female mice by crossing *Pgr-Cre*^*+/*−^ male mice with *Dcaf13*^*fl/fl*^ female mice (Fig. S1A). Subsequently, we tested the knockout efficiency of DCAF13 (Fig. S2A, B). We observed the fertility of *Dcaf13*^fl/fl^ (control) and cKO adult females that were housed with fertile WT males at a ratio of 2:1 for six months. Our results revealed that the cKO female mice exhibited normal mating behavior, with vaginal plugs detected, just as the controls did. However, the litter size of cKO female mice was consistently 0, indicating complete infertility (Fig. 1A).

As *Pgr-Cre* is also expressed in the ovary [14], we further investigated whether the structure and function of the ovaries cKO mice were affected. Histological analysis of adult female ovaries showed that the ovaries of cKO mice were similar in size and morphology to those of controls, with normal follicles and corpora lutea (Fig. S1B). Superovulation of cKO females revealed that cKO mice had normal oocyte size and morphology compared to controls, with no significant difference in the number of oocytes (Fig. S1C, D). Additionally, the levels of estrogen and progesterone in cKO females showed no significant differences after superovulation stimulation compared to controls (Fig. S1E, F). In summary, our findings indicate that DCAF13 deletion does not result in any significant abnormalities in the morphological structure and function of the ovaries in cKO mice. The *Dcaf13* cKO mice can be used to study the specific role of DCAF13 in the uterus without affecting ovarian function.

### Abnormal uterine structure and function of cKO female mice

To explore the etiology of infertility in cKO female mice, we conducted an investigation into the expression of DCAF13 in the uterus using the Human Protein Atlas Database (https://www.proteinatlas.org/). Our search results suggest that DCAF13 may play an essential role in uterine function in mice. To further elucidate the impact of DCAF13 deletion on uterine morphology, we conducted a comparative analysis of non-pregnant adult uteri at the same estrous stage. Our analysis revealed that the cKO uteri exhibited significantly reduced length and thickness compared to those of control mice. Specifically, the uterine length of control mice measured approximately 3.5 cm, while that of cKO mice was only 1.5 cm (Fig. 1B). Furthermore, the uterine weight of cKO mice was notably decreased (Fig. 1C). Histological examination of the uteri indicated that the luminal epithelium, stroma, and muscular layer of the uterus in cKO mice were markedly thinner than those in control mice (Fig. 1D). Additionally, the cross-sectional area of the uterus was significantly reduced (Fig. 1E), suggesting a critical role for DCAF13 in uterine architecture. To assess whether the structural abnormalities in the cKO uterus affect its normal function, we examined the implantation sites of female mice cohabiting with fertile males on the fifth day after observing the plugs to evaluate embryo implantation. Our findings revealed a lack of implantation sites in the uterus of cKO mice compared to the control group (Fig. 1F, G). Furthermore, qPCR experiments demonstrated a significant reduction in the transcript levels of implantation marker molecules (*Lif, Wnt4, Wnt7a*) in cKO mice (Fig. 1H–J). Thus, our results indicate the essential role of DCAF13 in maintaining the normal morphological structure and function of the uterus.

### Insufficient endometrium proliferation, increased apoptosis, and DNA damage in cKO female mice

**Fig. 2:**
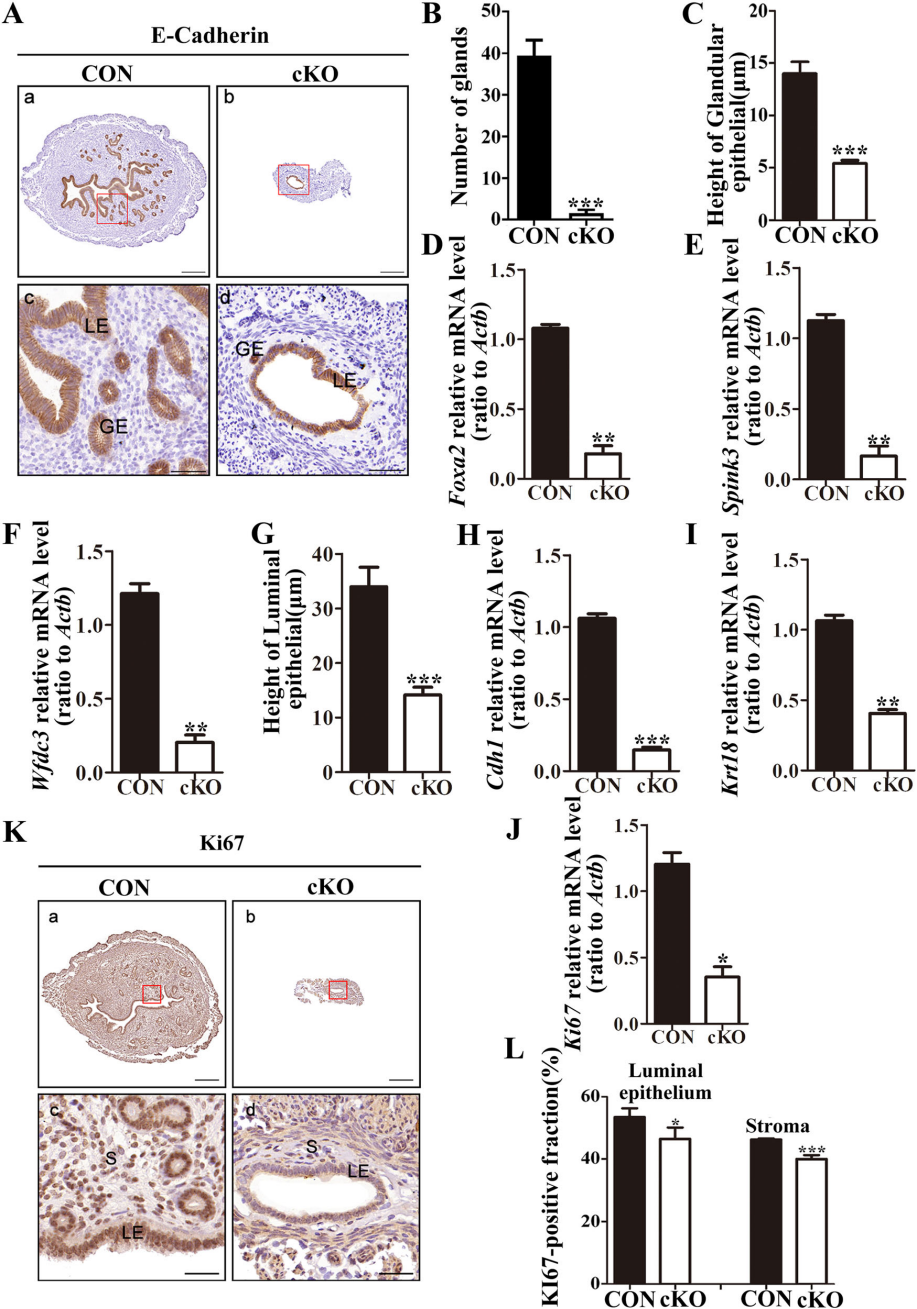
The thin endometrium and insufficient proliferation of endometrial cells in adult *Dcaf13* cKO female mice. **A** E-cadherin staining of uterine cross-sections from adult control mice and *Dcaf13* cKO mice showing the epithelial cell area. **B** Compared with control mice, the number of glands in the uterus of *Dcaf13* cKO mice was significantly reduced. **C** The height of endometrial glandular epithelium in *Dcaf13* cKO mice was significantly lower than that in control mice. **D**–**F**. The mRNA level was lower in *Dcaf13* cKO mice than that of control mice. **G** *Dcaf13* cKO mice exhibited significantly lower endometrial epithelial height than that of control mice. **H**, **I** The mRNA levels of endometrial epithelial marker molecules in *Dcaf13* cKO mice were lower than those in control mice. **J**. qPCR revealed a significantly downregulated *Ki67* mRNA in *Dcaf13* cKO mice, indicating reduced cell proliferation. **K** The expression of KI67 in the uterus of adult control mice and *Dcaf13* cKO mice. **L** Immunohistochemical quantification shows a significant reduction in the expression of KI67 in endometrial luminal epithelial cells and stromal cells of *Dcaf13* cKO mice compared to control mice. LE luminal epithelium, S stroma, GE glandular epithelium. The enlarged images in c and d correspond to the red boxes in images a and b, with scale measurements of 200 μm for a and b, and 50 μm for c and d. *P < 0.05; **P < 0.01; ***P < 0.001.

The endometrium, a crucial component of the uterus, plays a vital role in facilitating embryo implantation [11]. The normalcy of the endometrium directly affects the success of the embryo implantation process [30]. Our immunohistochemical experiments detecting the well-established epithelial cell marker E-cadherin (encoded by gene *Cdh1*) revealed significant abnormalities in the endometrial structure of cKO mice (Fig. 2A). Specifically, cKO mice showed a marked reduction in the number of uterine glands (Fig. 2B). Additionally, there was a decrease in the height of the glandular epithelium (Fig. 2C), and qPCR experiments demonstrated downregulation of endometrial gland marker molecules (*Foxa2, Spink3, Wfdc3*) detected by qPCR experiments (Fig. 2D–F). Similarly, the height of the luminal epithelium in cKO mice also decreased significantly (Fig. 2G). Concurrently, compared with the control group, the cKO uterus exhibited a significant decrease in the expression of epithelial marker molecules (*Cdh1*, *Krt18*) (Fig. 2H, I). These findings suggest that the lack of DCAF13 may induce abnormal proliferation of endometrial cells, ultimately leading to endometrial thinning.

To further explore the etiology of endometrial thinning in cKO mice, we conducted an analysis of the expression of the cell proliferation marker Ki67 in the uterus. Our findings revealed a significant reduction in the transcript level of *Ki67* in cKO mice (Fig. 2J). Additionally, immunohistochemistry results revealed a significantly lower expression of Ki67 in the endometrial epithelial and stromal cells of cKO mice compared to the control group (Fig. 2K, L). This finding strongly suggests that the endometrial cells in cKO mice experience insufficient proliferation.

**Fig. 3:**
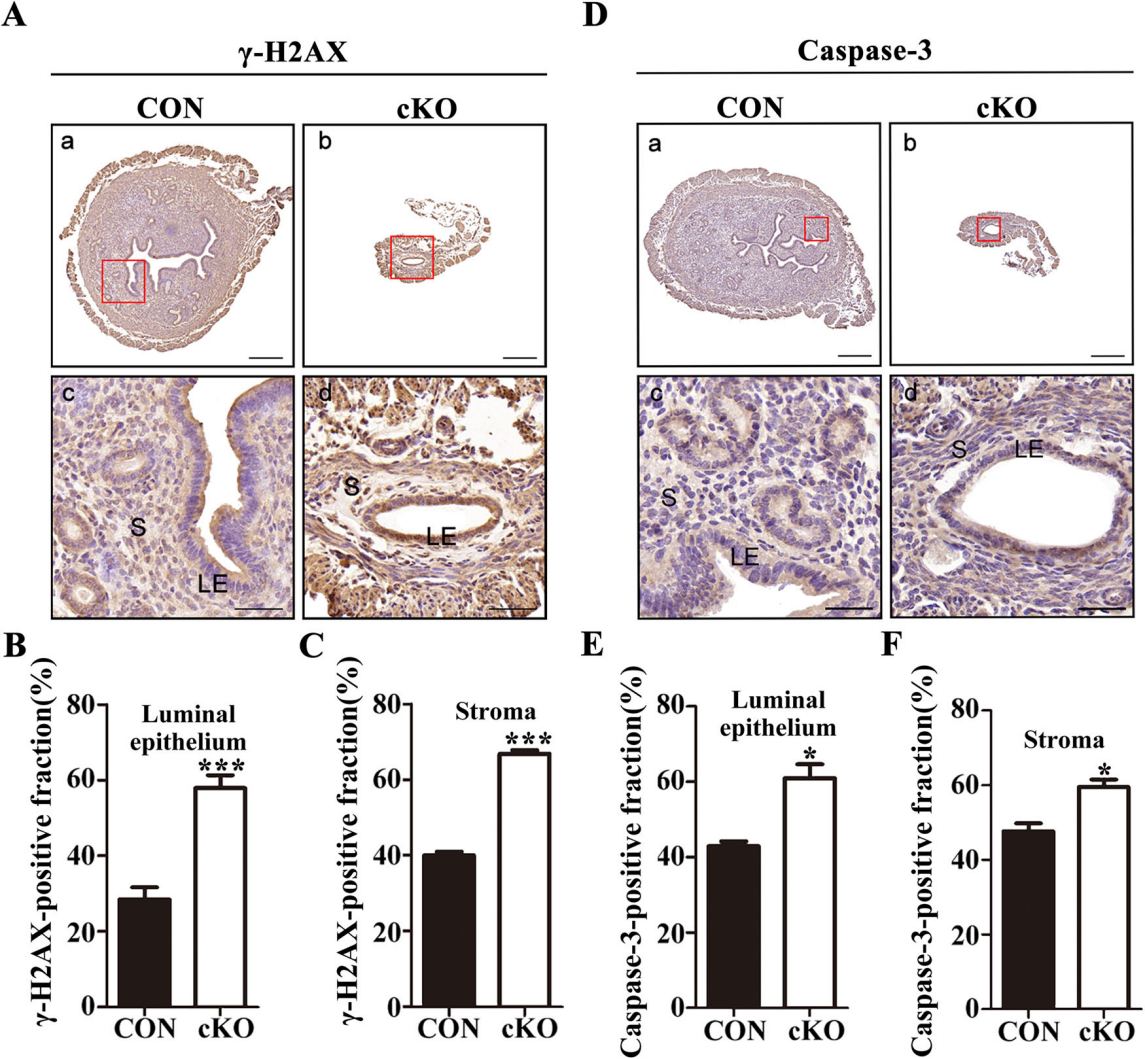
The endometrial cells of *Dcaf13* cKO female mice exhibit increased DNA damage and initiate cell apoptosis. **A** The expression level of γ-H2AX in the uterus of adult control mice and *Dcaf13* cKO mice. **B**, **C** Immunohistochemical quantification shows that the expression of γ-H2AX in the endometrial luminal epithelial cells and stromal cells of *Dcaf1*3 cKO mice was significantly reduced compared to that of control mice. **D** The expression level of Caspase-3 in the uterus. **E**, **F** Immunohistochemical quantification shows that the expression of Caspase-3 in the endometrial luminal epithelial cells and stromal cells of *Dcaf13* cKO mice was significantly decreased compared to control mice. The enlarged images in c and d correspond to the red boxes in images a and b, with scale measurements of 200 μm for a and b, and 50 μm for c and d. LE luminal epithelium, S stroma. *P < 0.05; **P < 0.01; ***P < 0.001.

Subsequently, we utilized immunohistochemistry to examine the expression of the DNA damage marker molecule γ-H2AX. Our results clearly demonstrated a substantially higher expression of γ-H2AX in the endometrial epithelial and stromal cells of cKO mice (Fig. 3A–C). This upregulation indicates increased DNA damage within the endometrial cells of cKO mice. It is well - known that excessive DNA damage can lead to genome instability and has the potential to trigger cell apoptosis, as previously reported [31]. Our subsequent experiments further confirmed an elevated expression of the apoptosis - related proteins Caspase-3 and Cleaved caspase-3 in the epithelial and stromal cells of cKO mice (Figs. 3D–F and S2C, D). These results strongly suggest the initiation of apoptosis in the endometrial cells of cKO mice. Taking together, our findings strongly suggest that the absence of DCAF13 inhibits the proliferation and promotes the apoptosis of endometrial cells in mice. These combined effects ultimately lead to endometrial thinning.

### Abnormal response of the endometrium to hormones in cKO female mice

**Fig. 4:**
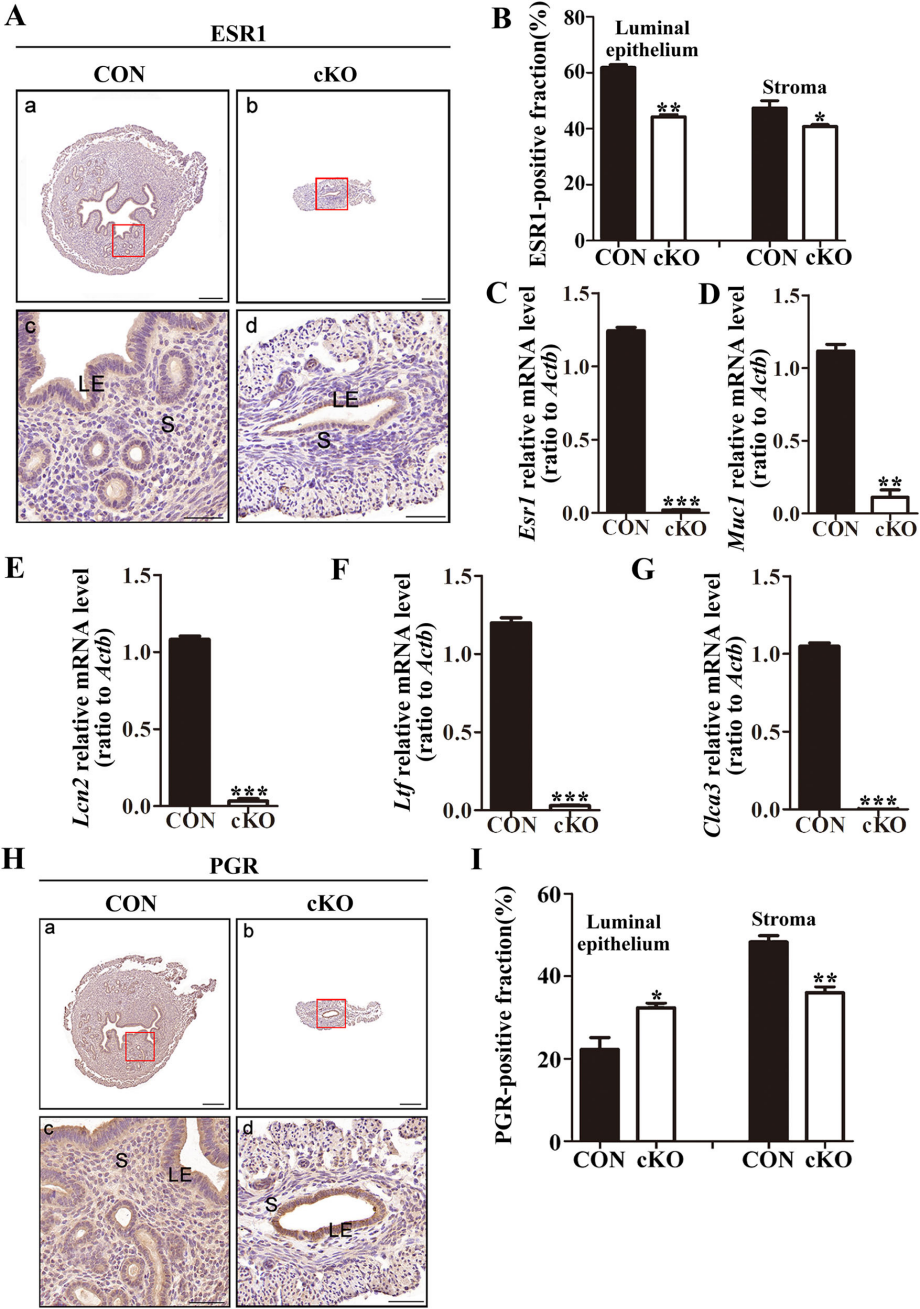
The endometrium of *Dcaf13* cKO female mice exhibited abnormal responses to estrogen and progesterone. **A** The expression level of estrogen receptor ESR1 in the uterus of control and *Dcaf13* cKO mice. **B** Immunohistochemical quantification shows a significant reduction in the expression of ESR1 in the endometrial luminal epithelial cells and stromal cells of *Dcaf13* cKO mice. **C** qPCR analysis revealed a significant downregulation of *Esr1* in *Dcaf13* cKO mice. **D**–**G** qPCR results show a significant downregulation of mRNA levels of estrogen-responsive genes in the uterus of *Dcaf13* cKO mice. **H** The expression level of PGR in the uterus. **I** Immunohistochemical quantification shows a significant upregulation of PGR expression in endometrial luminal epithelial cells of *Dcaf13* cKO mice, but a significant downregulation in endometrial stromal cells. The enlarged images in c and d correspond to the red boxes in images a and b, with scale measurements of 200 μm for a and b, and 50 μm for c and d. LE luminal epithelium, S stroma. *P < 0.05; **P < 0.01; ***P < 0.001.

Estrogen and progesterone are essential for regulating the proliferation and differentiation of endometrial cells during embryo implantation and early pregnancy [32–35]. Estrogen specifically recognizes and binds to the estrogen receptor (ESR1) in endometrial stromal cells, which in turn controls the production of paracrine factors like epidermal growth factor and insulin-like growth factor, influencing the proliferation of endometrial epithelial cells [36–38]. Estrogen also activates ESR1 in epithelial cells, promoting their differentiation [39]. To study the impact of DCAF13 deletion on these processes, we conducted immunohistochemical experiments to evaluate the expression of ESR1 in the uteri of mice. Our findings revealed that the staining intensity in the uteri of cKO mice was weaker compared to the control group, indicating lower levels of ESR1 expression (Fig. 4A). Quantitative analysis of the immunohistochemical results confirmed significantly reduced expression of ESR1 in both endometrial epithelial and stromal cells of cKO mice (Fig. 4B). The levels of *Esr1* transcript were also significantly lower in cKO mice (Fig. 4C). Furthermore, we observed a significant decrease in the expression of estrogen-responsive genes (*Muc1, Ltf, Lcn2, Clca3*) in the uteri of cKO mice (Fig. 4D–G), indicating an inadequate response of the endometrium to estrogen [40].

Progesterone-mediated regulation of endometrial cell proliferation requires specific binding to the PGR. Therefore, we performed immunohistochemistry to detect PGR expression in the uterus of mice. Our results showed that PGR was expressed in the uterus of both cKO and control mice (Fig. 4H). Interestingly, PGR expression in endometrial luminal epithelial cells was significantly upregulated in cKO mice compared to the control group. However, PGR expression in endometrial stromal cells was significantly down-regulated (Fig. 4I). We also used qPCR to detect mRNA levels of *Pgr* in the uterus of cKO mice, but found no significant difference from the control group (Fig. S3A). Additionally, qPCR results revealed a significant downregulation of progesterone-responsive genes (*Hand2, Nr2f2*) and an upregulation of others (*Lrp2, Areg*) in the uterus of cKO mice, while transcript levels of *Ihh* were not statistically different (Fig. S3B–F). These results suggest that the endometrium of cKO mice exhibits a disordered response to progesterone. In conclusion, our results indicate that DCAF13 deletion disrupts the normal expression of both estrogen and progesterone receptors, as well as hormonal responsive genes regulated by these hormones in the mouse endometrium.

### Increased expression of SUV39H2 leads to elevated H3K9me3 levels

**Fig. 5:**
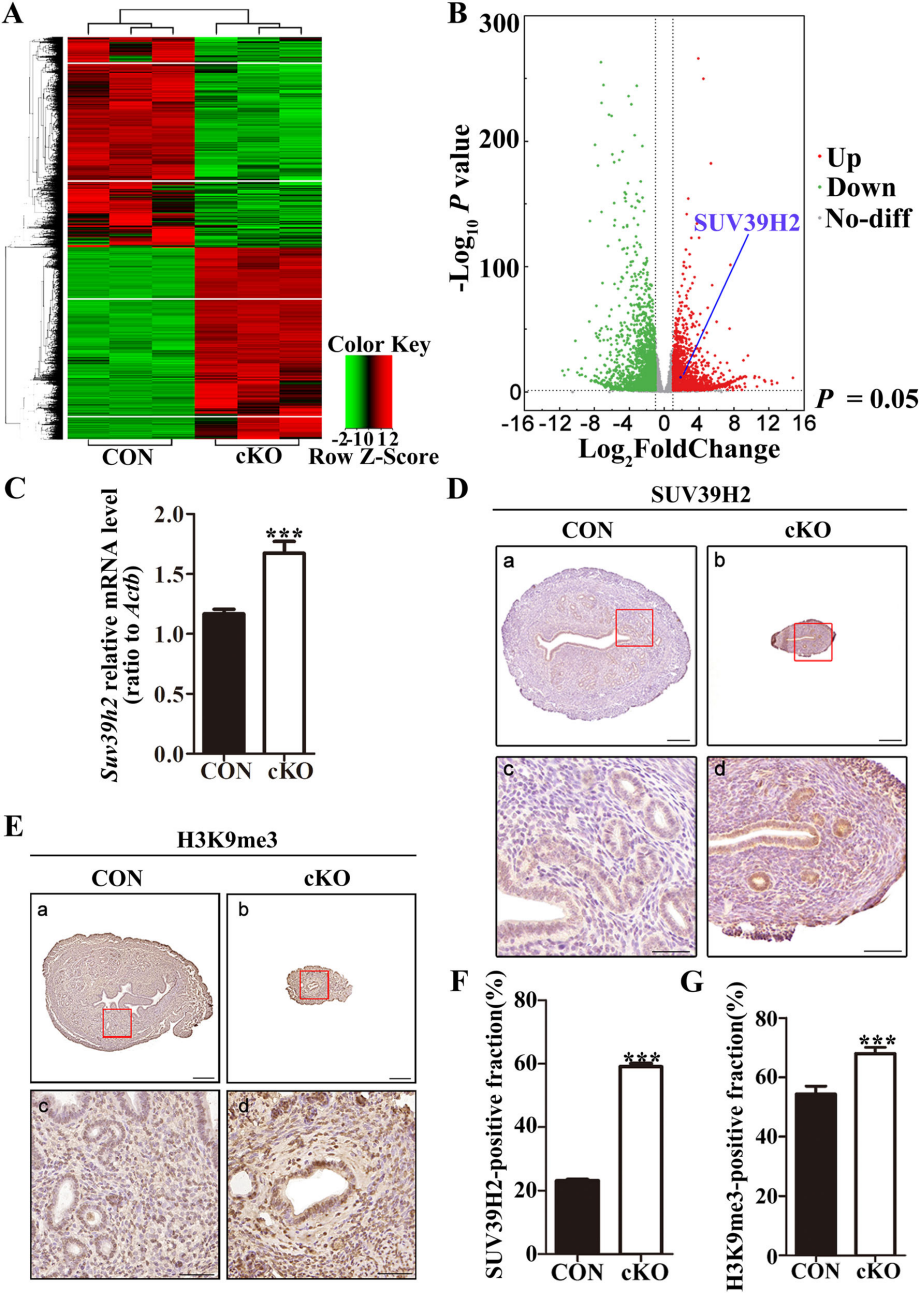
Increased H3K9me3 levels in the uterus of *Dcaf13* cKO female mice. **A** Uterine tissues from adult control mice and *Dcaf13* cKO mice were subjected to RNA-seq analysis, and heat maps were generated to display differentially expressed genes. **B** Volcano map was used to visualize the differentially expressed genes in uterine tissues. **C** qPCR analysis revealed a significant upregulation of *Suv39h2* level in *Dcaf13* cKO mice. **D** Immunohistochemical analysis showed the expression of SUV39H2 in the uterus. **E** The levels of H3K9me3 in the uterus. **F**, **G** Immunohistochemical quantification demonstrated a significant increase in SUV39H2 and H3K9me3 expression in *Dcaf13* cKO mice. The enlarged images in Fig. c and Fig. d correspond to the red boxes in images Fig. a and Fig. b, with scale measurements of 200 μm for a and b, and 50 μm for c and d. *P < 0.05; **P < 0.01; ***P < 0.001.

To investigate the underlying mechanism of infertility in cKO mice, we employed RNA-seq to analyze uterine tissue from adult mice. By integrating the sequencing data, we identified DEGs and selected key genes to generate a heat map (Fig. 5A). Additionally, we created a volcano map based on the expression of all genes, which identified the significantly upregulated *Suv39h2* (Fig. 5B). We then confirmed the transcription of *Suv39h2* in the uterus of cKO mice via qPCR, which increased significantly compared to the control (Fig. 5C). We further examined the expression of SUV39H2 using immunohistochemistry, which demonstrated a notable upregulation in the cKO group (Fig. 5D).

SUV39H2 is a highly conserved methyltransferase that mediates di-methylation and tri-methylation modifications of H3K9 (H3K9me2, H3K9me3), with H3K9me3 being more prevalent [41–43]. Therefore, we examined the levels of H3K9me3 expression and found that the staining was darker (Fig. 5E). Quantitative statistical analysis of the results from the immunohistochemical experiments further confirmed that the levels of SUV39H2 and H3K9me3 in the uterus of cKO mice was significantly increased (Fig. 5F, G). In conclusion, these results demonstrate that the loss of DCAF13 leads to an increase in the expression of SUV39H2 and consequently elevated levels of H3K9me3.

### DCAF13 regulates H3K9me3 level by interacting with SUV39H2A

Due to limitations imposed by the small size of the uterus in cKO mice, obtaining sufficient materials for subsequent experiments was unfeasible. Therefore, we proceeded to study the function of DCAF13 in vitro. We synthesized five siRNAs targeting *Dcaf*13, and verified their knockdown efficiency using Hela cells. We detect the transcript and expression levels of DCAF13 using qPCR and Western Blotting from Hela cells transfected with *Dcaf*13 siRNA for 48 h, respectively (Fig. S4A–C). The *siDcaf*13#1 and *siDcaf*13#4 exhibited higher knockdown efficiency and were utilized in subsequent experiments.

**Fig. 6:**
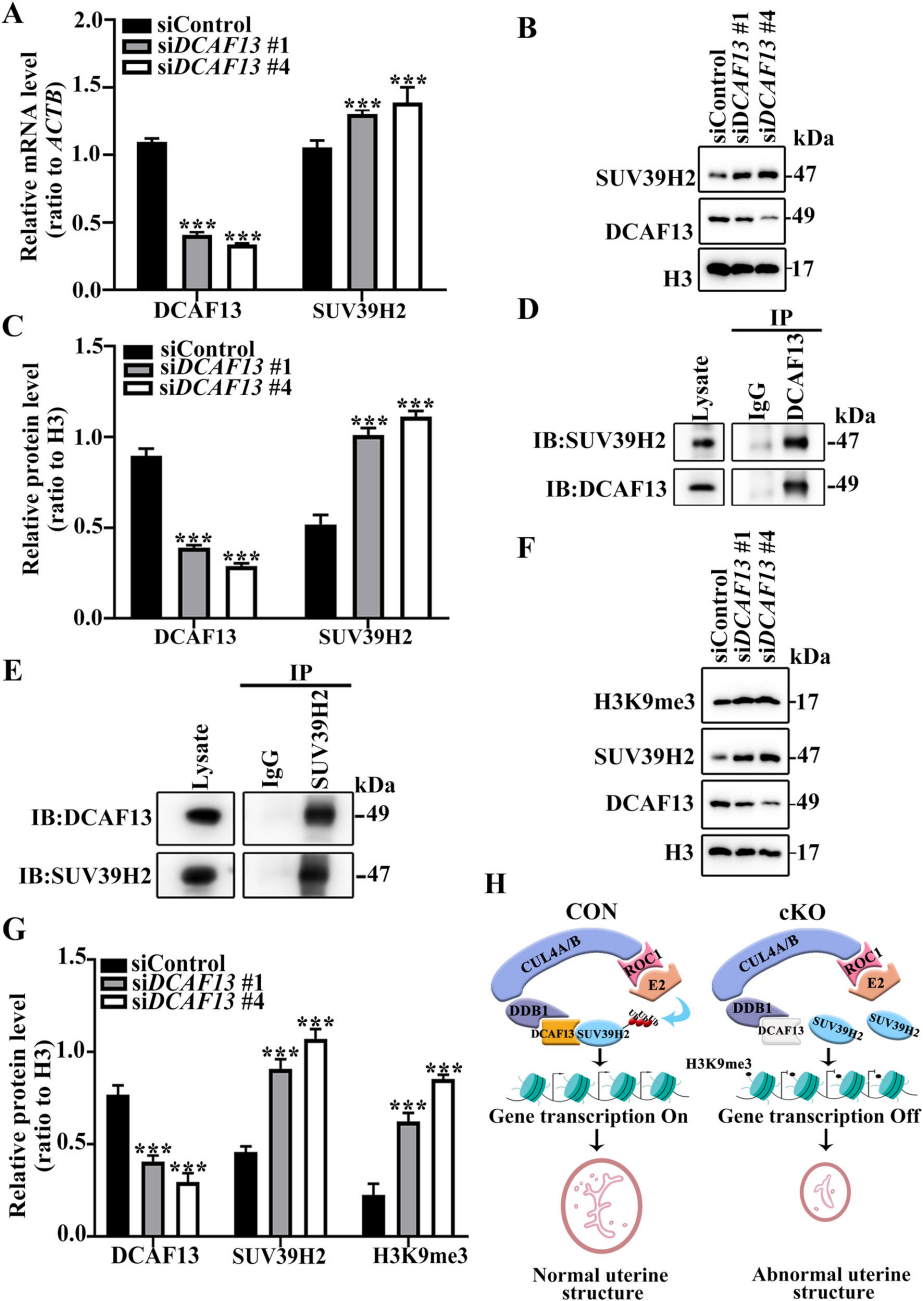
DCAF13 regulates H3K9me3 level by interacting with SUV39H2. **A** qPCR results show that the mRNA levels of *SUV39H2* in si*DCAF13*#1 and si*DCAF13*#4 groups are significantly higher compared to control cells. **B** Western Blotting was used to detect the expression level of SUV39H2. **C** After transfecting si*DCAF13*#1 and si*DCAF13*#4 into Hela cells, the expression of the SUV39H2 protein was significantly upregulated. **D**, **E**. The interaction between DCAF13 protein and SUV39H2 protein was demonstrated through IP and Western Blotting. **F** Analysis of the H3K9me3 level via Western Blotting after si*DCAF13*#1 and si*DCAF13*#4 transfection. **G** The quantification of H3K9me3 detected by Western Blotting. ***P < 0.001. **H** A proposed model suggests that DCAF13 plays an essential role in maintaining the morphology, structure, and function of the uterus. In normal circumstances, DCAF13 facilitates the polyubiquitination of SUV39H2, which helps in maintaining low levels of H3K9me3 and promotes the transcription of necessary genes for normal uterine function. However, the knockdown of DCAF13 leads to the accumulation of SUV39H2, resulting in elevated H3K9me3 levels, and inhibits transcription, ultimately leading to abnormal uterine morphology, structure, and function.

We subsequently transfected Hela cells with *siDcaf* 13#1 and *siDcaf*13 #4 and detect the level of *SUV39H2* by qPCR. The results showed that *SUV39H2* level was significantly increased in DCAF13-knockdown cells compared with control cells (Fig. 6A). Western Blotting results also showed that SUV39H2 in DCAF13 knockdown cells was significantly upregulated, consistent with the qPCR results (Fig. 6B, C). These findings suggest that the experimental results obtained using Hela cells can reflect the situation in the mouse uterus to some extent. Therefore, Hela cells can be used for subsequent experiments.

DCAF13 has been reported to interact with SUV39H1 to promote embryonic development by maintaining a low level of intracellular H3K9me3 via ubiquitination degradation through the proteasome pathway [22]. Therefore, we hypothesized that there may be an interaction between DCAF13 and SUV39H2. To test this hypothesis, we conducted protein immunoprecipitation experiments at the cellular level. We lysed Hela cells and used DCAF13 and SUV39H2 antibodies to verify their interaction. Western Blotting revealed that the interaction between DCAF13 and SUV39H2 existed, regardless of which antibody was used (Fig. 6D, E). These findings provide conclusive evidence that there is indeed an in vivo interaction between DCAF13 and SUV39H2.

We then further investigated whether the interplay between DCAF13 and SUV39H2 could modulate intracellular H3K9me3 level. We collected and lysed Hela cells that had been transfected with *Dcaf*13 siRNA for 48 h, and detected the levels of intracellular H3K9me3 through Western Blotting. Our results demonstrate a significant increase in the H3K9me3 level in DCAF13-knockdown Hela cells compared to control cells (Fig. 6F, G). These results suggest that knocking down DCAF13 disrupts the interplay between DCAF13 and SUV39H2, leading to a rise in intracellular H3K9me3 levels, ultimately resulting in abnormal uterine morphology, structure, and function (Fig. 6H).

## Discussion

In this study, we investigated the function and mechanism of DCAF13 in female reproduction using *Dcaf13* cKO mice. We found that *Dcaf13* cKO female mice were completely infertile despite having normal ovarian structure and function. *Dcaf13* cKO embryos could develop to the blastocyst stage but were unable to implant, and the expression of implantation markers was significantly reduced. The uteri of *Dcaf13* cKO mice were particularly small and thin. Compared to the control group, the proliferation of the uterine endometrium of *Dcaf13* cKO mice was insufficient, along with increased DNA damage and apoptosis of stromal cells. The expression levels of estrogen receptors and estrogen-responsive genes in the uterine endometrium of *Dcaf13* cKO mice were significantly decreased, while progesterone receptor expression was abnormally high in epithelial cells and abnormally low in stromal cells, leading to disarray in the expression levels of progesterone response genes. Further studies revealed that the expression of SUV39H2 in the uteri of *Dcaf13* cKO mice was upregulated, resulting in an increase in H3K9me3 levels. Immunoprecipitation showed that DCAF13 could interact with SUV39H2. Deletion of DCAF13 disrupted this interaction, leading to an increase in H3K9me3 levels. These results suggest that DCAF13 is necessary for successful implantation and maintenance of pregnancy.

DCAF13 is also known as WDSOF1 (WD Repeat and SOF Domain-containing Protein 1) encoded by *Dcaf13* [44]. This gene is located in a chromosomal region with high-frequency amplification in many tumors, namely 8q22.3 [45, 46]. DCAF13 belongs to the DCAF family of proteins. In addition to DCAF13, this family also includes DCAF1, DCAF2, and DCAF11, all of which contain a seven-repeat closed-loop helix domain (WD40) [47, 48], which plays important roles. Previous studies have reported that DCAF13 is a CRL4 adapter required for preimplantation embryonic development [22]. In this study, we found that DCAF13 is essential for the development and differentiation of the uterus as it matures. When DCAF13 is missing, the uterus becomes smaller and thinner.

Estrogen and progesterone work together to regulate endometrial cell proliferation and differentiation, which are essential for embryo attachment and pregnancy. The mechanism of estrogen regulation involves estrogen binding to ESR1 in endometrial stromal cells, which then regulates paracrine factors such as epidermal growth factor and insulin-like growth factor to impact the proliferation and differentiation of endometrial epithelial cells [49]. Additionally, estrogen directly promotes endometrial epithelial cell differentiation through ESR1 activation in the epithelial cells [37]. On the one hand, progesterone binds to PGR in endometrial epithelial cells to promote epithelial cell differentiation and inhibit the proliferation of endometrial epithelial cells induced by estrogen, while also encouraging stromal cell proliferation in endometrial stromal cells [50]. Our data showed that estrogen and progesterone levels were normal in *Dcaf13* cKO mice. However, ESR1 expression in epithelial and stromal cells of the *Dcaf13* cKO mice were significantly reduced. This reduction in ESR1 expression compromised the proliferation and differentiation of epithelial and stromal cells, preventing embryo implantation. Additionally, PGR expression was significantly reduced in endometrial stromal cells of *Dcaf13* cKO mice, which was also unfavorable for uterine stromal cell proliferation. Moreover, the transcription level of estrogen-responsive genes was decreased, while the transcription of progesterone-responsive genes was disordered in the uterus of *Dcaf13* cKO mice. These dysregulations disrupted the proliferation of endometrial cells, together with the increased DNA damage, which in turn led to apoptosis of endometrial cells, resulting in the tiny uteri of the *Dcaf13* cKO mice, ultimately the failure of embryo implantation. This suggests that DCAF13 is critical for the proliferation and differentiation of mouse endometrial cells.

It has been reported that DCAF13 can interact with SUV39H1 protein. Upon interaction, SUV39H1 is ubiquitinated and degraded via the proteasome pathway, which helps maintain low levels of intracellular H3K9me3 and promotes embryo development [22]. SUV39H2 is a homolog of SUV39H1, and both of them have the SET and Chromo structural domains [51], with a high degree of amino acid sequence identity [43]. SUV39H2, like SUV39H1, is an important histone lysine methyltransferase that functions to trimethylate H3K9 [52]. H3K9me3 is associated with gene repression and plays a central role in heterochromatin formation, regulation of telomere length, and repetitive gene silencing [53, 54]. In addition, SUV39H1 and SUV39H2 are also markers for the development of different cancers, with high or low expression of SUV39H1 posing risks, while high expression of SUV39H2 is often detrimental in most cases.

For preimplantation embryonic development, CRL4-DCAF13-mediated SUV39H1 degradation is essential for polyubiquitination and proteasomal degradation, as well as subsequent removal of H3K9me3. This process facilitates progressive genome reprogramming and contributes to zygotic gene expression [25]. In current study, we discovered that in the uterus, DCAF13 targeted SUV39H2 instead of SUV39H1 to remove H3K9me3.In the uterus of *Dcaf13* cKO mice, DCAF13 was unable to interact with SUV39H2. As a result, SUV39H2 could not be degraded through the ubiquitin-proteasome pathway, leading to a large accumulation of SUV39H2 in the cells. This accumulation caused H3K9me3 levels to rise, inhibiting the transcription of related genes and ultimately affecting the morphology, structure, and function of the mouse uterus. This suggests that DCAF13 is an important component of the uterine morphology and structure in mice, and is an essential substrate for the CRL4 complex to function by interacting with SUV39H2. However, further investigation is needed to fully understand the ubiquitination-proteasome degradation process of SUV39H2.

## Materials and methods

### Animals

All the mice used in the experiments had a C57BL/6 background. They were housed under a 12 h light/dark cycle at the Experimental Animal Center of Shandong Normal University. Three *Dcaf13*^*fl/fl*^ female mice (a gift from Prof. Hengyu Fan of Zhejiang University) were bred to expand the population. The offspring were co-caged with progesterone receptor (*Pgr*) *cre*^*+/−*^ (The Jackson laboratory, Ellsworth, CA, USA) male mice, resulting in the production of *Pgr cre*^*+/−*^; *Dcaf13*^*fl/wt*^ mice and *Dcaf13*^*fl/wt*^ mice. *Pgr cre*^*+/−*^; *Dcaf13*^*fl/wt*^ mice were then co-housed in the same cage with *Dcaf13*^*fl/fl*^ mice and gave birth to *Pgr cre*^*+/−*^; *Dcaf13*^*fl/fl*^ (cKO) female mice. All animal experiments were conducted following the “Guidelines for the Care and Use of Laboratory Animals” of Shandong Normal University and were authorized by the Animal Ethics Committee of Shandong Normal University.

### Quantitative polymerase chain reaction (qPCR)

RNA was extracted from the samples using the TRIzol reagent (Tiangen, Beijing, China) in accordance with the manufacturer’s instructions. The extracted RNA was subsequently reverse transcribed into complementary DNA (cDNA) using a cDNA synthesis kit (Yeasen, Shanghai, China). For the qPCR analysis, a LightCycler^®^96 instrument (Roche Pharma Ltd, Basel, Switzerland) was used. The SYBR Green Master Mix (Yeasen), along with specific primers (as listed in Supplementary Table 1) for the target genes was utilized. The qPCR procedure was carried out under the following conditions: an initial denaturation at 95 °C for 10 min; followed by 40 cycles of denaturation at 95 °C for 10 s, annealing at 58 °C for 20 s, and extension at 72 °C for 20 s; The procedure was ended with a final extension step at 72 °C for 5 min. *Actb* was used as an internal control. The data analysis was performed using the 2^−ΔΔCt^ formula.

### Western blotting

Protein extraction was carried out using RIPA (Beyotime Biotechnology Co., Ltd, Shanghai, China) from mouse tissues and Hela cells. Next, 15 mg of protein was resolved using SDS-PAGE electrophoresis (Solarbio Science &Technology Co., Ltd., Beijing, China) and transferred onto a polyvinylidene fluoride membrane (Millipore, Bedford, MA, USA). The membrane was then blocked with 5% nonfat dry milk at room temperature for 1 h. After blocking, the membrane was incubated overnight at 4 °C with primary antibodies, namely DCAF13 (1:1000 dilution in 1% BSA, Abcam, Cambridge, UK), SUV39H2 (1:1000 dilution in 1% BSA, Abcam), H3K9me3 (1:1000 dilution in 1% BSA, HuaBio Biotechnology Co., LTD, Hangzhou, China), or β-actin (1:5000 dilution in 1% BSA, HuaBio). Following this, the membrane was washed three times with TBST and a horseradish peroxidase enzyme-linked secondary antibody (1:5000 dilution in 1% BSA, HuaBio) was added and incubated at room temperature for 1 h. The membrane was subsequently rinsed three times with TBST. The protein bands were visualized using an enhanced chemiluminescence (ECL) Western Blotting Kit (Millipore, Bedford, MA, USA) and an ECL Western Blotting analysis system (Tanon 5500 Multi, Shanghai, China). To analyze the relative intensities of the bands, the Quantiscan software (Biosoft, GreatShelford, UK) was utilized for analysis and normalization to β-actin within the same blot.

### Immunohistochemistry

The mouse uterus and ovary tissue specimens were fixed with 4% paraformaldehyde (Servicebio Biotechnology Co., Ltd., Wuhan, China) for 24 h, dehydrated using graded ethanol (ranging from 55% to 100%), and embedded in paraffin. The samples were then sliced to a thickness of 5 μm using a paraffin microtome (Leica, Wetzlar, Germany). Subsequently, the sections were deparaffinized with xylene, dehydrated with decreasing concentrations of alcohol (from 100% to 75%), and then subjected to high-pressure repair in citric acid antigen retrieval solution (Beyotime). After several washes with phosphate-buffered saline powder (PBS) (Servicebio), the sections were blocked in 3% hydrogen peroxide in the dark for 15 min to block endogenous peroxidase activity. The primary antibody diluent (Servicebio) was used to block the sections for 1 h. The sections were then incubated with primary antibodies overnight at 4 °C. The primary antibodies used were DCAF13 (1:200 dilution, Abcam), SUV39H2 (1:200 dilution, Abcam), H3K9me3 (1:200 dilution, HuaBio), PGR (1:200 dilution, HuaBio), ERα (1:200 dilution, Proteintech Group, Inc, Wuhan, China), Ki67 (1:500 dilution, Servicebio), γ-H2AX (1:500 dilution, Bioss Biotechnology Co., Ltd, Beijing, China), and E-cadherin (1:500 dilution, Cell Signaling Technology, Inc, Boston, United States). After washing the sections three times with PBS, they were incubated with goat anti-rabbit secondary antibody (1:200, ZSGB-BIO Biotechnology Co., Ltd., Beijing, China) for 1 h at room temperature, and then washed three times with PBS. Finally, the sections were stained with a DAB kit (ZSGB-BIO) to produce a visual positive signal, re-stained with hematoxylin (Solarbio), and sealed with neutral gum (Sinopharm Chemical Reagent Co., Ltd, Shanghai, China). An automated digital slide scanner (Panoramic MIDI II, 3Dhistech Ltd, Budapest, Hungary) was used for final photography, and Image-Pro Plus version 6.0 (Media Cybernetics, Silver Spring, MD, USA) was employed for quantitative analysis of immunohistochemical staining.

### Histological analysis

A portion of the paraffin block was sectioned into 5 μm slices using a paraffin microtome (Leica). Subsequently, the slices were subjected to dewaxing and dehydration using alcohol as mentioned for immunohistochemistry. The sections were then immersed in water and stained with hematoxylin (Solarbio) for an appropriate duration, followed by microscopic examination. After color observation, a brief differentiation with hydrochloric acid was performed, and the sections were examined under the microscope again. Finally, the sections were stained with eosin staining solution (Solarbio), dehydrated twice using alcohol and xylene, and mounted with neutral gum (Sinopharm).

### Visualizing implantation sites

On the day following the cohabitation of male and female mice, the vaginal plug was assessed, and its discovery date was recorded as day post-coitum (dpc) 0.5. Afterward, on dpc 4.5, a 10 IU intravenous injection of trypan blue (Beyotime) was administered through the tail veins of female mice. The mice were euthanized after receiving treatment for 10-15 min, and their uteri were extracted, examined, photographed, and quantified.

### Superovulation

To induce superovulation, female mice at postnatal day 28 were selected. Pregnant horse serum gonadotropin (Ningbo Sansheng Biological Technology Co., Ningbo, Zhejiang, China) was injected into each female mouse at a dose of 5 IU, followed by an injection of human chorionic gonadotropin (Ningbo Sansheng) into each mouse (5 IU) 48 h later. 14–16 h later, oocytes were retrieved from the oviduct. The oocytes with cumulus cells were then transferred to MII medium (Sigma-Aldrich, Saint Louis, MO, USA) containing hyaluronic acid (Sigma-Aldrich) and washed repeatedly. Subsequently, the oocytes were counted and photographed under a fully automated inverted microscope (Leica).

### Hormone analysis

Blood samples were collected from mice via cardiopuncture. The samples were then centrifuged, and the resulting serum was stored at −20 °C. The levels of P4 and E2 in the serum were analyzed as previously reported using enzyme-linked immunosorbent assay (ELISA) kits (Prog ELISA Kit and E2 ELISA Kit, Cusabio Biological Engineering Co., Ltd, Wuhan, China) according to the manufacturer’s instructions. Optical density measurements were obtained at a wavelength of 450 mm using a microplate reader (Molecular Devices, San Jose, CA, USA). Mouse progesterone and estrogen concentrations were determined by generating a standard curve. The coefficients of variation (CVs) for both intra- and inter-assay were less than 15%. The minimum detectable concentration of the progesterone kit was 0.3 ng/ml, and for estrogen it was 25 pg/ml.

### The construction of RNA sequencing libraries and the subsequent sequencing

Uterine samples were collected from three female *Dcaf13*^*fl/fl*^ and cKO mice. RNA was extracted using TRIzol reagent (Thermo Fisher Scientific) and stored at −80 °C. The quality of the RNA samples was assessed using the Bioanalyzer Agilent 2200. RNA samples with a RNA integrity number greater than 7.0 were considered suitable for creating cDNA libraries. The following steps were used to construct the cDNA libraries: First, poly-A containing mRNA was separated from 1 µg total RNA using oligomerized (dT) magnetic beads. The separated mRNA was then cleaved into fragments of 200–600 bp using divalent cations at 85 °C for 6 min. Then the first and second strands of cDNA were synthesized from the cleaved RNA. Subsequently, the cDNA fragments were end-repaired, A-tailed, and connected with an index adapter. The linked cDNA product was purified, and the second-strand cDNA was removed using uracil DNA glycosylase (Thermo Fisher Scientific). The purified first-strand cDNA was then amplified by PCR to generate a cDNA library. The quality of the library was assessed using the Agilent 2200, followed by 150 bp paired-end sequencing on the NovaSeq 6000 platform (Novogene Corp., Sacramento, CA).

### RNA sequencing mapping and identification of differentially expressed genes (DEGs)

Before performing read mapping, the raw reads were processed to obtain clean reads by removing adapter sequences and low-quality reads. These clean reads were then aligned to the mouse genome (mm10, NCBI) using Hisat2. DEG analysis was done using the DESeq2 algorithm. After statistical analysis, genes with a *P*-value < 0.05 or a fold change > 2 or < 0.5 were considered to be differentially expressed.

### Gene ontology (GO) and pathway analysis

To understand the biological significance of the DEGs, GO analysis was conducted. GO annotations were downloaded from NCBI (http://www.ncbi.nlm.nih.gov/), UniProt (http://www.uniprot.org/), and Gene Ontology (http://www.geneontology.org/). Significant GO terms were determined based on a *P*-value < 0.05 and FDR < 0.05, using the Fisher’s exact test. Pathway analysis was performed to identify significant pathways associated with the DEGs using the KEGG database. The Fisher’s exact test was used to determine the significance, with a threshold set at *P*-value < 0.05.

### Small interfering RNA transfection (siRNA)

Human cervical cancer (HeLa) cells were cultured in DMEM/F12 (Thermo Fisher Scientific, Waltham, MA, USA) supplemented with 10% fetal bovine serum (Biological Industries, Beit She’an, Israel) at 37 °C in a CO2 incubator. The culture medium was refreshed every 48 h, and cells were examined daily under an inverted microscope (Olympus, Tokyo, Japan). Once the cell density reached 40%, the cells were transfected with siRNA (Ribobio Co., LTD, Guangzhou, China) using Lipofectamine 3000 (Thermo Fisher Scientific) in Opti-MEM (Thermo Fisher Scientific), following the manufacturer’s instructions. The culture medium was replaced after 4 to 6 h, and the cells were harvested after 48 h of transfection.

### Immunoprecipitation

The Hela cell (1 × 10^7^) were collected and centrifuged at 12,000 × *g* for 10 s at 4 °C. The supernatant was removed, and a RIPA buffer (Beyotime) containing protease inhibitors was added. The sample was placed in an ice bath for 30 min and fully cracked by multiple shocks during the ice bath. The sample was then centrifuged at 12,000 × *g* for 20 min. The supernatant was collected. Additionally, a small amount of supernatant was transferred and the corresponding volume of 5×SDS (Beyotime) was added. This mixture was heated at 95 °C for 5 min, centrifuged at 12,000 × *g* for 3 min after returning to room temperature, and then stored at −20 °C. as a Lysate group. The remaining supernatant was evenly divided and appropriate amounts of IgG (Beyotime) and target protein antibody of the same properties were added, respectively. The samples were incubated at 4 °C for 2 h. Then the Protein A/G beads (Beyotime) were added, evenly mixed, and left overnight at 4 °C. Subsequently, the RIPA Buffer was added and centrifuged at 4 °C at 2000 × *g*, for 1 min each time, repeating this process 6 to 7 times. The supernatant was discarded, and the corresponding volume of 2×SDS was added to the sample. It was heated at 95 °C. for 5 min, and then centrifuged at 12000 × *g* for 3 min after returning to room temperature. The sample was saved as an IP group at −20 °C. Finally, the interaction between proteins was determined by Western Blotting assay.

### Statistical analysis

All the data were statistically analyzed using GraphPad Prism (Version Animals 8.2.1 for Windows, GraphPad Software, San Diego, CA, USA). The differences between groups were analyzed using a two-tailed Student’s *t*-test. The data are presented as the mean ± SEM. Values were considered statistically significant if *P* < 0.05. All experiments were repeated at least three times.

## Supplementary information

The sequence list of the primers.

FIG S

WB Raw data

## Data Availability

The underlying data supporting the results of this study will be made available upon reasonable request to the corresponding author. This is done in the spirit of open science and allows others in the field to validate our results and use the data for further exploration.
