# Supplementary material for: DCAF13 is essential for mouse uterine function and fertility
Source: Cell Death Discov. 2025 Aug 1;11:359. doi: 10.1038/s41420-025-02583-w (PMC12316921; doi:10.1038/s41420-025-02583-w)
Supplement: Supplementary file 1 — The sequence list of the primers. [file 41420_2025_2583_MOESM1_ESM.docx]

**Supplementary Table 1. The sequence list of the primers.**

| **Gene** | **Sequences** |
| --- | --- |
| *Bmp2* | GCTTCCGTCCCTTTCATTTCT |
|  | AGCCTCCATTTTTGGTAAGGTTT |
| *Lif* | GCCCCAGAAGTAAAACCTTCAG |
|  | CCTTCCATTTCTCTCCATTCCAA |
| *Wnt4* | AGACGTGCGAGAAACTCAAAG |
|  | GGAACTGGTATTGGCACTCCT |
| *Wnt7a* | GGCTTCTCTTCGGTGGTAGC |
|  | TGAAACTGACACTCGTCCAGG |
| *Foxa2* | CCCTACGCCAACATGAACTCG |
|  | GTTCTGCCGGTAGAAAGGGA |
| *Spink3* | TTTGGCCCTGCTGAGTTTAGC |
|  | TGGCATAAGTAATTCCGTCAGTC |
| *Wfdc3* | GAGAGCACGCATTGAGAGGTG |
|  | ACAGGATTCGTCTCCGGTACA |
| *Cdh1* | CAGGTCTCCTCATGGCTTTGC |
|  | CTTCCGAAAAGAAGGCTGTCC |
| *Krt18* | TCAAGATCATCGAAGACCTGAGG |
|  | GCGCATGGCTAGTTCTGTC |
| *Ki67* | ATCATTGACCGCTCCTTTAGGT |
|  | GCTCGCCTTGATGGTTCCT |
| *Esr1* | CCTCCCGCCTTCTACAGGT |
|  | CACACGGCACAGTAGCGAG |
| *Muc1* | GGCATTCGGGCTCCTTTCTT |
|  | TGGAGTGGTAGTCGATGCTAAG |
| *Ltf* | TGAGGCCCTTGGACTCTGT |
|  | ACCCACTTTTCTCATCTCGTTC |
| *Lcn2* | GGGAAATATGCACAGGTATCCTC |
|  | CATGGCGAACTGGTTGTAGTC |
| *Clca3* | CTGTCTTCCTCTTGATCCTCCA |
|  | CGTGGTCTATGGCGATGACG |
| *Pgr* | GGGGTGGAGGTCGTACAAG |
|  | GCGAGTAGAATGACAGCTCCTT |
| *Hand2* | GAGAACCCCTACTTCCACGG |
|  | GACAGGGCCATACTGTAGTCG |
| *Nr2f2* | CATCGAGAACATTTGCGAACTG |
|  | GTCGGCTGACATGGGTGAAG |
| *Lrp2* | AAAATGGAAACGGGGTGACTT |
|  | GGCTGCATACATTGGGTTTTCA |
| *Areg* | GGTCTTAGGCTCAGGCCATTA |
|  | CGCTTATGGTGGAAACCTCTC |
| *Ihh* | CTCTTGCCTACAAGCAGTTCA |
|  | CCGTGTTCTCCTCGTCCTT |
| *Suv39h2* (mouse) | CCAACCAGGCACTCCCATCTACG |
|  | GGGATACGTTTCCATATCGAGCTGC |
| *Dcaf13* | TTTCCTGTAGACAAAAGTCGAAGCA |
|  | GCATTAGCTTTCCACAGGCG |
| *SUV39H2* (human) | TGGGGTGTAAAGACCCTTGTG |
|  | ATTCCCTTGTTGTCATAGAAC |
| *Actb* (mouse) | TGTTACCAACTGGGACGACA |
|  | GGGGTGTTGAAGGTCTCAAA |
| *ACTB* (human) | TGTTACCAACTGGGACGACA |
|  | GGGGTGTTGAAGGTCTCAAA |
