# Supplementary material for: DCAF13 is essential for mouse uterine function and fertility
Source: Cell Death Discov. 2025 Aug 1;11:359. doi: 10.1038/s41420-025-02583-w (PMC12316921; doi:10.1038/s41420-025-02583-w)
Supplement: Supplementary file 2 — FIG S [file 41420_2025_2583_MOESM2_ESM.docx]

FIG S1


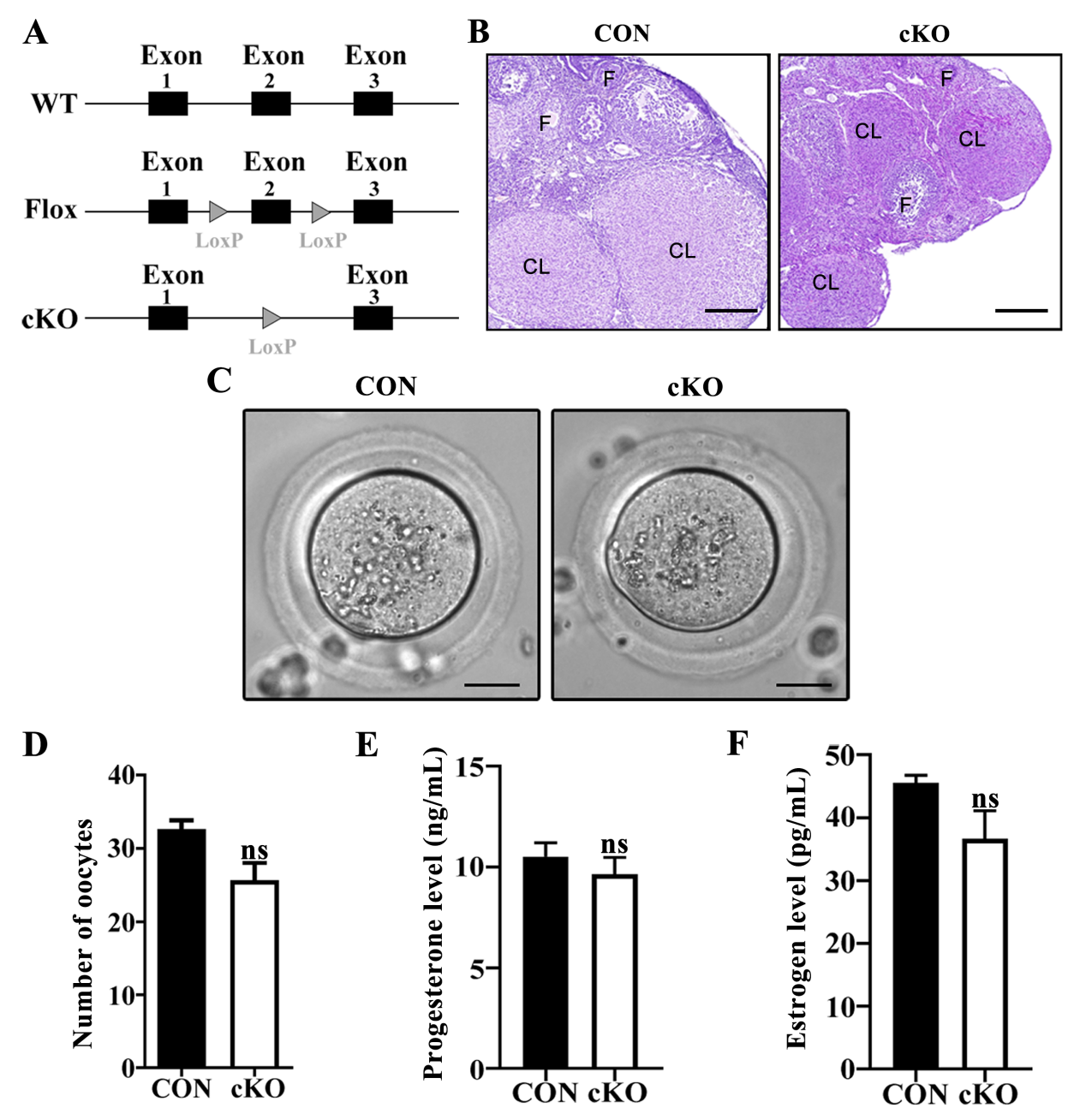


**Figure S1. *Dcaf13* cKO female mice displayed infertility despite having normal ovarian structure and function.**

**A.** The construction principle of *Dcaf13* cKO mice. **B.** HE staining revealed the structure of the ovaries. CL: corpus luteum, F: follicles. The scale is 250 μm. **C.** The morphology of oocytes after superovulation. The scale is 250 μm. **D.** No significant difference was observed in the number of oocytes. **E and F.** There was no statistical variance in the serum levels of progesterone or estrogen between control and *Dcaf13* cKO mice.

FIG S2


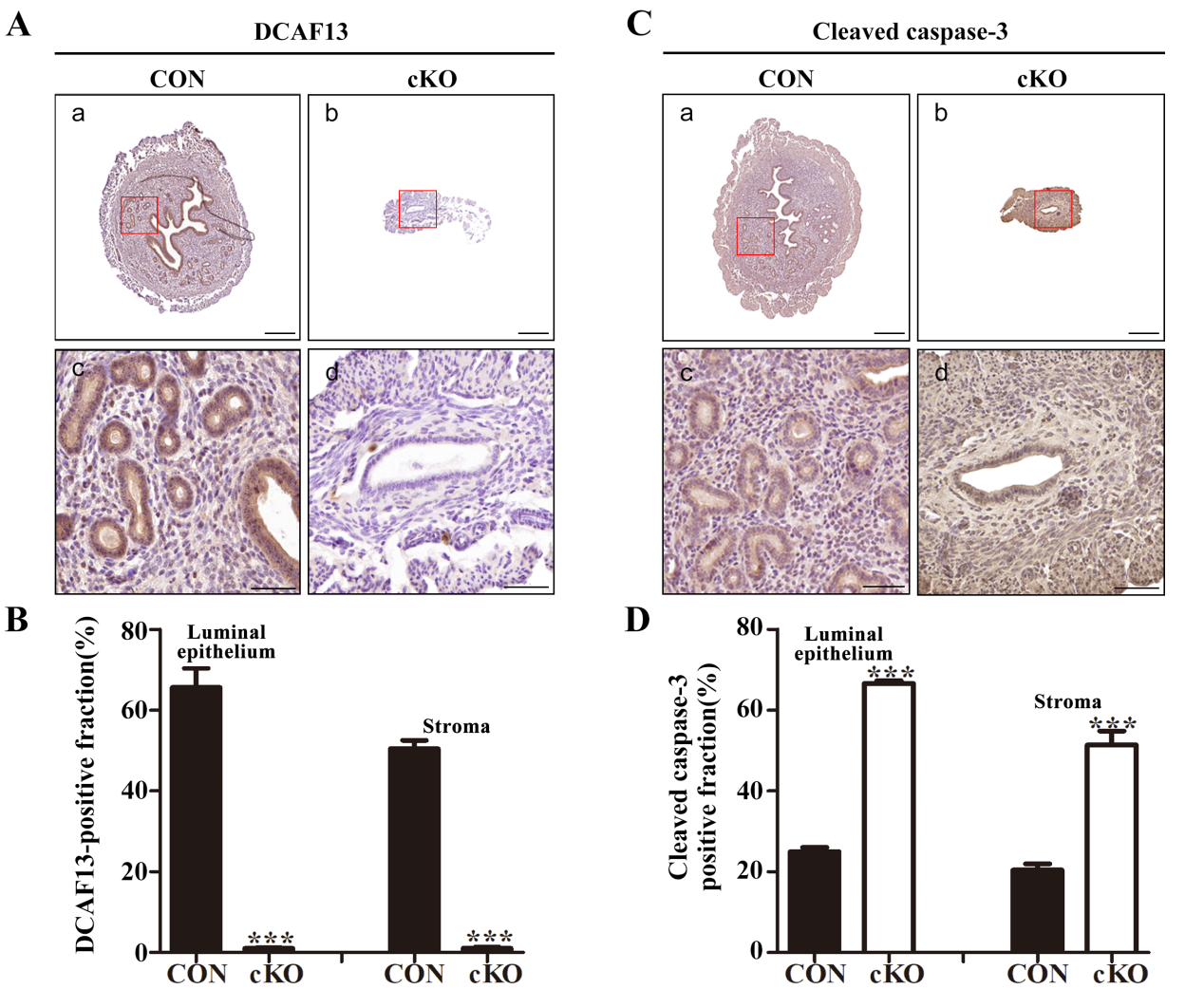


**Figure S2** **Verified the knockout efficiency in the uterus of *Dcaf13* cKO female mice and the expression of Cleaved caspase-3.**

**A.** The expression levels of DCAF13 in the uterus of control and *Dcaf13* cKO mice. **B.** Immunohistochemical quantification showed that DCAF13 expression was significantly decreased in *Dcaf13* cKO mice. **C.** The expression levels of Cleaved caspase-3 in the uterus of control and *Dcaf13* cKO mice. **D.** Immunohistochemical quantification showed that Cleaved caspase-3 expression was significantly increased in *Dcaf13* cKO mice. The enlarged images in Fig. c and Fig. d correspond to the red boxes in images Fig. a and Fig. b, with scale measurements of 200 μm for Fig. a and Fig. b, and 50 μm for Fig. c and Fig. d. LE: luminal epithelium, S: stroma. ****P*<0.001.

FIG S3


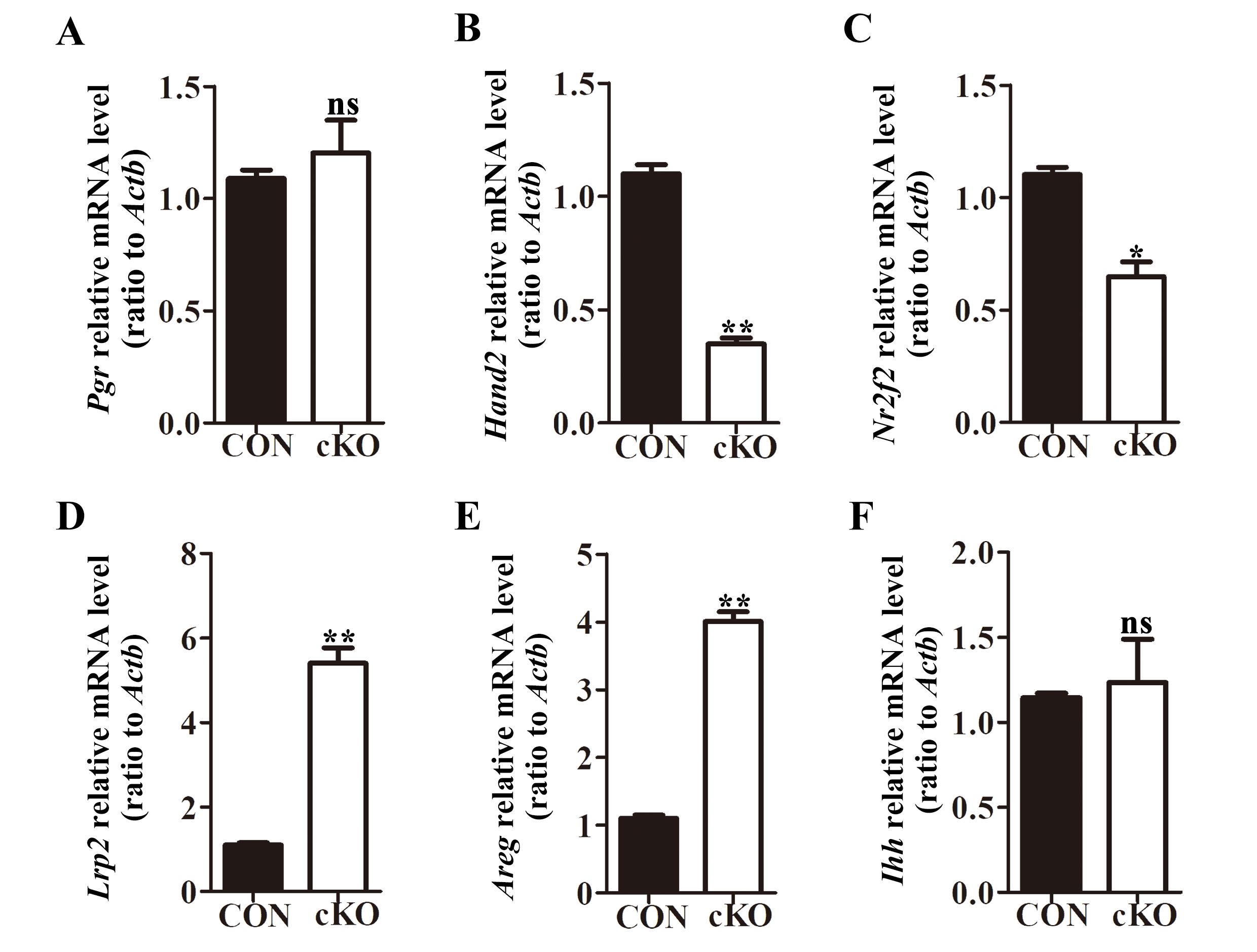


**Figure S3. Disorders of progesterone-responsive genes in the uterus of *Dcaf13* cKO mice**.

**A.** The qPCR analysis revealed no significant difference in the *Pgr* level in the uterus of *Dcaf13* cKO mice compared to that of the control mice. **B-F.** The qPCR experiments detected the mRNA levels of progesterone-responsive genes. The results revealed that compared to the control group, the mRNA levels of *Hand2* and *Nr2f2* in the uterus of cKO mice were significantly decreased. Conversely, *Lrp2* and *Areg* levels were significantly increased, while *Ihh* level showed no statistical difference. * *P*<0.05, ***P*<0.01.

FIG S4


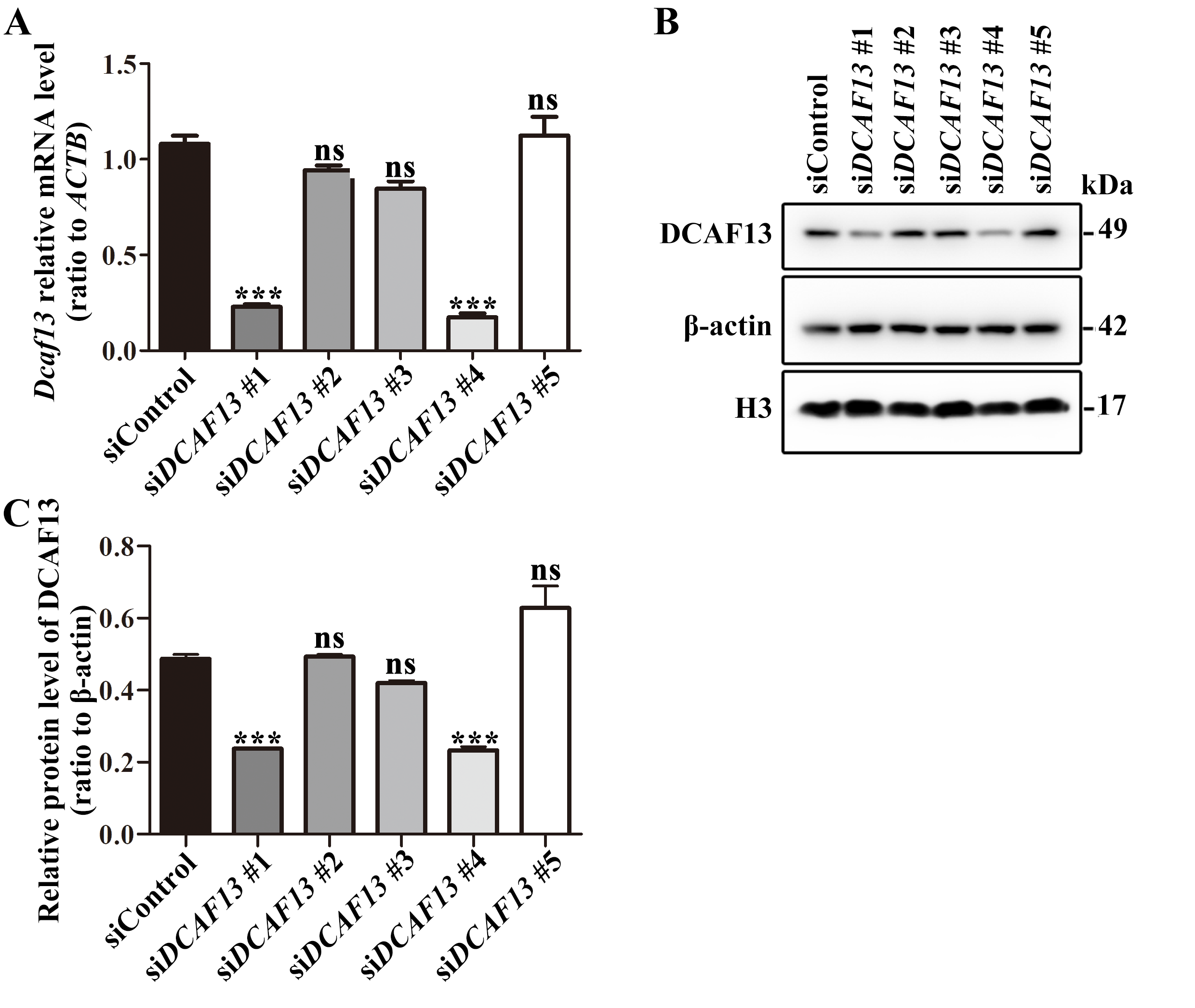


**Figure S4. Hela cells were validated for DCAF13 knockdown efficiency.**

**A.** qPCR assay was performed to detect the knockdown efficiency of si*DCAF13*. **B.** Western Blotting assay was conducted to detect the knockdown efficiency of si*DCAF13*. **C.** The quantification of the Western Blotting results reveals that si*DCAF13*#1 and si*DCAF13*#4 exhibited significant knockdown efficiencies. ****P*<0.001.
