# Supplementary figures and images for: DCAF13 is essential for mouse uterine function and fertility (part 2 of 2)
Source: Cell Death Discov. 2025 Aug 1;11:359. doi: 10.1038/s41420-025-02583-w (PMC12316921; doi:10.1038/s41420-025-02583-w)

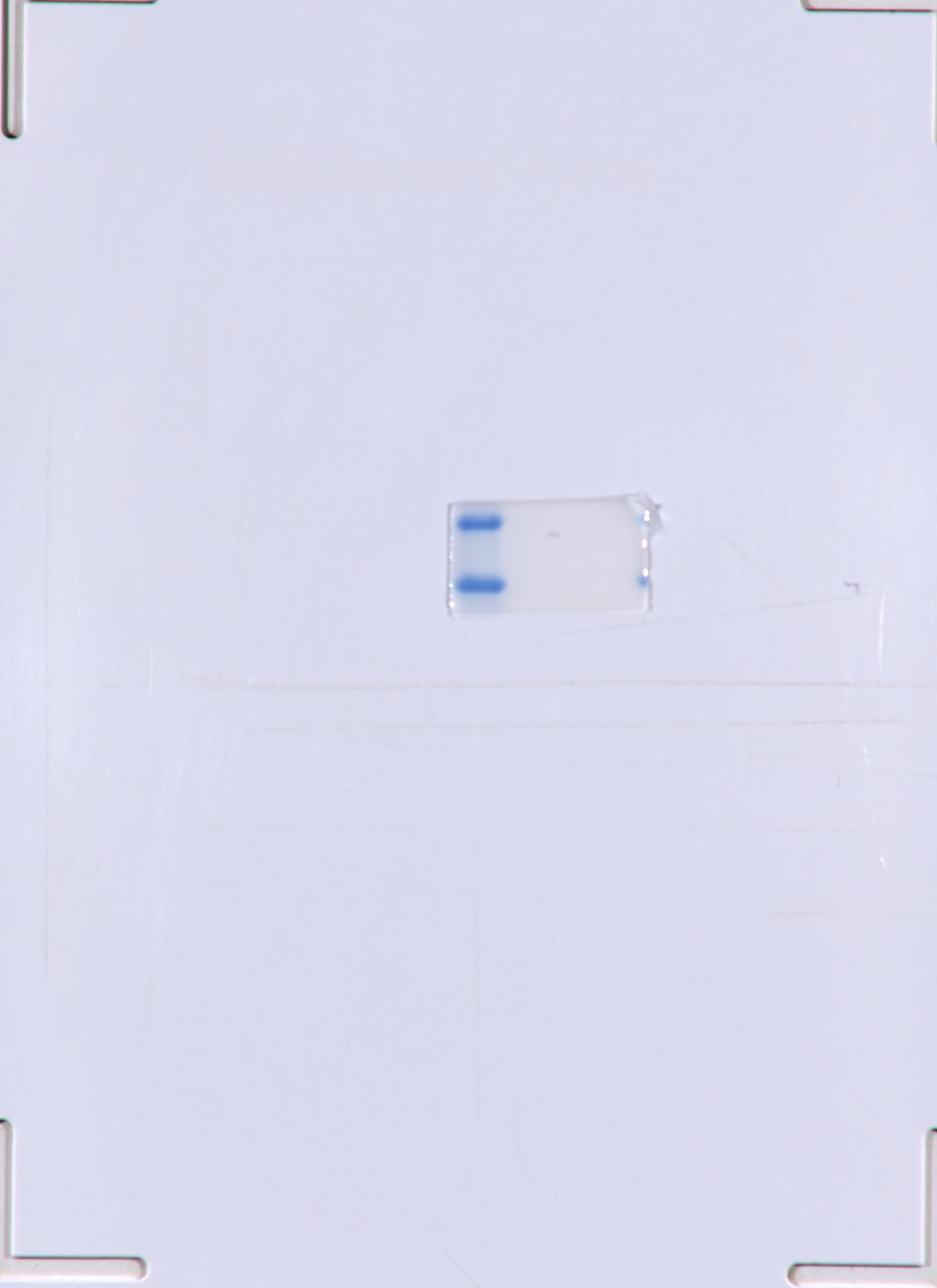

Supplement: Supplementary file 3 — WB Raw data [file 41420_2025_2583_MOESM3_ESM.zip › Figure 6 Panel D/suv39h2 ip 2022.04.03_11.26.14_Ch/suv39h2 ip 2022.04.03_11.26.14_Ch-Marker.jpg]

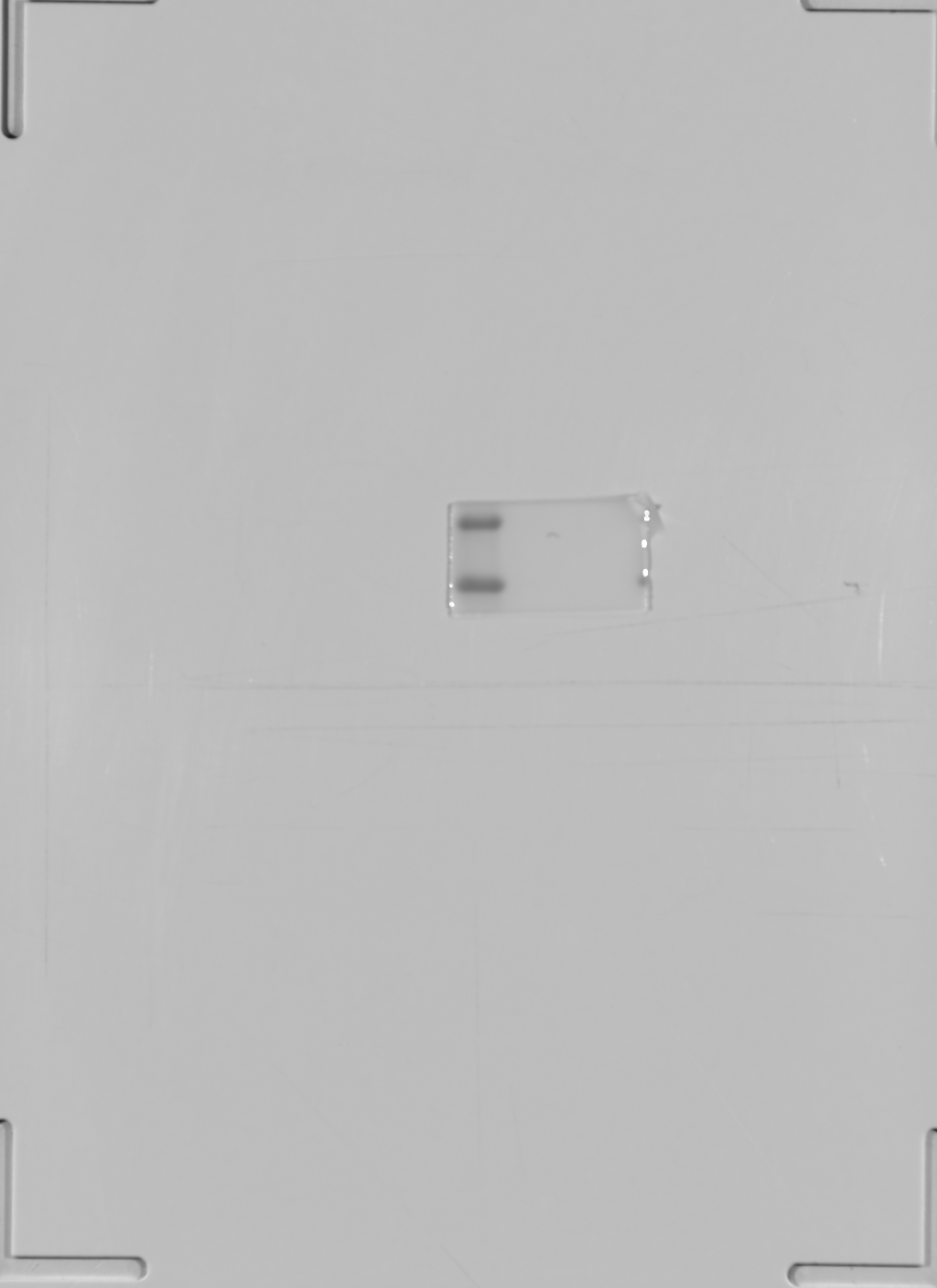

Supplement: Supplementary file 3 — WB Raw data [file 41420_2025_2583_MOESM3_ESM.zip › Figure 6 Panel D/suv39h2 ip 2022.04.03_11.26.14_Ch/suv39h2 ip 2022.04.03_11.26.14_Ch-Marker.tif]

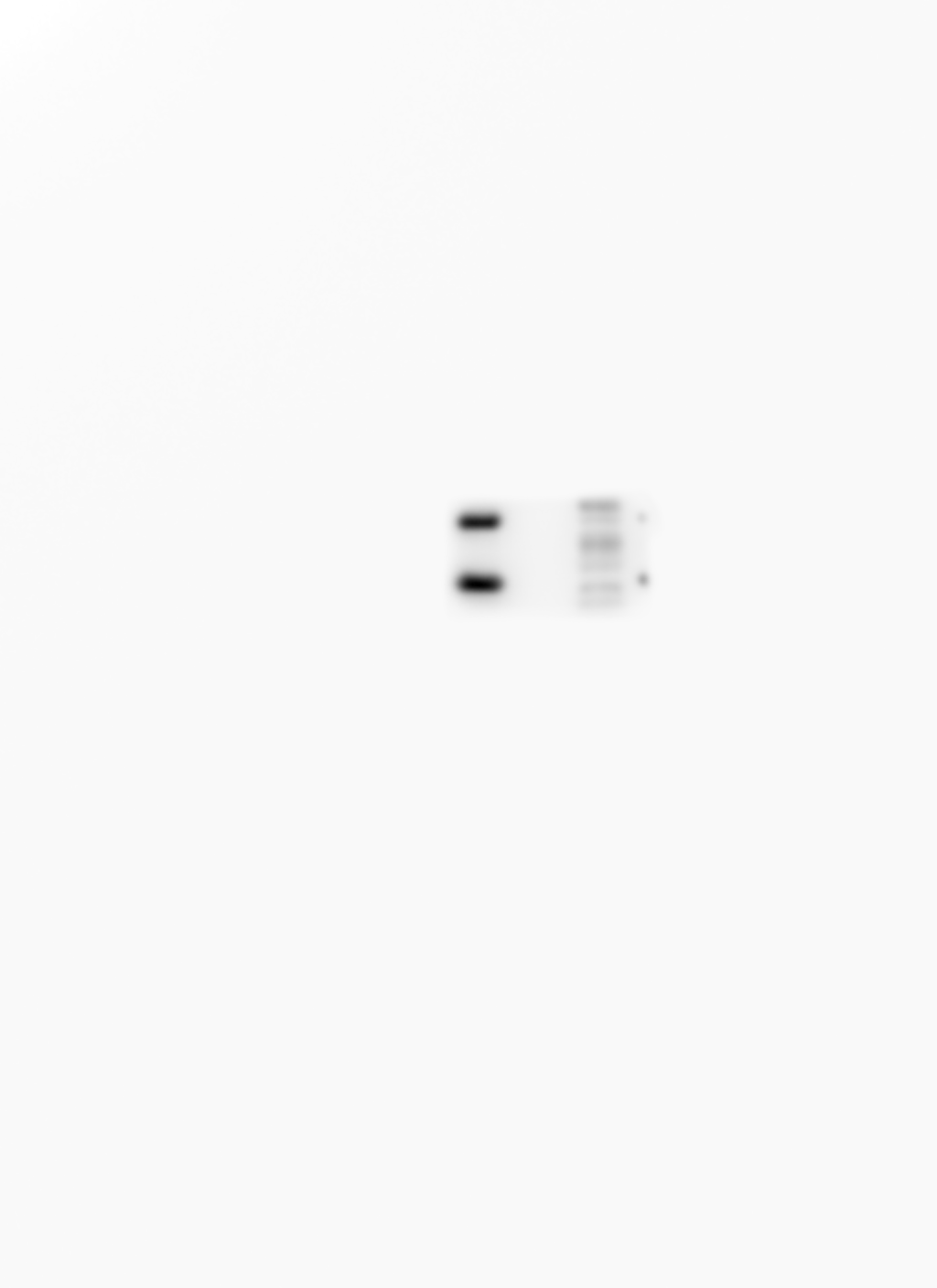

Supplement: Supplementary file 3 — WB Raw data [file 41420_2025_2583_MOESM3_ESM.zip › Figure 6 Panel D/suv39h2 ip 2022.04.03_11.26.14_Ch/suv39h2 ip 2022.04.03_11.26.14_Ch.tif]

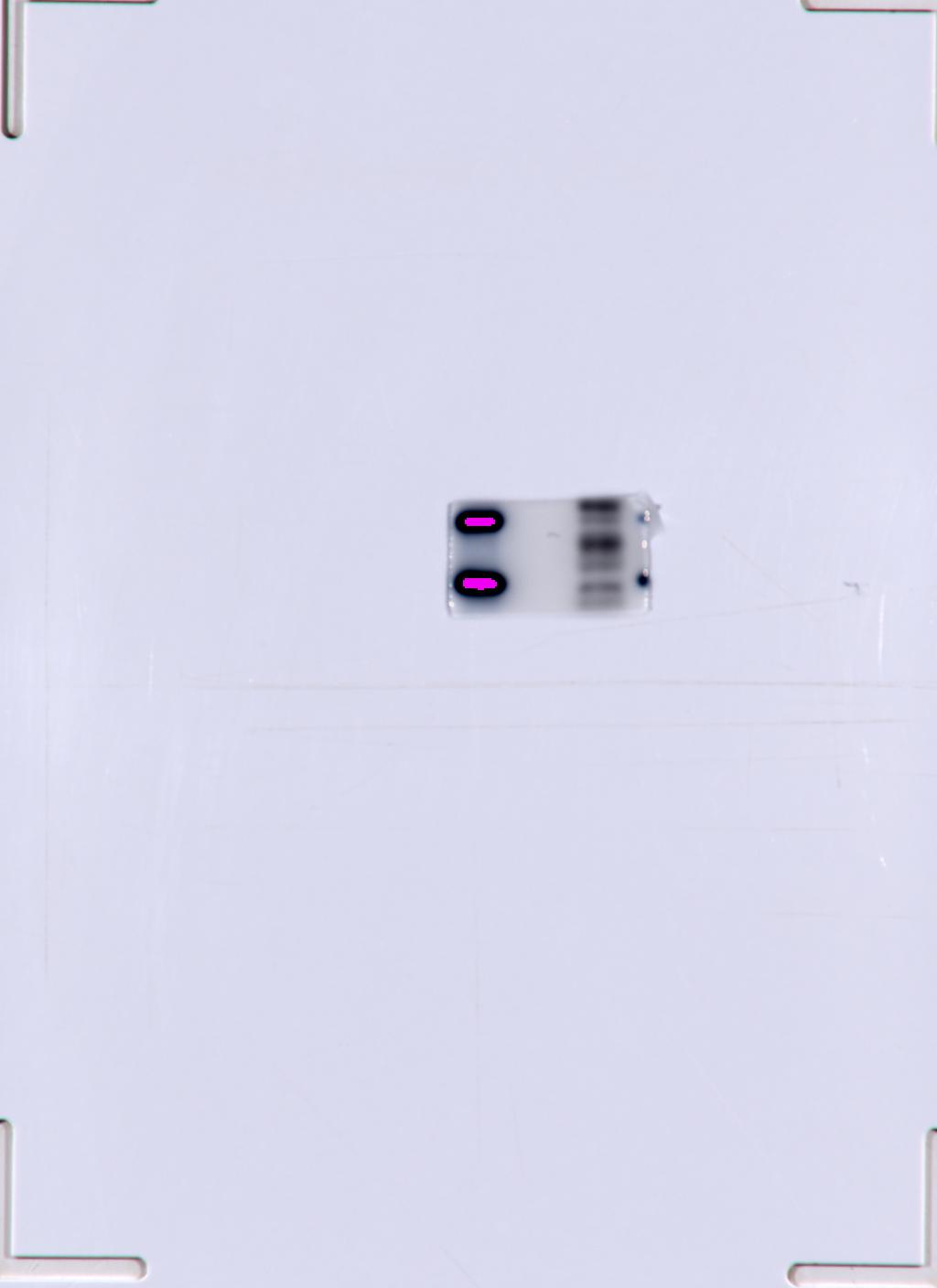

Supplement: Supplementary file 3 — WB Raw data [file 41420_2025_2583_MOESM3_ESM.zip › Figure 6 Panel D/suv39h2 ip 2022.04.03_11.27.20_Ch/suv39h2 ip 2022.04.03_11.27.20_Ch+Marker.jpg]

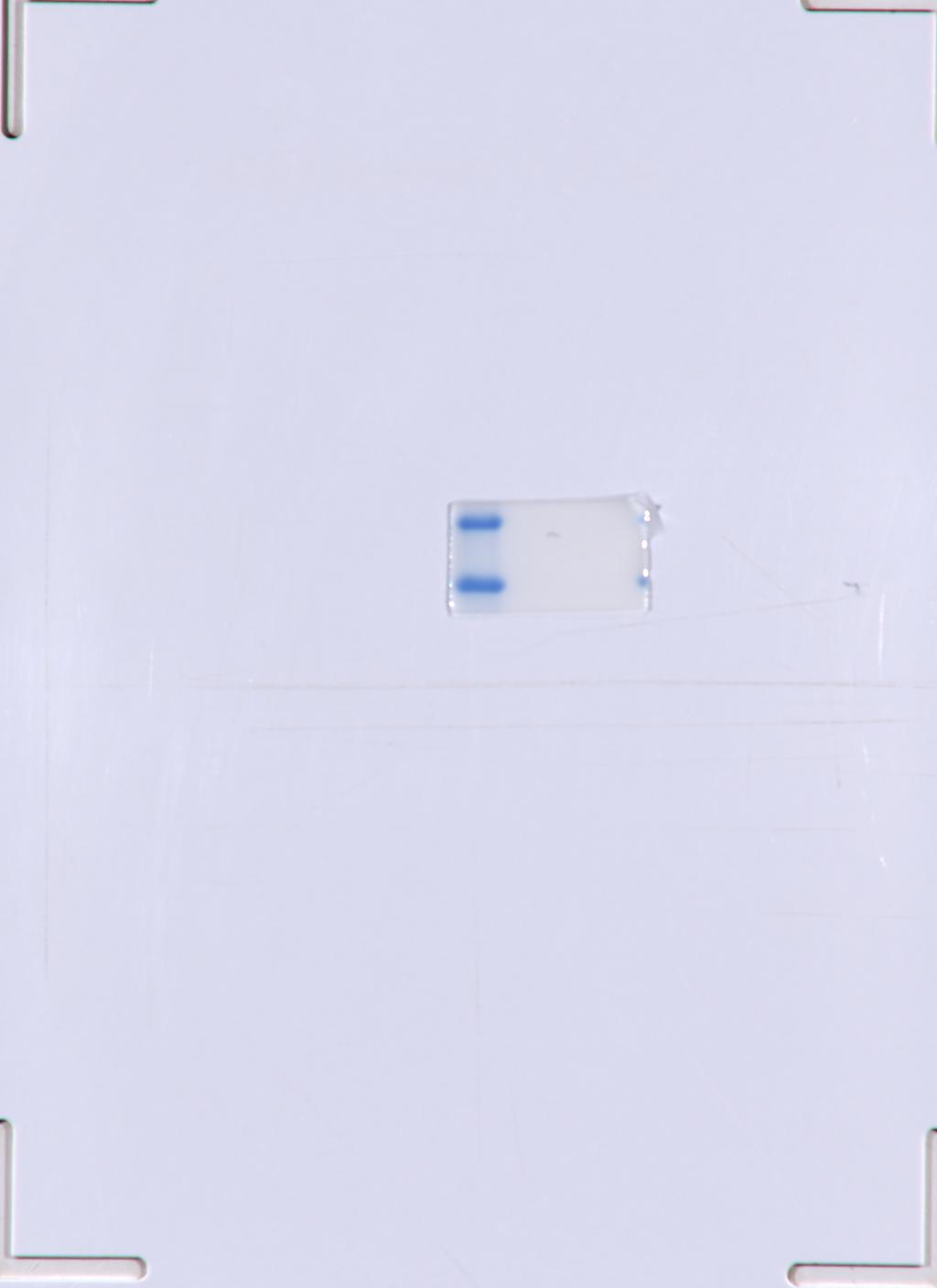

Supplement: Supplementary file 3 — WB Raw data [file 41420_2025_2583_MOESM3_ESM.zip › Figure 6 Panel D/suv39h2 ip 2022.04.03_11.27.20_Ch/suv39h2 ip 2022.04.03_11.27.20_Ch-Marker.jpg]

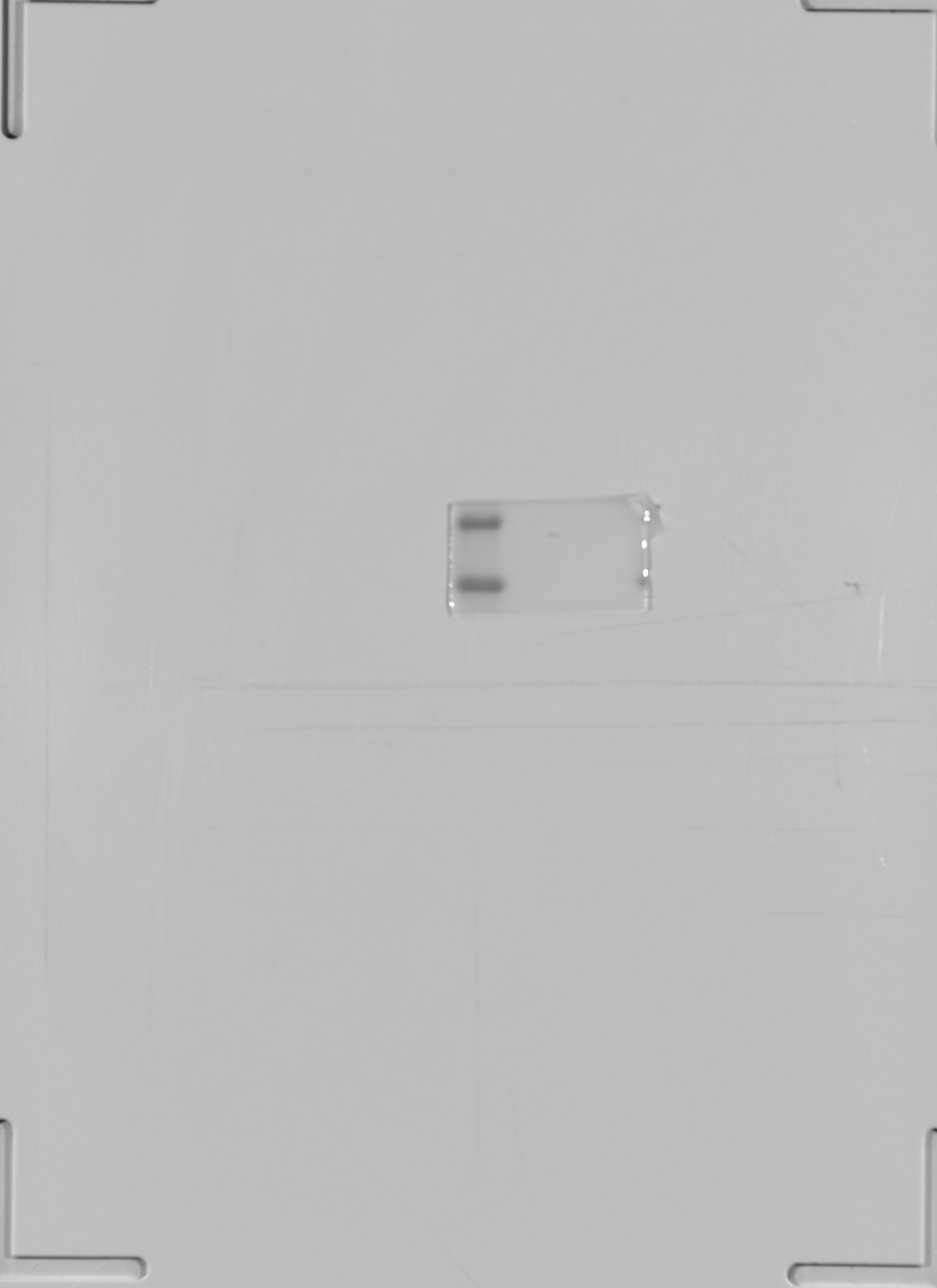

Supplement: Supplementary file 3 — WB Raw data [file 41420_2025_2583_MOESM3_ESM.zip › Figure 6 Panel D/suv39h2 ip 2022.04.03_11.27.20_Ch/suv39h2 ip 2022.04.03_11.27.20_Ch-Marker.tif]

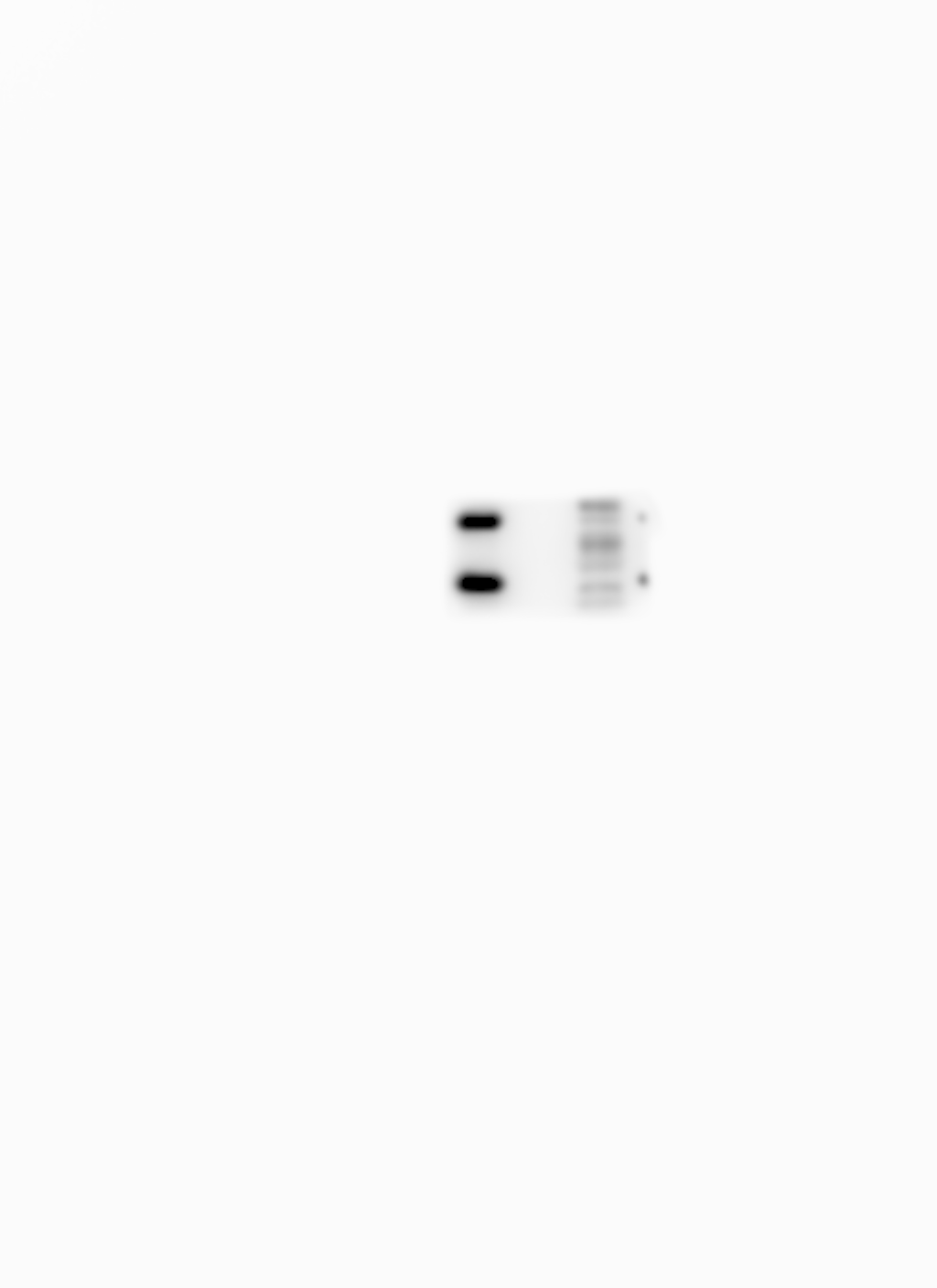

Supplement: Supplementary file 3 — WB Raw data [file 41420_2025_2583_MOESM3_ESM.zip › Figure 6 Panel D/suv39h2 ip 2022.04.03_11.27.20_Ch/suv39h2 ip 2022.04.03_11.27.20_Ch.tif]

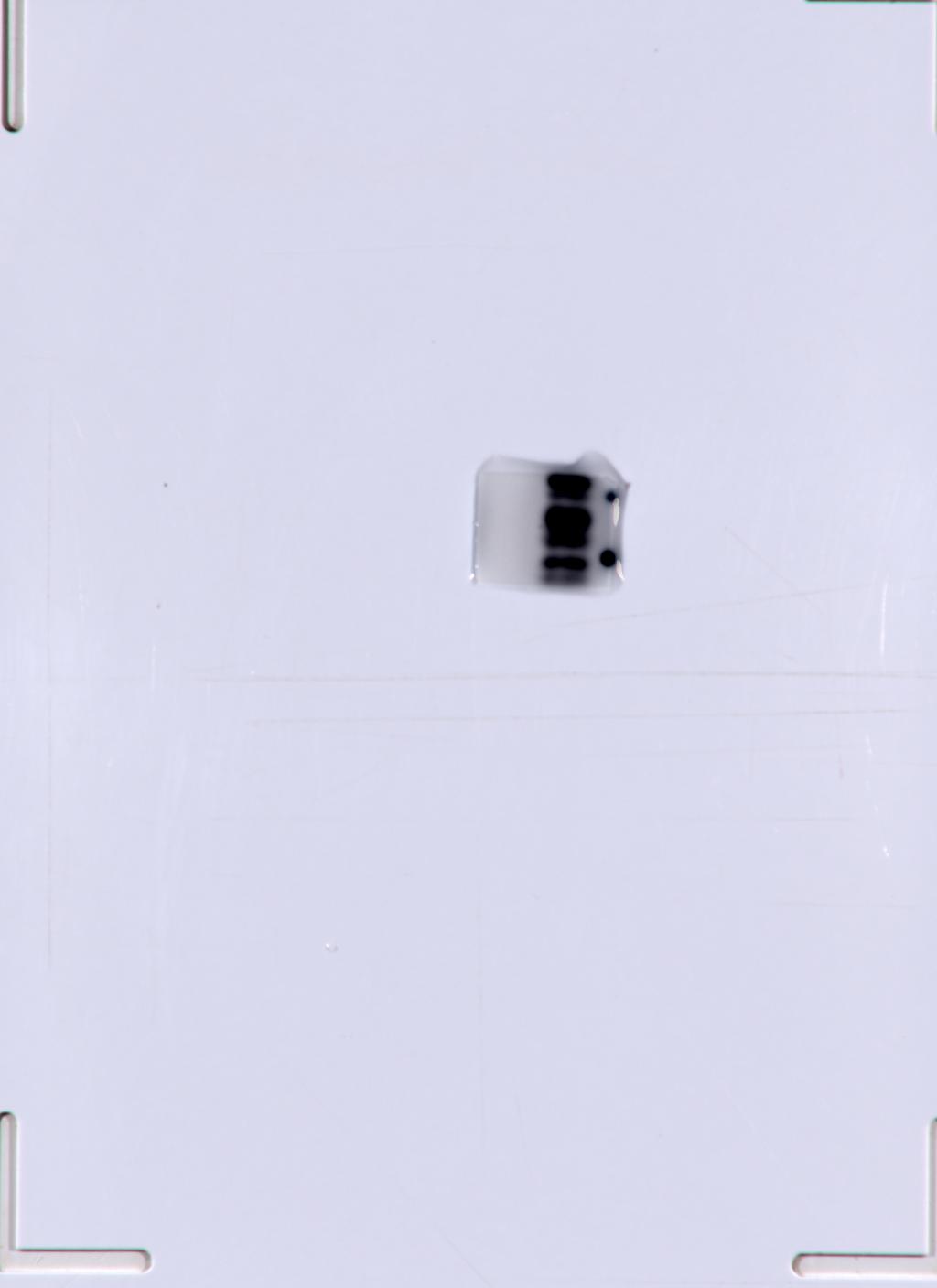

Supplement: Supplementary file 3 — WB Raw data [file 41420_2025_2583_MOESM3_ESM.zip › Figure 6 Panel D/suv39h2 ip 2022.04.03_11.29.23_Ch/suv39h2 ip 2022.04.03_11.29.23_Ch+Marker.jpg]

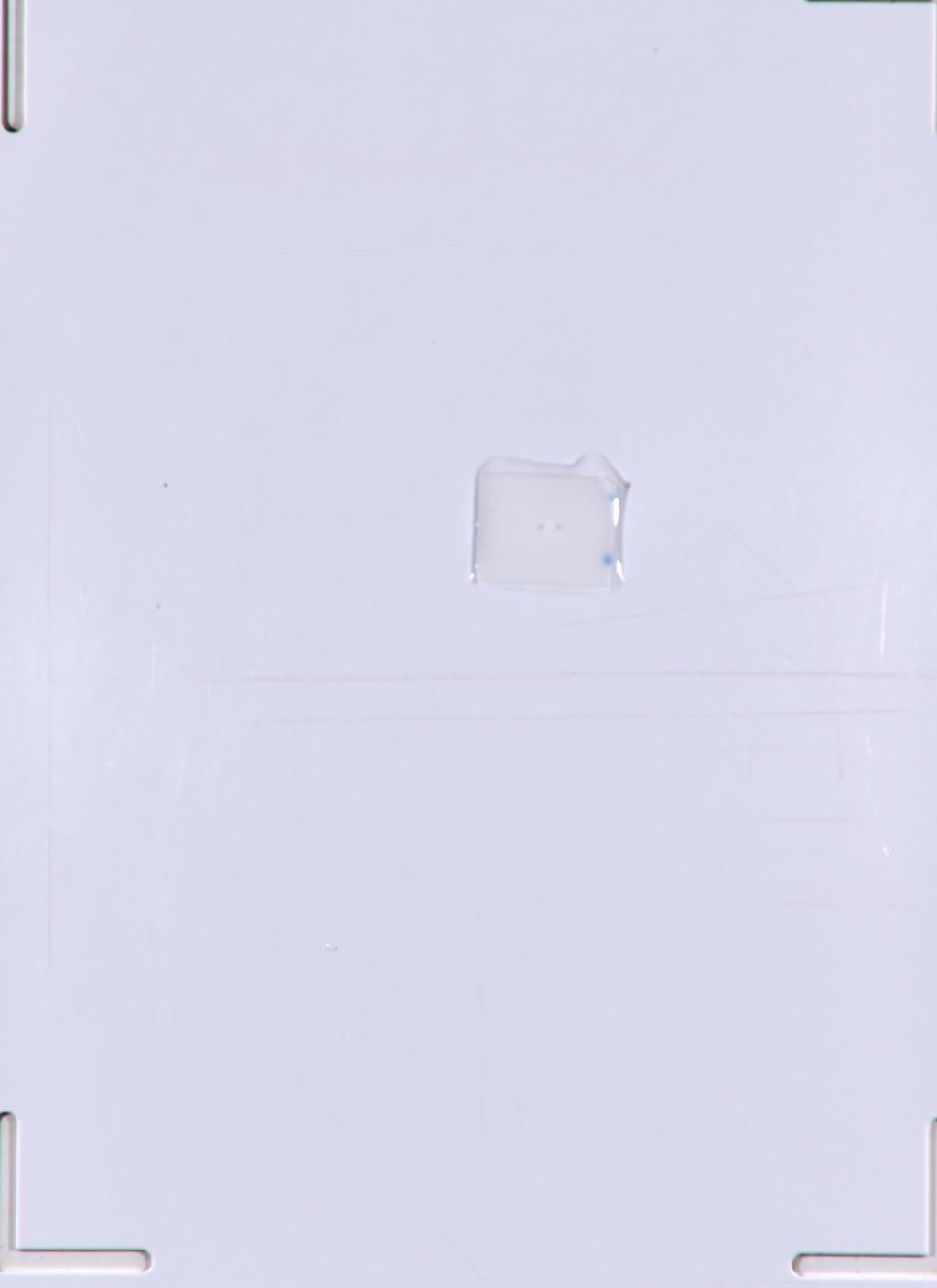

Supplement: Supplementary file 3 — WB Raw data [file 41420_2025_2583_MOESM3_ESM.zip › Figure 6 Panel D/suv39h2 ip 2022.04.03_11.29.23_Ch/suv39h2 ip 2022.04.03_11.29.23_Ch-Marker.jpg]

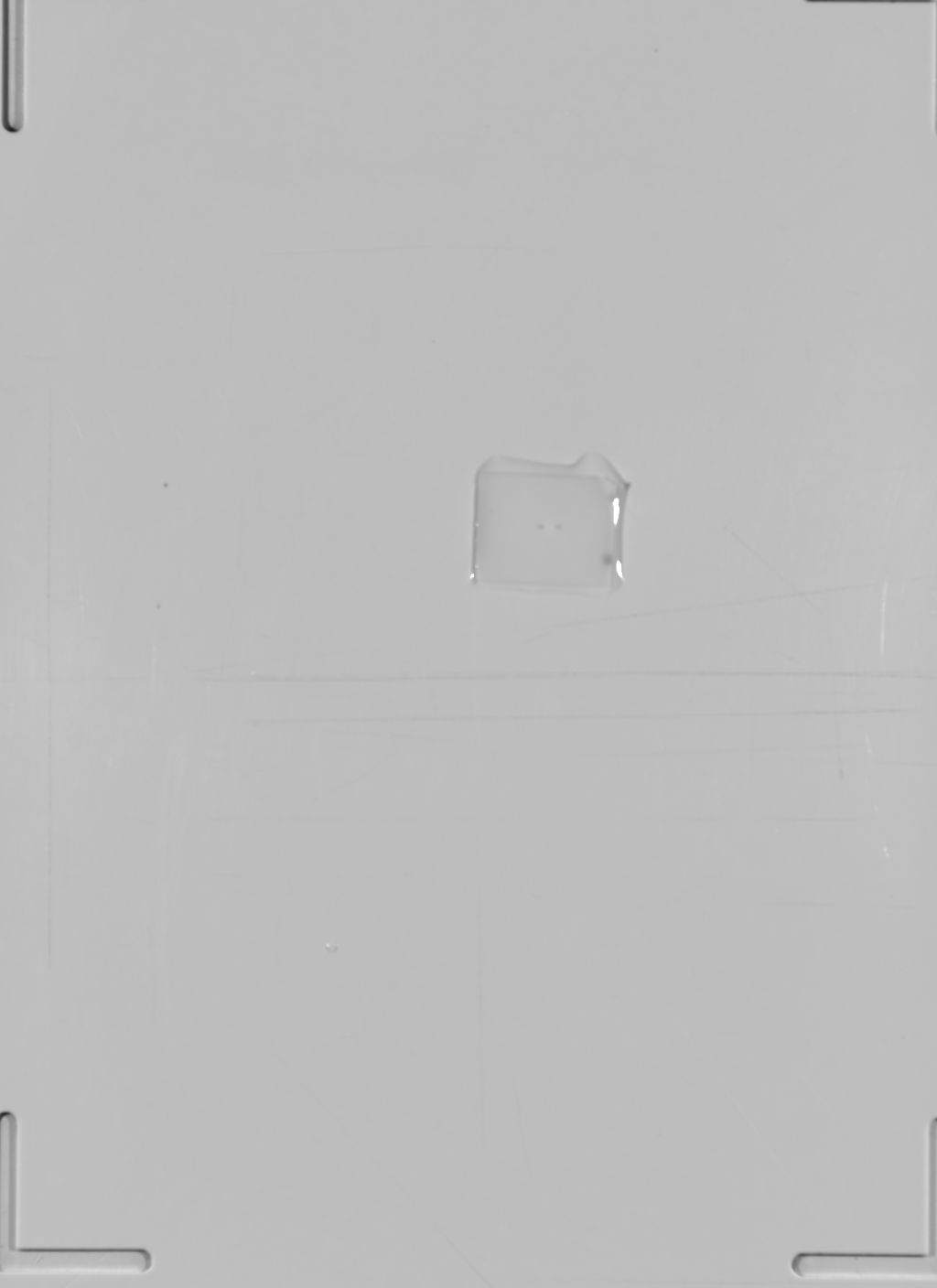

Supplement: Supplementary file 3 — WB Raw data [file 41420_2025_2583_MOESM3_ESM.zip › Figure 6 Panel D/suv39h2 ip 2022.04.03_11.29.23_Ch/suv39h2 ip 2022.04.03_11.29.23_Ch-Marker.tif]

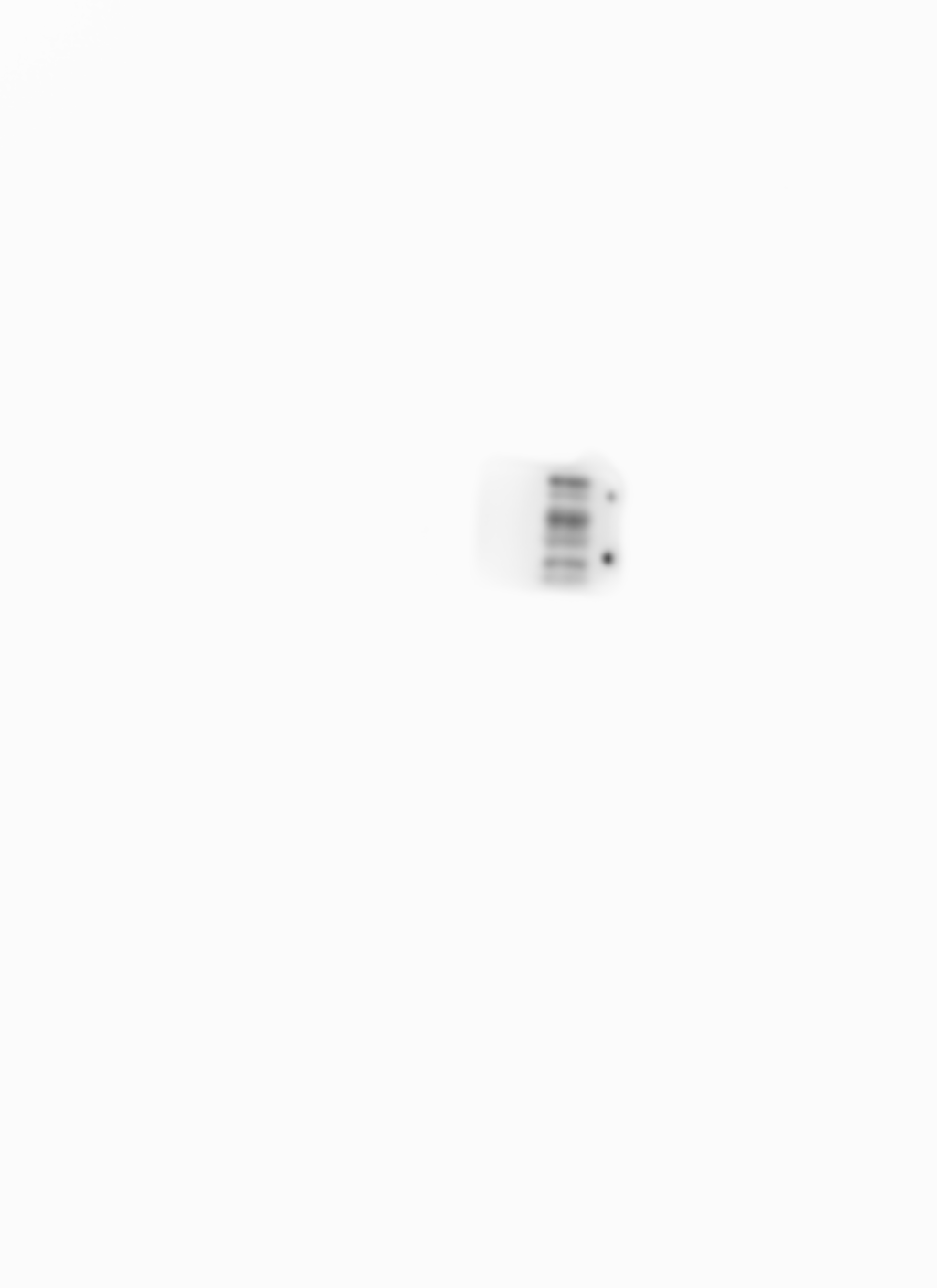

Supplement: Supplementary file 3 — WB Raw data [file 41420_2025_2583_MOESM3_ESM.zip › Figure 6 Panel D/suv39h2 ip 2022.04.03_11.29.23_Ch/suv39h2 ip 2022.04.03_11.29.23_Ch.tif]

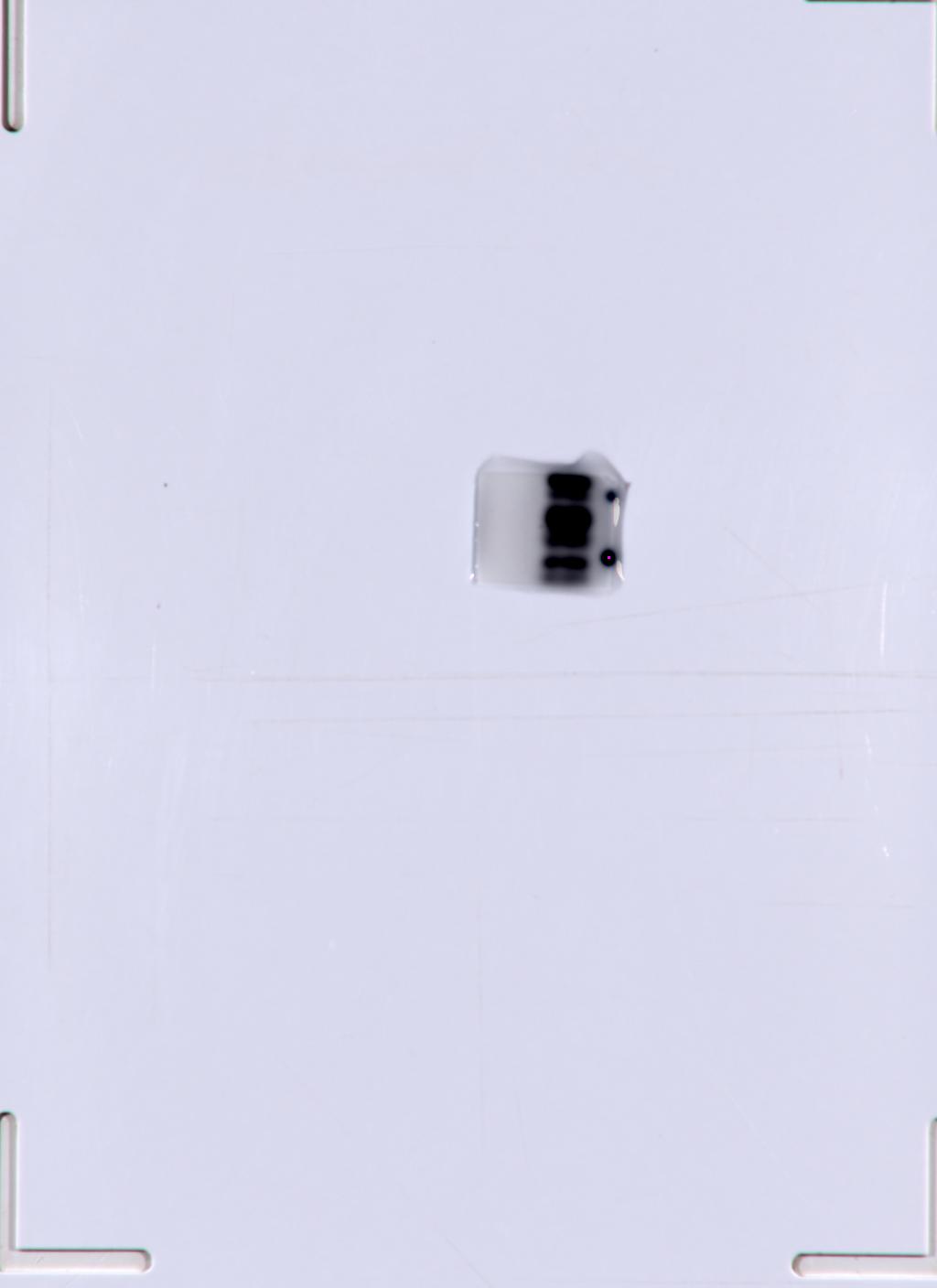

Supplement: Supplementary file 3 — WB Raw data [file 41420_2025_2583_MOESM3_ESM.zip › Figure 6 Panel D/suv39h2 ip 2022.04.03_11.30.57_Ch/suv39h2 ip 2022.04.03_11.30.57_Ch+Marker.jpg]

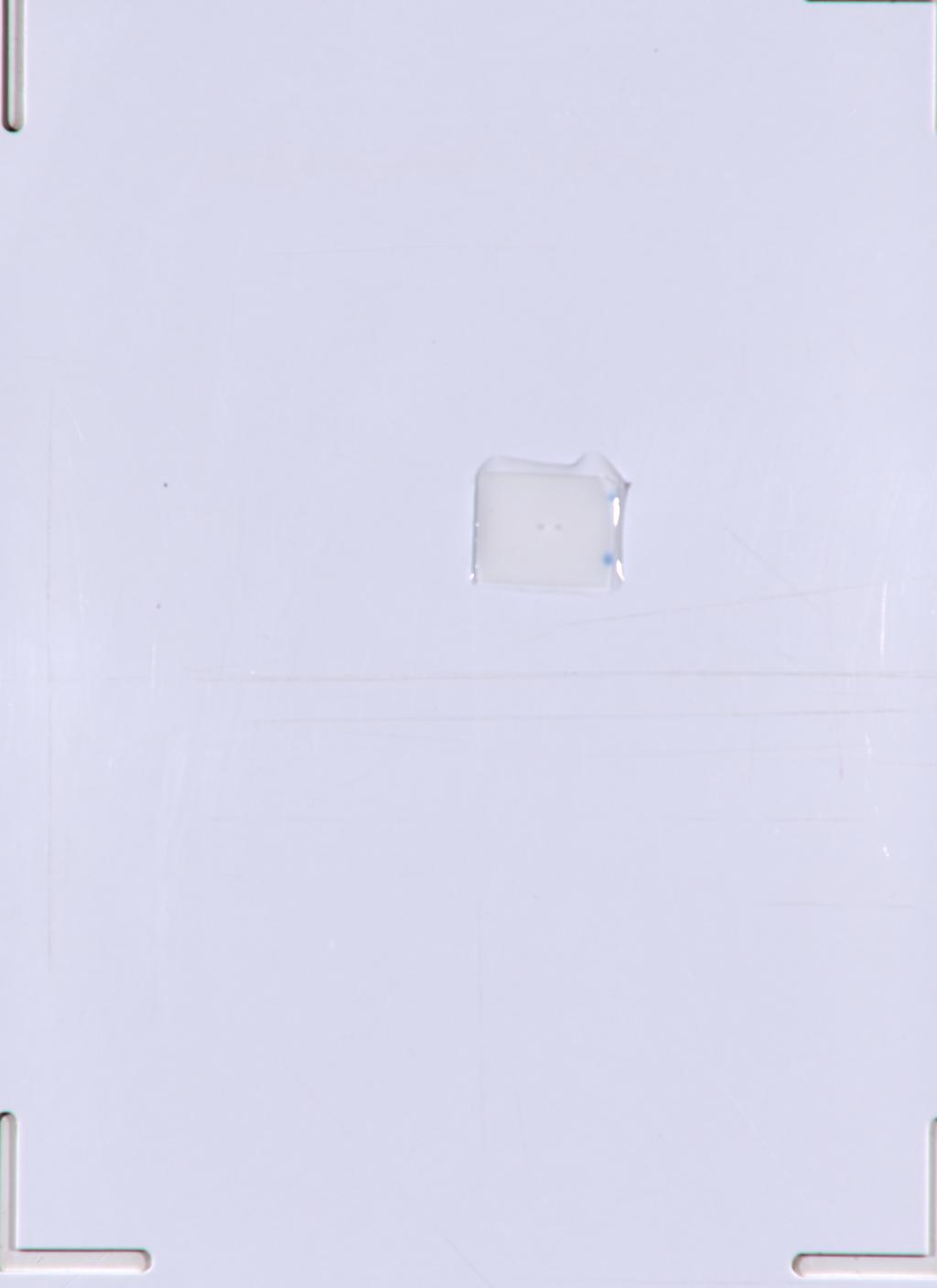

Supplement: Supplementary file 3 — WB Raw data [file 41420_2025_2583_MOESM3_ESM.zip › Figure 6 Panel D/suv39h2 ip 2022.04.03_11.30.57_Ch/suv39h2 ip 2022.04.03_11.30.57_Ch-Marker.jpg]

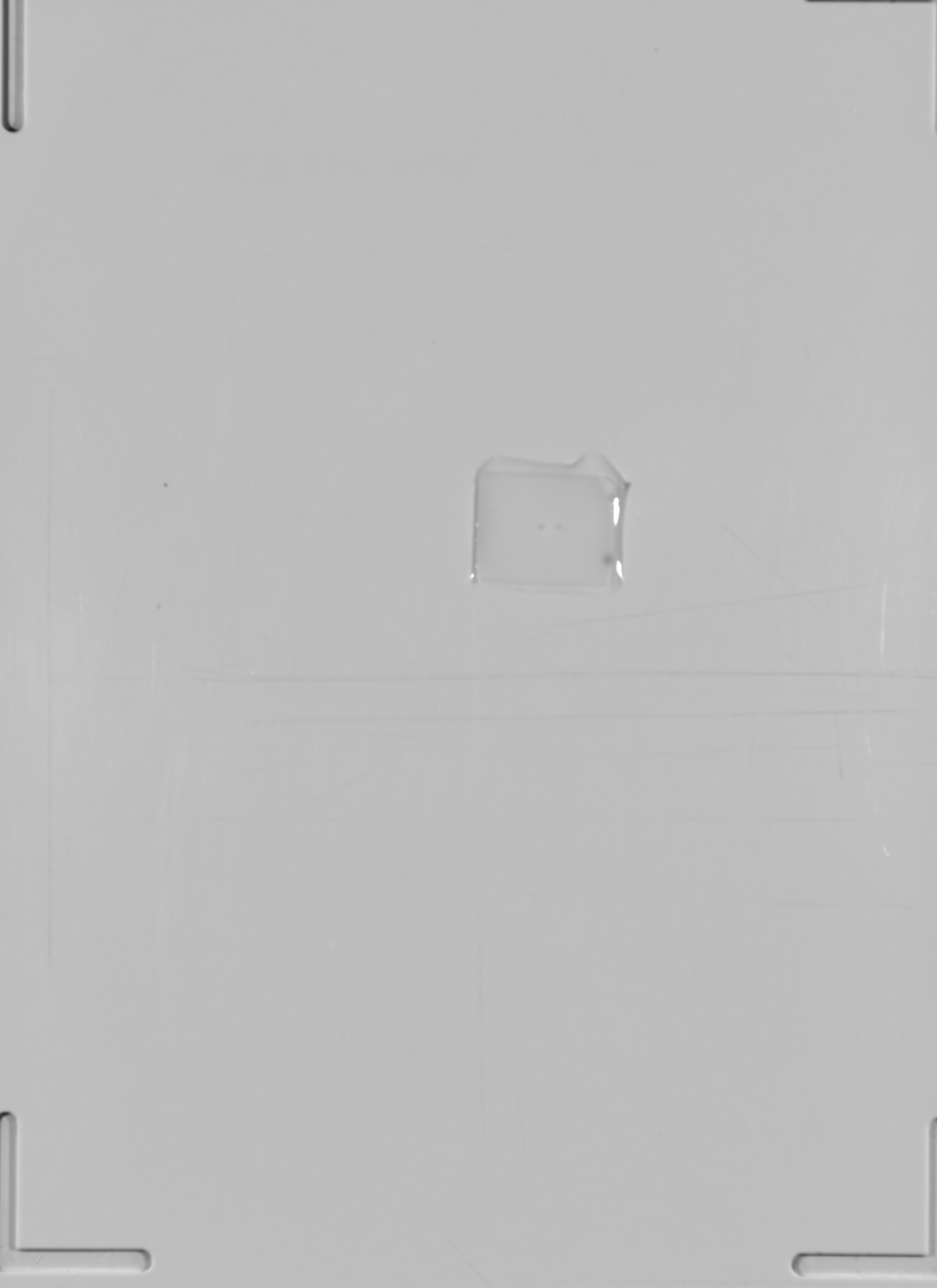

Supplement: Supplementary file 3 — WB Raw data [file 41420_2025_2583_MOESM3_ESM.zip › Figure 6 Panel D/suv39h2 ip 2022.04.03_11.30.57_Ch/suv39h2 ip 2022.04.03_11.30.57_Ch-Marker.tif]

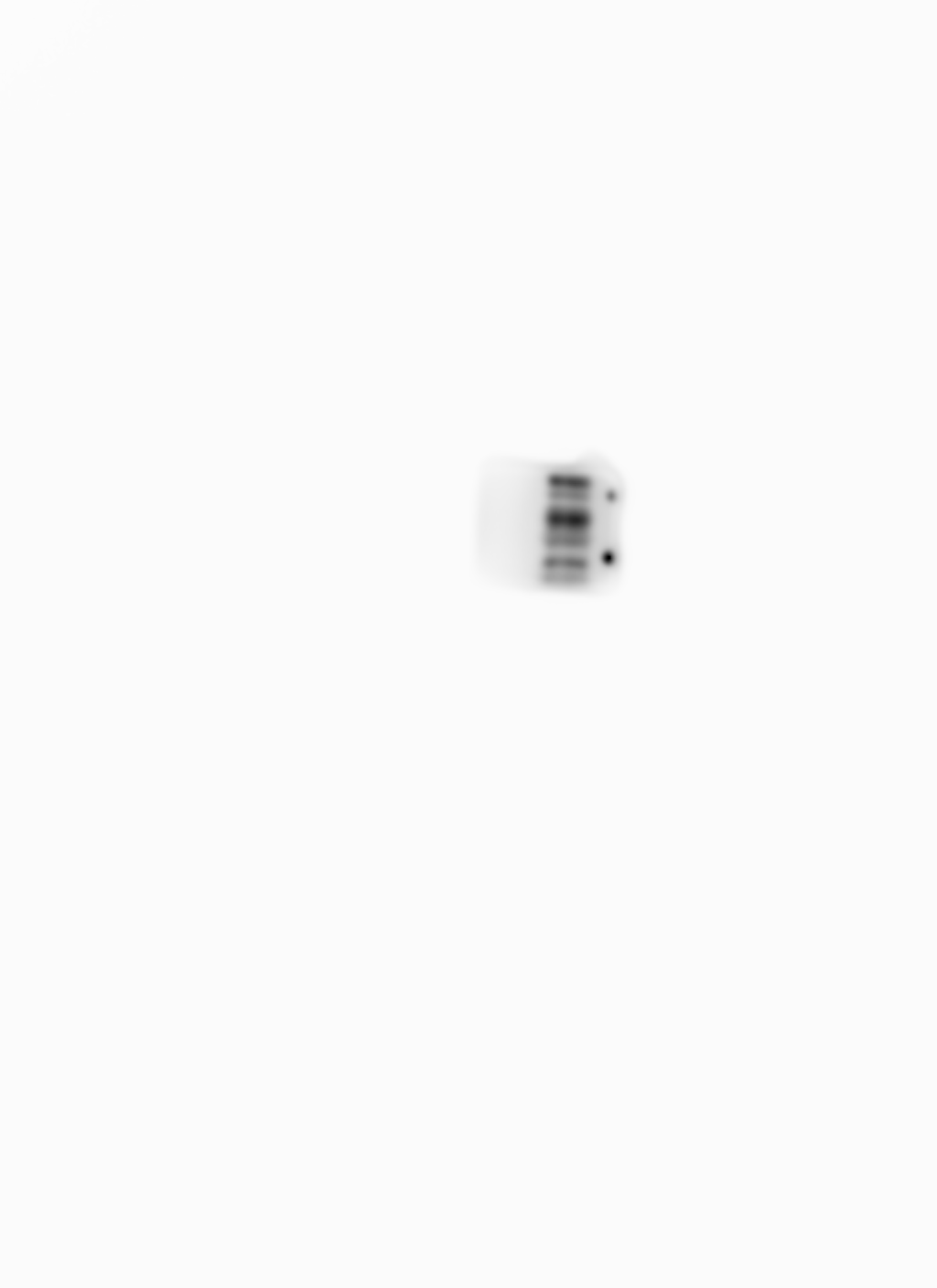

Supplement: Supplementary file 3 — WB Raw data [file 41420_2025_2583_MOESM3_ESM.zip › Figure 6 Panel D/suv39h2 ip 2022.04.03_11.30.57_Ch/suv39h2 ip 2022.04.03_11.30.57_Ch.tif]

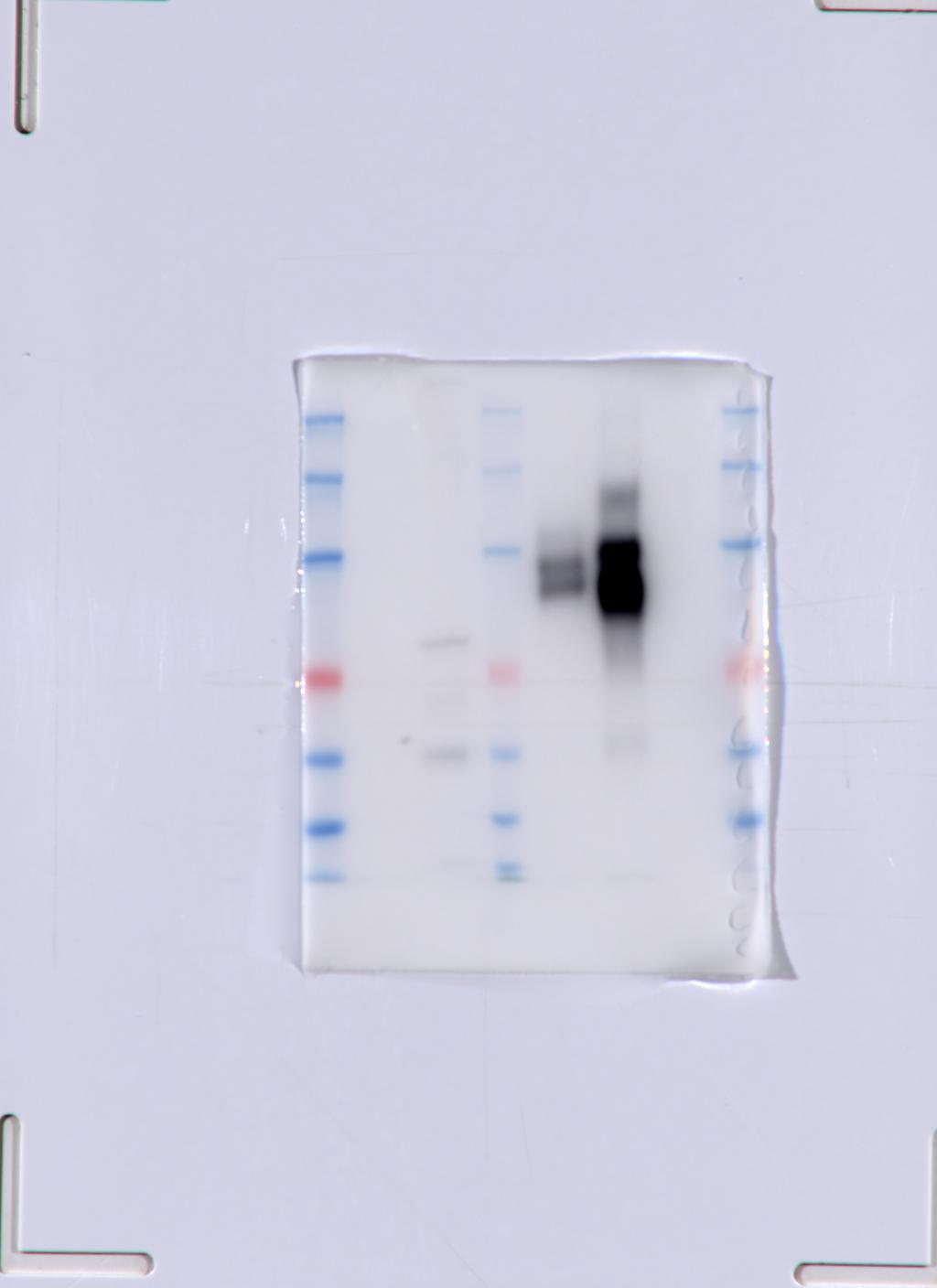

Supplement: Supplementary file 3 — WB Raw data [file 41420_2025_2583_MOESM3_ESM.zip › Figure 6 Panel E/dcaf13 2022.04.09_12.32.28_Ch/dcaf13 2022.04.09_12.32.28_Ch+Marker.jpg]

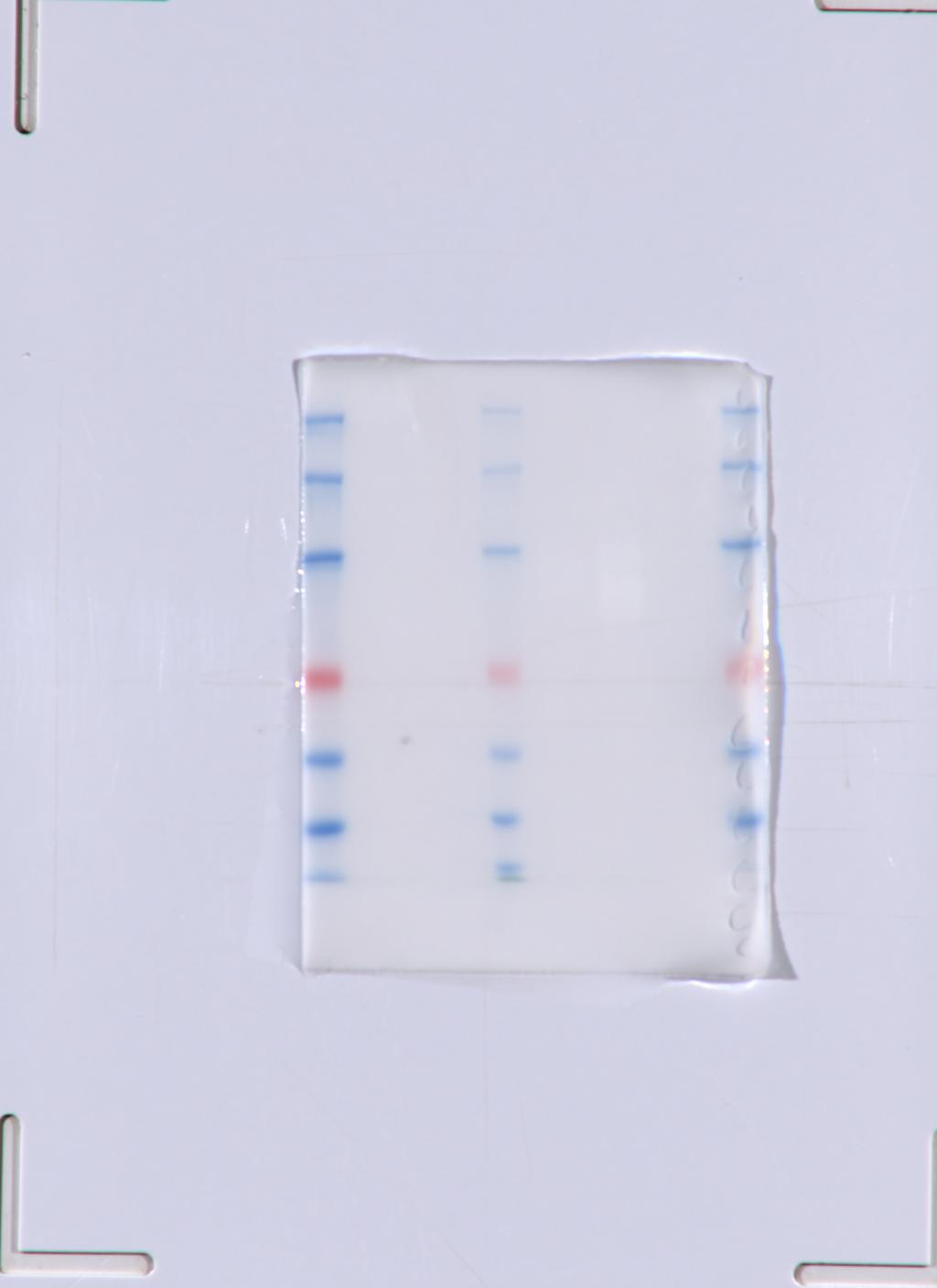

Supplement: Supplementary file 3 — WB Raw data [file 41420_2025_2583_MOESM3_ESM.zip › Figure 6 Panel E/dcaf13 2022.04.09_12.32.28_Ch/dcaf13 2022.04.09_12.32.28_Ch-Marker.jpg]

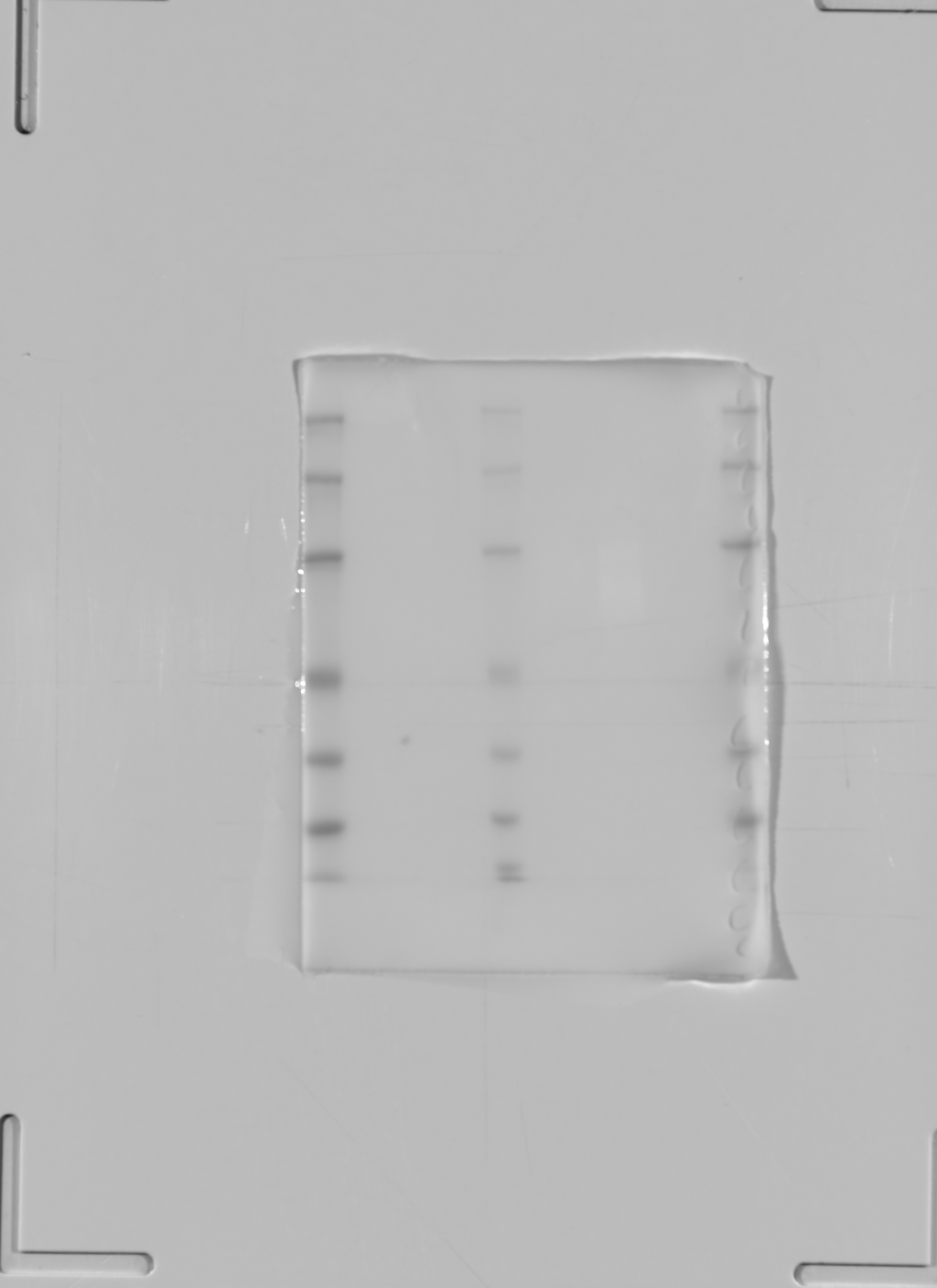

Supplement: Supplementary file 3 — WB Raw data [file 41420_2025_2583_MOESM3_ESM.zip › Figure 6 Panel E/dcaf13 2022.04.09_12.32.28_Ch/dcaf13 2022.04.09_12.32.28_Ch-Marker.tif]

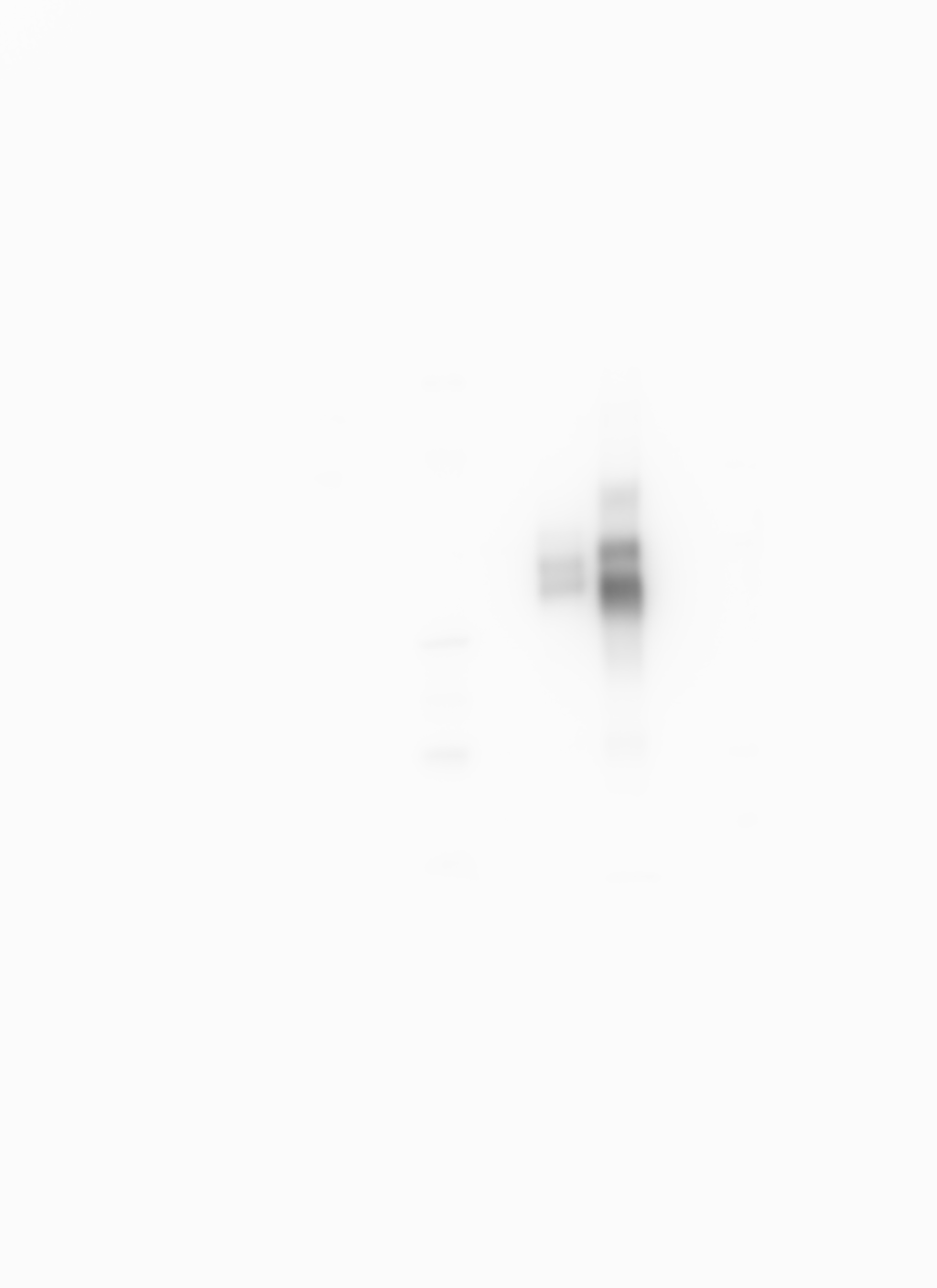

Supplement: Supplementary file 3 — WB Raw data [file 41420_2025_2583_MOESM3_ESM.zip › Figure 6 Panel E/dcaf13 2022.04.09_12.32.28_Ch/dcaf13 2022.04.09_12.32.28_Ch.tif]

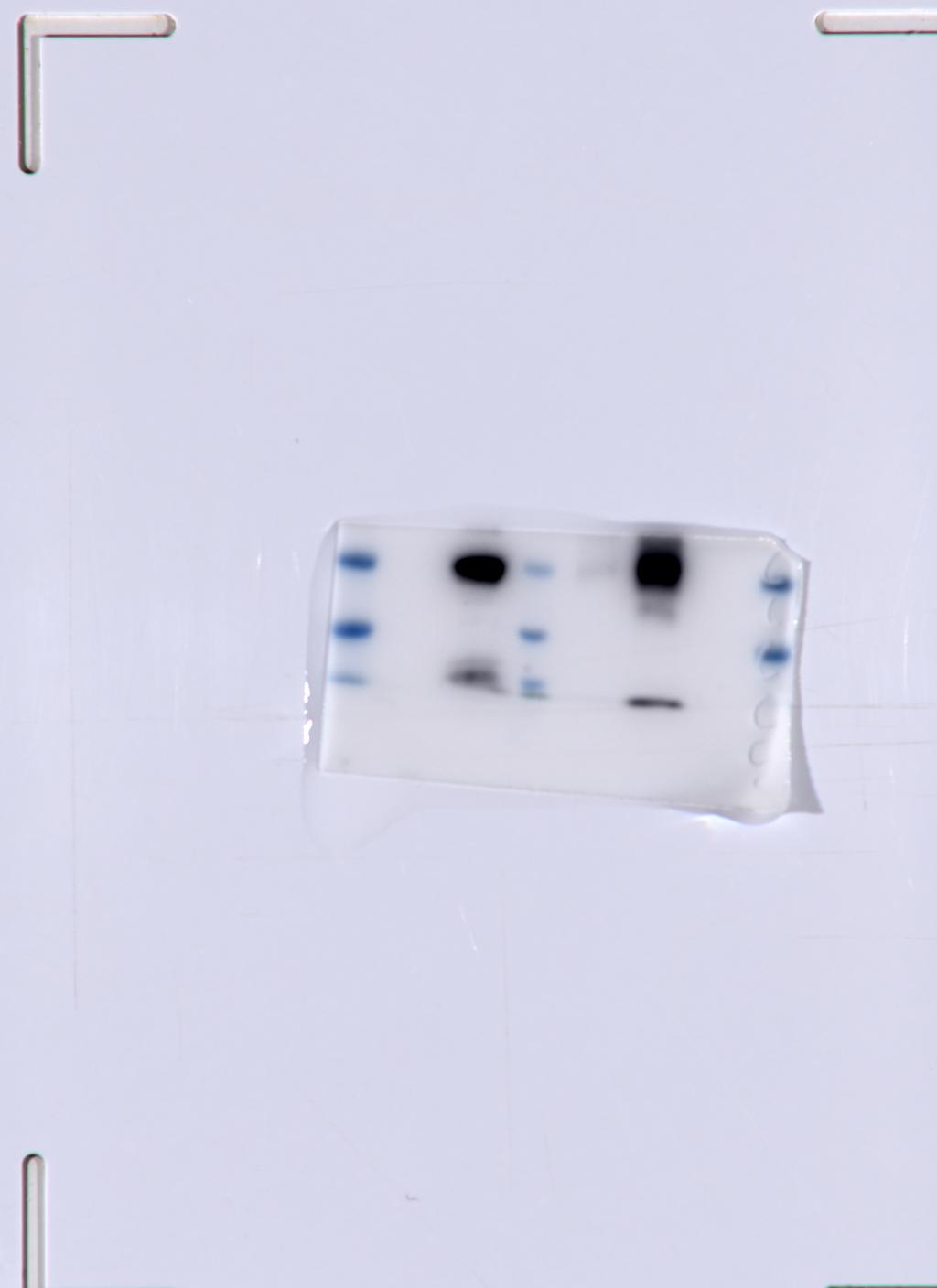

Supplement: Supplementary file 3 — WB Raw data [file 41420_2025_2583_MOESM3_ESM.zip › Figure 6 Panel E/dcaf13 2022.04.09_12.34.44_Ch/dcaf13 2022.04.09_12.34.44_Ch+Marker.jpg]

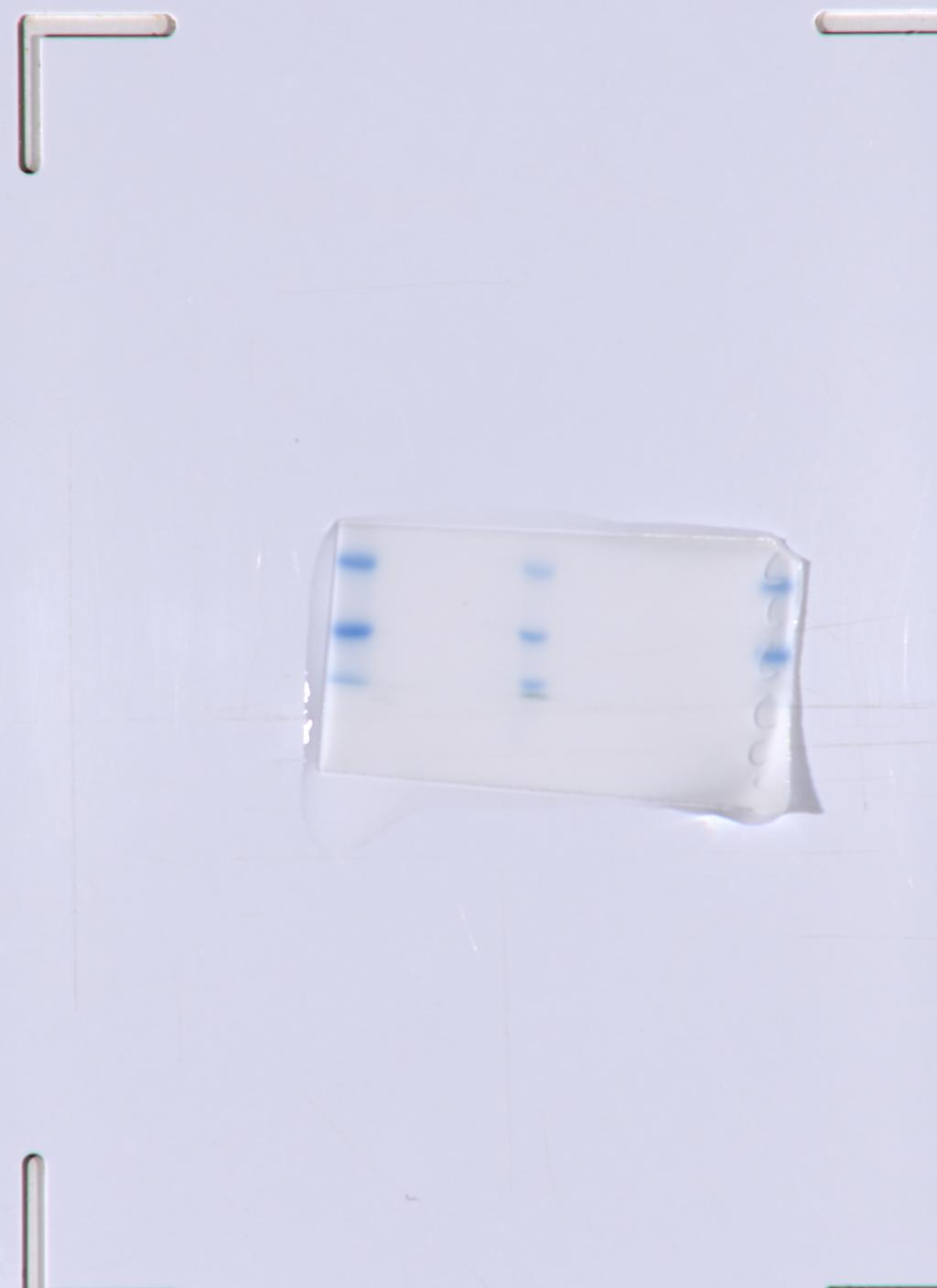

Supplement: Supplementary file 3 — WB Raw data [file 41420_2025_2583_MOESM3_ESM.zip › Figure 6 Panel E/dcaf13 2022.04.09_12.34.44_Ch/dcaf13 2022.04.09_12.34.44_Ch-Marker.jpg]

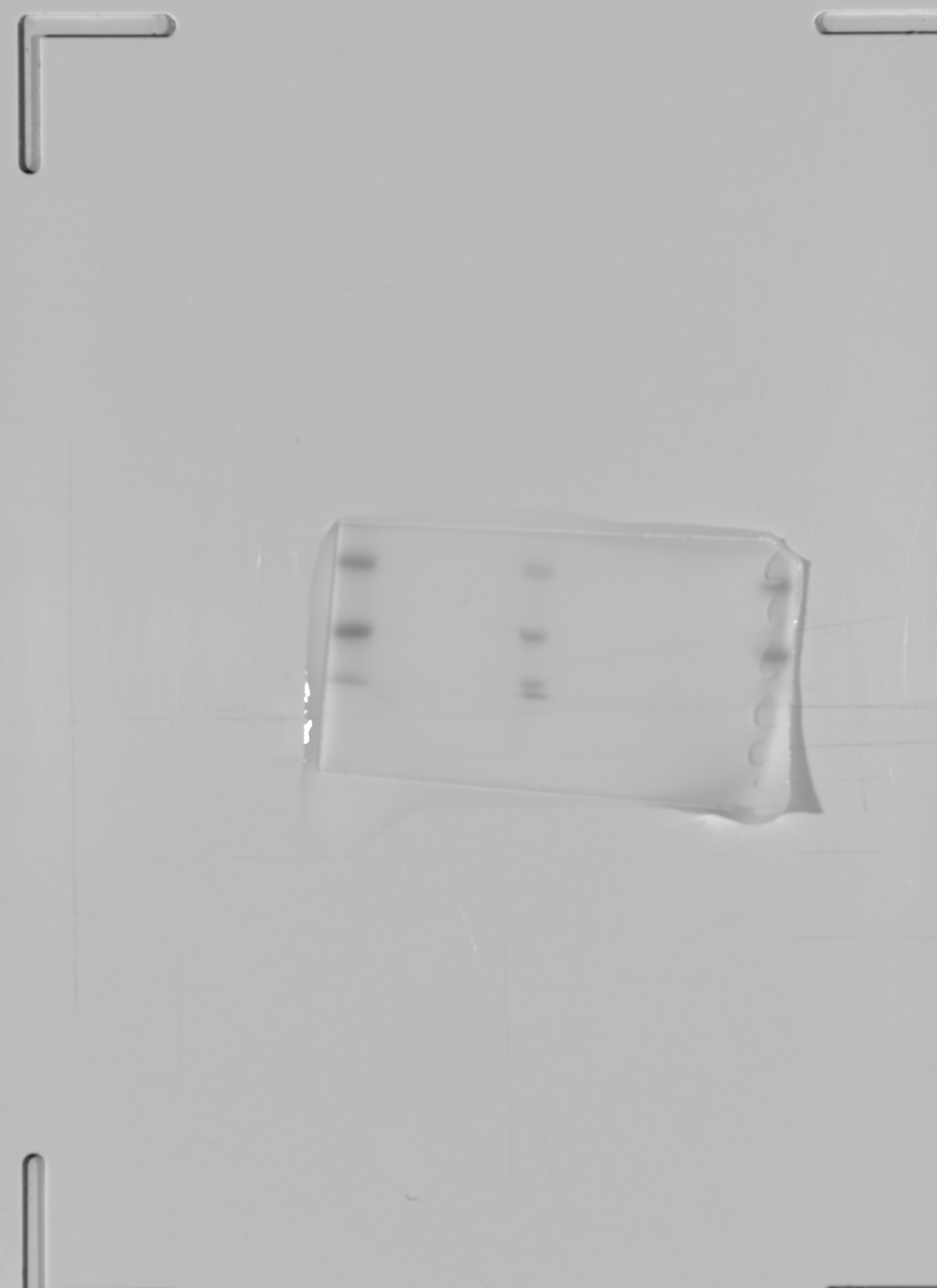

Supplement: Supplementary file 3 — WB Raw data [file 41420_2025_2583_MOESM3_ESM.zip › Figure 6 Panel E/dcaf13 2022.04.09_12.34.44_Ch/dcaf13 2022.04.09_12.34.44_Ch-Marker.tif]

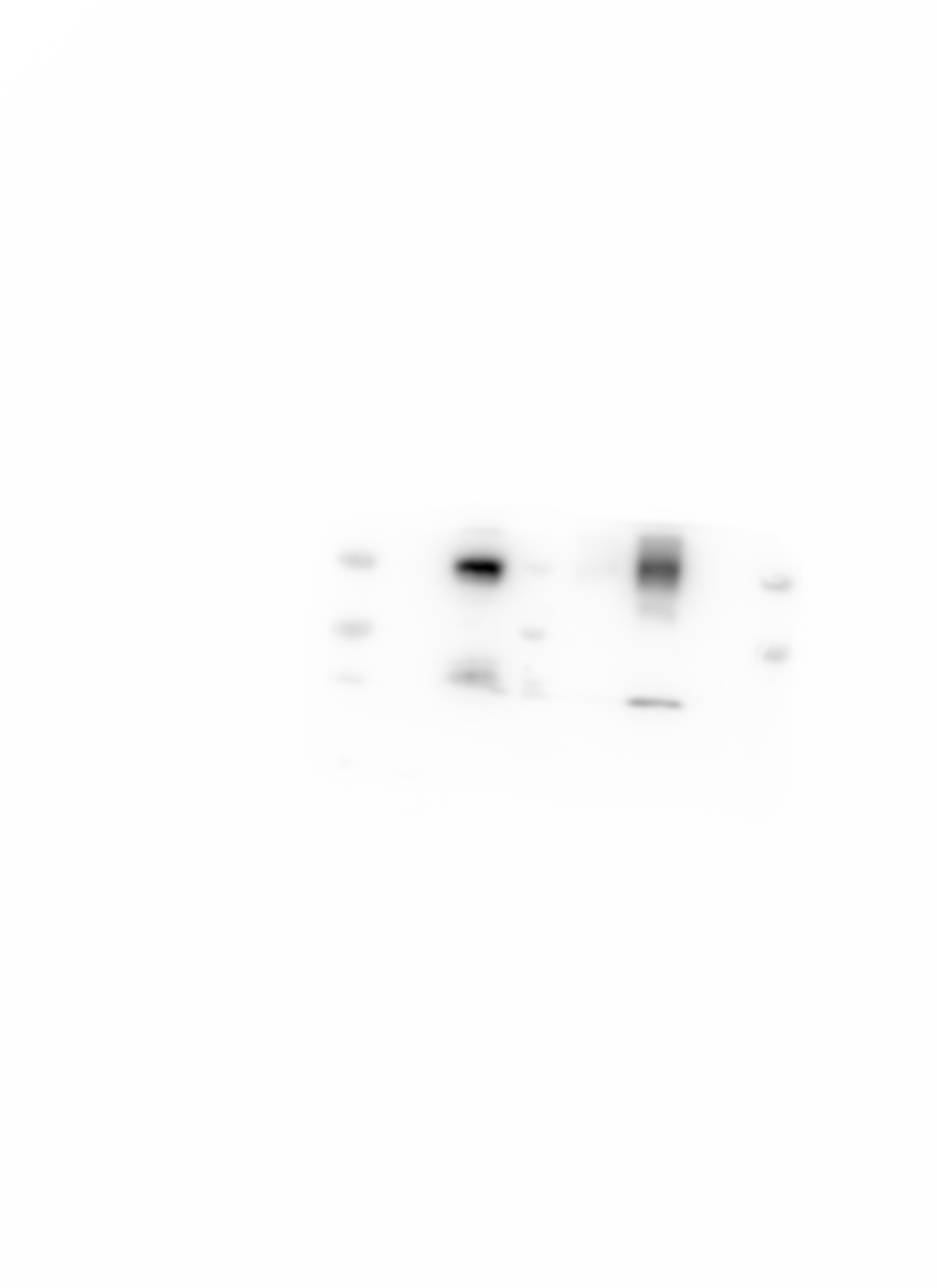

Supplement: Supplementary file 3 — WB Raw data [file 41420_2025_2583_MOESM3_ESM.zip › Figure 6 Panel E/dcaf13 2022.04.09_12.34.44_Ch/dcaf13 2022.04.09_12.34.44_Ch.tif]

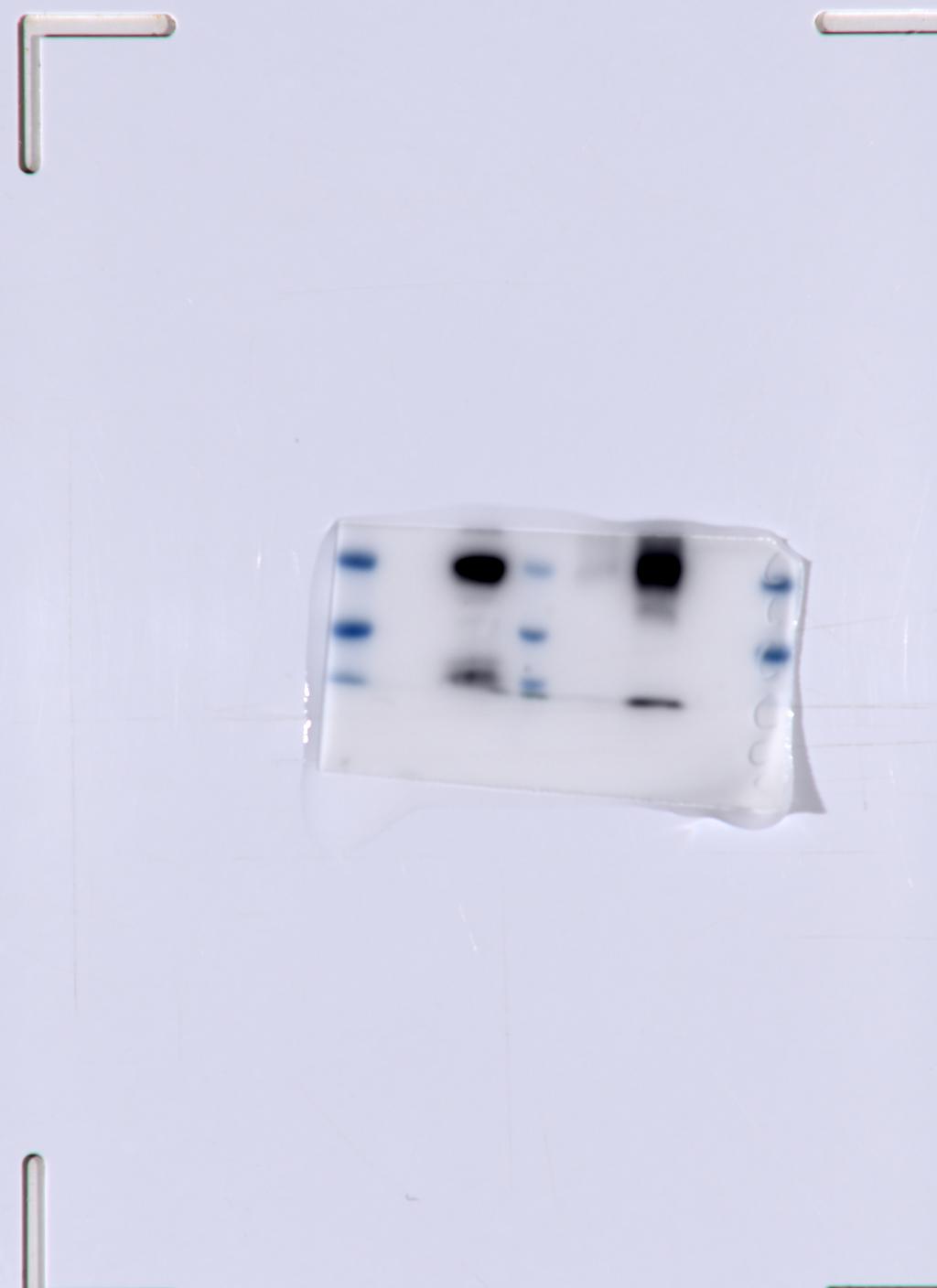

Supplement: Supplementary file 3 — WB Raw data [file 41420_2025_2583_MOESM3_ESM.zip › Figure 6 Panel E/dcaf13 2022.04.09_12.35.56_Ch/dcaf13 2022.04.09_12.35.56_Ch+Marker.jpg]

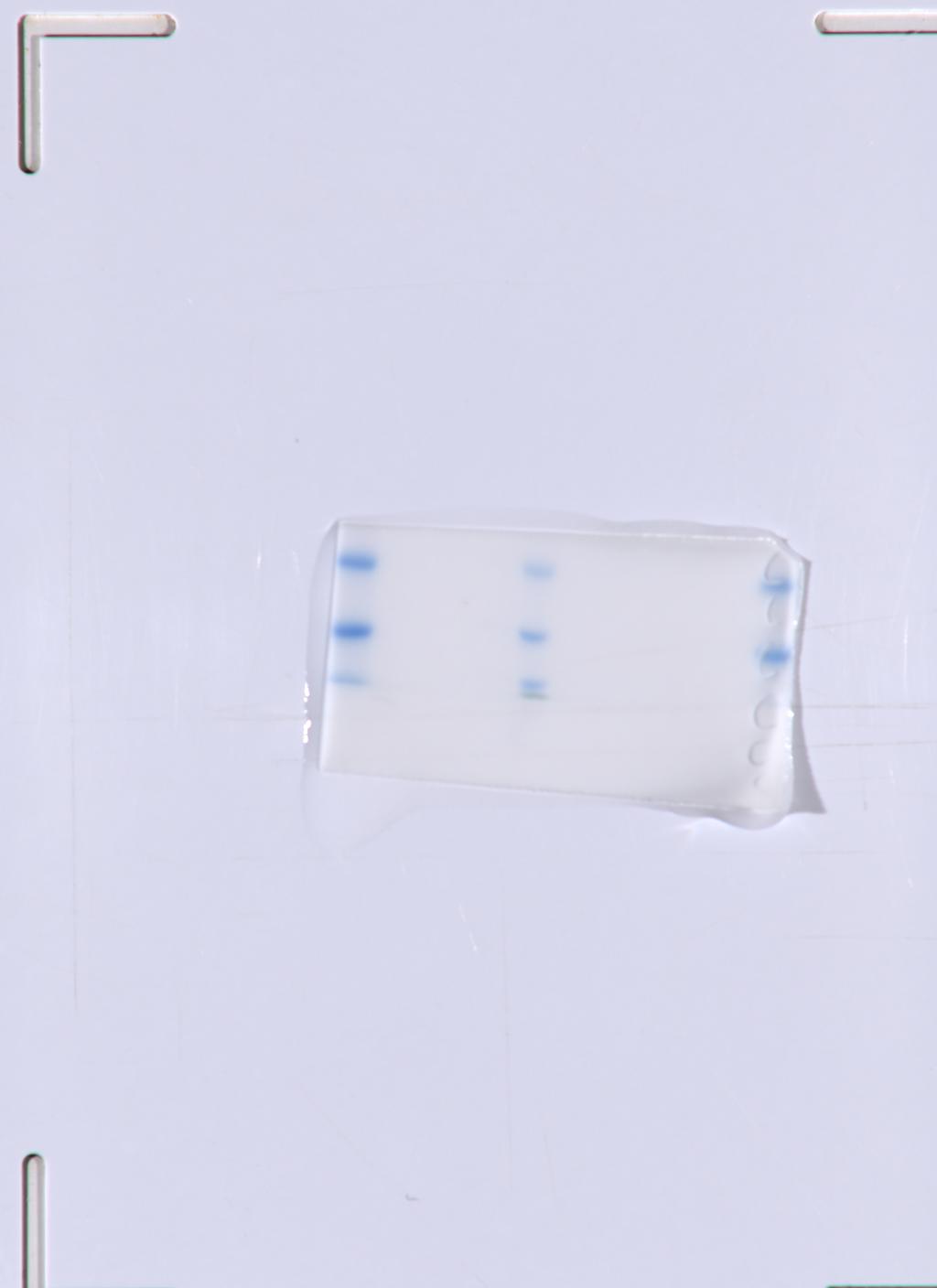

Supplement: Supplementary file 3 — WB Raw data [file 41420_2025_2583_MOESM3_ESM.zip › Figure 6 Panel E/dcaf13 2022.04.09_12.35.56_Ch/dcaf13 2022.04.09_12.35.56_Ch-Marker.jpg]

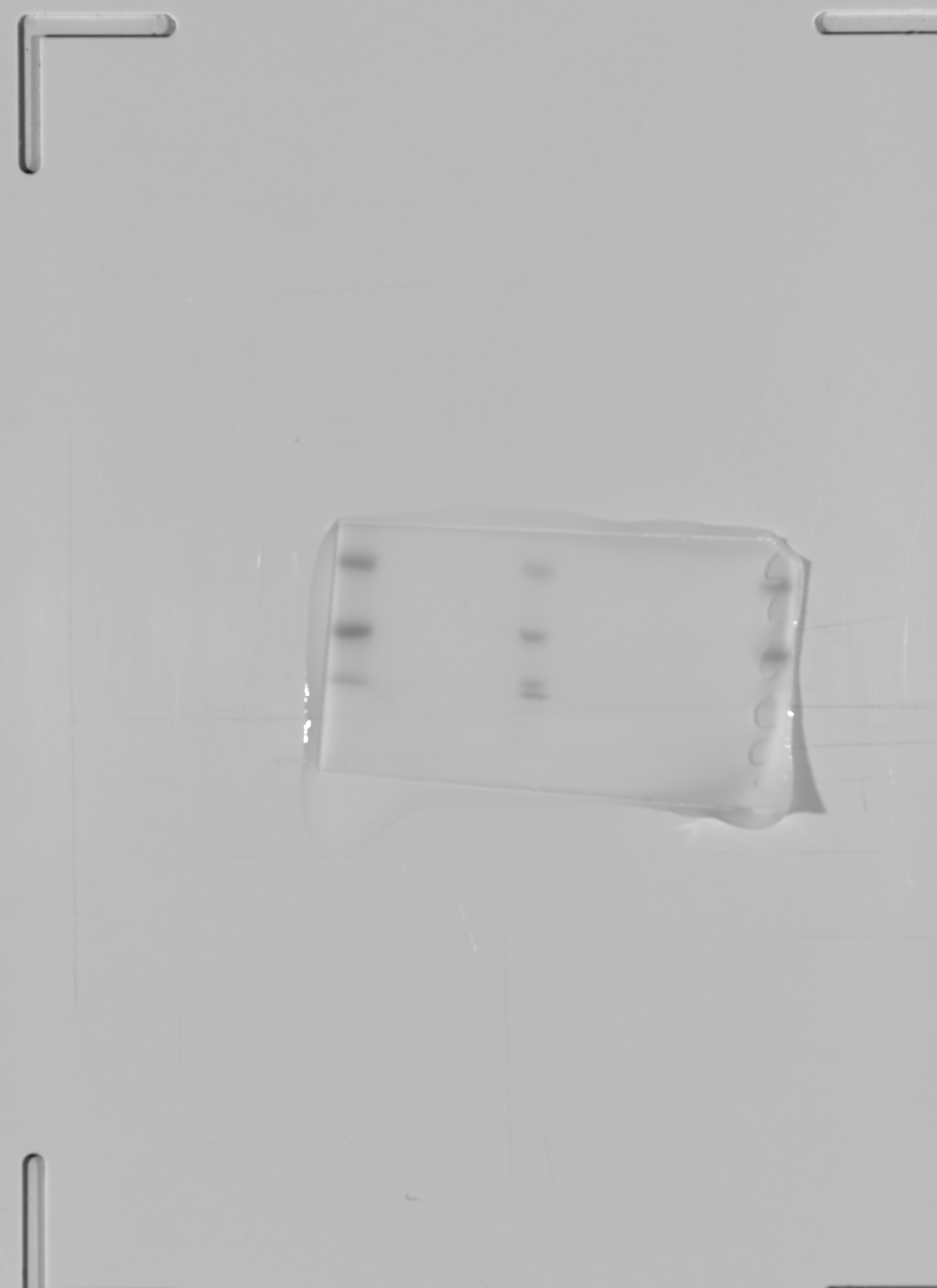

Supplement: Supplementary file 3 — WB Raw data [file 41420_2025_2583_MOESM3_ESM.zip › Figure 6 Panel E/dcaf13 2022.04.09_12.35.56_Ch/dcaf13 2022.04.09_12.35.56_Ch-Marker.tif]

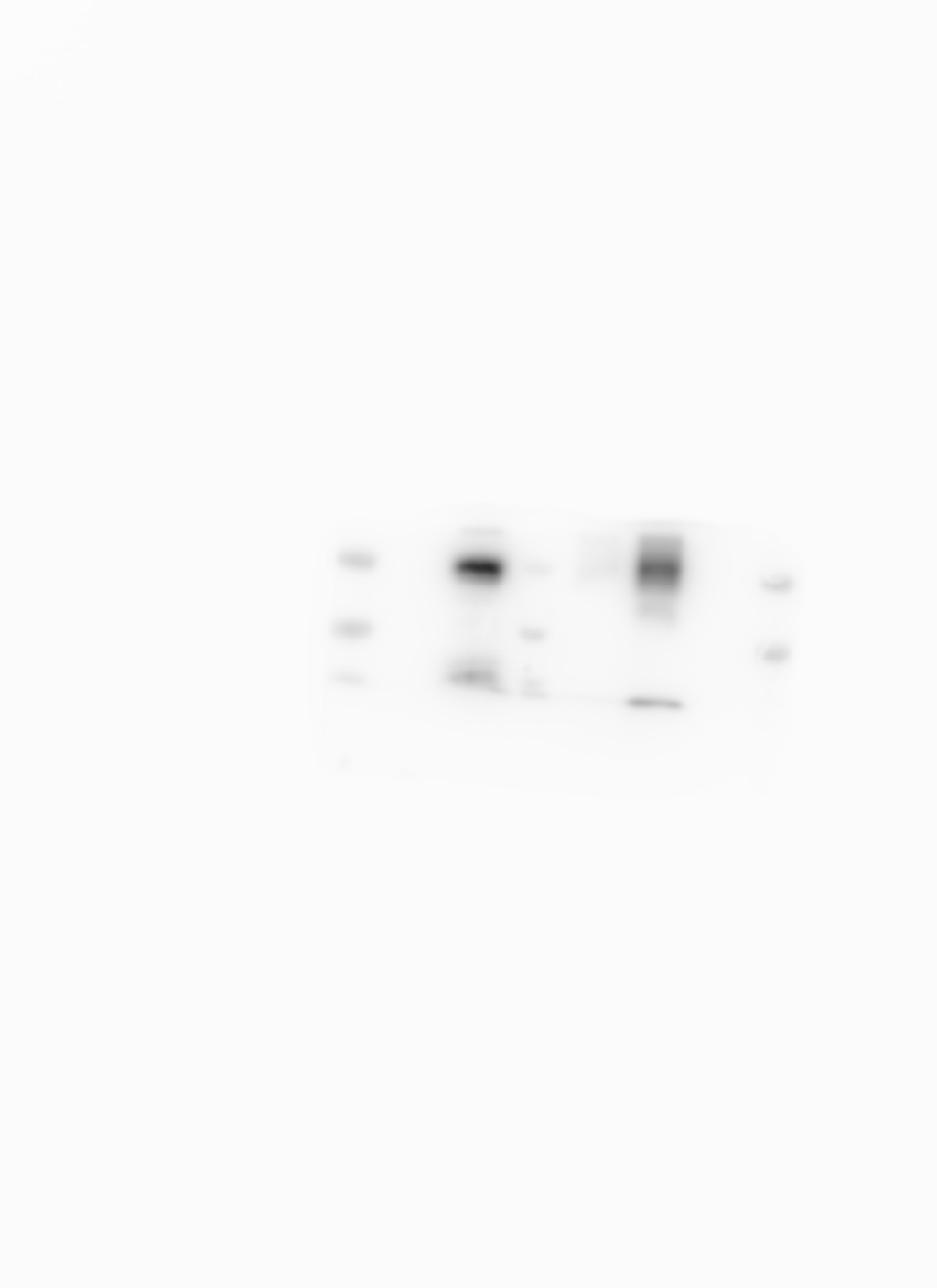

Supplement: Supplementary file 3 — WB Raw data [file 41420_2025_2583_MOESM3_ESM.zip › Figure 6 Panel E/dcaf13 2022.04.09_12.35.56_Ch/dcaf13 2022.04.09_12.35.56_Ch.tif]

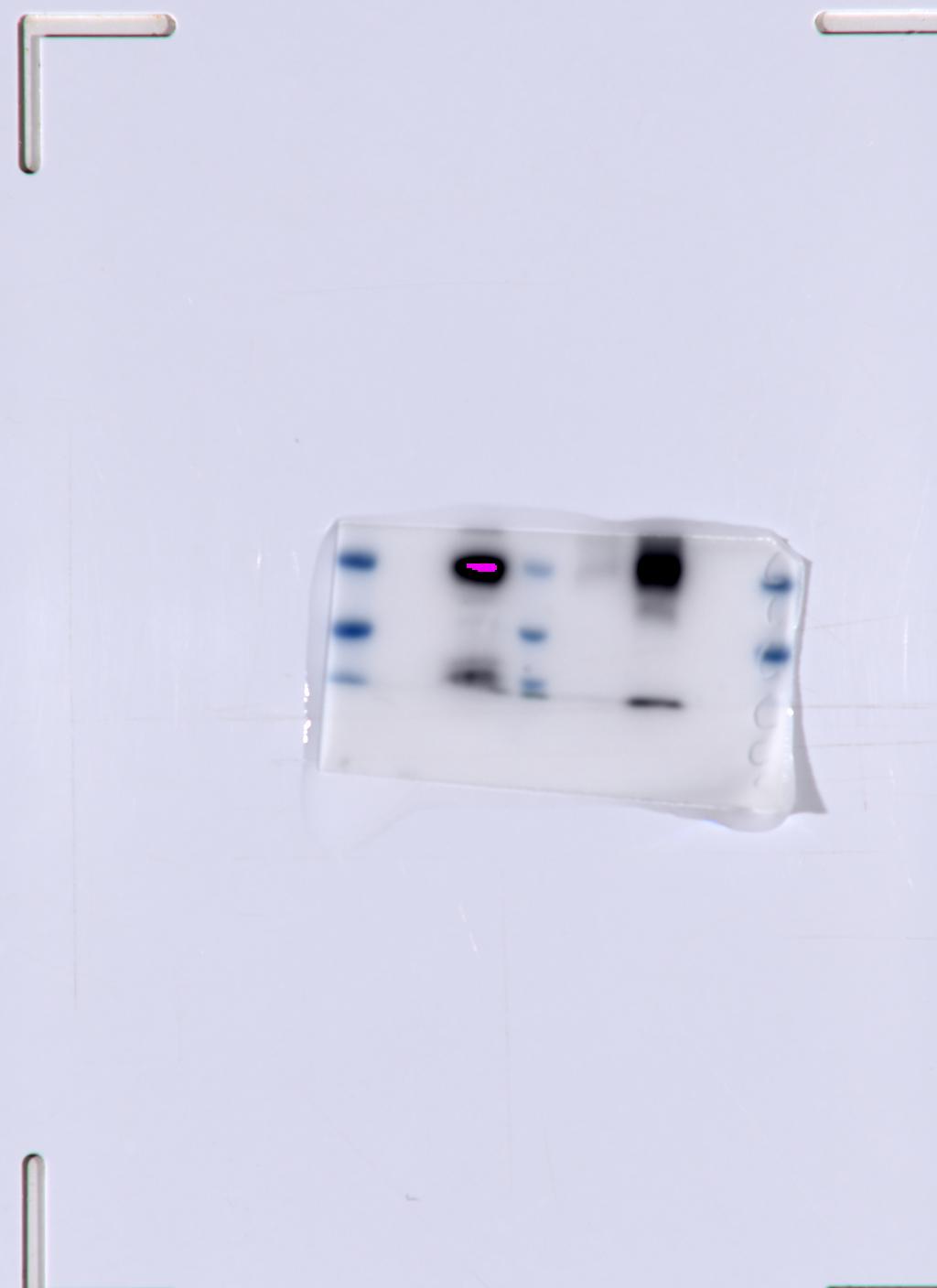

Supplement: Supplementary file 3 — WB Raw data [file 41420_2025_2583_MOESM3_ESM.zip › Figure 6 Panel E/dcaf13 2022.04.09_12.37.12_Ch/dcaf13 2022.04.09_12.37.12_Ch+Marker.jpg]

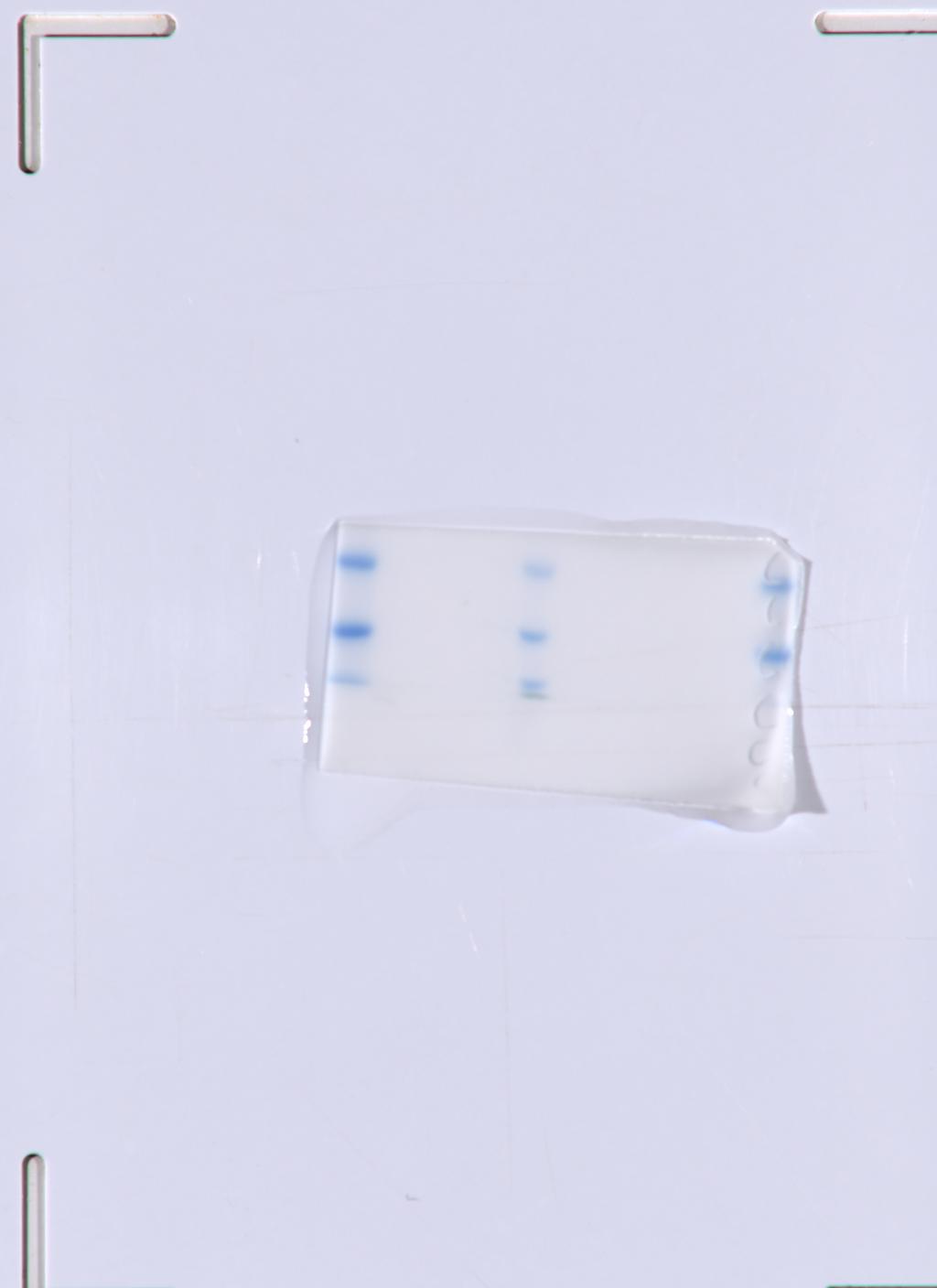

Supplement: Supplementary file 3 — WB Raw data [file 41420_2025_2583_MOESM3_ESM.zip › Figure 6 Panel E/dcaf13 2022.04.09_12.37.12_Ch/dcaf13 2022.04.09_12.37.12_Ch-Marker.jpg]

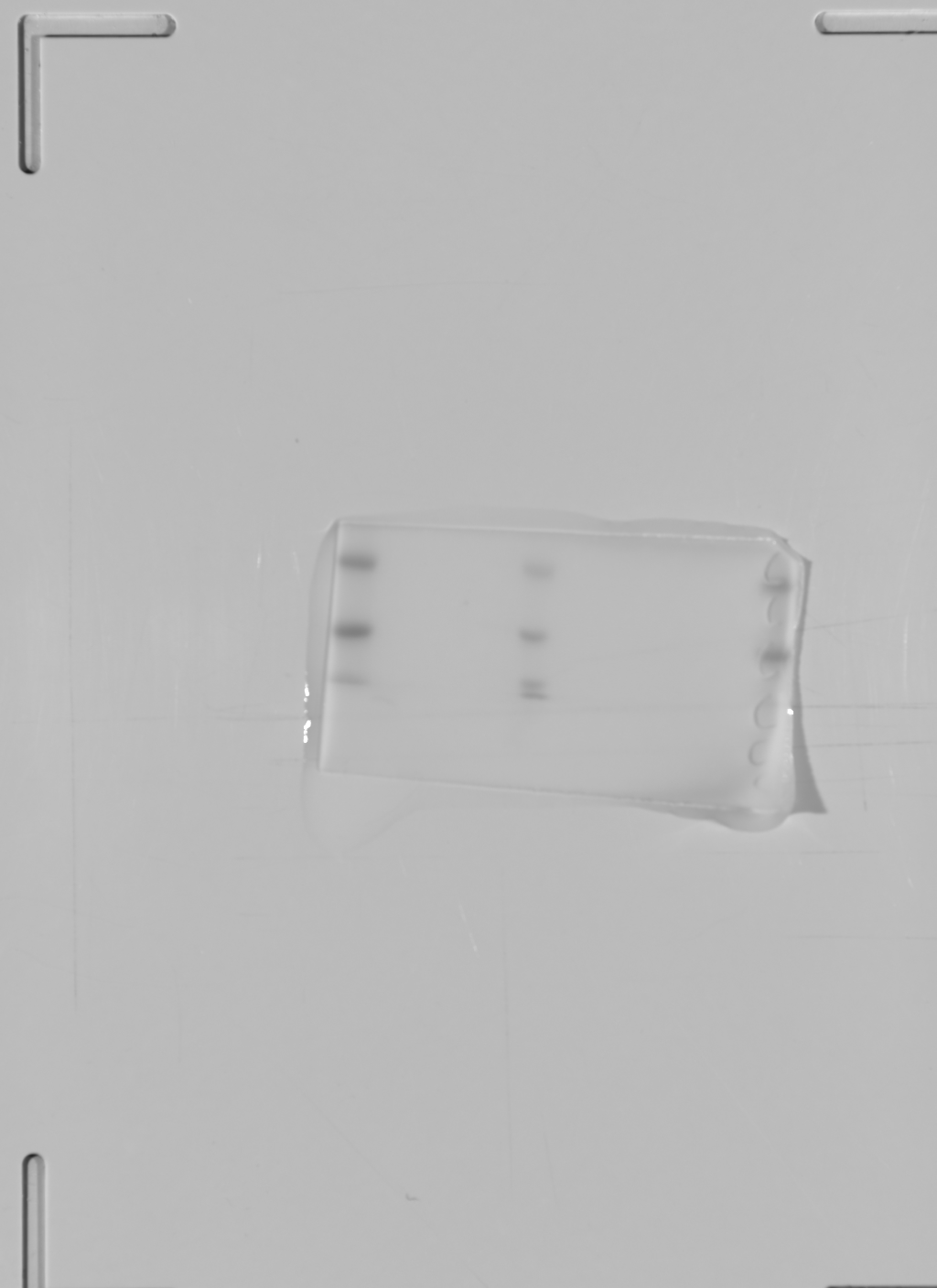

Supplement: Supplementary file 3 — WB Raw data [file 41420_2025_2583_MOESM3_ESM.zip › Figure 6 Panel E/dcaf13 2022.04.09_12.37.12_Ch/dcaf13 2022.04.09_12.37.12_Ch-Marker.tif]

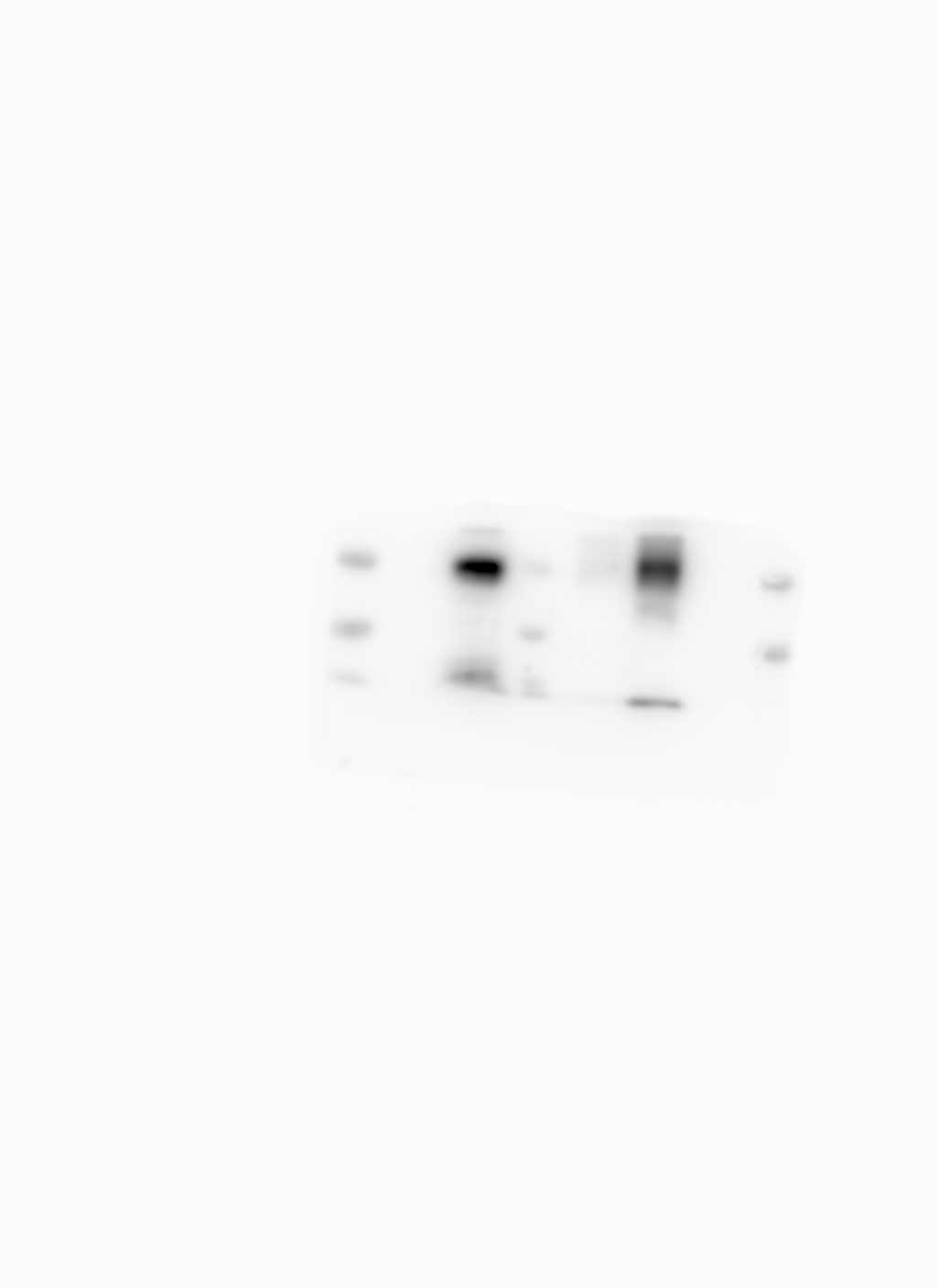

Supplement: Supplementary file 3 — WB Raw data [file 41420_2025_2583_MOESM3_ESM.zip › Figure 6 Panel E/dcaf13 2022.04.09_12.37.12_Ch/dcaf13 2022.04.09_12.37.12_Ch.tif]

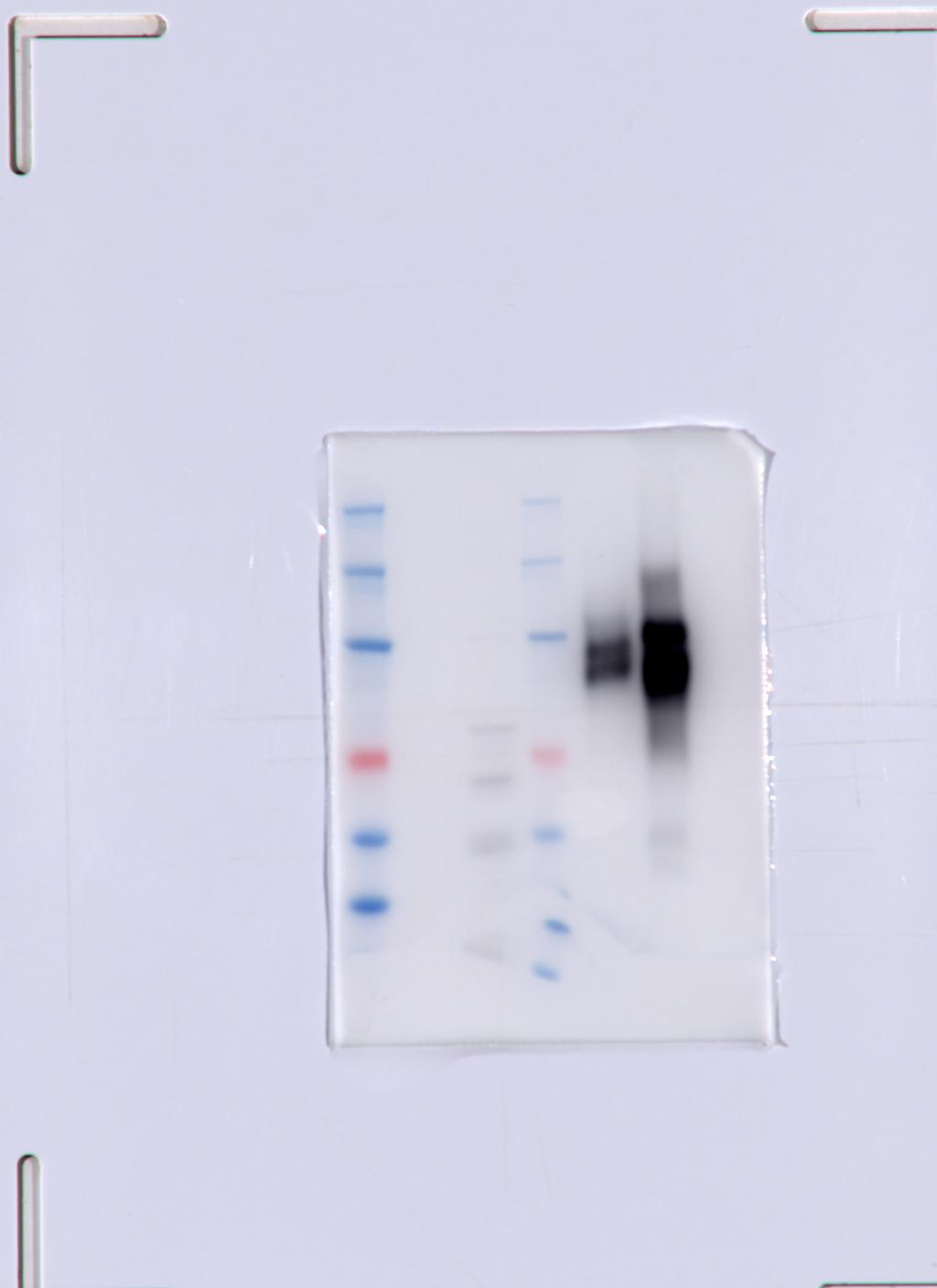

Supplement: Supplementary file 3 — WB Raw data [file 41420_2025_2583_MOESM3_ESM.zip › Figure 6 Panel E/suv39h2 2022.04.09_12.39.11_Ch/suv39h2 2022.04.09_12.39.11_Ch+Marker.jpg]

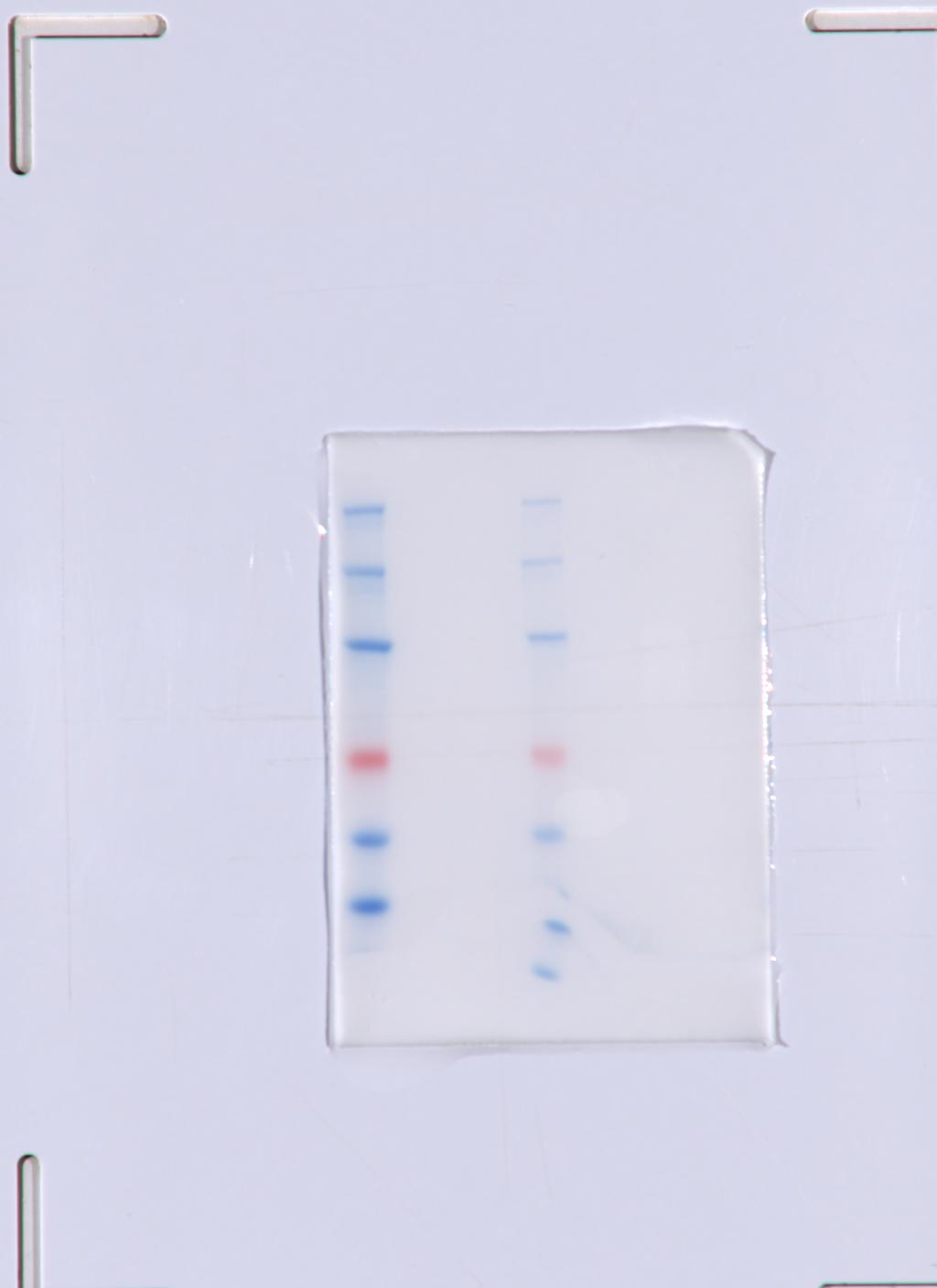

Supplement: Supplementary file 3 — WB Raw data [file 41420_2025_2583_MOESM3_ESM.zip › Figure 6 Panel E/suv39h2 2022.04.09_12.39.11_Ch/suv39h2 2022.04.09_12.39.11_Ch-Marker.jpg]

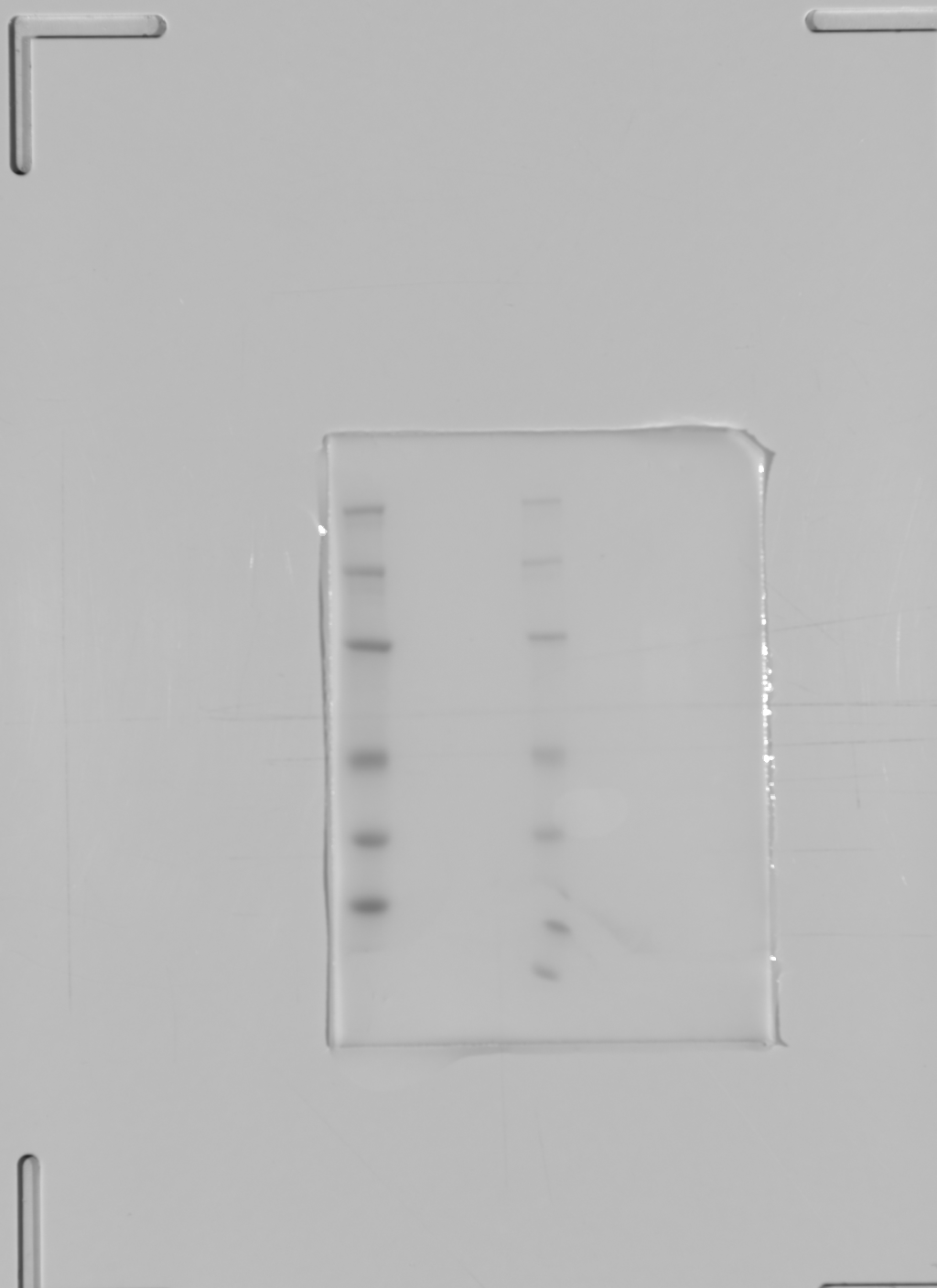

Supplement: Supplementary file 3 — WB Raw data [file 41420_2025_2583_MOESM3_ESM.zip › Figure 6 Panel E/suv39h2 2022.04.09_12.39.11_Ch/suv39h2 2022.04.09_12.39.11_Ch-Marker.tif]

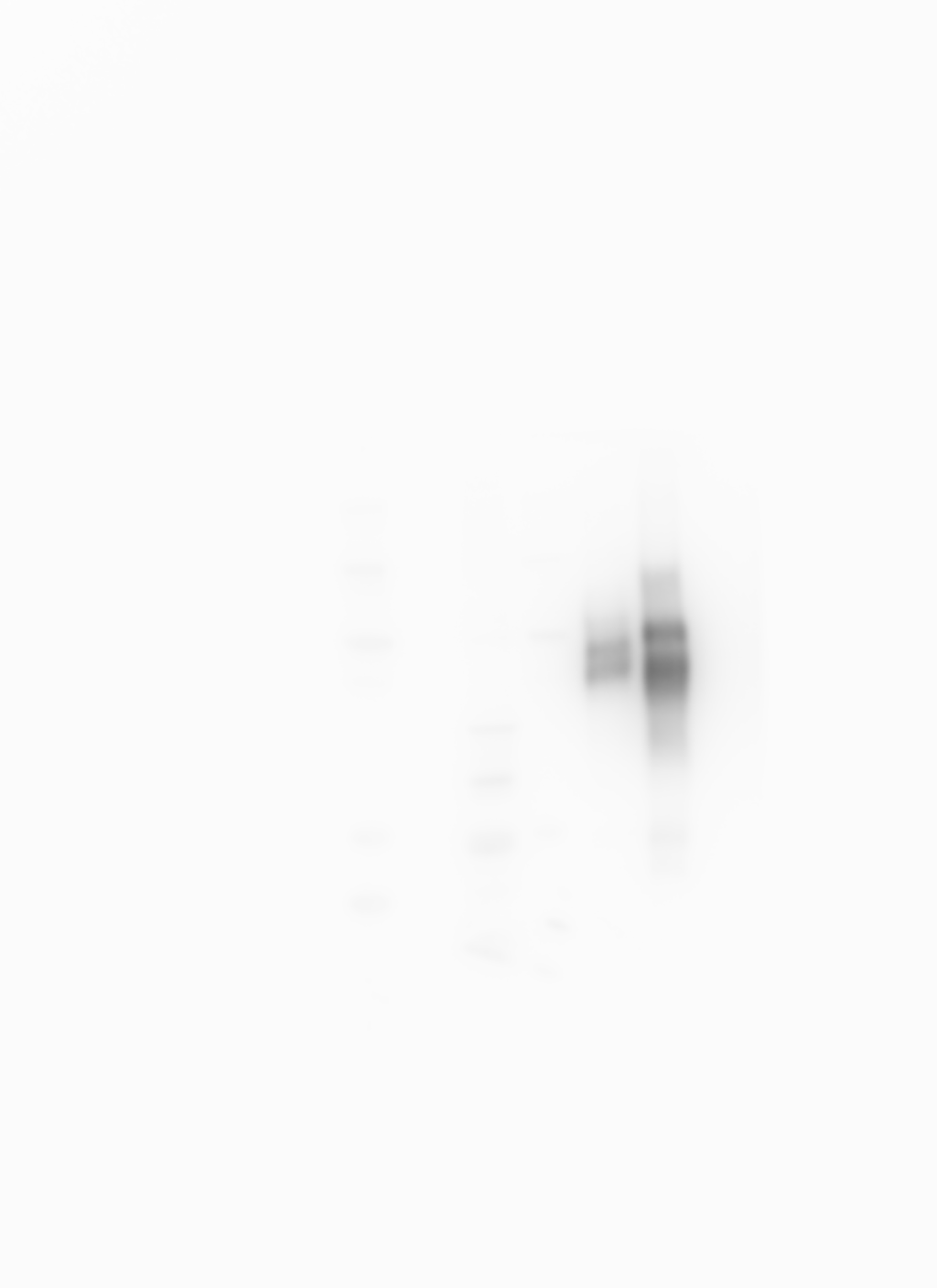

Supplement: Supplementary file 3 — WB Raw data [file 41420_2025_2583_MOESM3_ESM.zip › Figure 6 Panel E/suv39h2 2022.04.09_12.39.11_Ch/suv39h2 2022.04.09_12.39.11_Ch.tif]

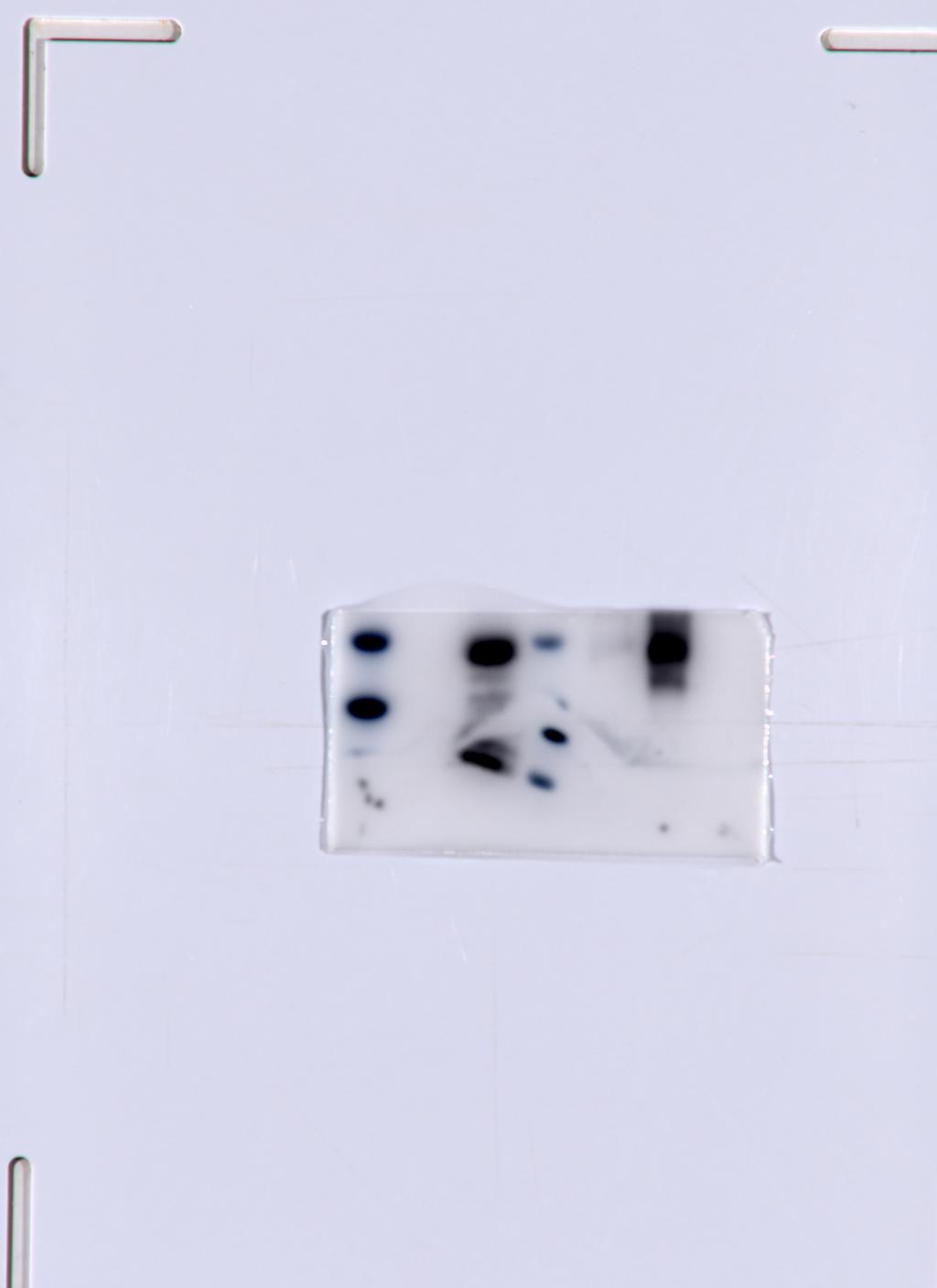

Supplement: Supplementary file 3 — WB Raw data [file 41420_2025_2583_MOESM3_ESM.zip › Figure 6 Panel E/suv39h2 2022.04.09_12.41.01_Ch/suv39h2 2022.04.09_12.41.01_Ch+Marker.jpg]

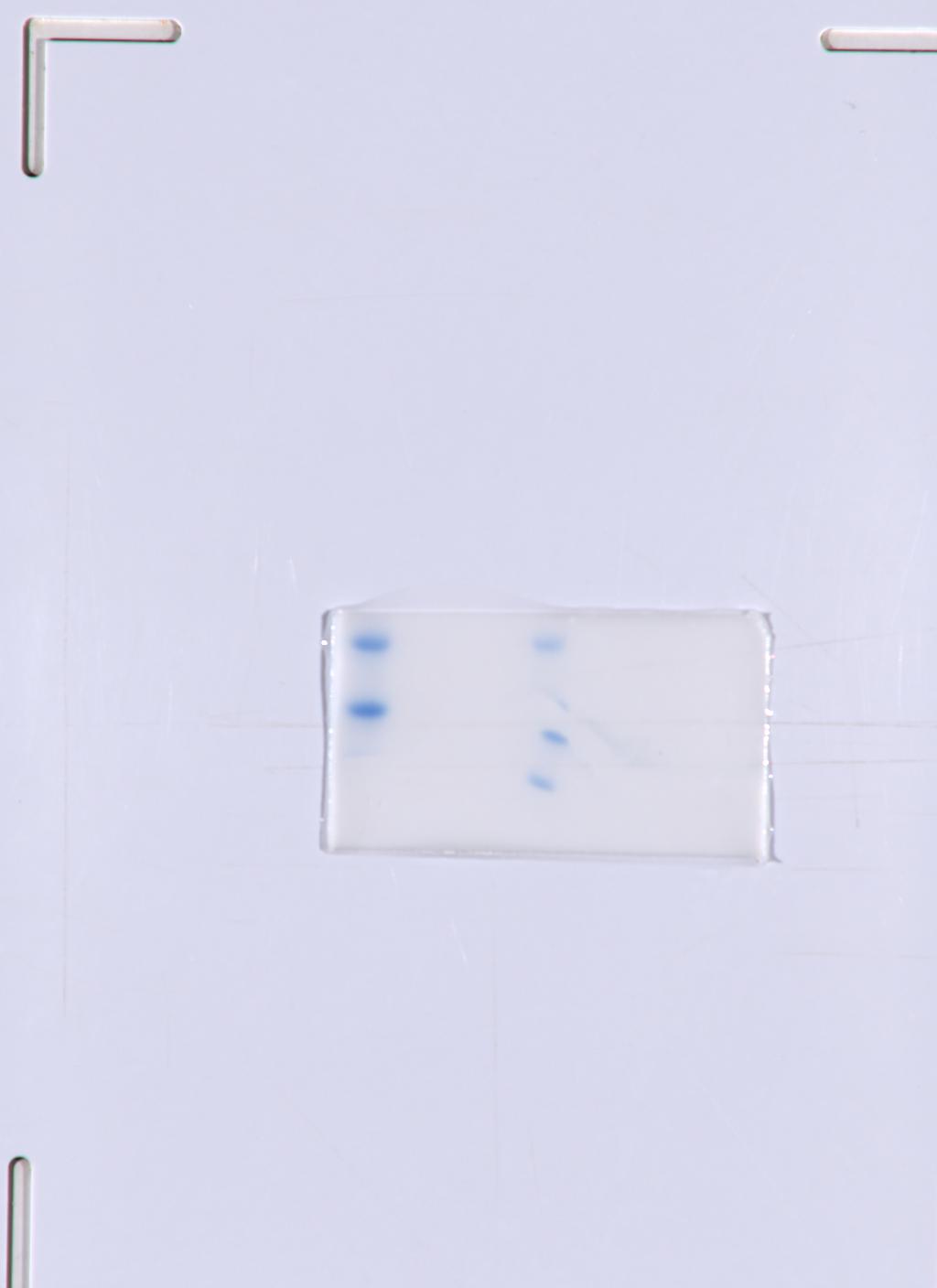

Supplement: Supplementary file 3 — WB Raw data [file 41420_2025_2583_MOESM3_ESM.zip › Figure 6 Panel E/suv39h2 2022.04.09_12.41.01_Ch/suv39h2 2022.04.09_12.41.01_Ch-Marker.jpg]

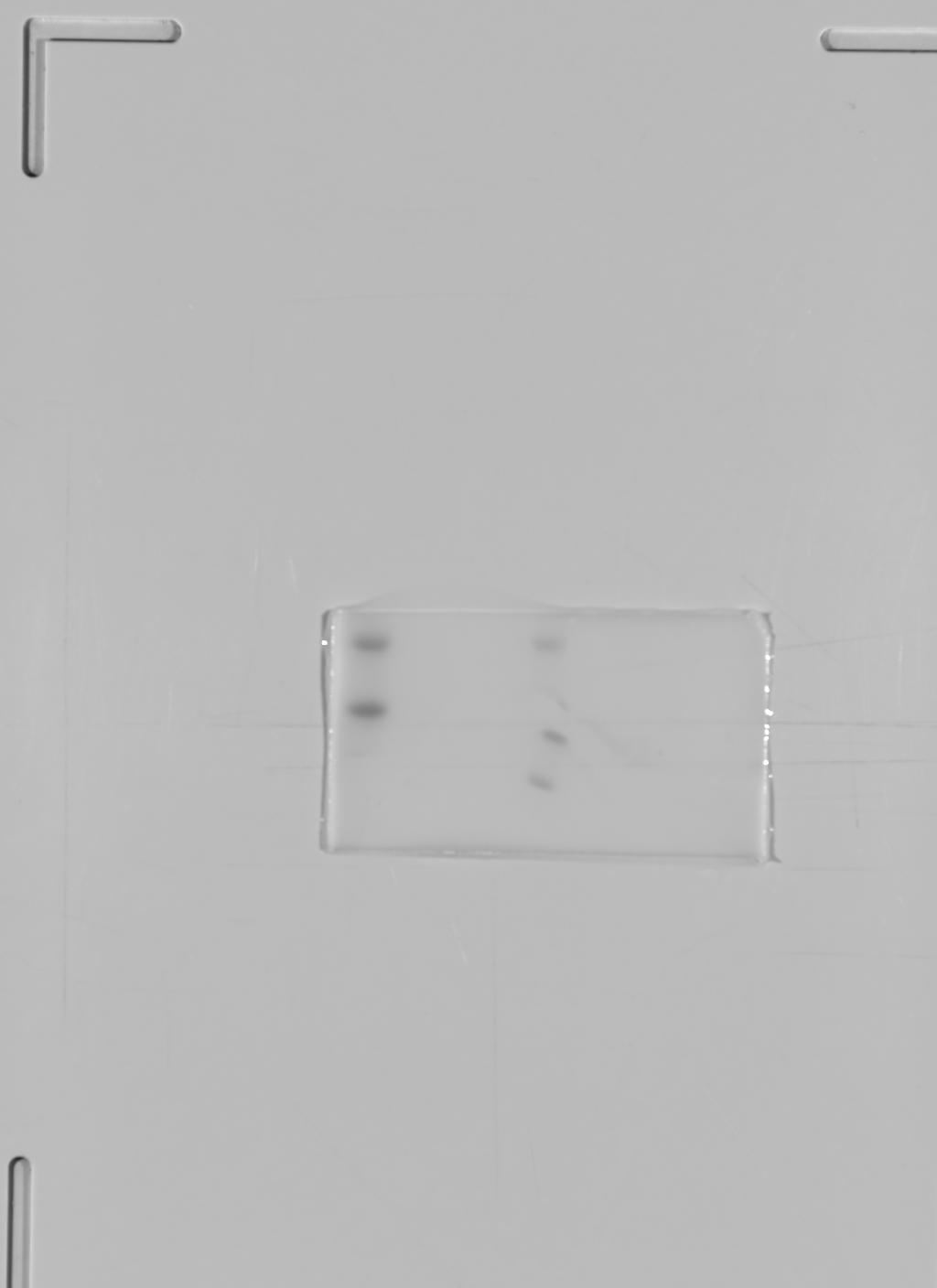

Supplement: Supplementary file 3 — WB Raw data [file 41420_2025_2583_MOESM3_ESM.zip › Figure 6 Panel E/suv39h2 2022.04.09_12.41.01_Ch/suv39h2 2022.04.09_12.41.01_Ch-Marker.tif]

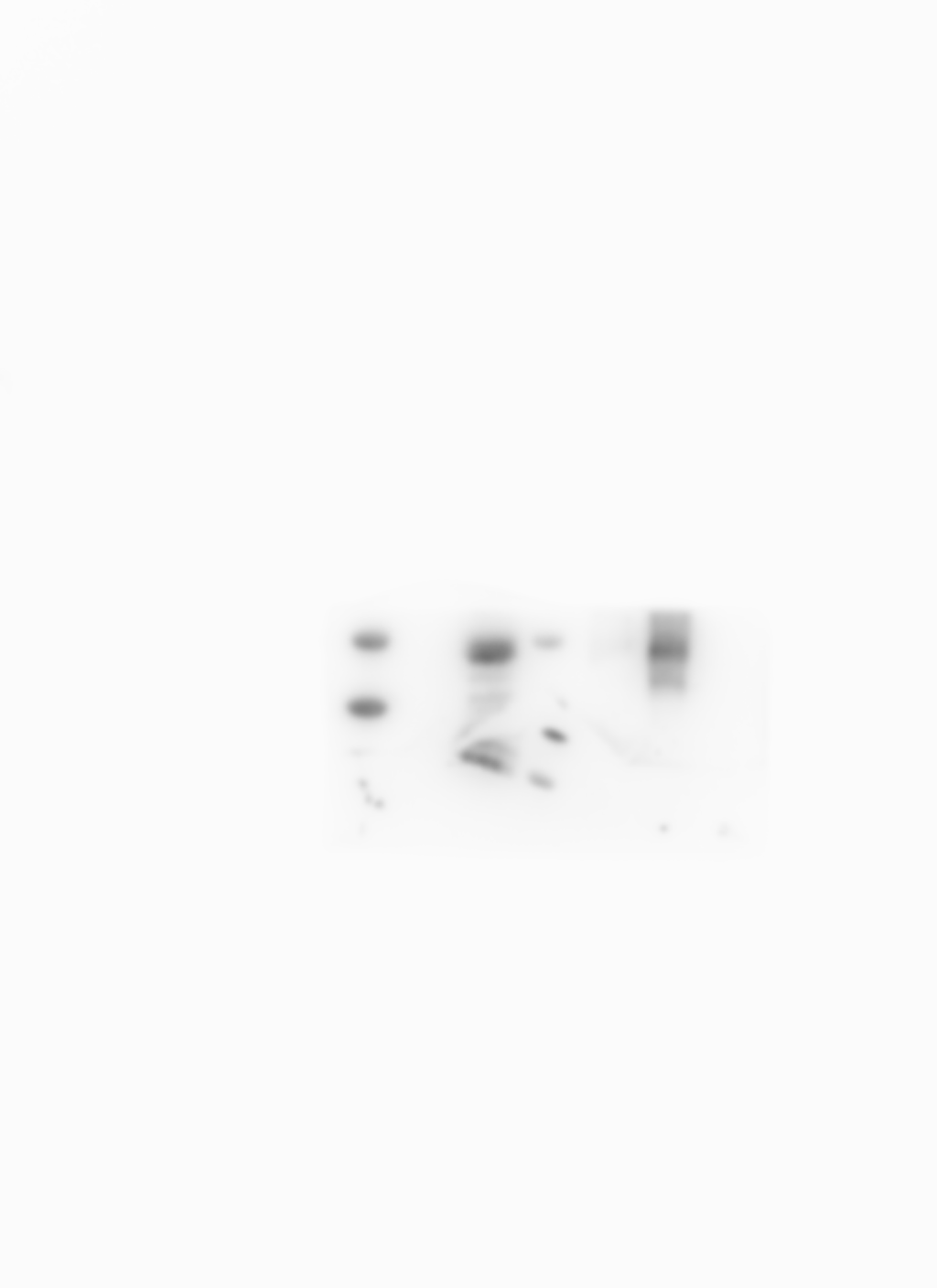

Supplement: Supplementary file 3 — WB Raw data [file 41420_2025_2583_MOESM3_ESM.zip › Figure 6 Panel E/suv39h2 2022.04.09_12.41.01_Ch/suv39h2 2022.04.09_12.41.01_Ch.tif]

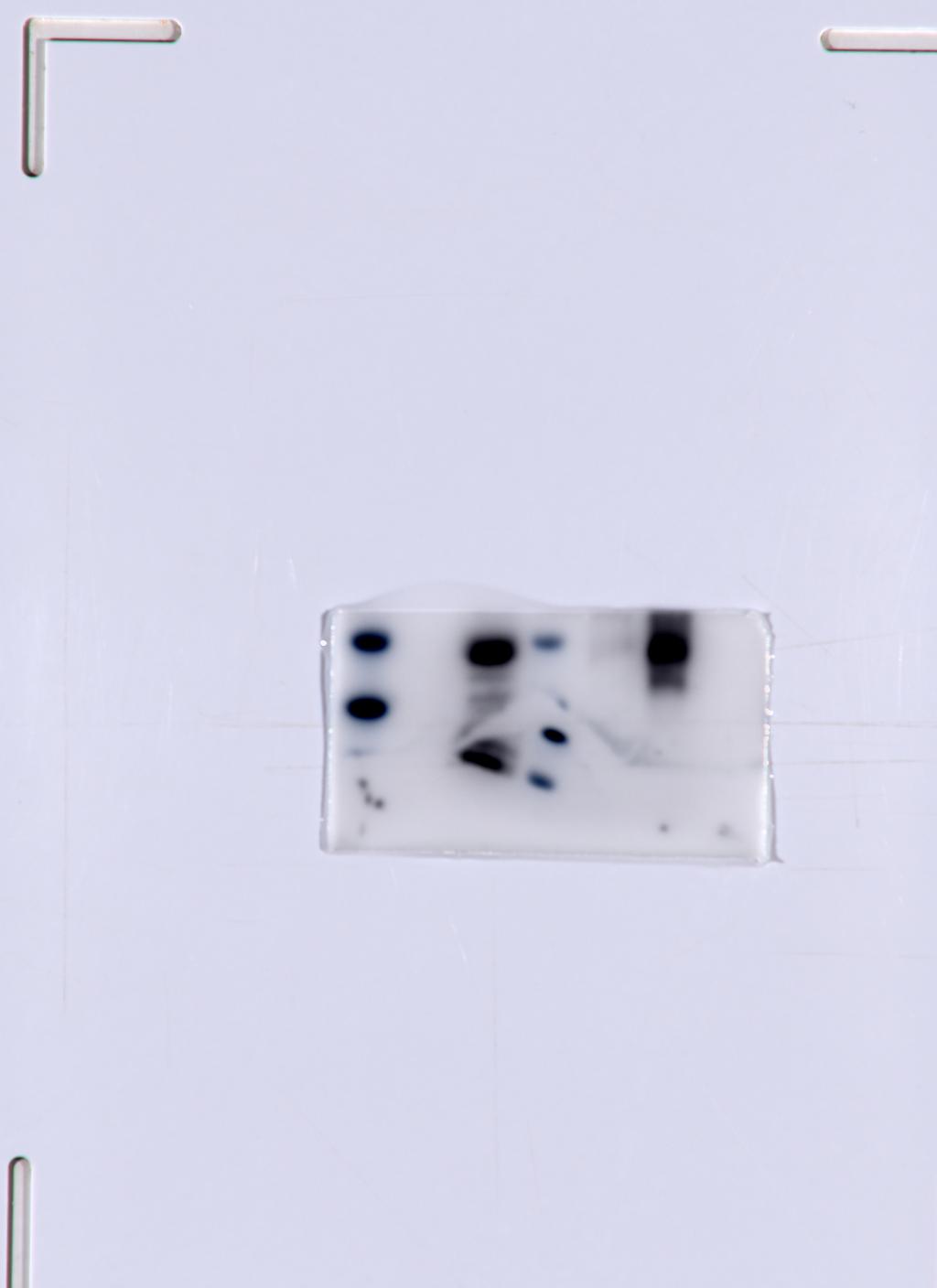

Supplement: Supplementary file 3 — WB Raw data [file 41420_2025_2583_MOESM3_ESM.zip › Figure 6 Panel E/suv39h2 2022.04.09_12.42.25_Ch/suv39h2 2022.04.09_12.42.25_Ch+Marker.jpg]

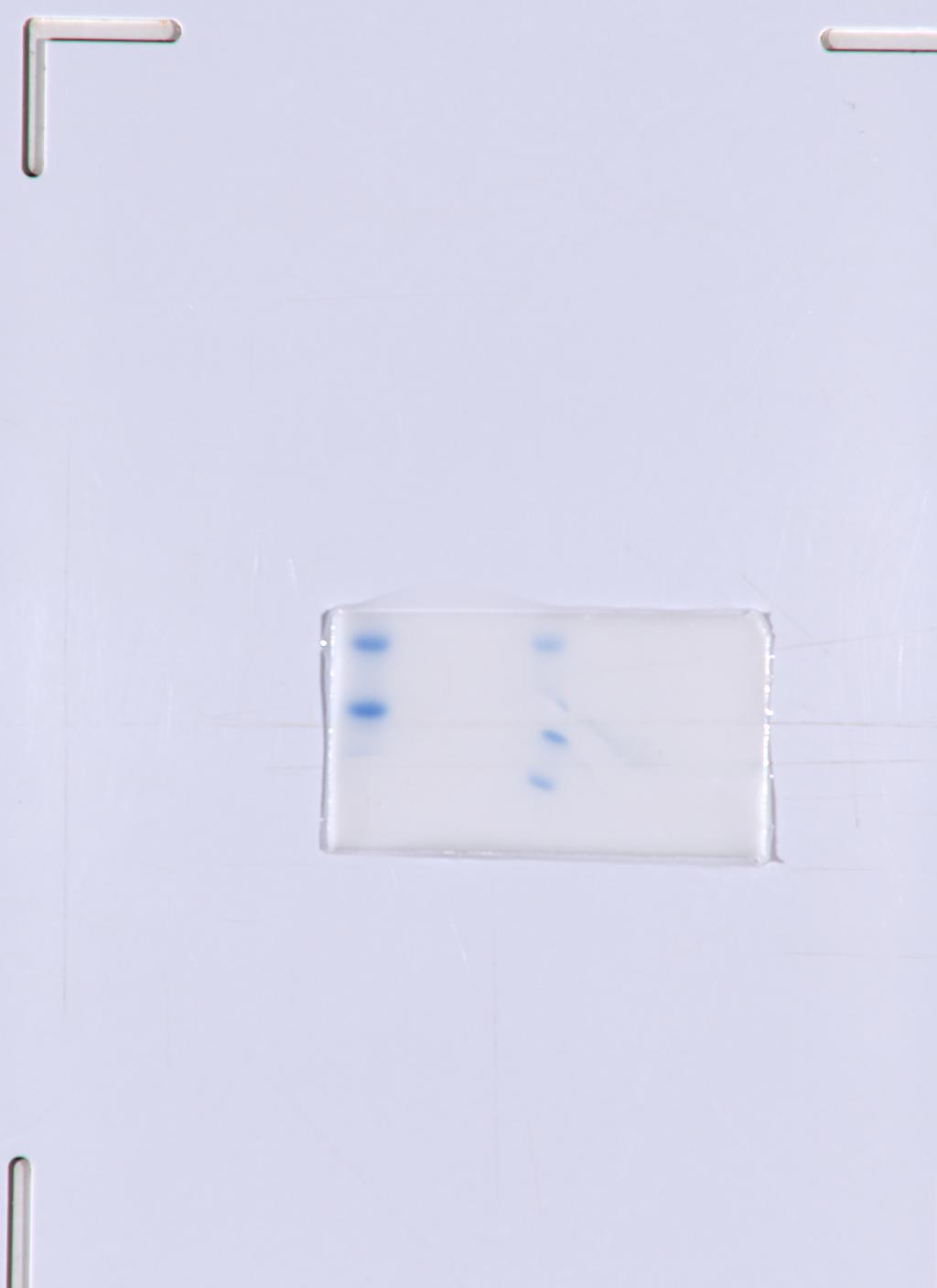

Supplement: Supplementary file 3 — WB Raw data [file 41420_2025_2583_MOESM3_ESM.zip › Figure 6 Panel E/suv39h2 2022.04.09_12.42.25_Ch/suv39h2 2022.04.09_12.42.25_Ch-Marker.jpg]

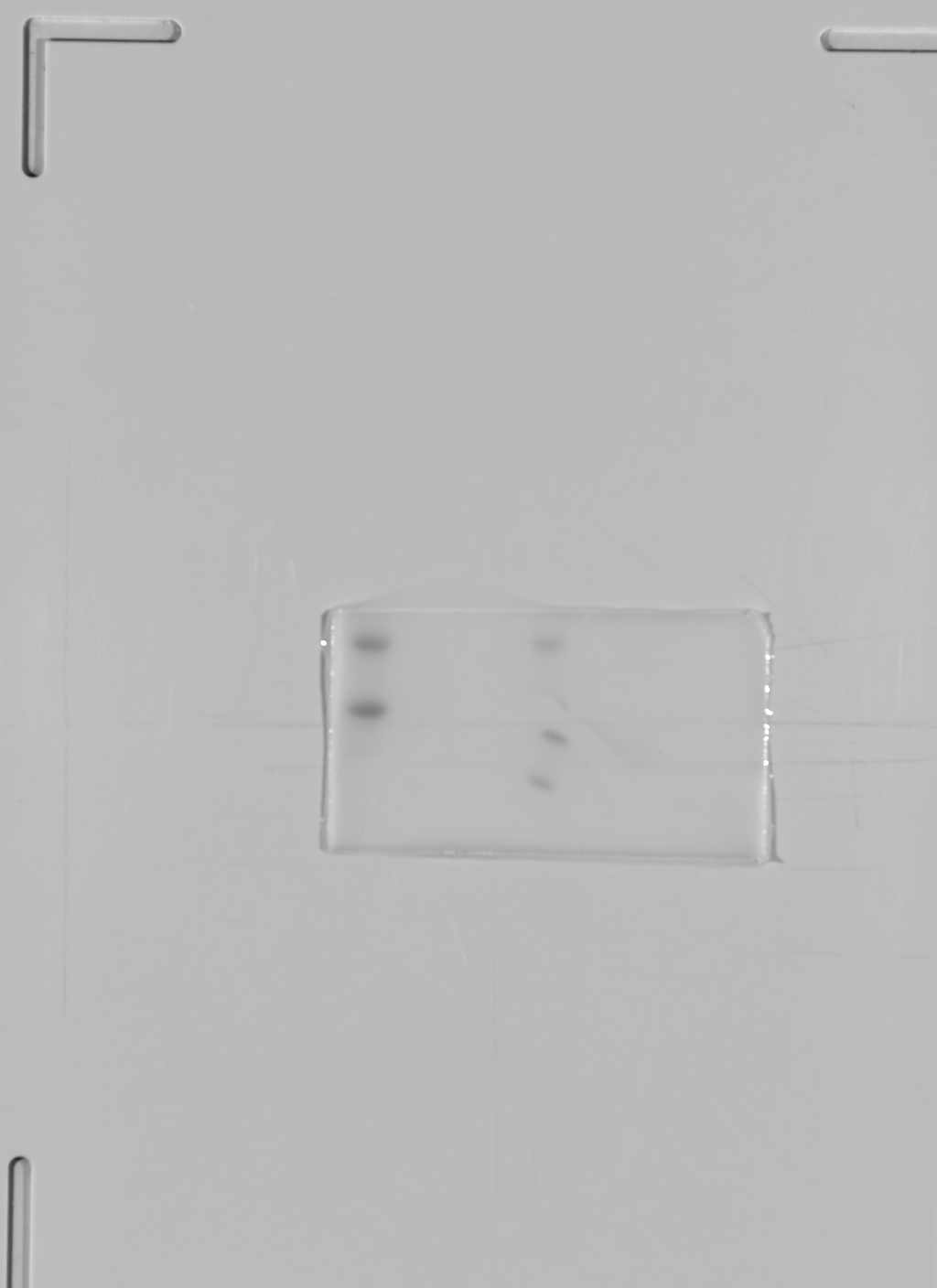

Supplement: Supplementary file 3 — WB Raw data [file 41420_2025_2583_MOESM3_ESM.zip › Figure 6 Panel E/suv39h2 2022.04.09_12.42.25_Ch/suv39h2 2022.04.09_12.42.25_Ch-Marker.tif]

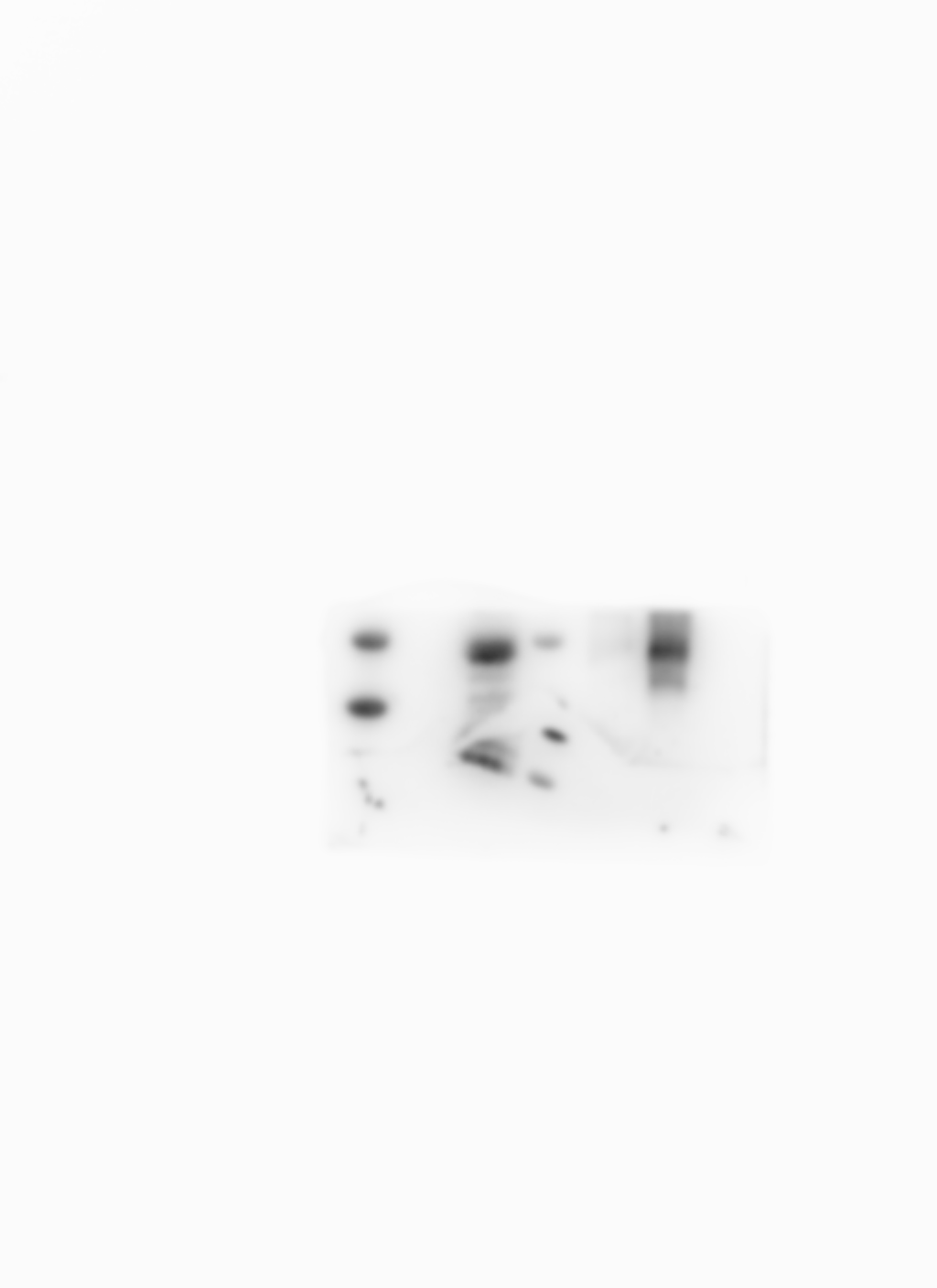

Supplement: Supplementary file 3 — WB Raw data [file 41420_2025_2583_MOESM3_ESM.zip › Figure 6 Panel E/suv39h2 2022.04.09_12.42.25_Ch/suv39h2 2022.04.09_12.42.25_Ch.tif]

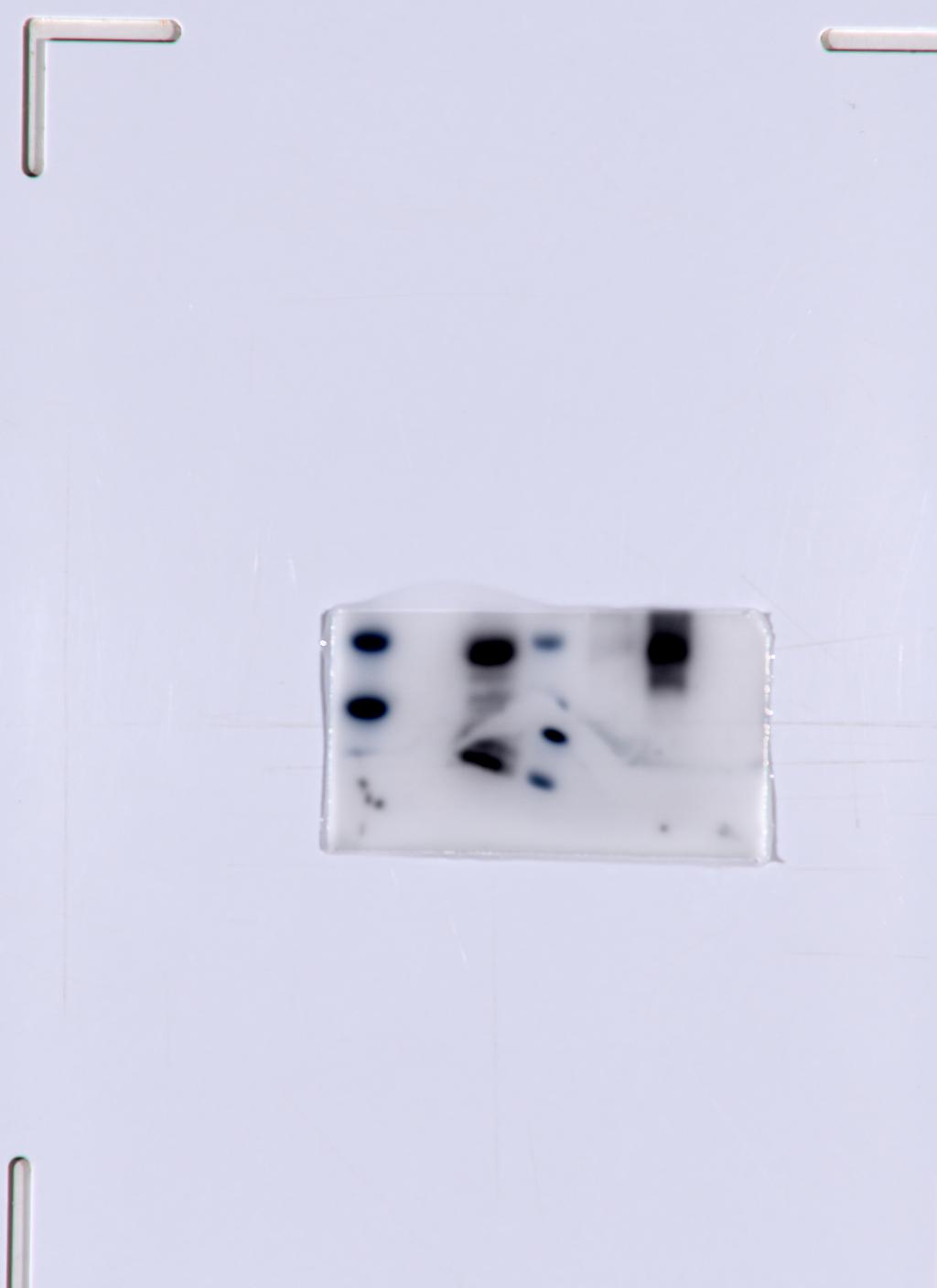

Supplement: Supplementary file 3 — WB Raw data [file 41420_2025_2583_MOESM3_ESM.zip › Figure 6 Panel E/suv39h2 2022.04.09_12.43.45_Ch/suv39h2 2022.04.09_12.43.45_Ch+Marker.jpg]

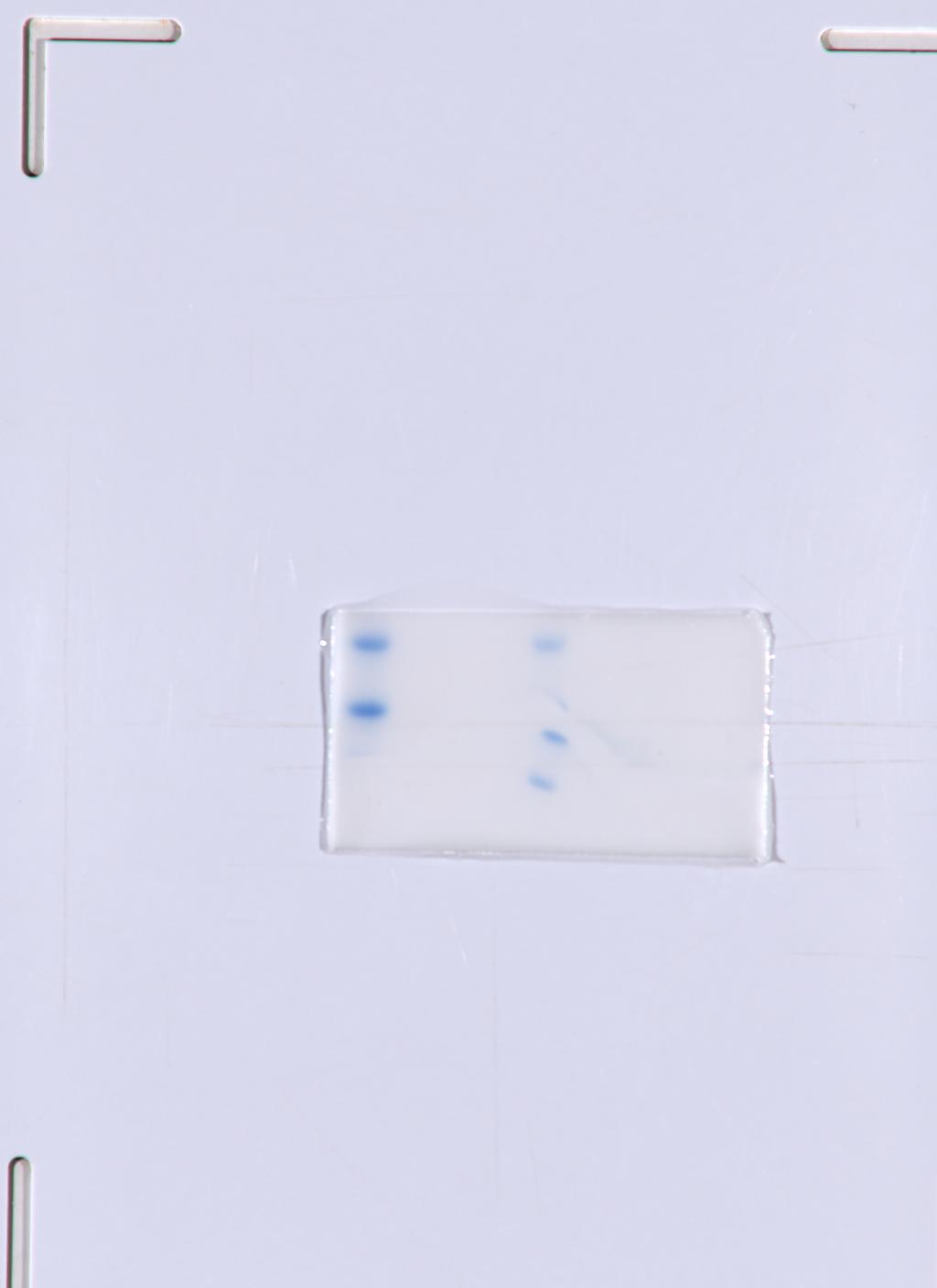

Supplement: Supplementary file 3 — WB Raw data [file 41420_2025_2583_MOESM3_ESM.zip › Figure 6 Panel E/suv39h2 2022.04.09_12.43.45_Ch/suv39h2 2022.04.09_12.43.45_Ch-Marker.jpg]

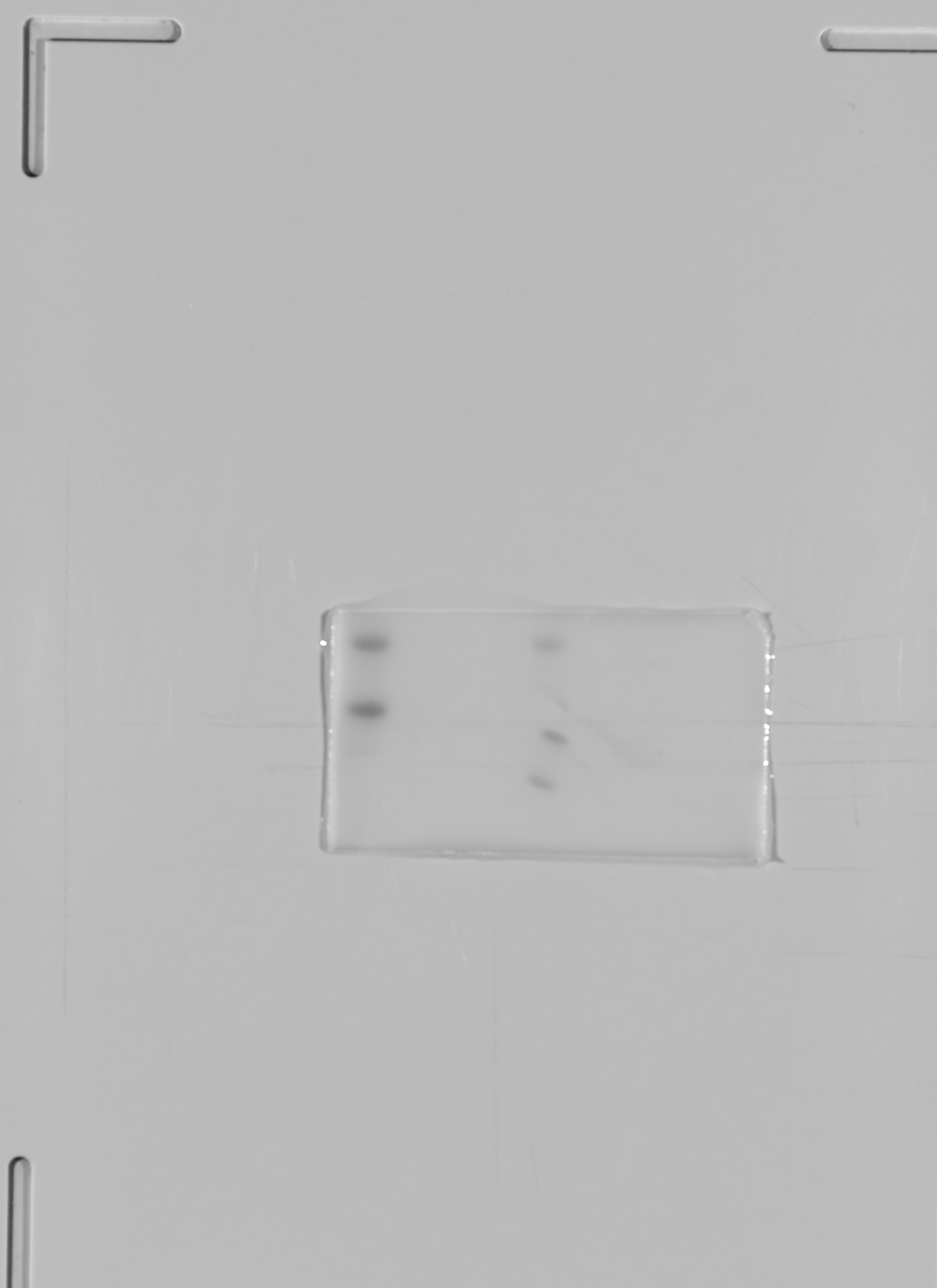

Supplement: Supplementary file 3 — WB Raw data [file 41420_2025_2583_MOESM3_ESM.zip › Figure 6 Panel E/suv39h2 2022.04.09_12.43.45_Ch/suv39h2 2022.04.09_12.43.45_Ch-Marker.tif]

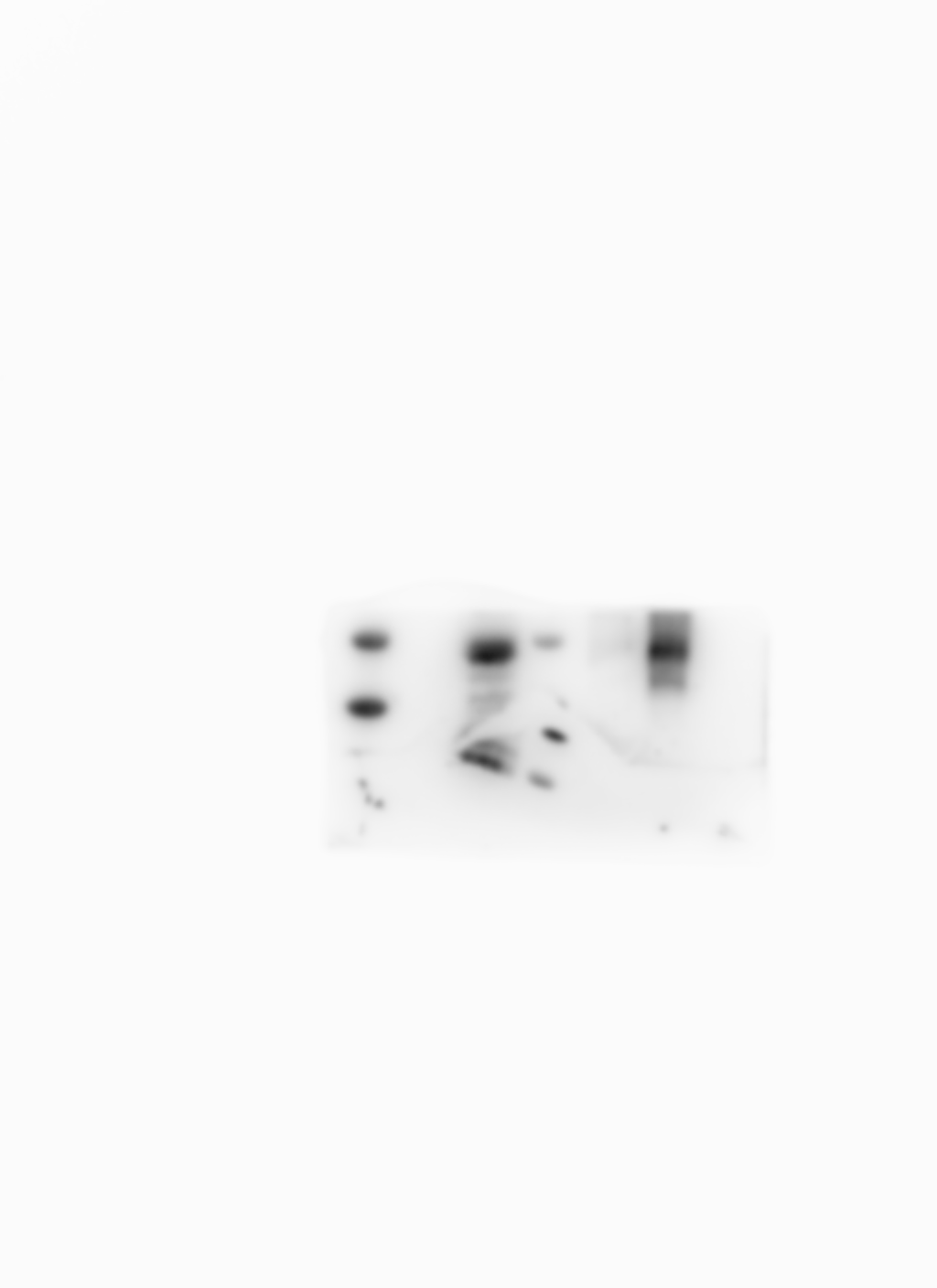

Supplement: Supplementary file 3 — WB Raw data [file 41420_2025_2583_MOESM3_ESM.zip › Figure 6 Panel E/suv39h2 2022.04.09_12.43.45_Ch/suv39h2 2022.04.09_12.43.45_Ch.tif]

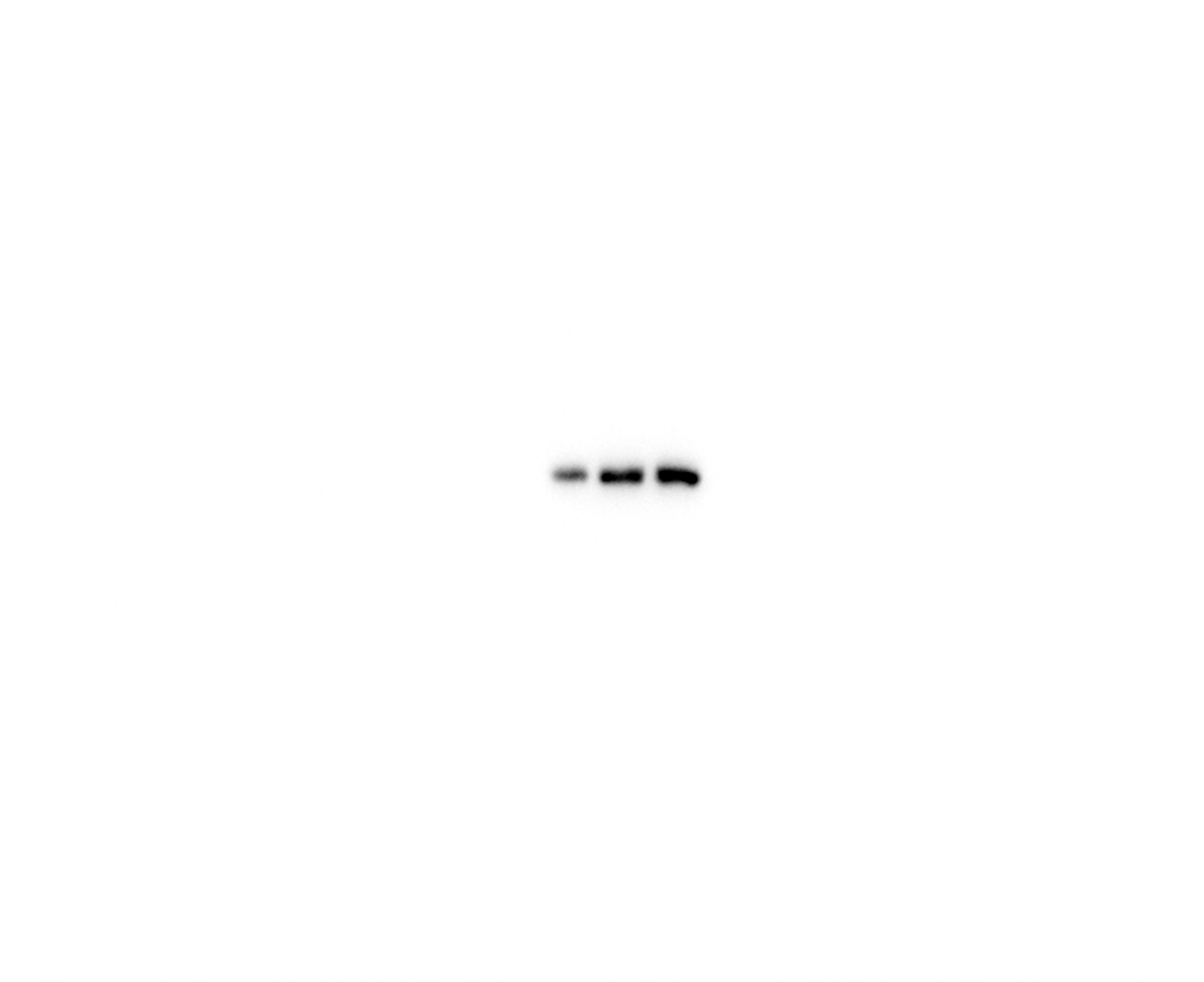

Supplement: Supplementary file 3 — WB Raw data [file 41420_2025_2583_MOESM3_ESM.zip › Figure 6 Panel F/SUV39H2/SUV39H2_1.Tif]

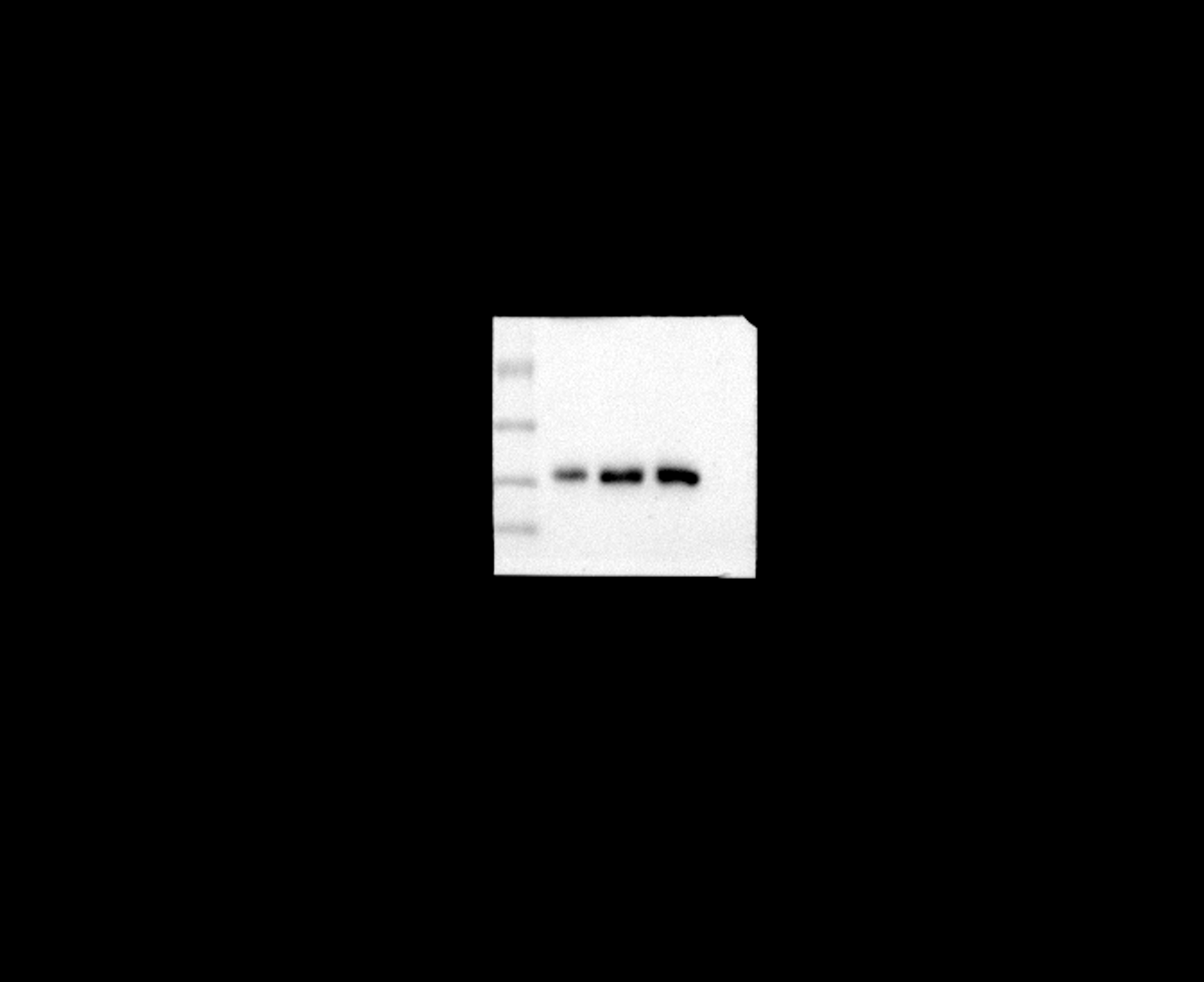

Supplement: Supplementary file 3 — WB Raw data [file 41420_2025_2583_MOESM3_ESM.zip › Figure 6 Panel F/SUV39H2/SUV39H2_1marker.Tif]

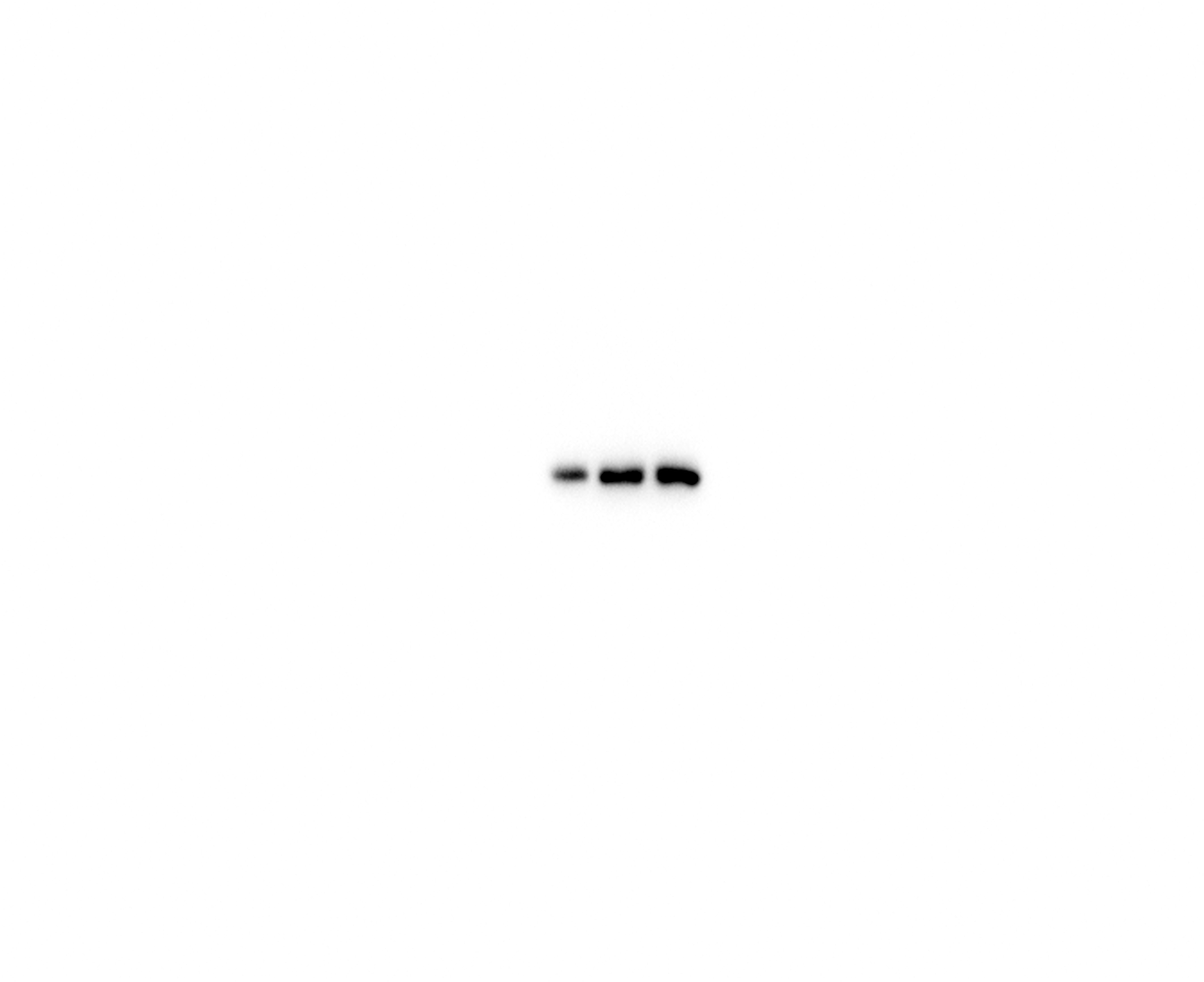

Supplement: Supplementary file 3 — WB Raw data [file 41420_2025_2583_MOESM3_ESM.zip › Figure 6 Panel F/SUV39H2/SUV39H2_2.Tif]

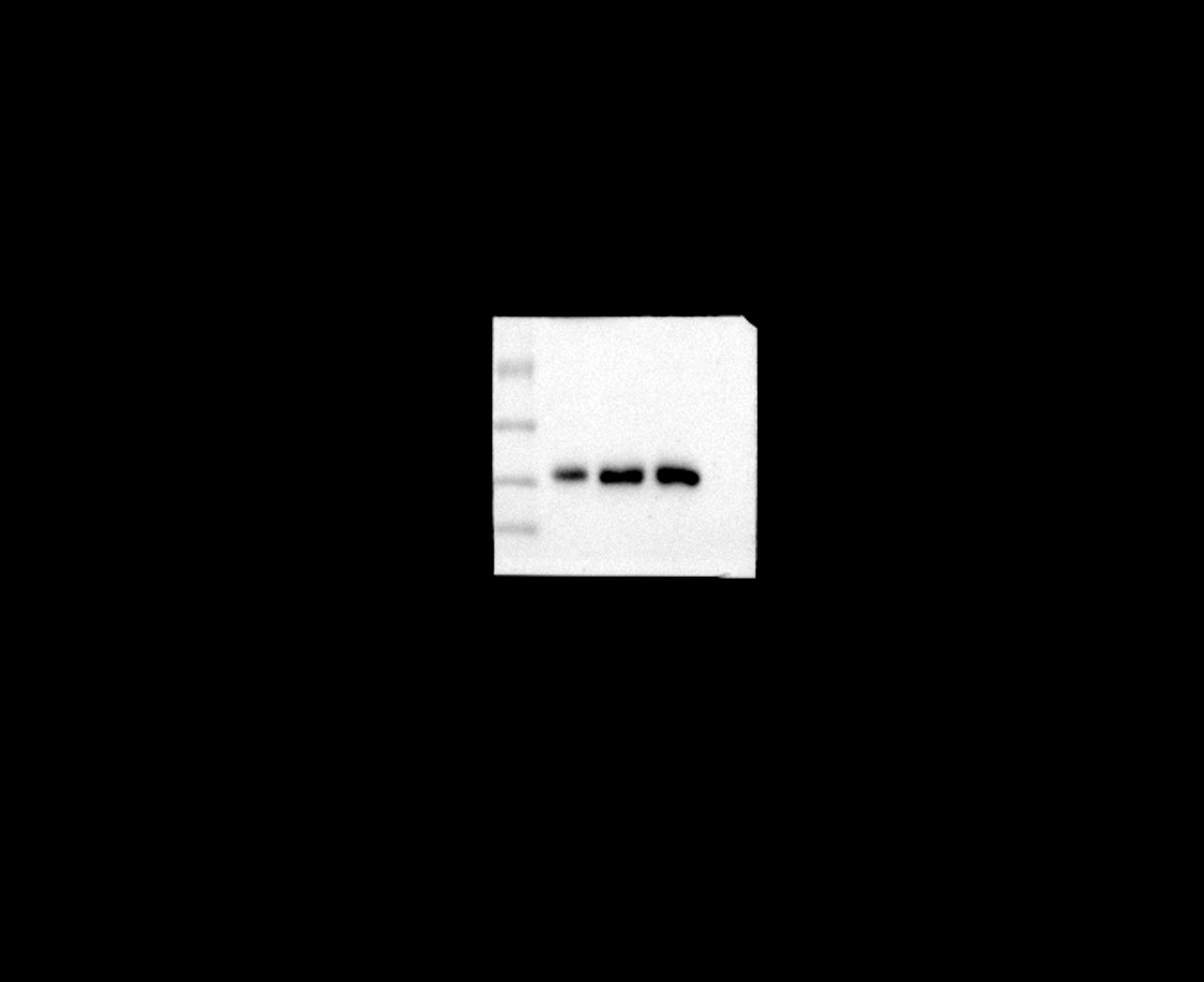

Supplement: Supplementary file 3 — WB Raw data [file 41420_2025_2583_MOESM3_ESM.zip › Figure 6 Panel F/SUV39H2/SUV39H2_2marker.Tif]

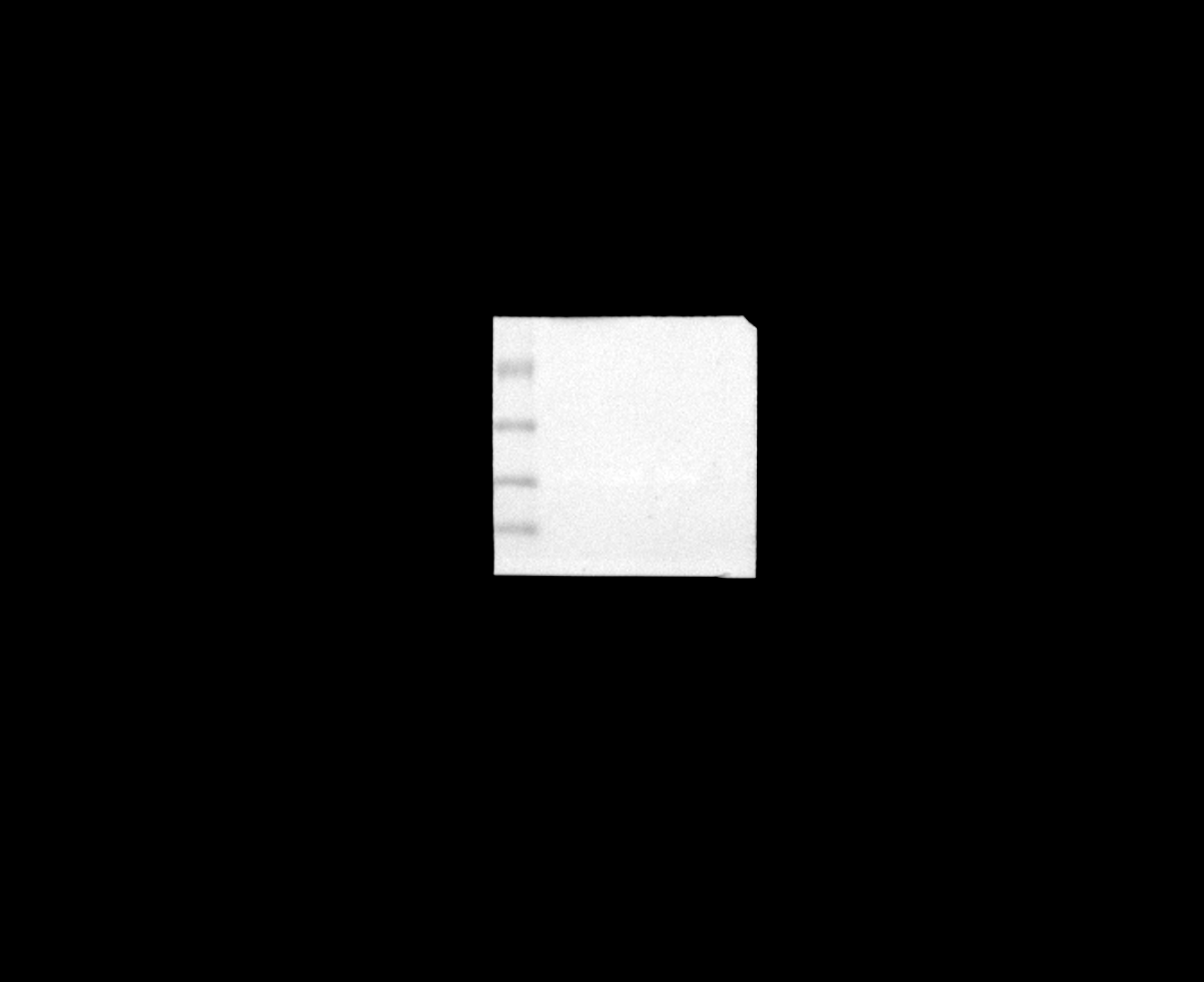

Supplement: Supplementary file 3 — WB Raw data [file 41420_2025_2583_MOESM3_ESM.zip › Figure 6 Panel F/SUV39H2/SUV39H2_marker.Tif]

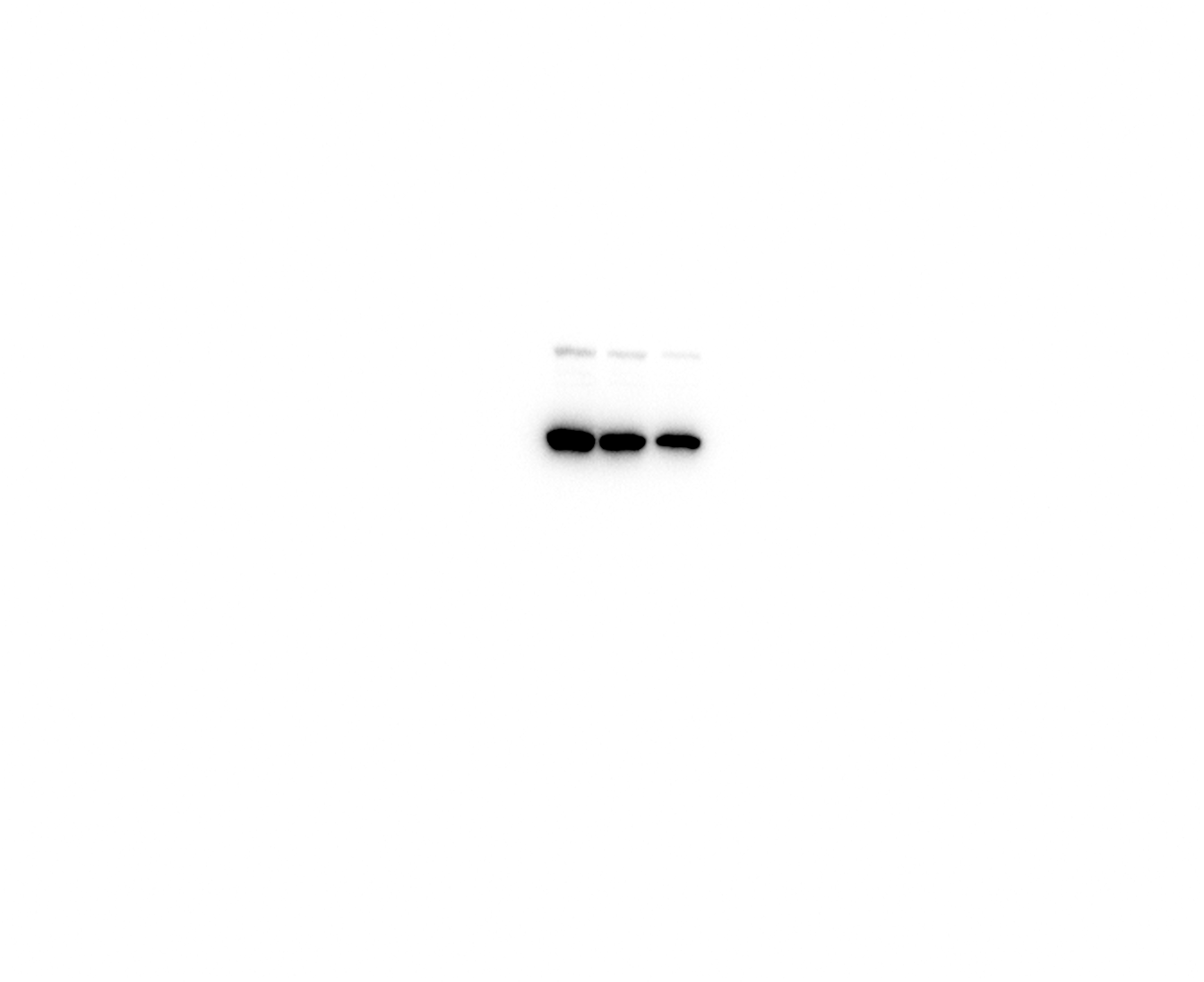

Supplement: Supplementary file 3 — WB Raw data [file 41420_2025_2583_MOESM3_ESM.zip › Figure 6 Panel F/DCAF13/dcaf13_1.Tif]

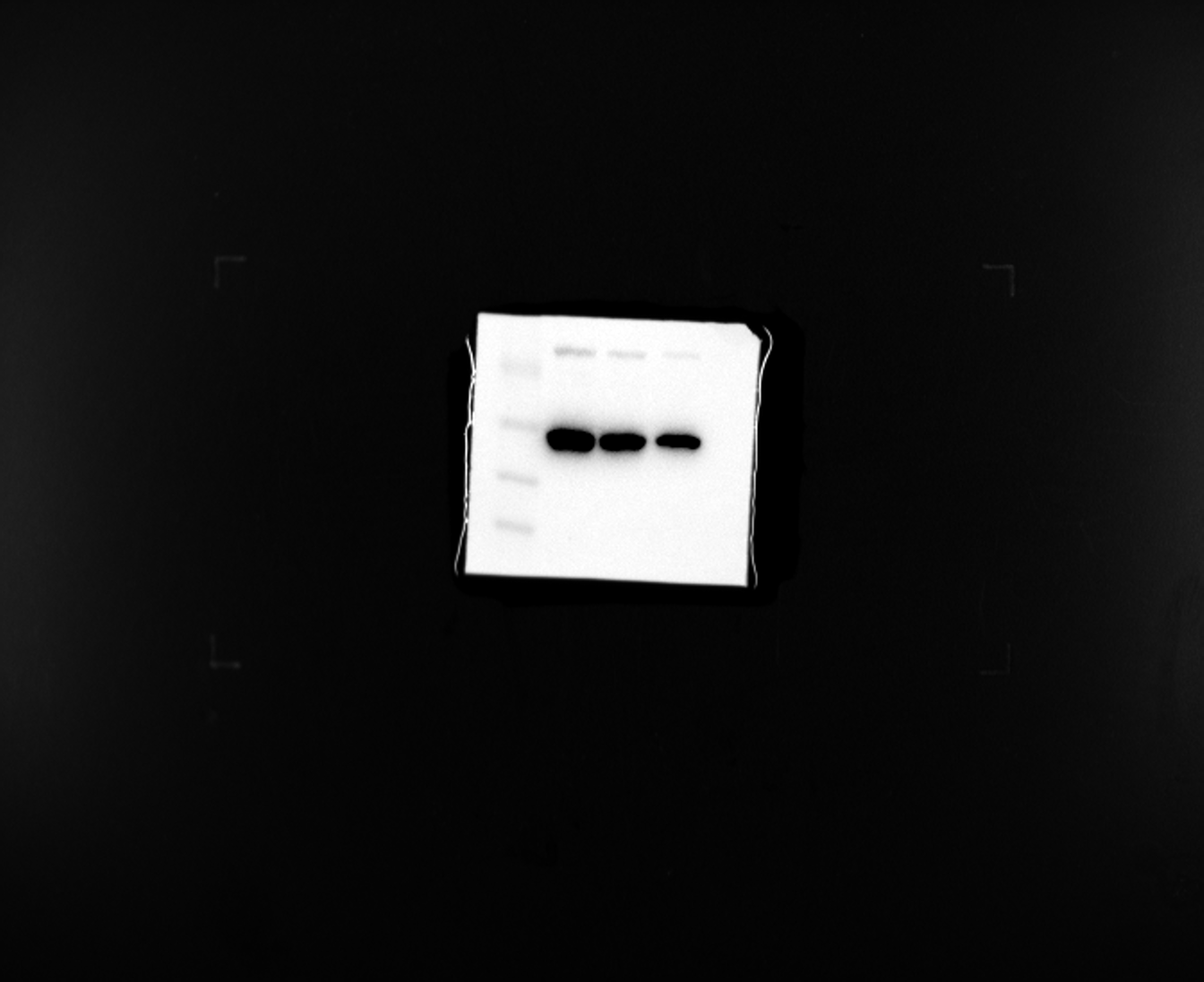

Supplement: Supplementary file 3 — WB Raw data [file 41420_2025_2583_MOESM3_ESM.zip › Figure 6 Panel F/DCAF13/dcaf13_1m.Tif]

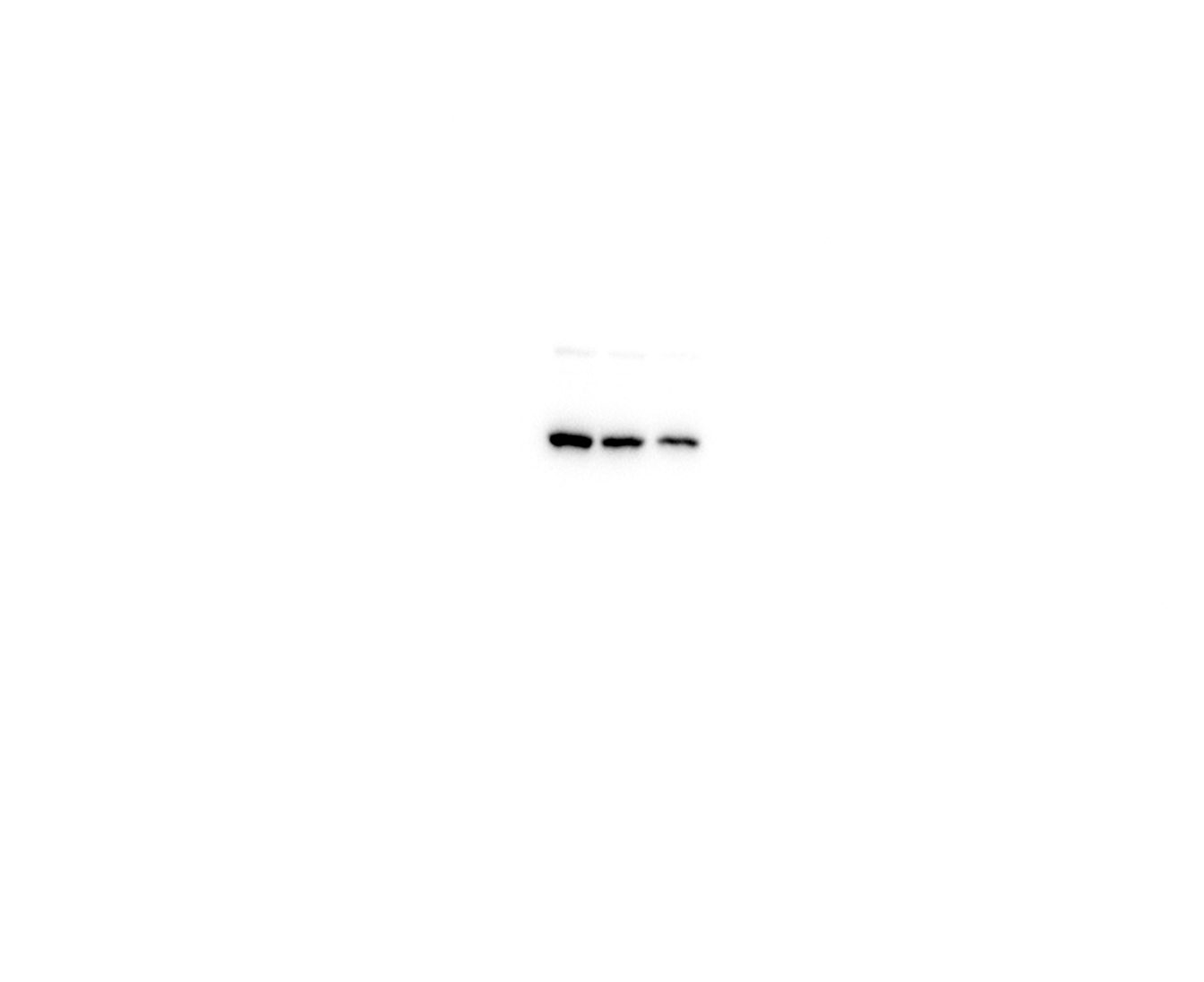

Supplement: Supplementary file 3 — WB Raw data [file 41420_2025_2583_MOESM3_ESM.zip › Figure 6 Panel F/DCAF13/dcaf13_2.Tif]

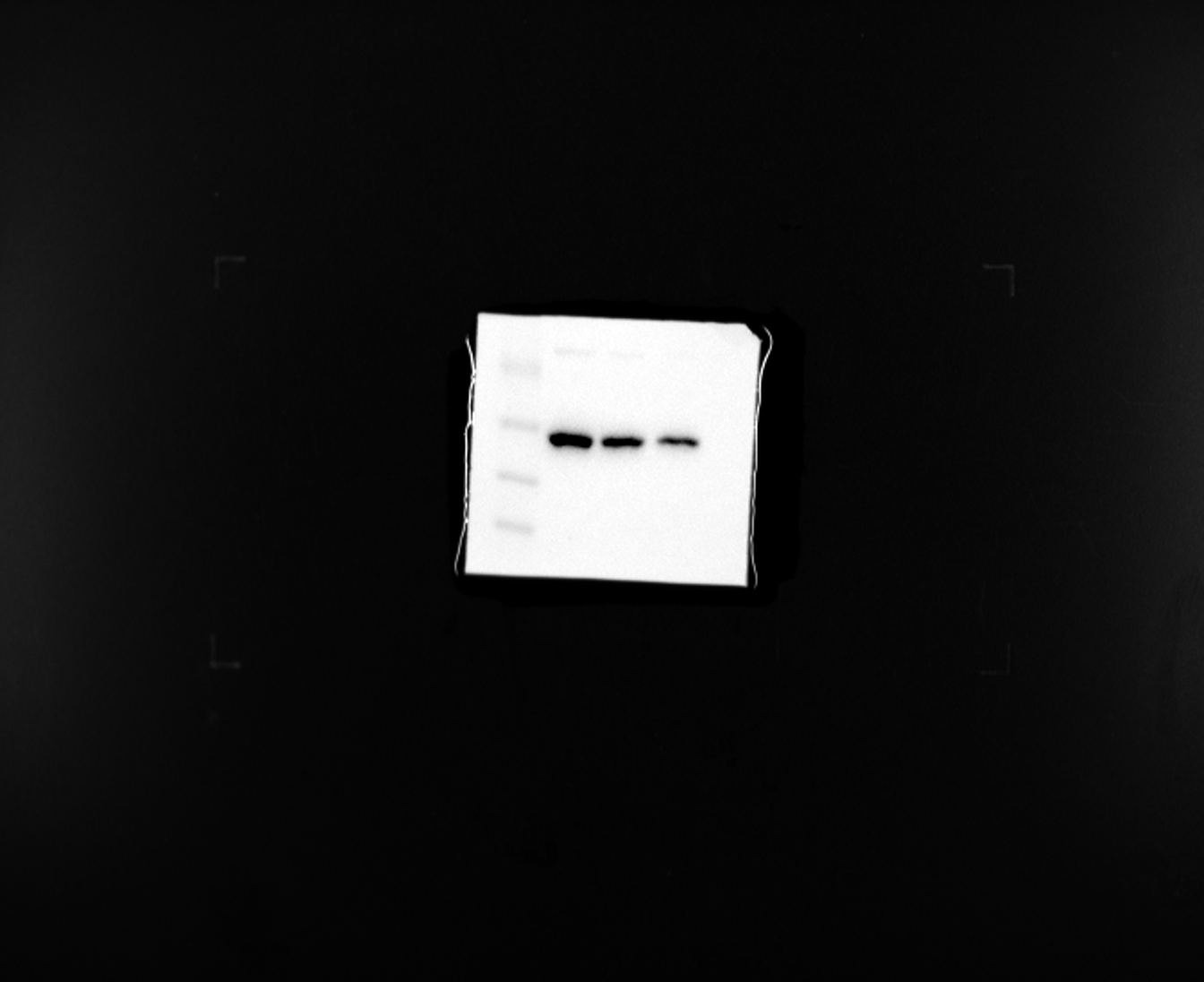

Supplement: Supplementary file 3 — WB Raw data [file 41420_2025_2583_MOESM3_ESM.zip › Figure 6 Panel F/DCAF13/dcaf13_2m.Tif]

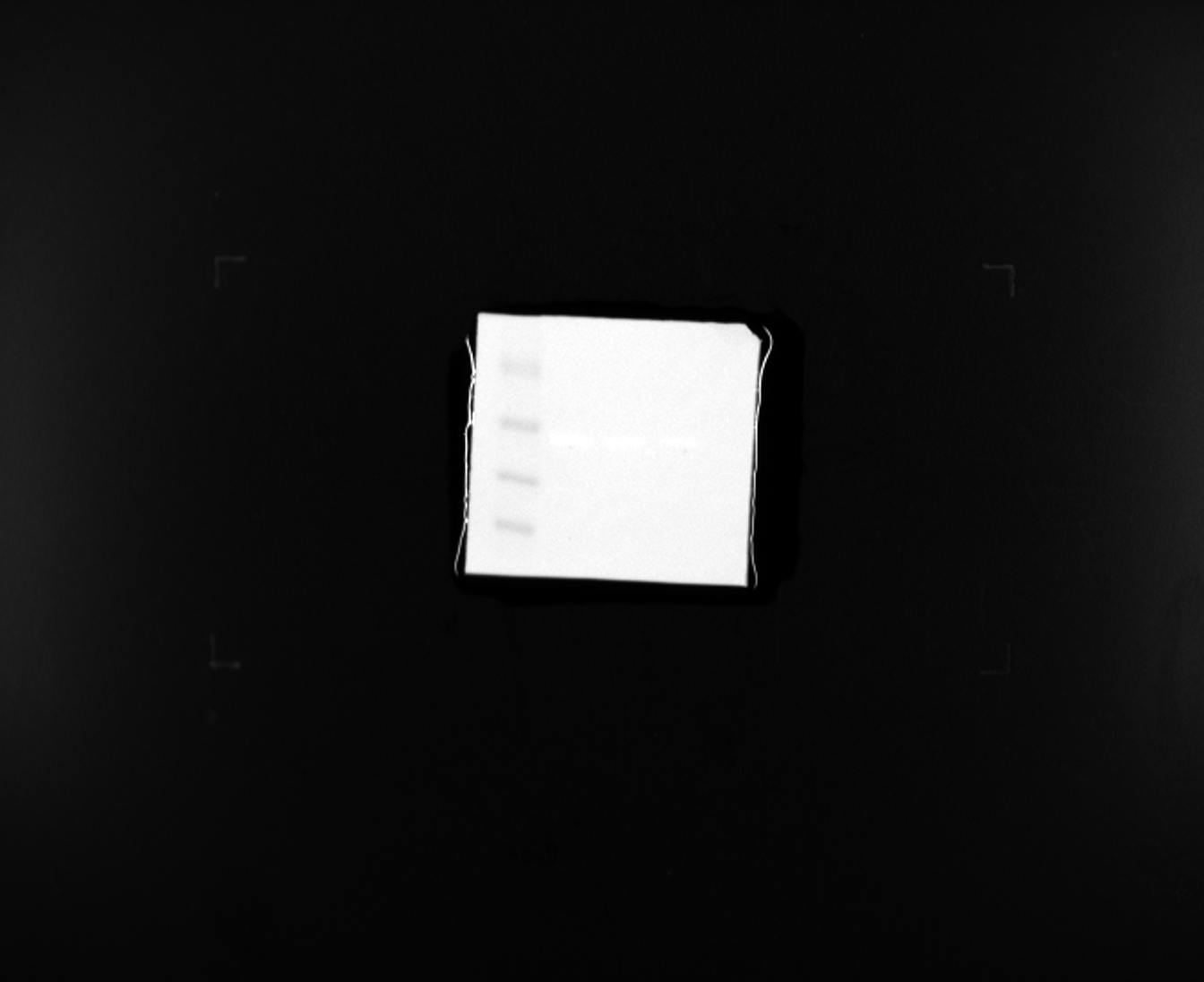

Supplement: Supplementary file 3 — WB Raw data [file 41420_2025_2583_MOESM3_ESM.zip › Figure 6 Panel F/DCAF13/dcaf13_m.Tif]

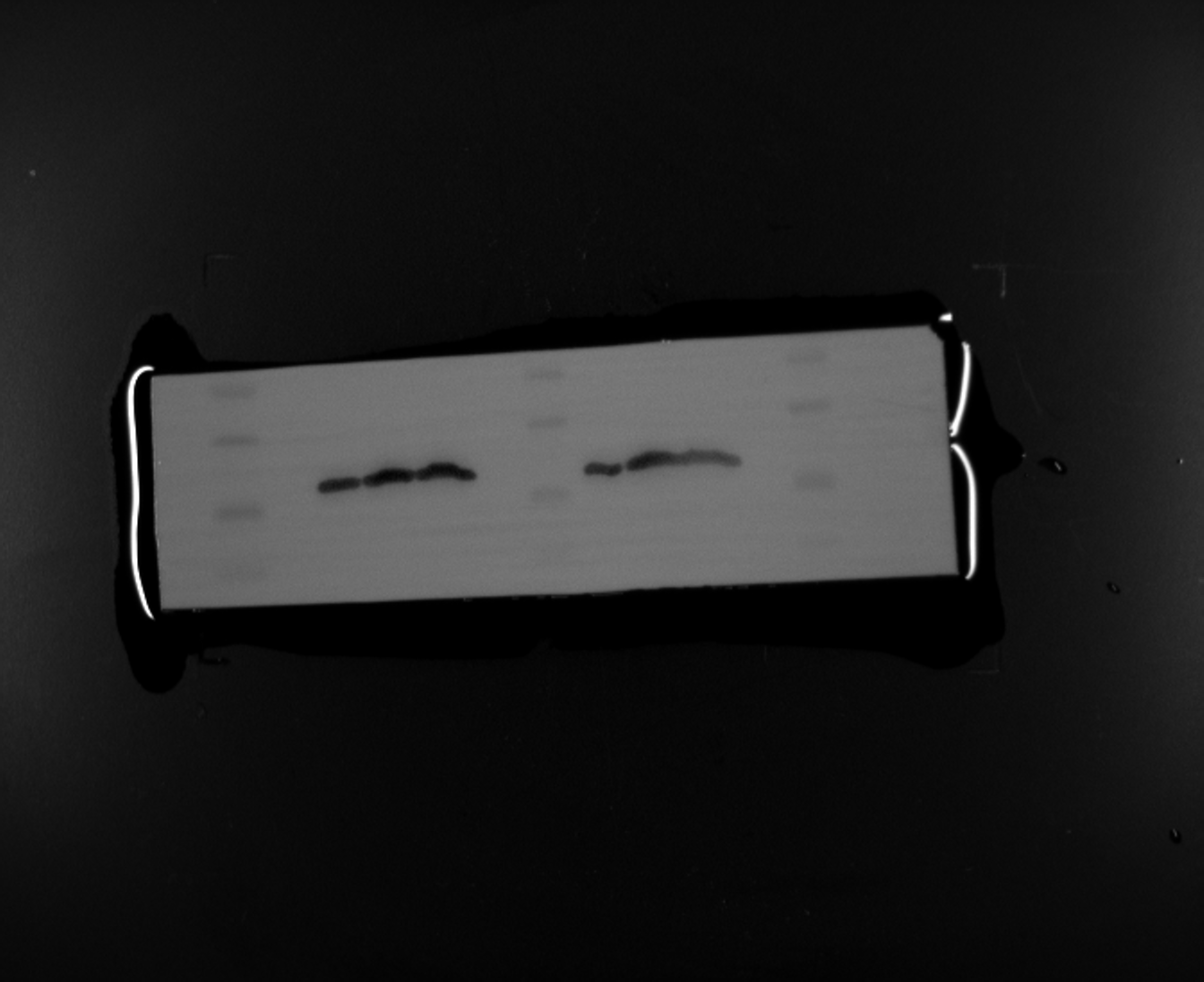

Supplement: Supplementary file 3 — WB Raw data [file 41420_2025_2583_MOESM3_ESM.zip › Figure 6 Panel F/H3K9me3/1+m.Tif]

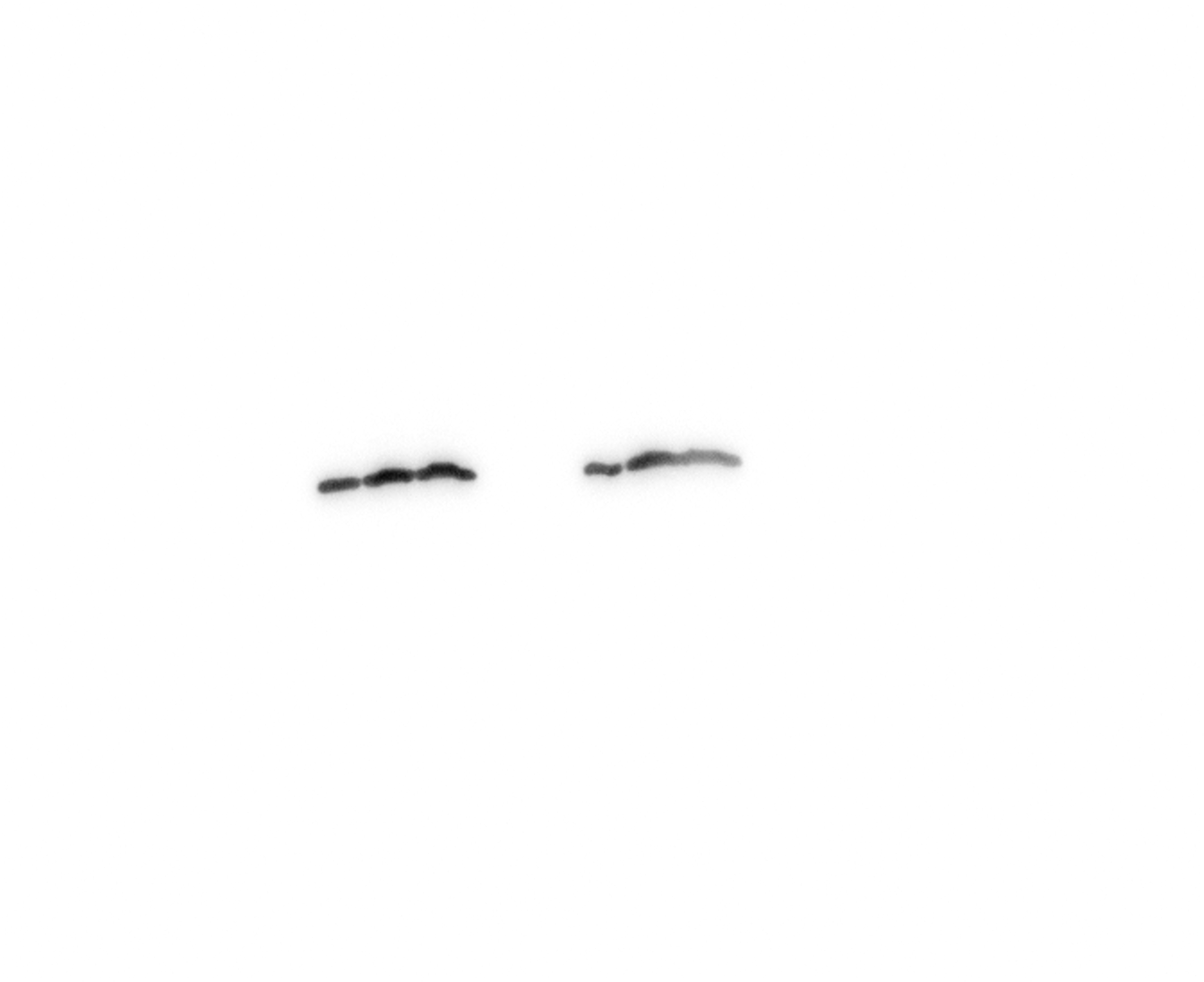

Supplement: Supplementary file 3 — WB Raw data [file 41420_2025_2583_MOESM3_ESM.zip › Figure 6 Panel F/H3K9me3/1.Tif]

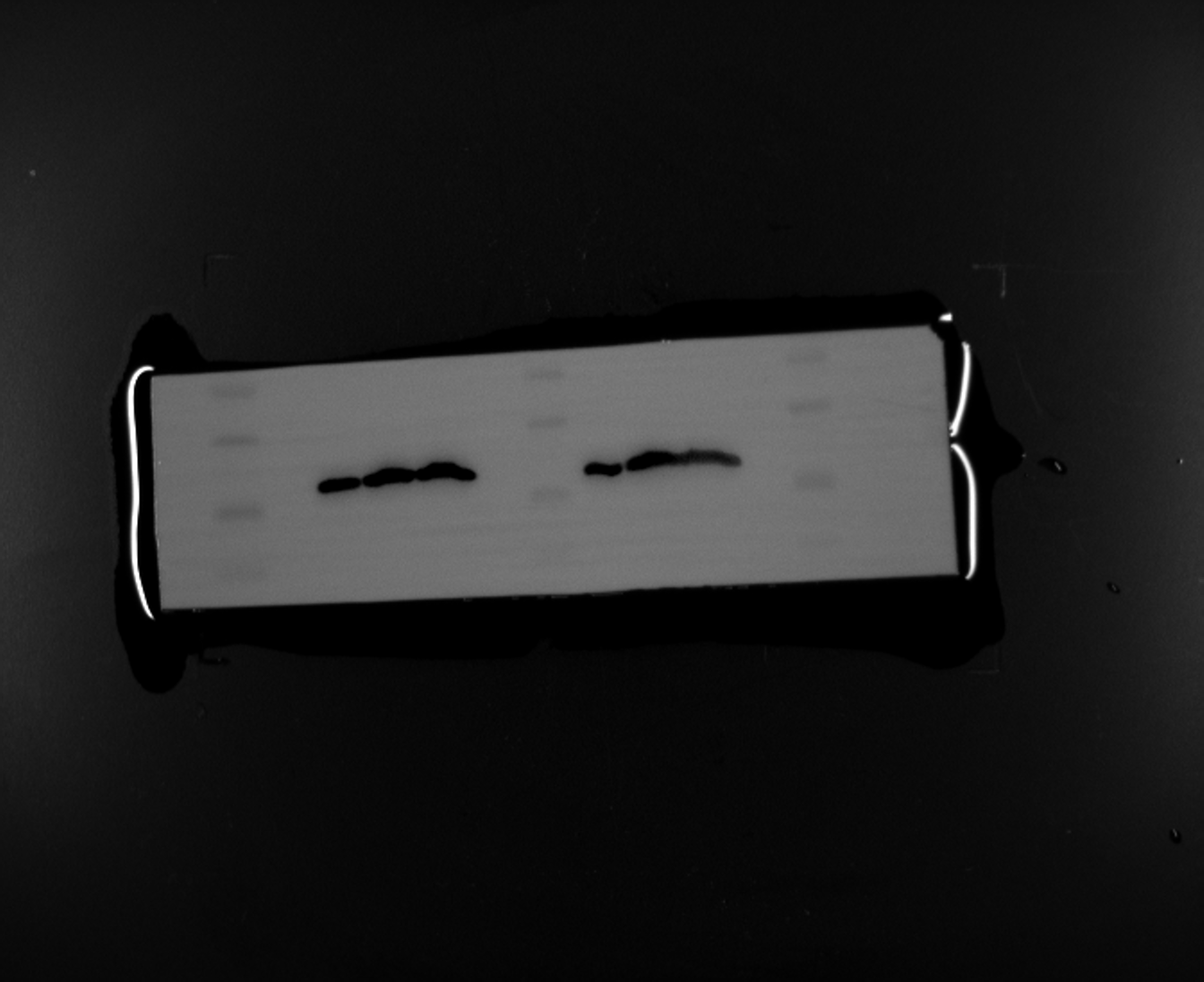

Supplement: Supplementary file 3 — WB Raw data [file 41420_2025_2583_MOESM3_ESM.zip › Figure 6 Panel F/H3K9me3/2+m.Tif]

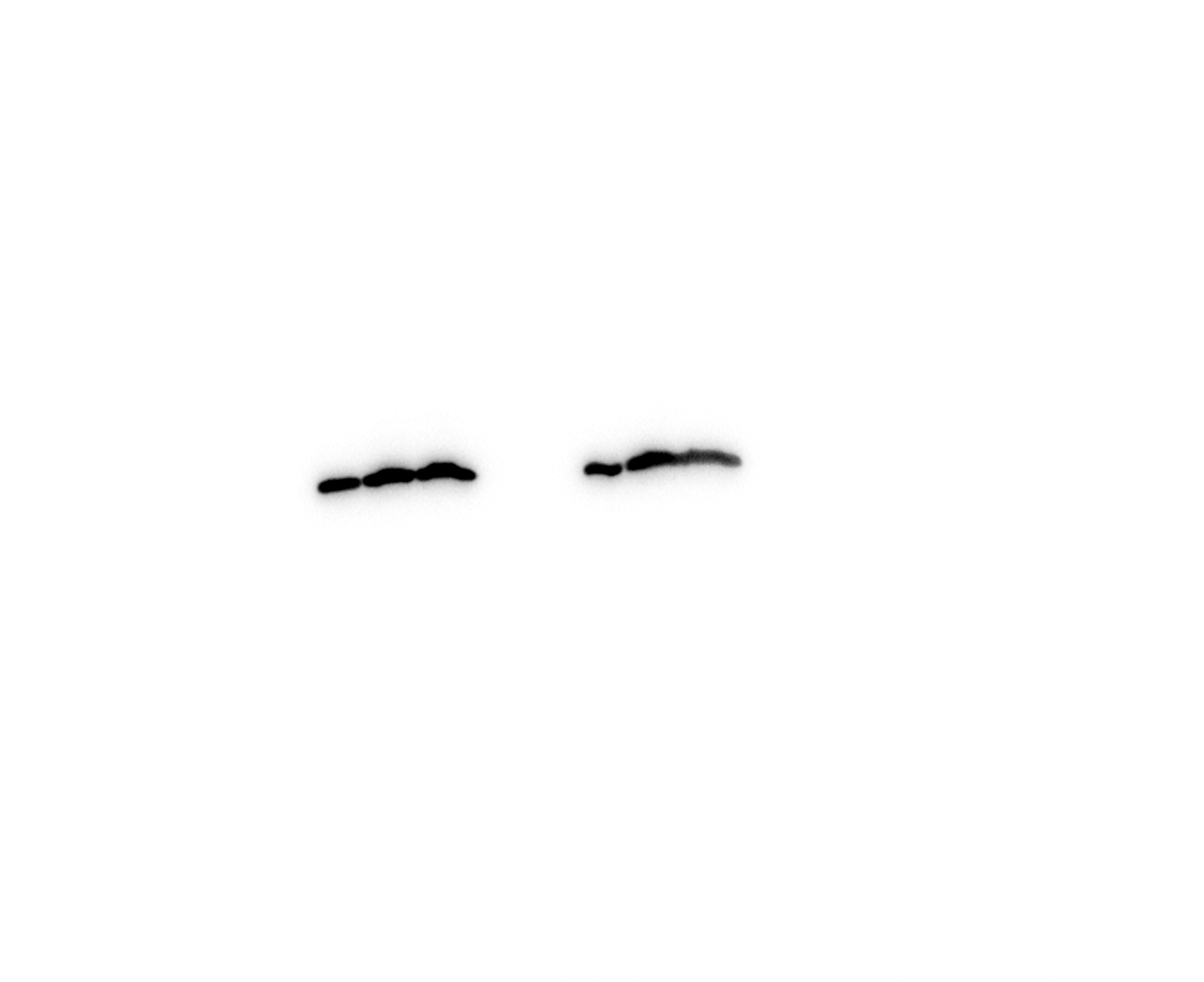

Supplement: Supplementary file 3 — WB Raw data [file 41420_2025_2583_MOESM3_ESM.zip › Figure 6 Panel F/H3K9me3/2.Tif]

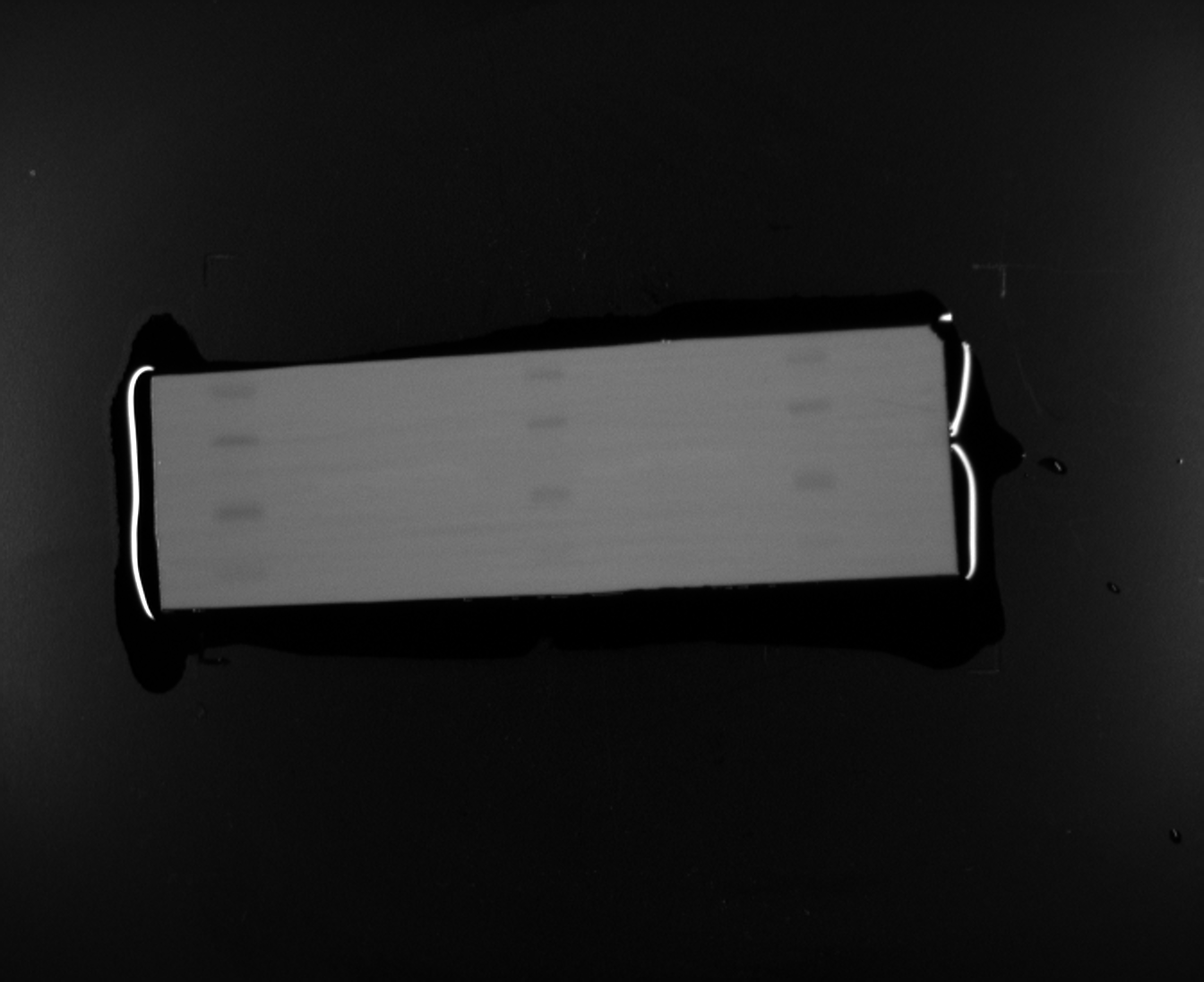

Supplement: Supplementary file 3 — WB Raw data [file 41420_2025_2583_MOESM3_ESM.zip › Figure 6 Panel F/H3K9me3/m.Tif]
