# Supplementary figures and images for: DCAF13 is essential for mouse uterine function and fertility (part 1 of 2)
Source: Cell Death Discov. 2025 Aug 1;11:359. doi: 10.1038/s41420-025-02583-w (PMC12316921; doi:10.1038/s41420-025-02583-w)

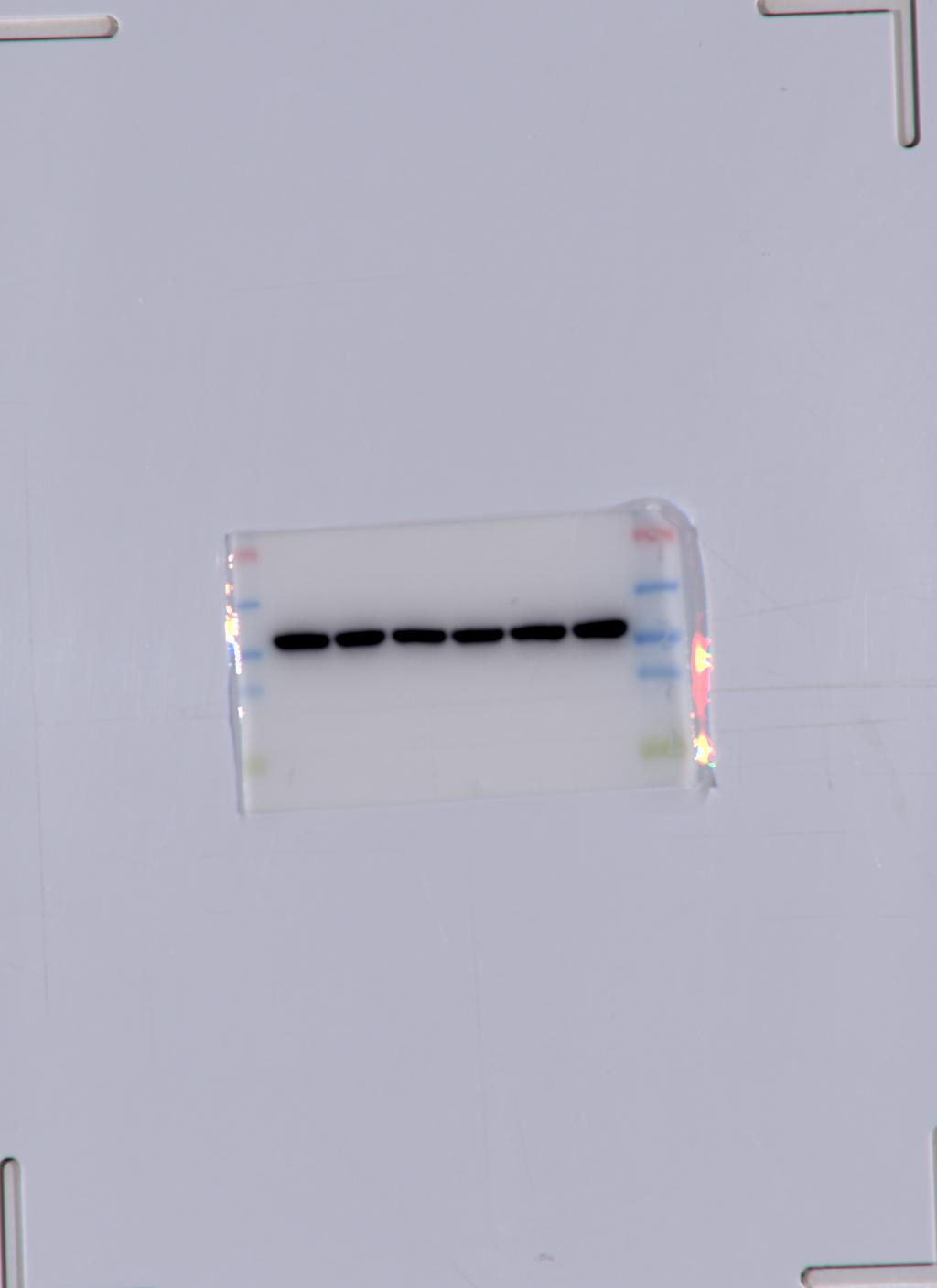

Supplement: Supplementary file 3 — WB Raw data [file 41420_2025_2583_MOESM3_ESM.zip › Figure S3 Panel B/actin 2022.04.14_11.37.10_Ch/actin 2022.04.14_11.37.10_Ch+Marker.jpg]

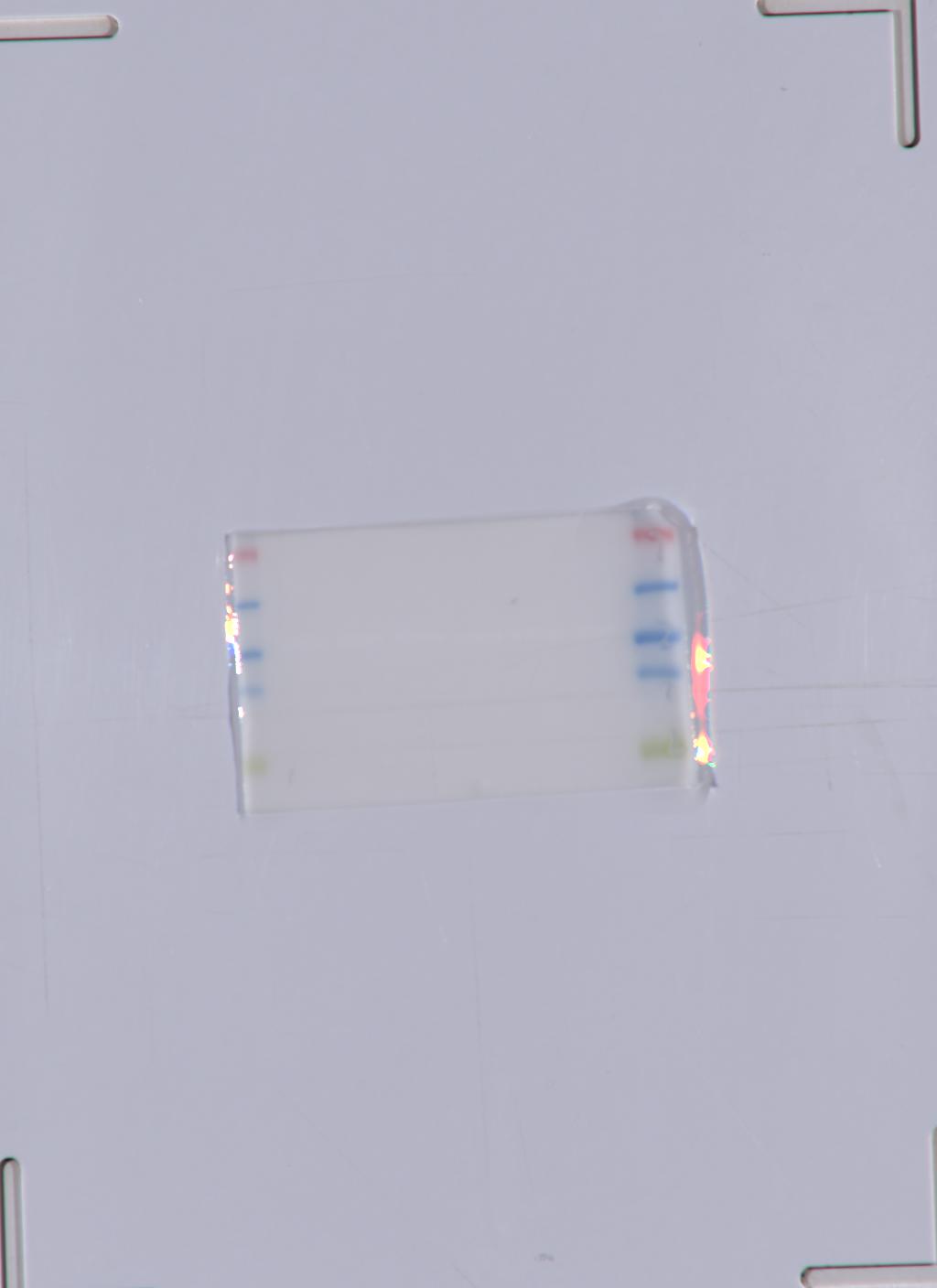

Supplement: Supplementary file 3 — WB Raw data [file 41420_2025_2583_MOESM3_ESM.zip › Figure S3 Panel B/actin 2022.04.14_11.37.10_Ch/actin 2022.04.14_11.37.10_Ch-Marker.jpg]

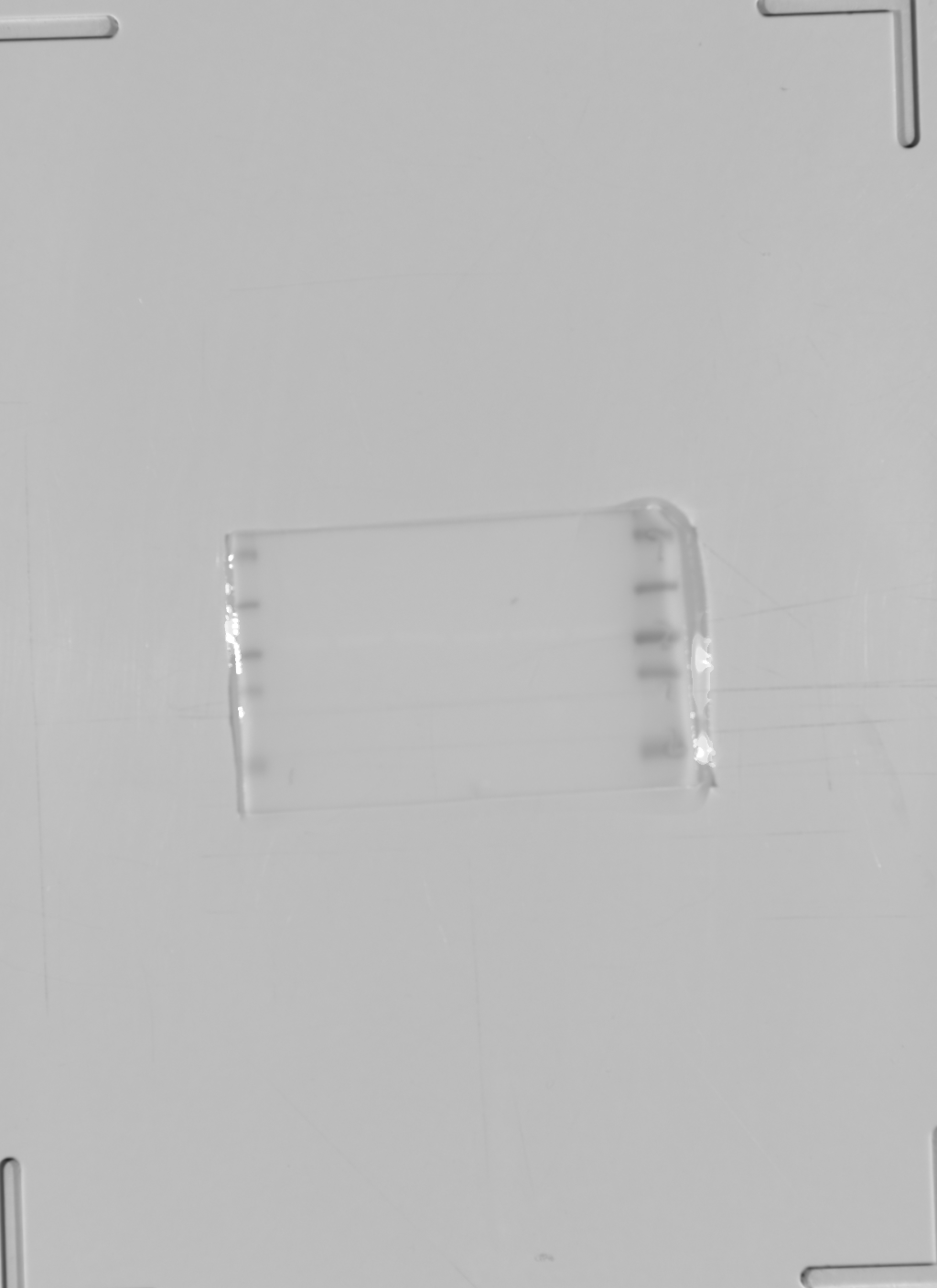

Supplement: Supplementary file 3 — WB Raw data [file 41420_2025_2583_MOESM3_ESM.zip › Figure S3 Panel B/actin 2022.04.14_11.37.10_Ch/actin 2022.04.14_11.37.10_Ch-Marker.tif]

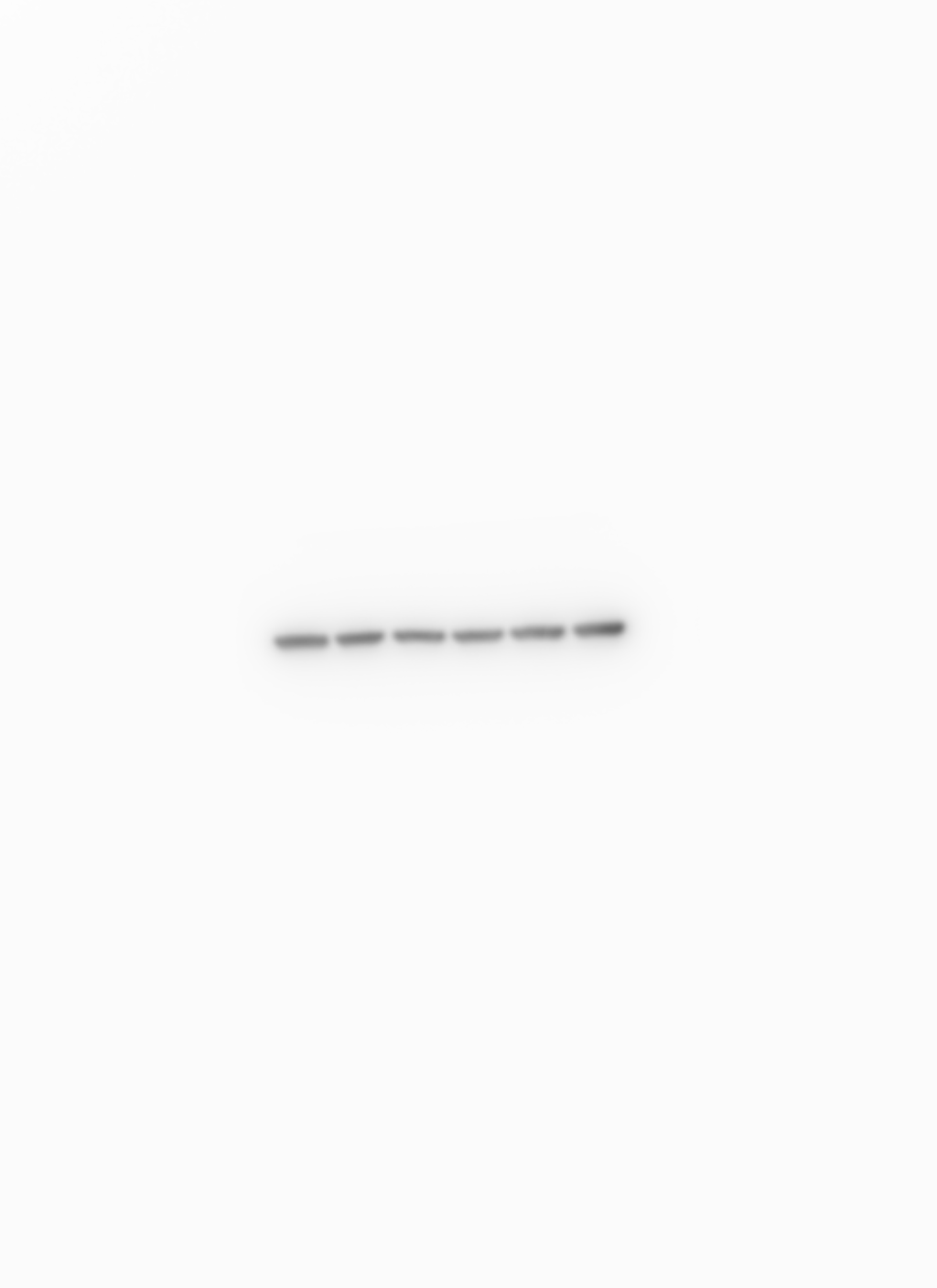

Supplement: Supplementary file 3 — WB Raw data [file 41420_2025_2583_MOESM3_ESM.zip › Figure S3 Panel B/actin 2022.04.14_11.37.10_Ch/actin 2022.04.14_11.37.10_Ch.tif]

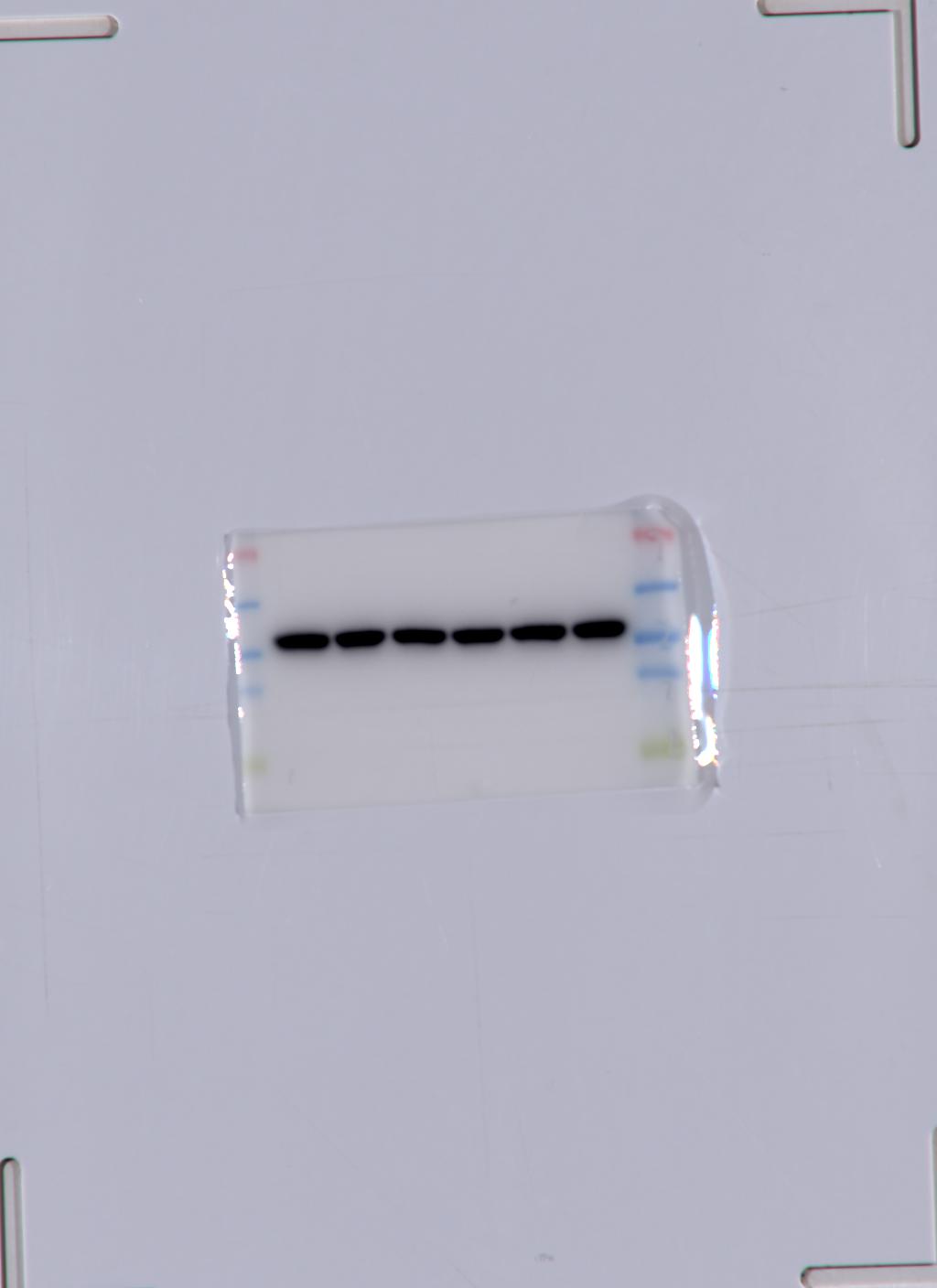

Supplement: Supplementary file 3 — WB Raw data [file 41420_2025_2583_MOESM3_ESM.zip › Figure S3 Panel B/actin 2022.04.14_11.38.17_Ch/actin 2022.04.14_11.38.17_Ch+Marker.jpg]

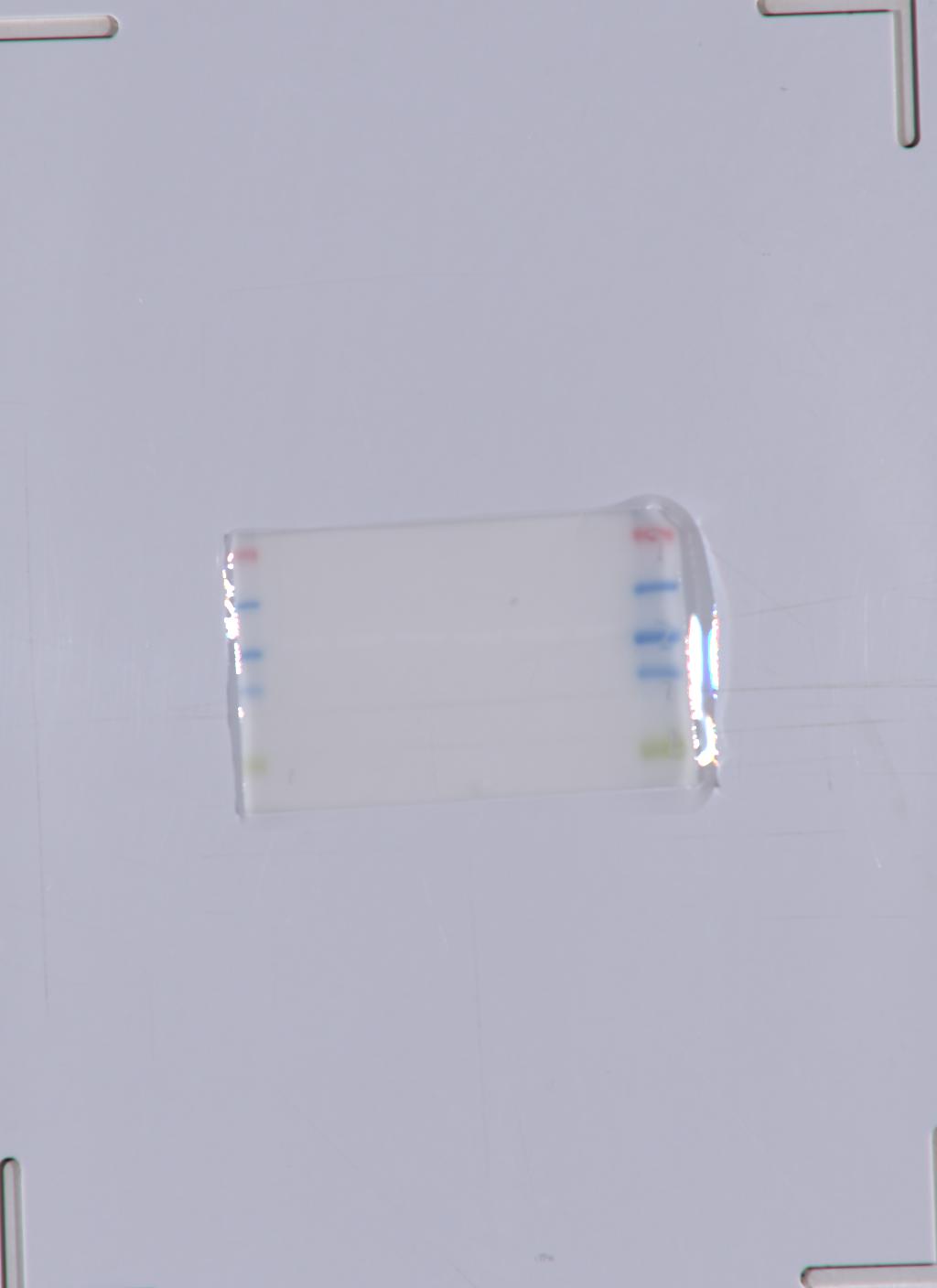

Supplement: Supplementary file 3 — WB Raw data [file 41420_2025_2583_MOESM3_ESM.zip › Figure S3 Panel B/actin 2022.04.14_11.38.17_Ch/actin 2022.04.14_11.38.17_Ch-Marker.jpg]

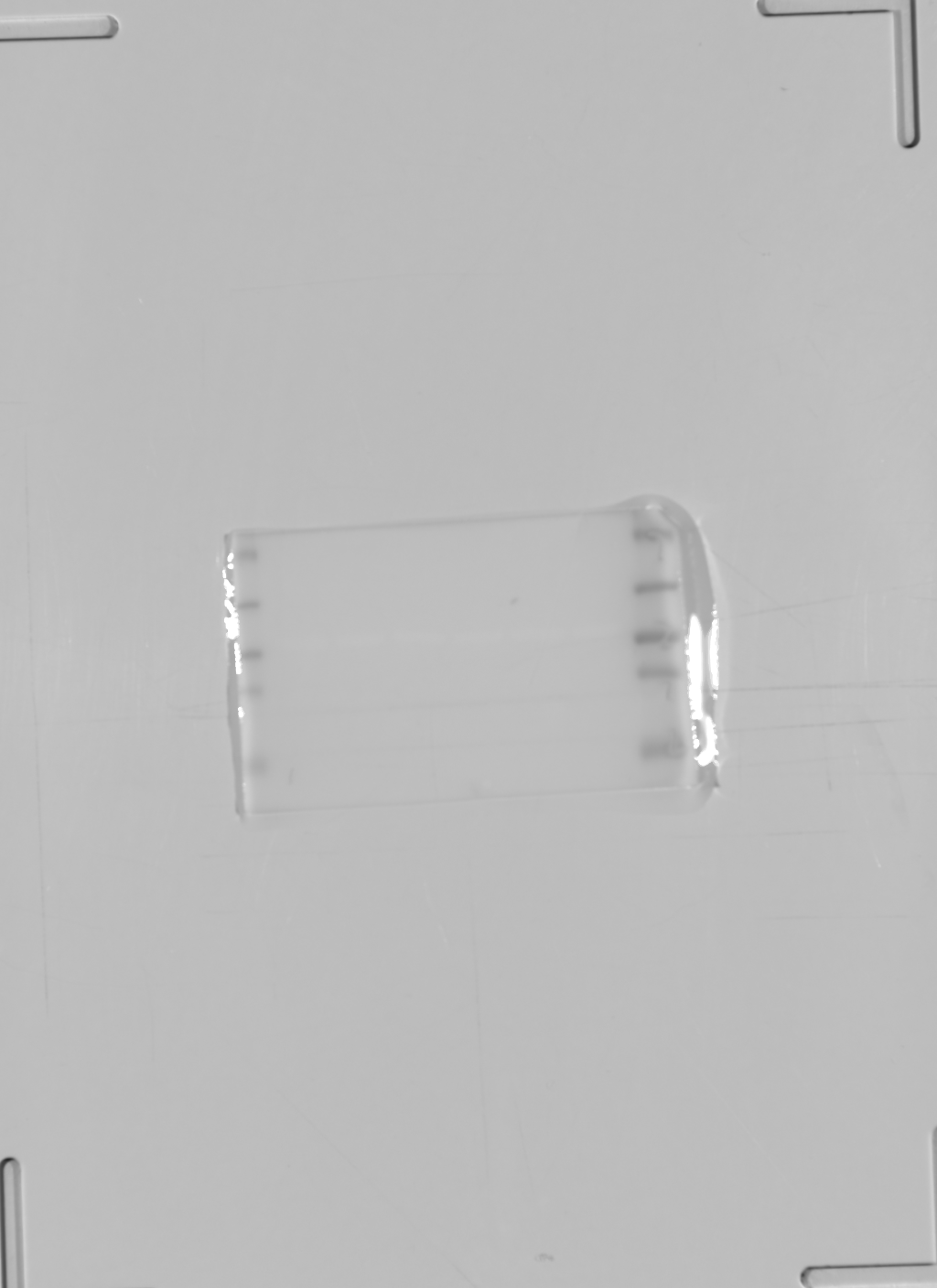

Supplement: Supplementary file 3 — WB Raw data [file 41420_2025_2583_MOESM3_ESM.zip › Figure S3 Panel B/actin 2022.04.14_11.38.17_Ch/actin 2022.04.14_11.38.17_Ch-Marker.tif]

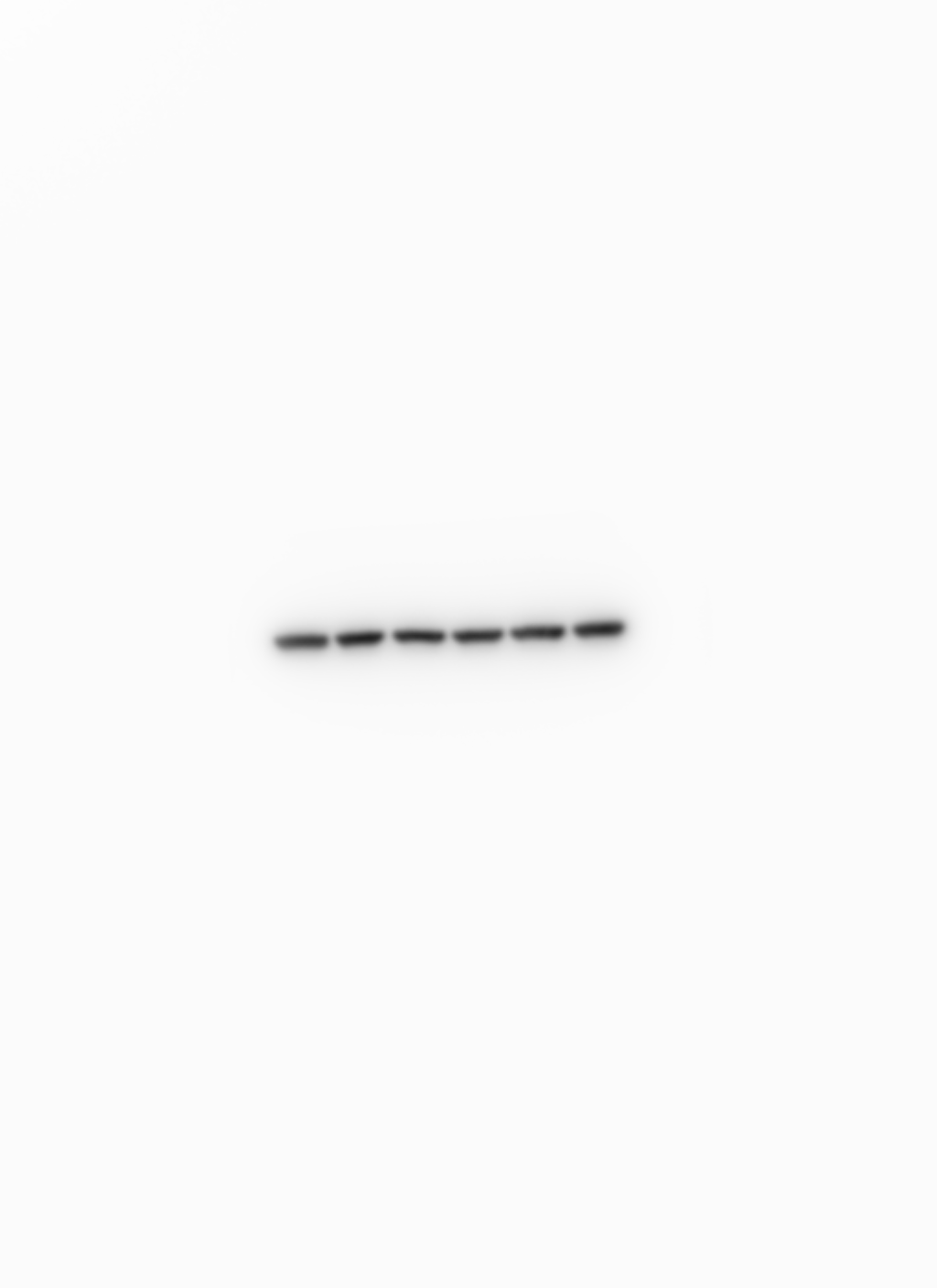

Supplement: Supplementary file 3 — WB Raw data [file 41420_2025_2583_MOESM3_ESM.zip › Figure S3 Panel B/actin 2022.04.14_11.38.17_Ch/actin 2022.04.14_11.38.17_Ch.tif]

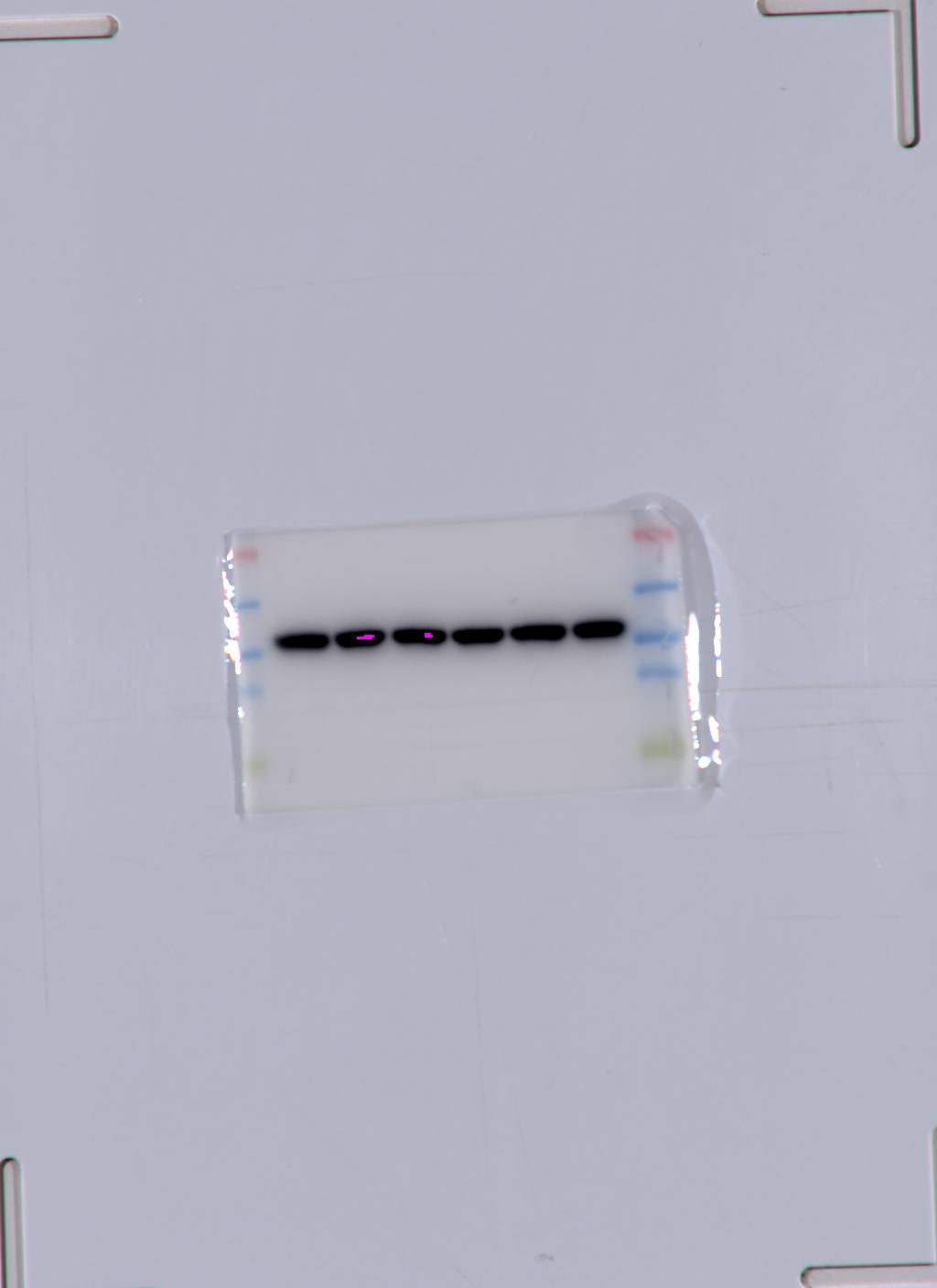

Supplement: Supplementary file 3 — WB Raw data [file 41420_2025_2583_MOESM3_ESM.zip › Figure S3 Panel B/actin 2022.04.14_11.39.23_Ch/actin 2022.04.14_11.39.23_Ch+Marker.jpg]

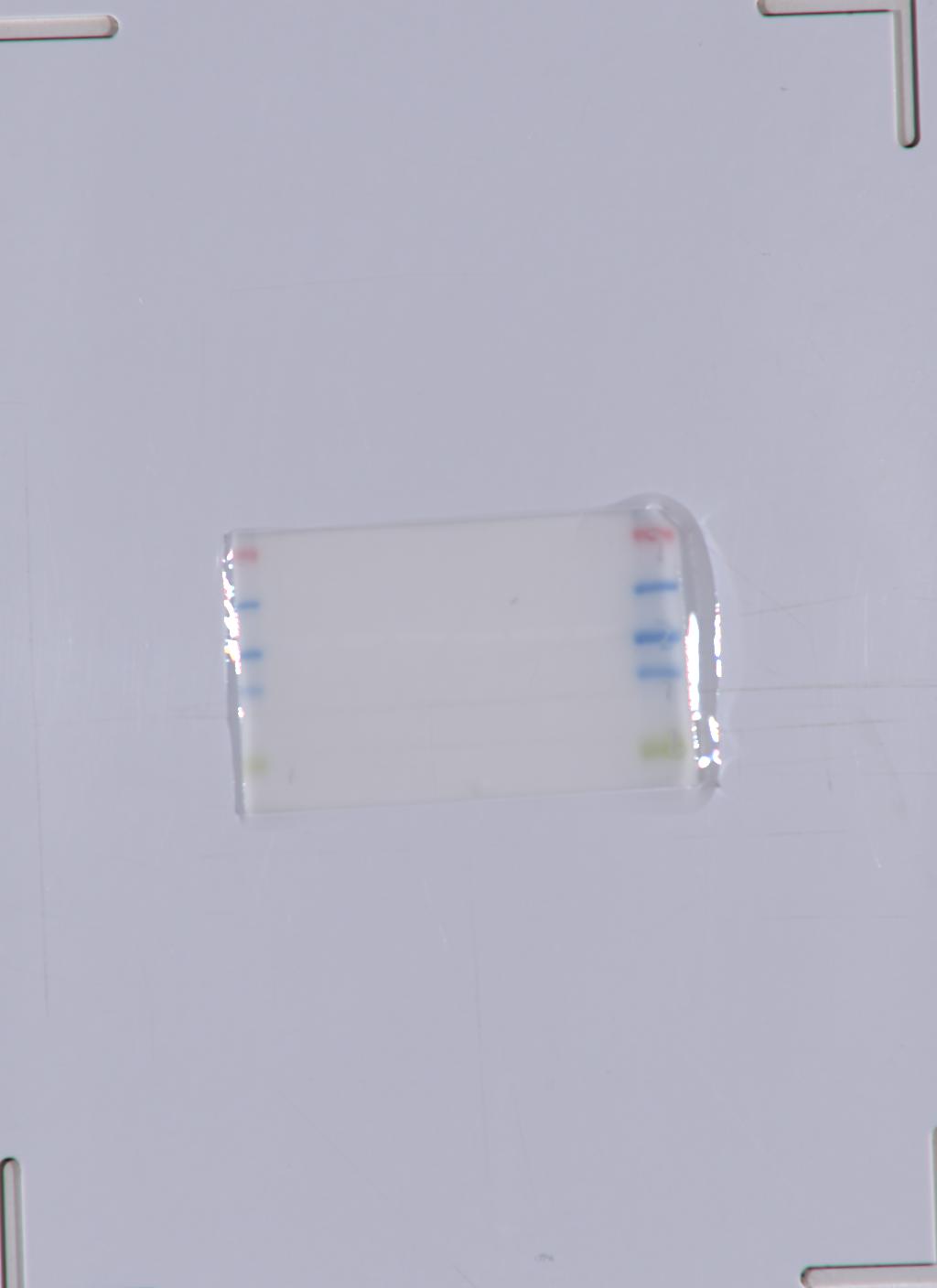

Supplement: Supplementary file 3 — WB Raw data [file 41420_2025_2583_MOESM3_ESM.zip › Figure S3 Panel B/actin 2022.04.14_11.39.23_Ch/actin 2022.04.14_11.39.23_Ch-Marker.jpg]

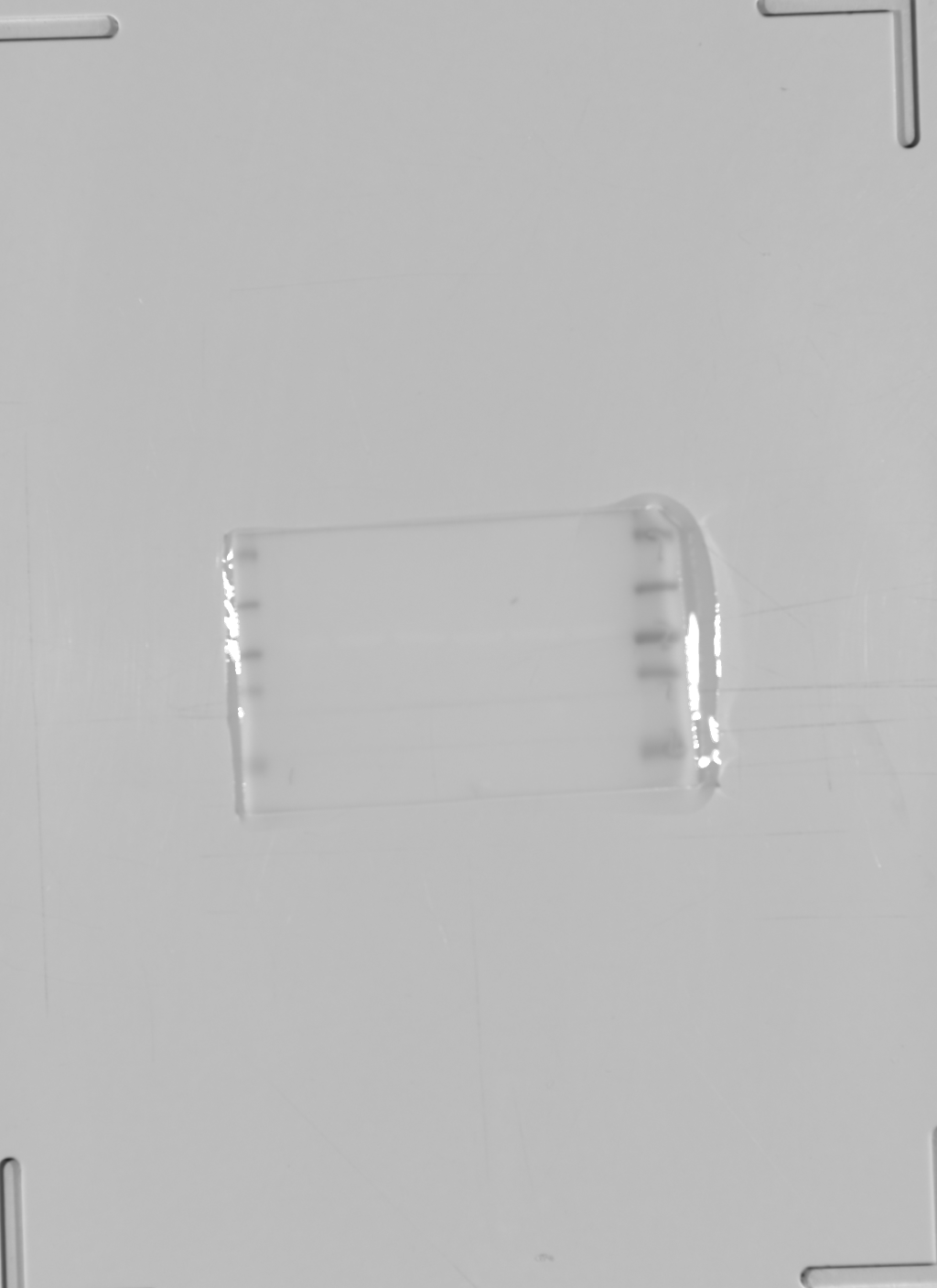

Supplement: Supplementary file 3 — WB Raw data [file 41420_2025_2583_MOESM3_ESM.zip › Figure S3 Panel B/actin 2022.04.14_11.39.23_Ch/actin 2022.04.14_11.39.23_Ch-Marker.tif]

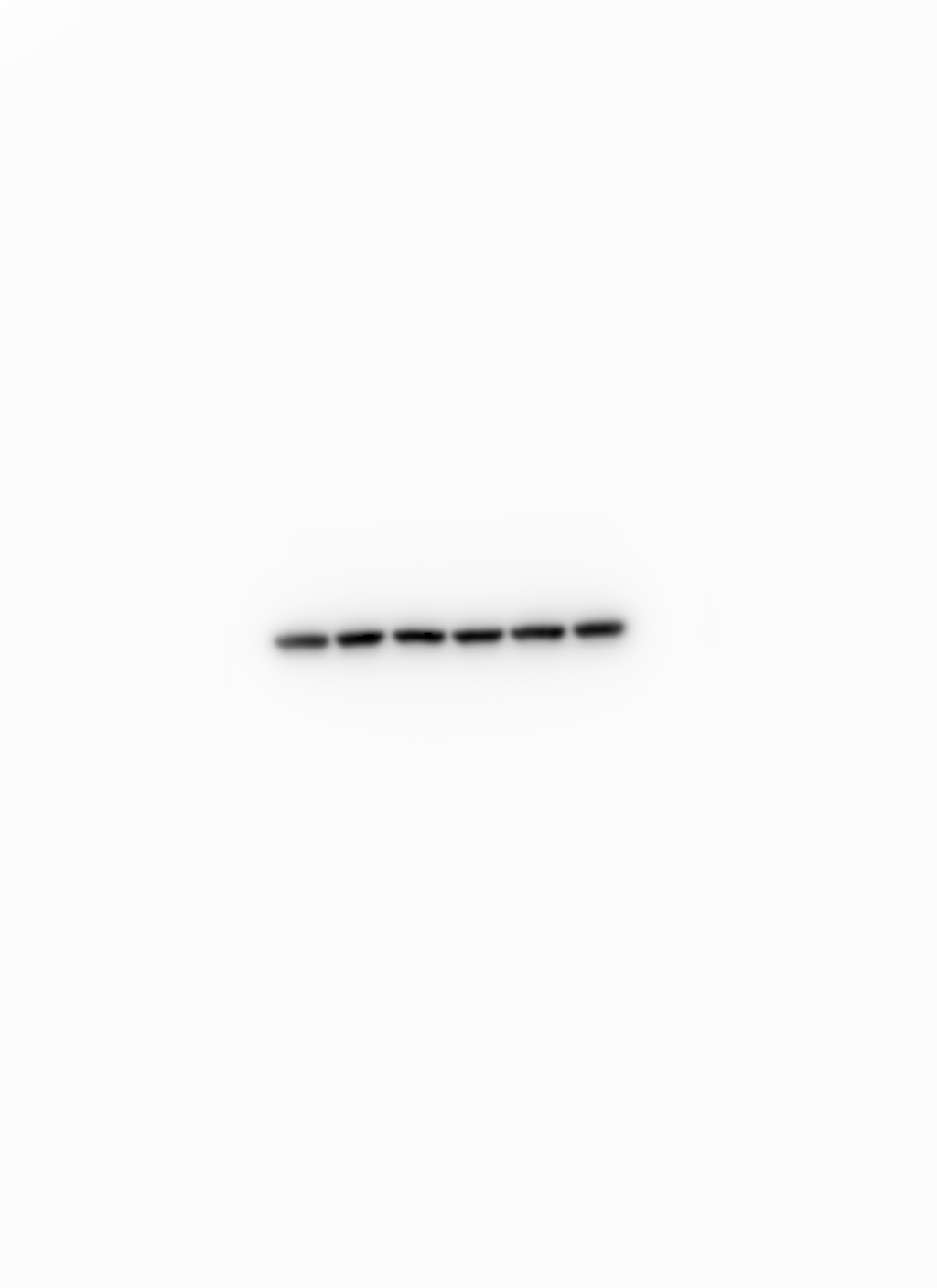

Supplement: Supplementary file 3 — WB Raw data [file 41420_2025_2583_MOESM3_ESM.zip › Figure S3 Panel B/actin 2022.04.14_11.39.23_Ch/actin 2022.04.14_11.39.23_Ch.tif]

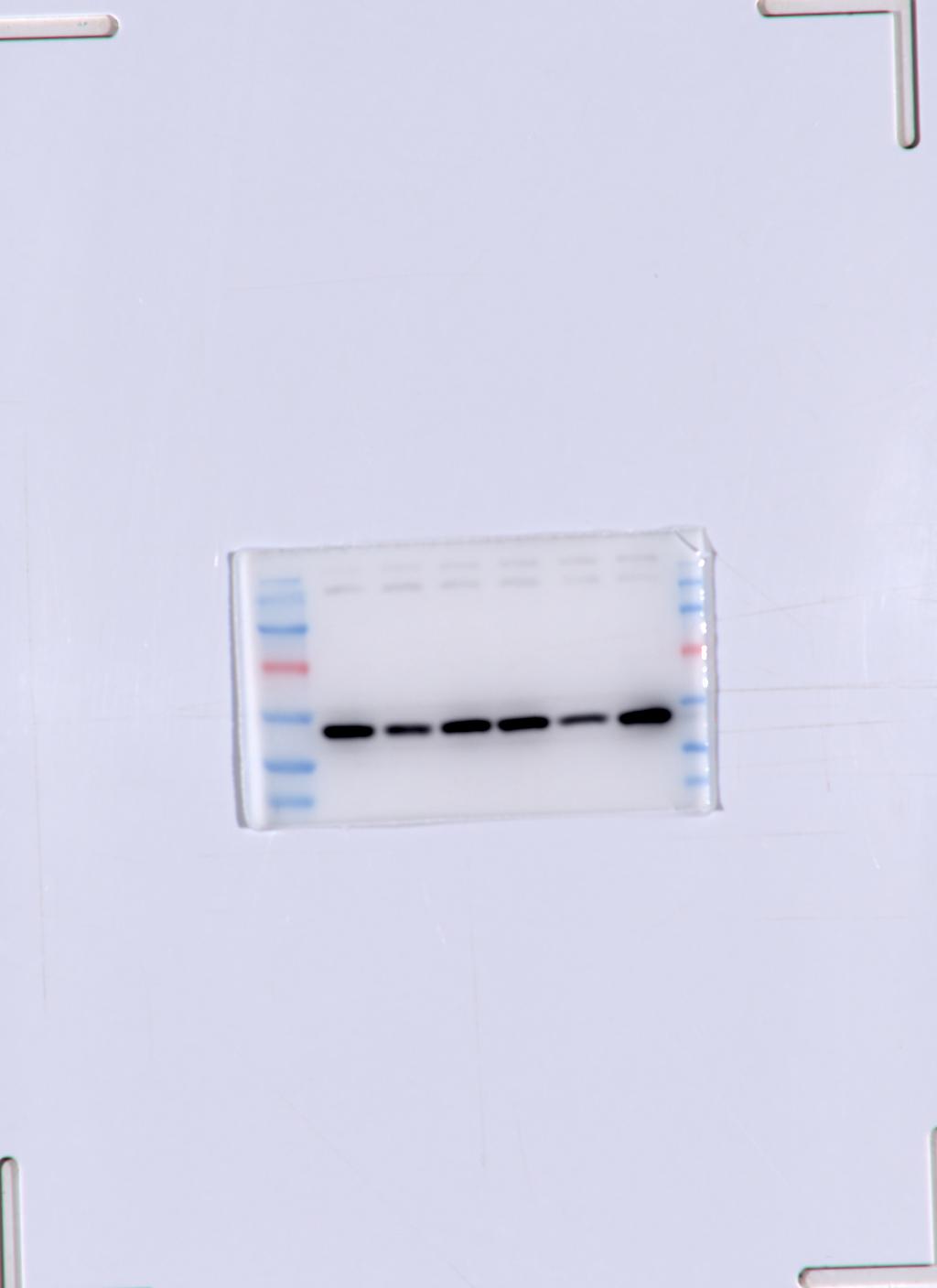

Supplement: Supplementary file 3 — WB Raw data [file 41420_2025_2583_MOESM3_ESM.zip › Figure S3 Panel B/dcaf13 2022.04.14_11.41.02_Ch/dcaf13 2022.04.14_11.41.02_Ch+Marker.jpg]

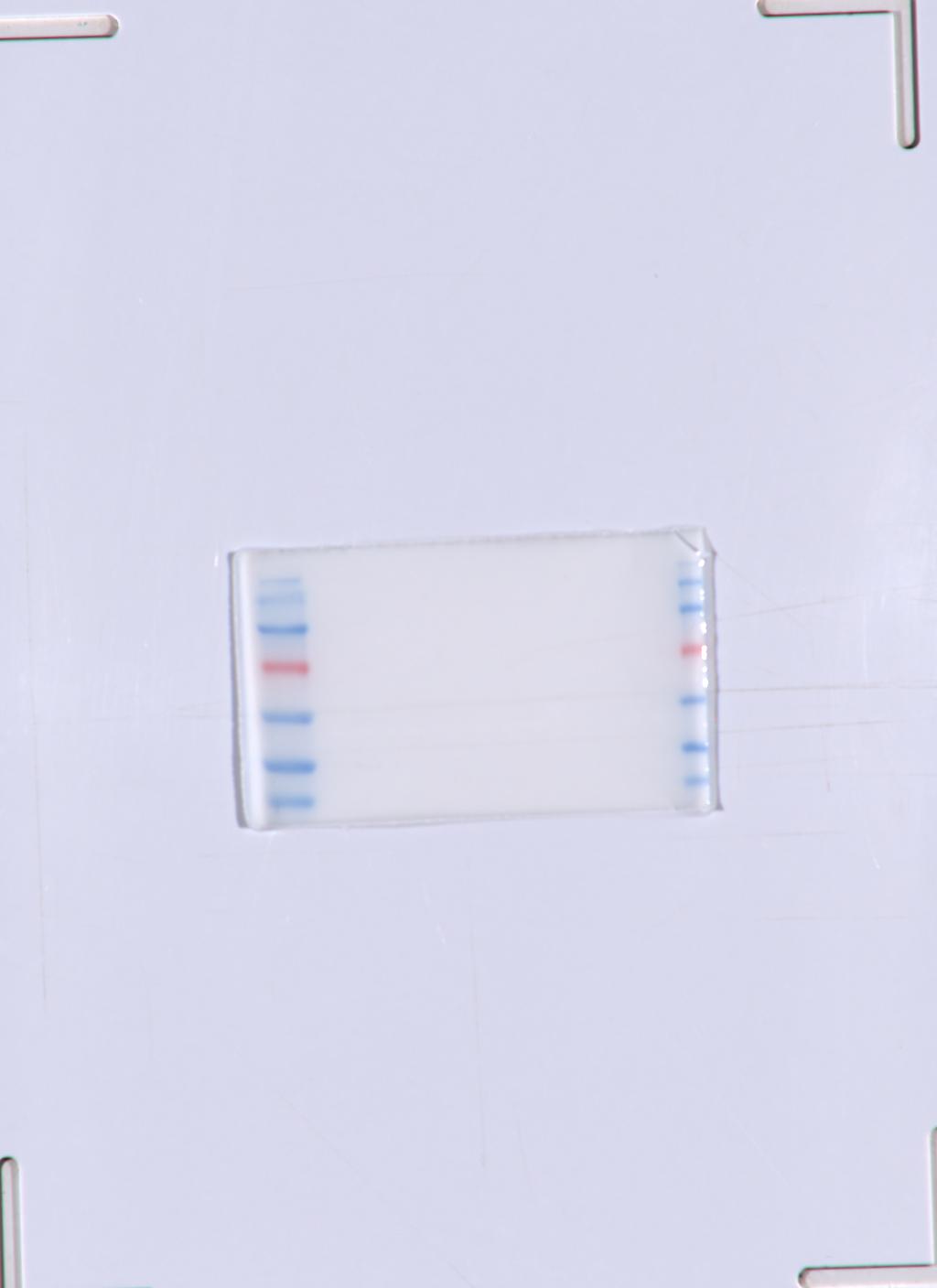

Supplement: Supplementary file 3 — WB Raw data [file 41420_2025_2583_MOESM3_ESM.zip › Figure S3 Panel B/dcaf13 2022.04.14_11.41.02_Ch/dcaf13 2022.04.14_11.41.02_Ch-Marker.jpg]

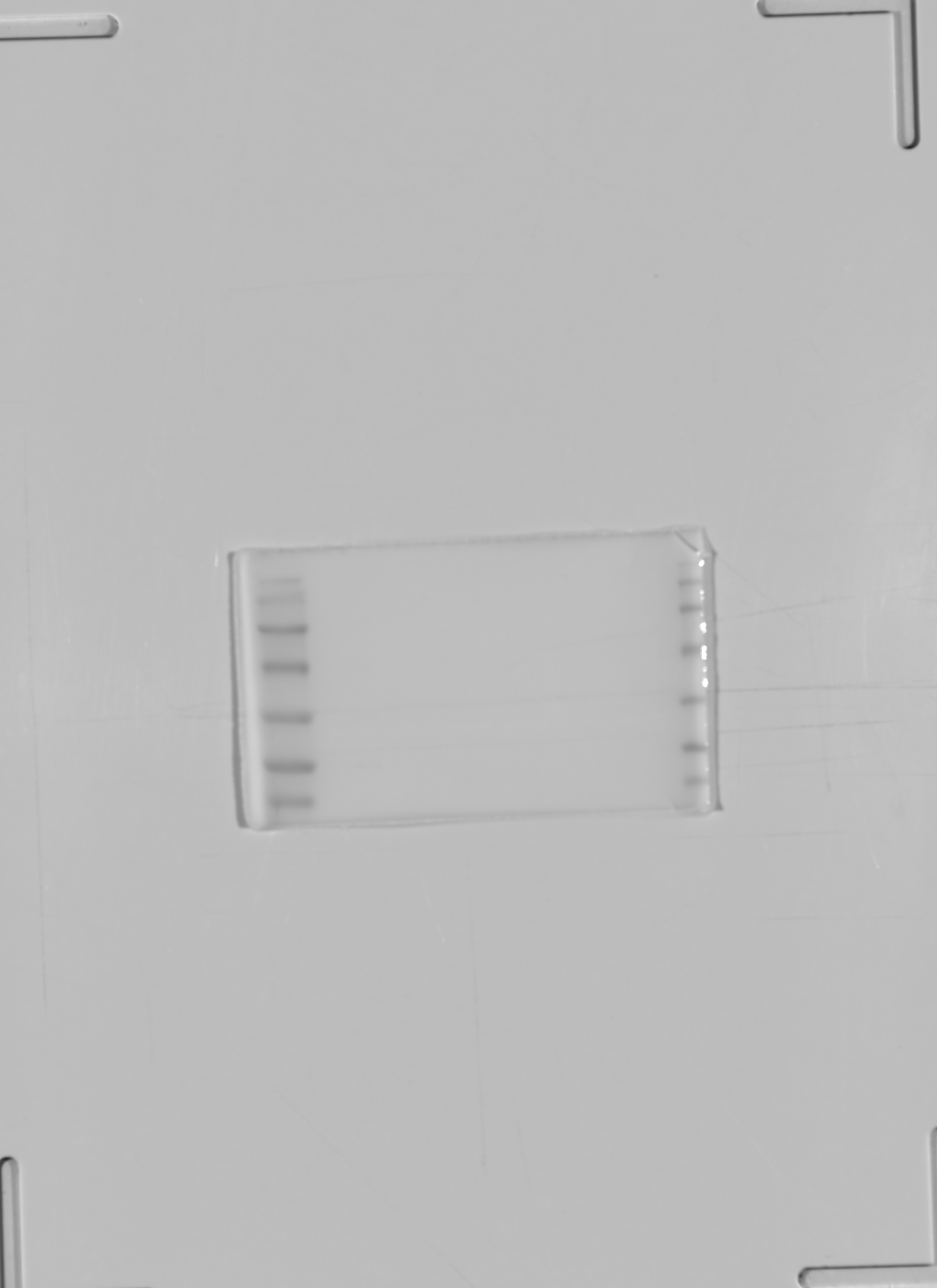

Supplement: Supplementary file 3 — WB Raw data [file 41420_2025_2583_MOESM3_ESM.zip › Figure S3 Panel B/dcaf13 2022.04.14_11.41.02_Ch/dcaf13 2022.04.14_11.41.02_Ch-Marker.tif]

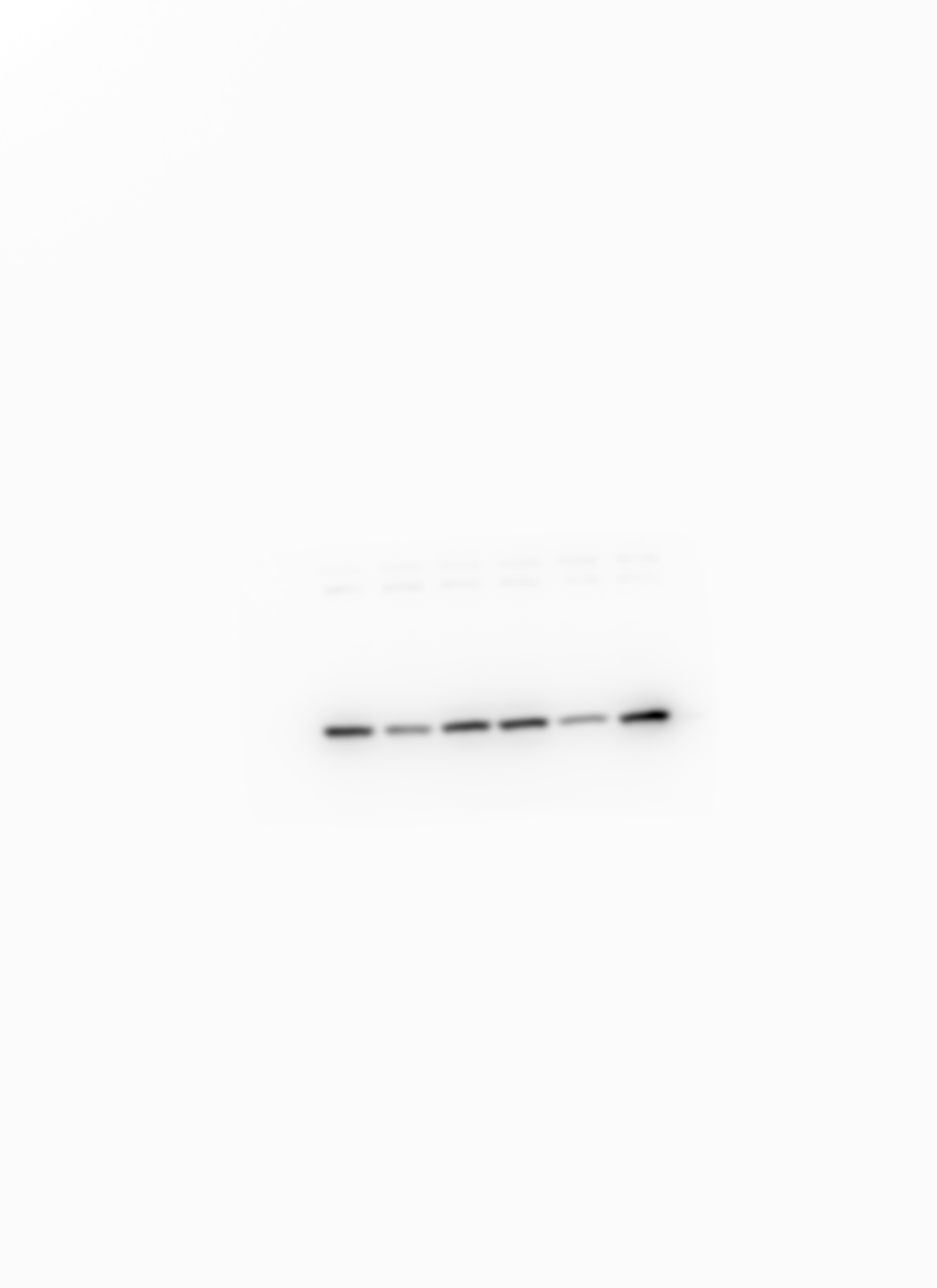

Supplement: Supplementary file 3 — WB Raw data [file 41420_2025_2583_MOESM3_ESM.zip › Figure S3 Panel B/dcaf13 2022.04.14_11.41.02_Ch/dcaf13 2022.04.14_11.41.02_Ch.tif]

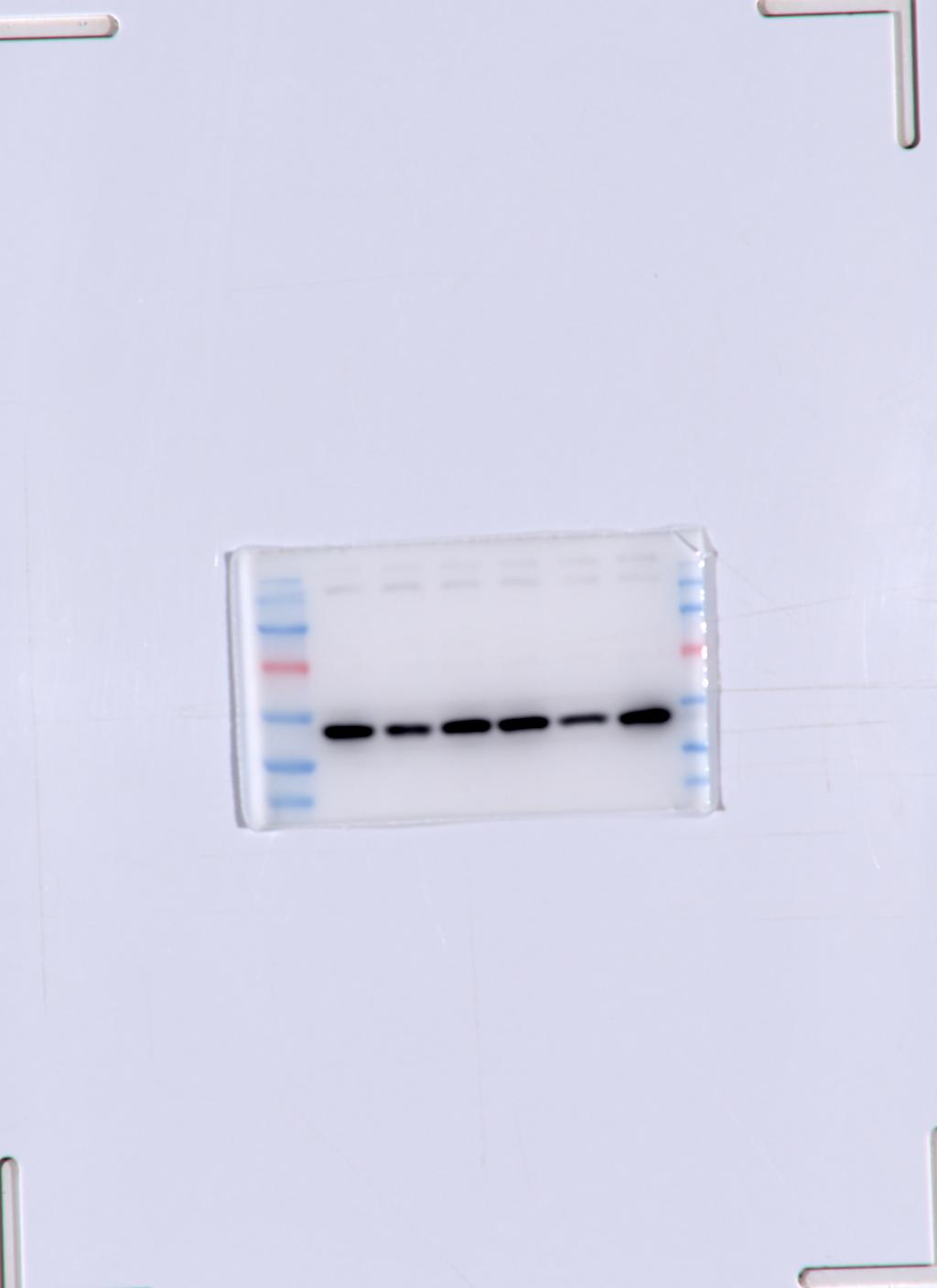

Supplement: Supplementary file 3 — WB Raw data [file 41420_2025_2583_MOESM3_ESM.zip › Figure S3 Panel B/dcaf13 2022.04.14_11.42.08_Ch/dcaf13 2022.04.14_11.42.08_Ch+Marker.jpg]

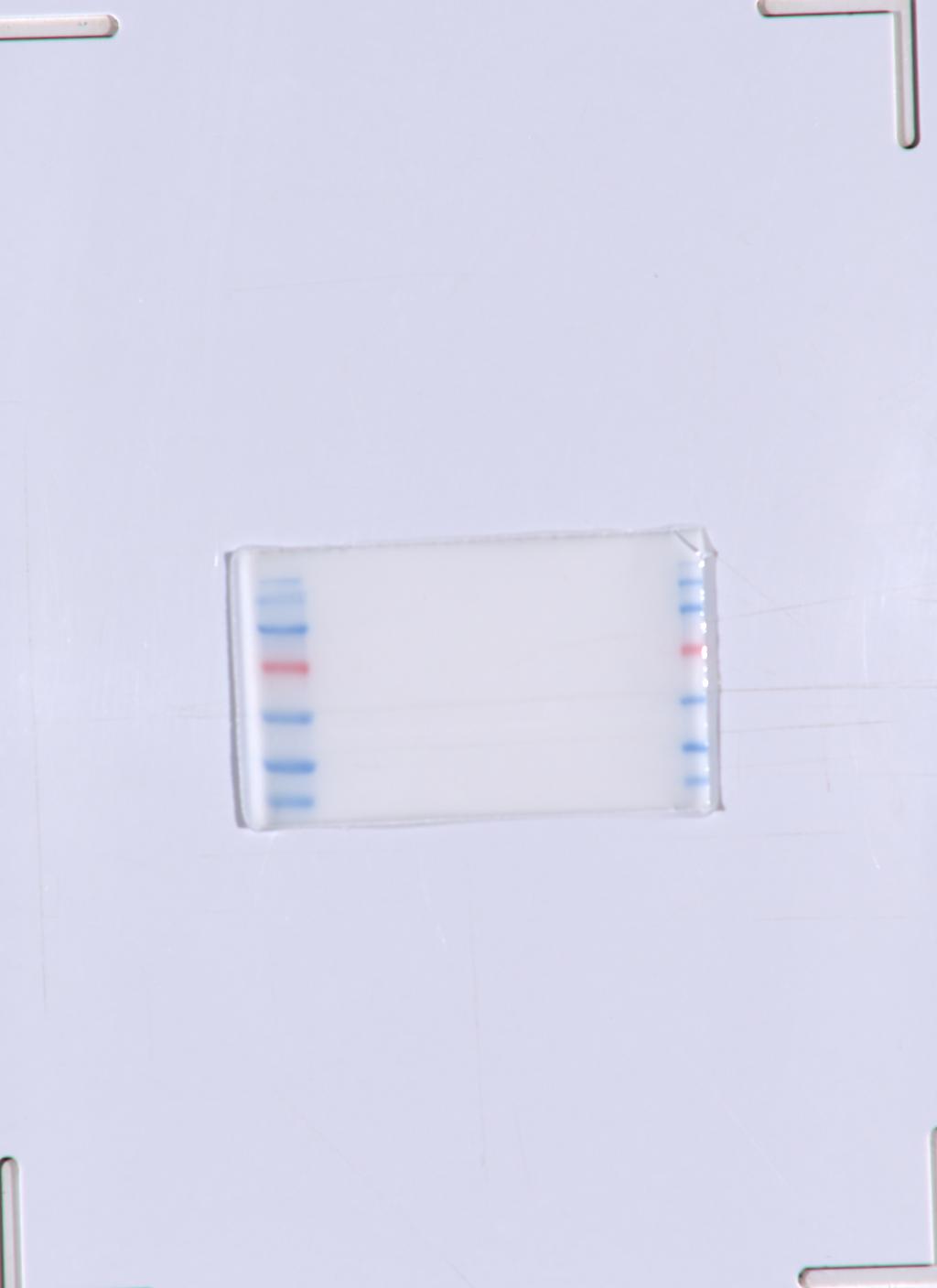

Supplement: Supplementary file 3 — WB Raw data [file 41420_2025_2583_MOESM3_ESM.zip › Figure S3 Panel B/dcaf13 2022.04.14_11.42.08_Ch/dcaf13 2022.04.14_11.42.08_Ch-Marker.jpg]

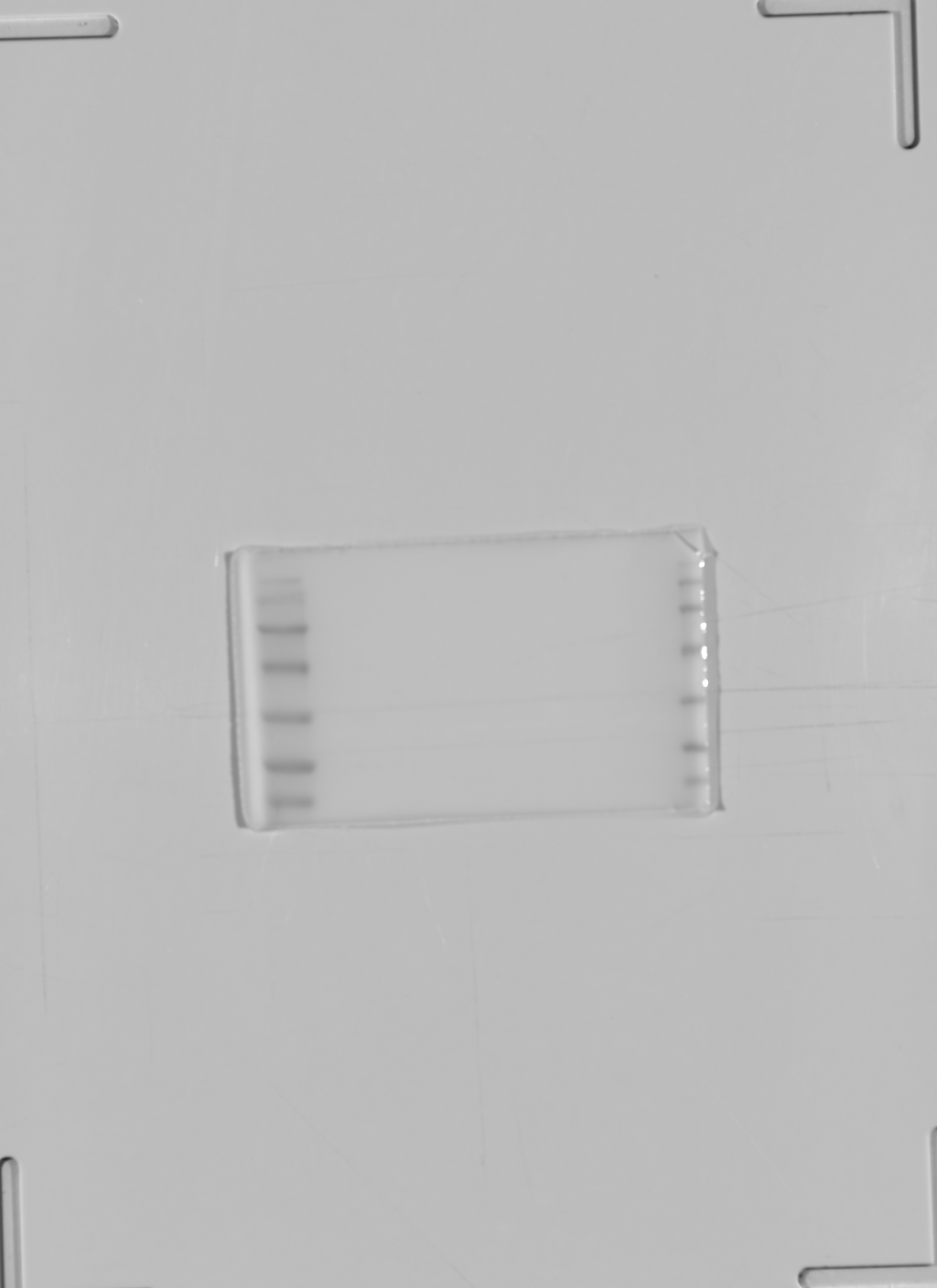

Supplement: Supplementary file 3 — WB Raw data [file 41420_2025_2583_MOESM3_ESM.zip › Figure S3 Panel B/dcaf13 2022.04.14_11.42.08_Ch/dcaf13 2022.04.14_11.42.08_Ch-Marker.tif]

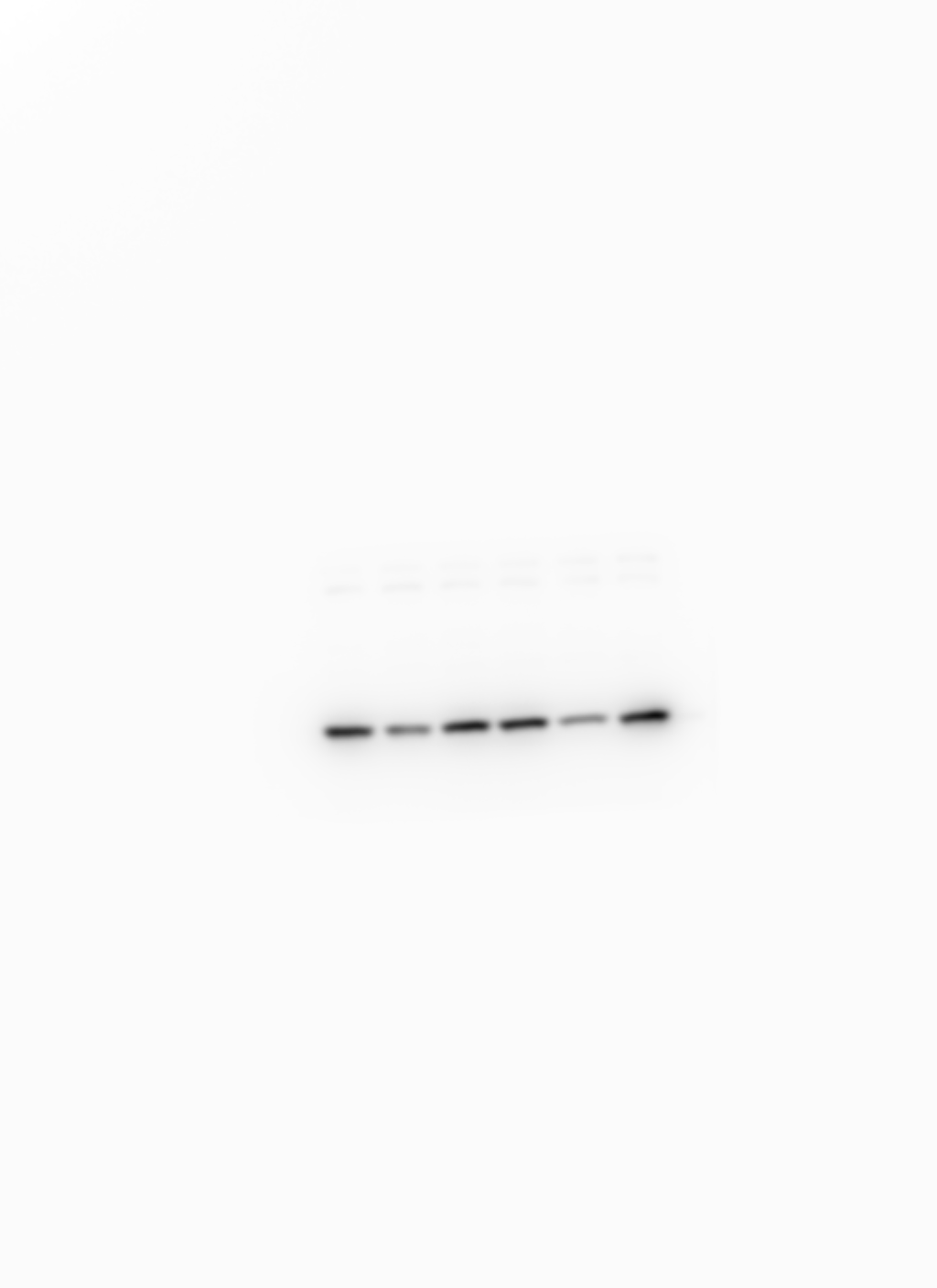

Supplement: Supplementary file 3 — WB Raw data [file 41420_2025_2583_MOESM3_ESM.zip › Figure S3 Panel B/dcaf13 2022.04.14_11.42.08_Ch/dcaf13 2022.04.14_11.42.08_Ch.tif]

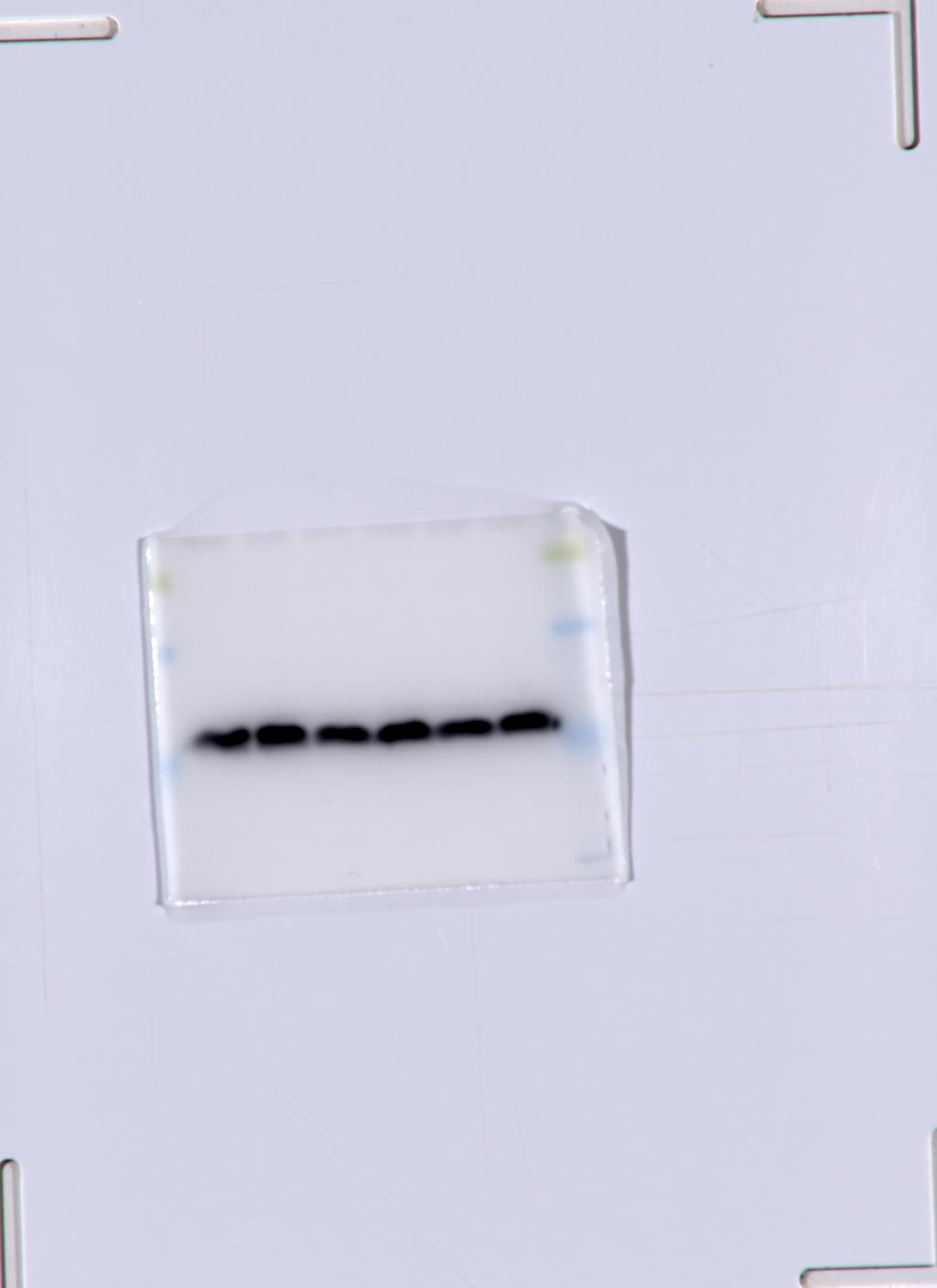

Supplement: Supplementary file 3 — WB Raw data [file 41420_2025_2583_MOESM3_ESM.zip › Figure S3 Panel B/h3 2022.04.14_11.58.40_Ch/h3 2022.04.14_11.58.40_Ch+Marker.jpg]

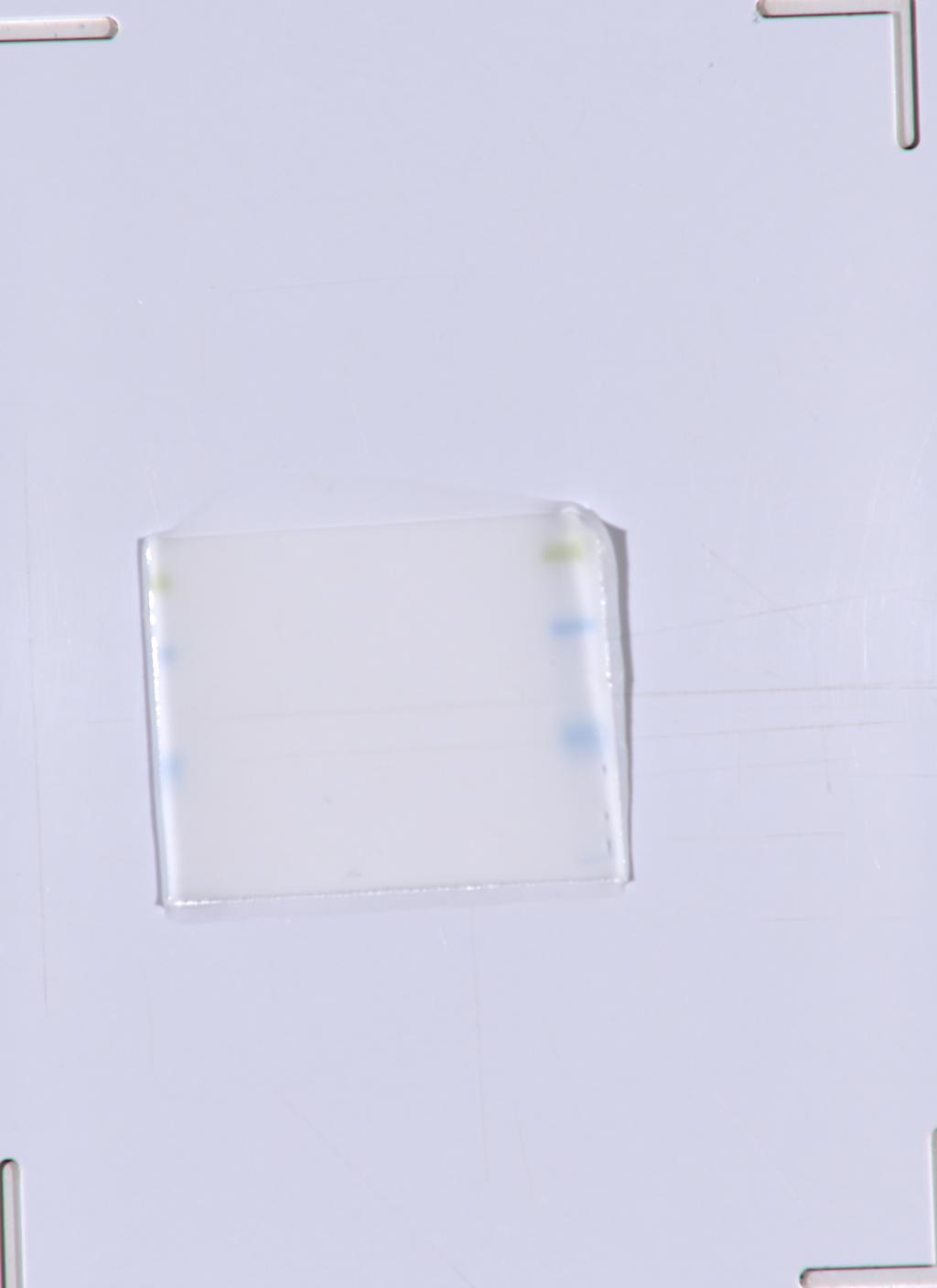

Supplement: Supplementary file 3 — WB Raw data [file 41420_2025_2583_MOESM3_ESM.zip › Figure S3 Panel B/h3 2022.04.14_11.58.40_Ch/h3 2022.04.14_11.58.40_Ch-Marker.jpg]

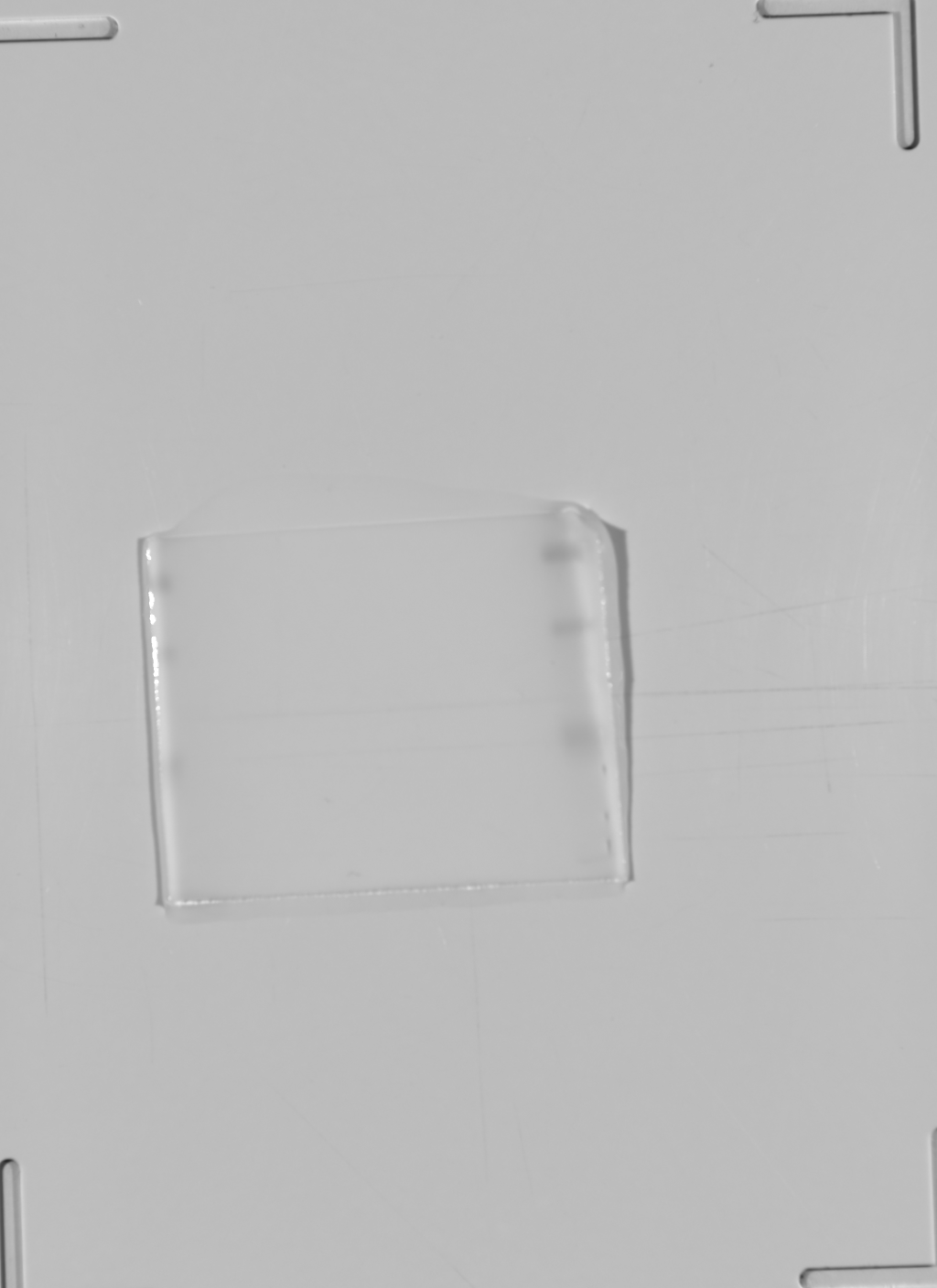

Supplement: Supplementary file 3 — WB Raw data [file 41420_2025_2583_MOESM3_ESM.zip › Figure S3 Panel B/h3 2022.04.14_11.58.40_Ch/h3 2022.04.14_11.58.40_Ch-Marker.tif]

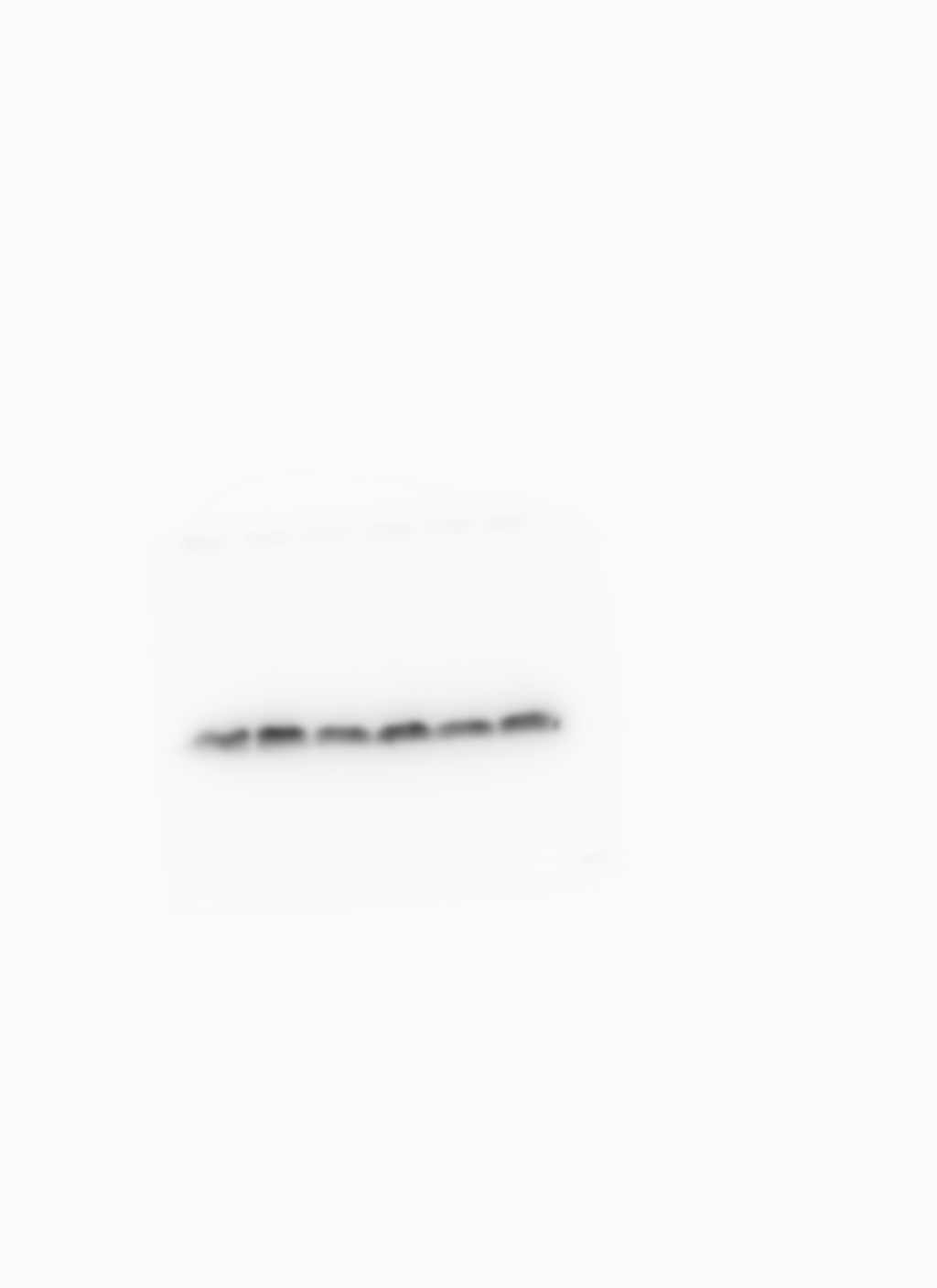

Supplement: Supplementary file 3 — WB Raw data [file 41420_2025_2583_MOESM3_ESM.zip › Figure S3 Panel B/h3 2022.04.14_11.58.40_Ch/h3 2022.04.14_11.58.40_Ch.tif]

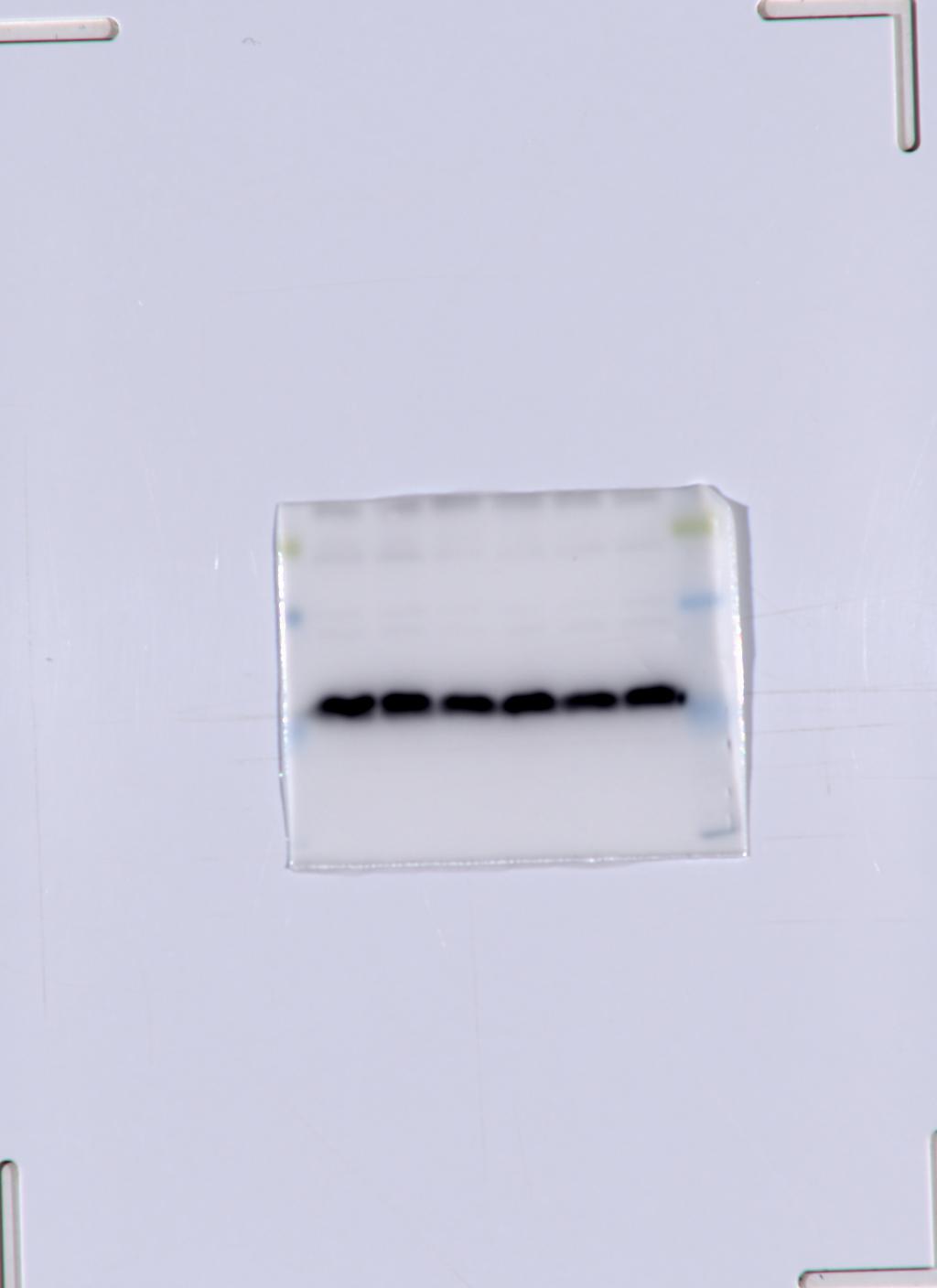

Supplement: Supplementary file 3 — WB Raw data [file 41420_2025_2583_MOESM3_ESM.zip › Figure S3 Panel B/h3 2022.04.14_12.01.36_Ch/h3 2022.04.14_12.01.36_Ch+Marker.jpg]

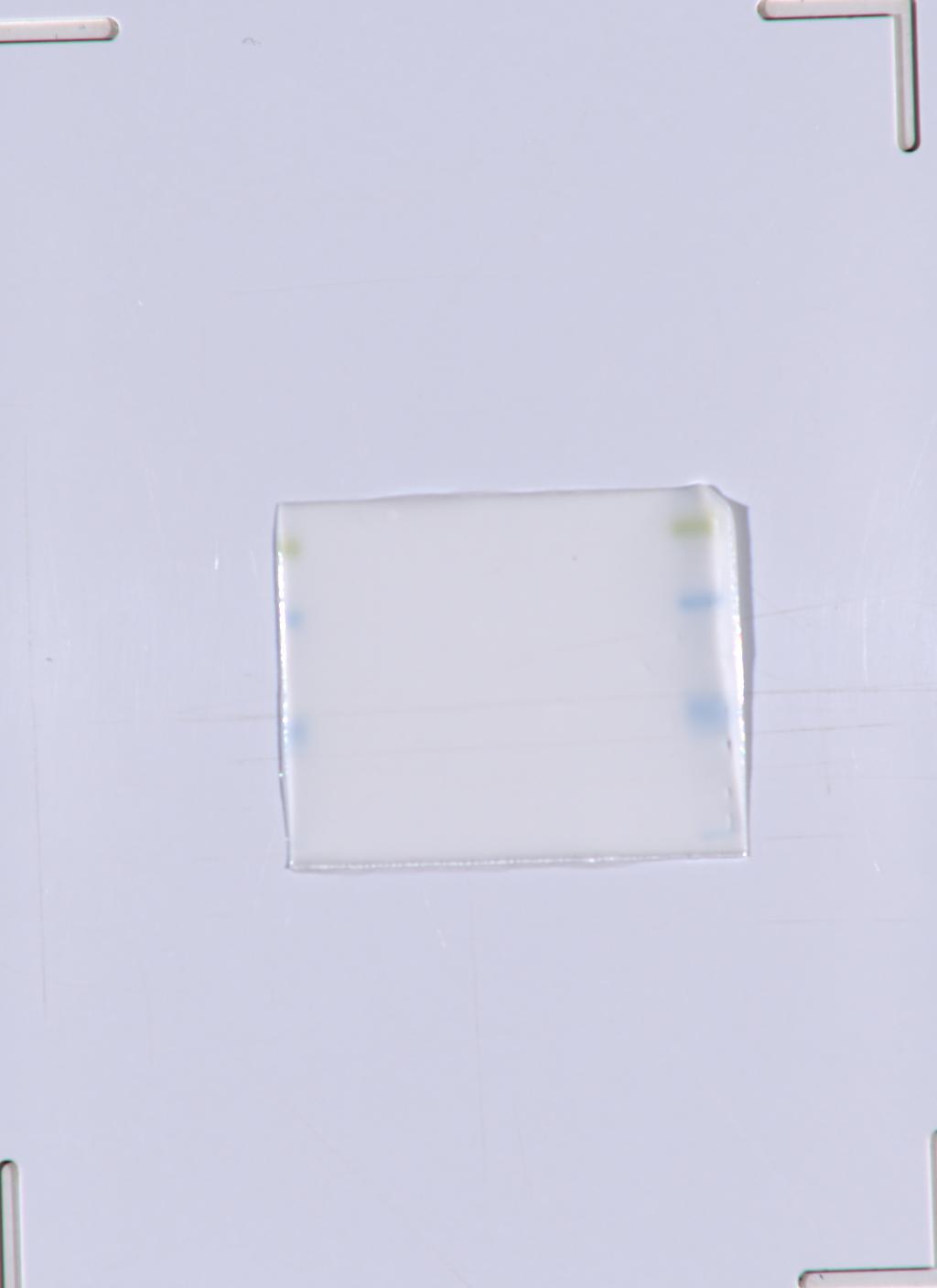

Supplement: Supplementary file 3 — WB Raw data [file 41420_2025_2583_MOESM3_ESM.zip › Figure S3 Panel B/h3 2022.04.14_12.01.36_Ch/h3 2022.04.14_12.01.36_Ch-Marker.jpg]

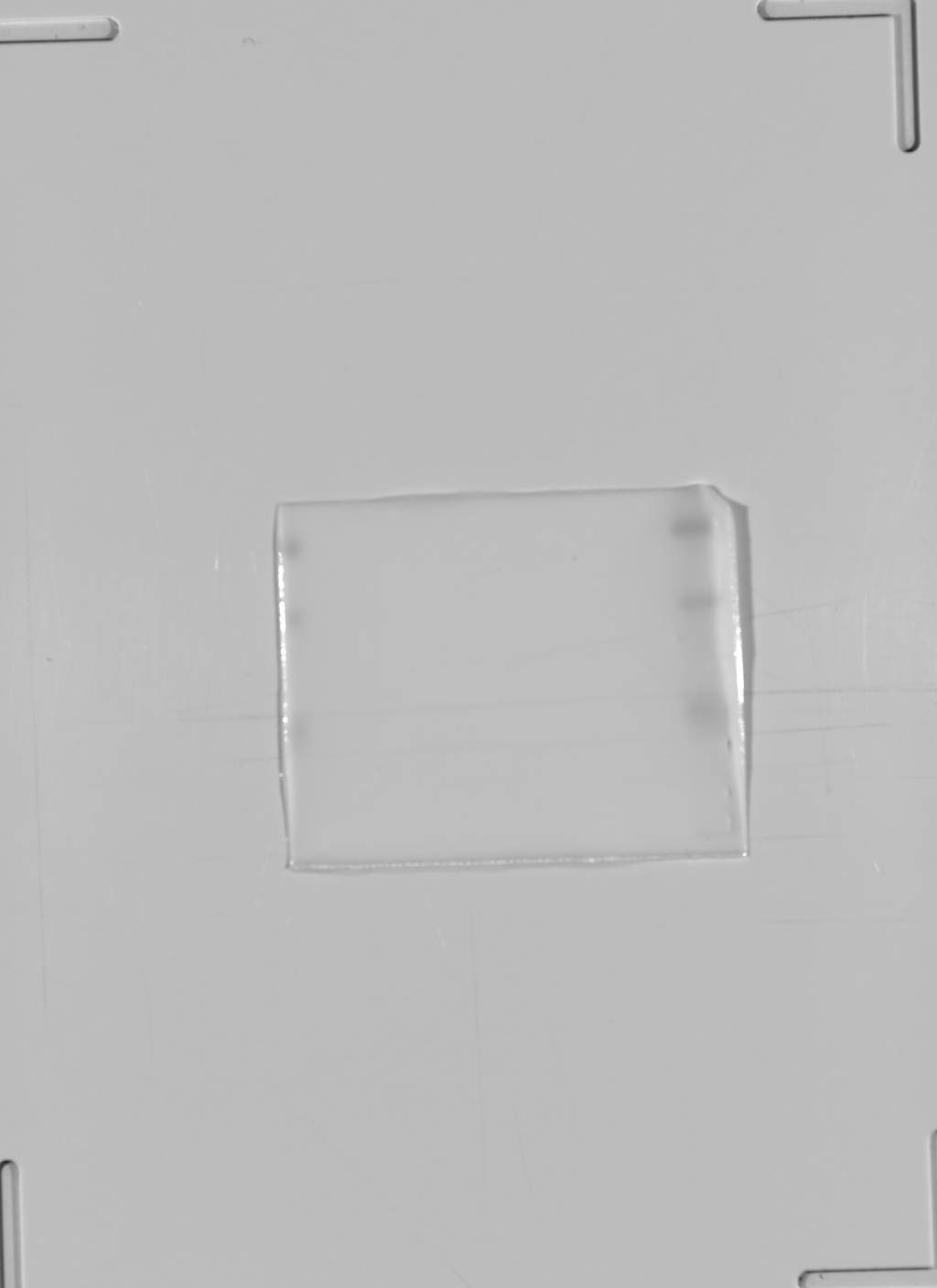

Supplement: Supplementary file 3 — WB Raw data [file 41420_2025_2583_MOESM3_ESM.zip › Figure S3 Panel B/h3 2022.04.14_12.01.36_Ch/h3 2022.04.14_12.01.36_Ch-Marker.tif]

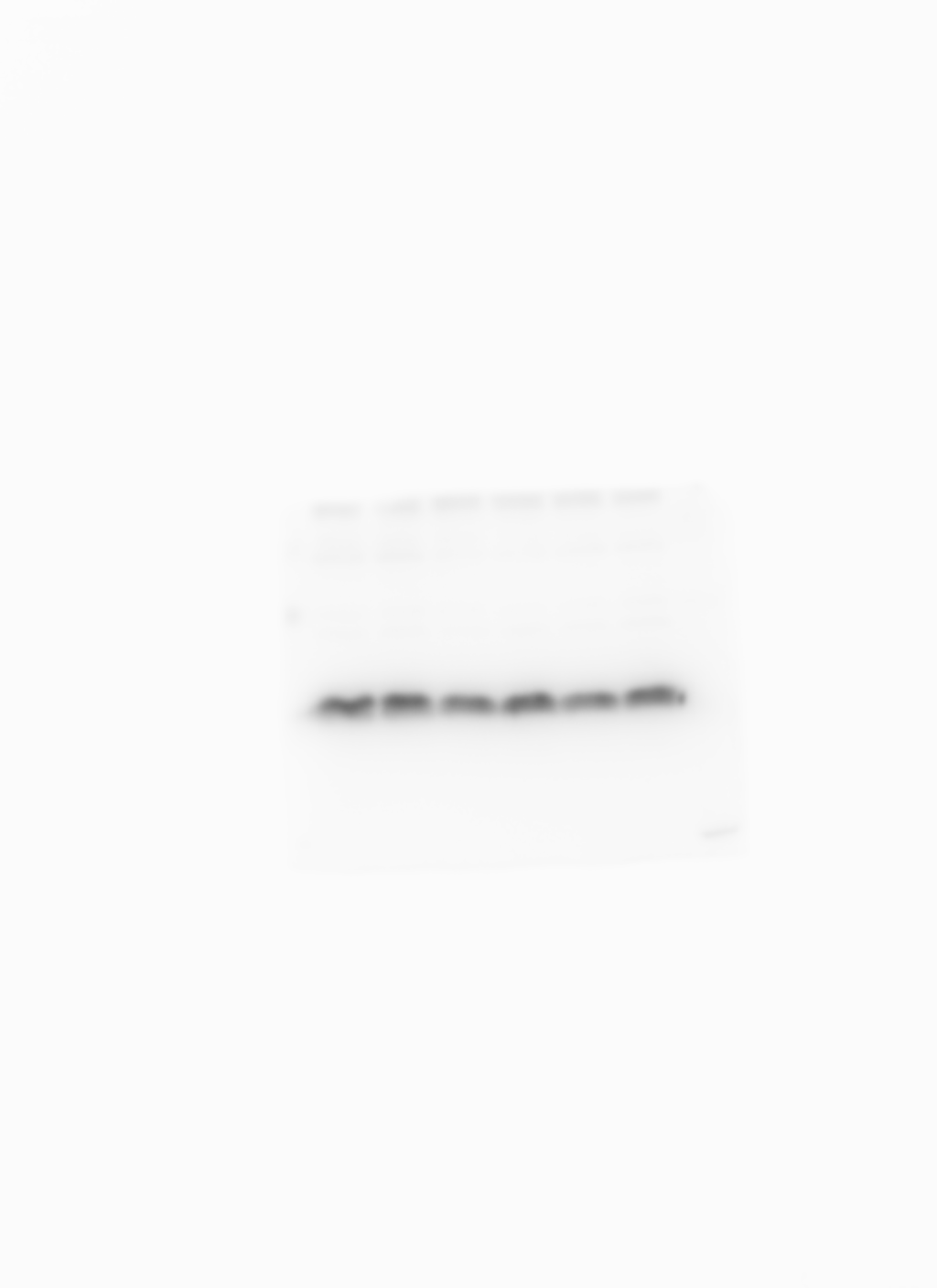

Supplement: Supplementary file 3 — WB Raw data [file 41420_2025_2583_MOESM3_ESM.zip › Figure S3 Panel B/h3 2022.04.14_12.01.36_Ch/h3 2022.04.14_12.01.36_Ch.tif]

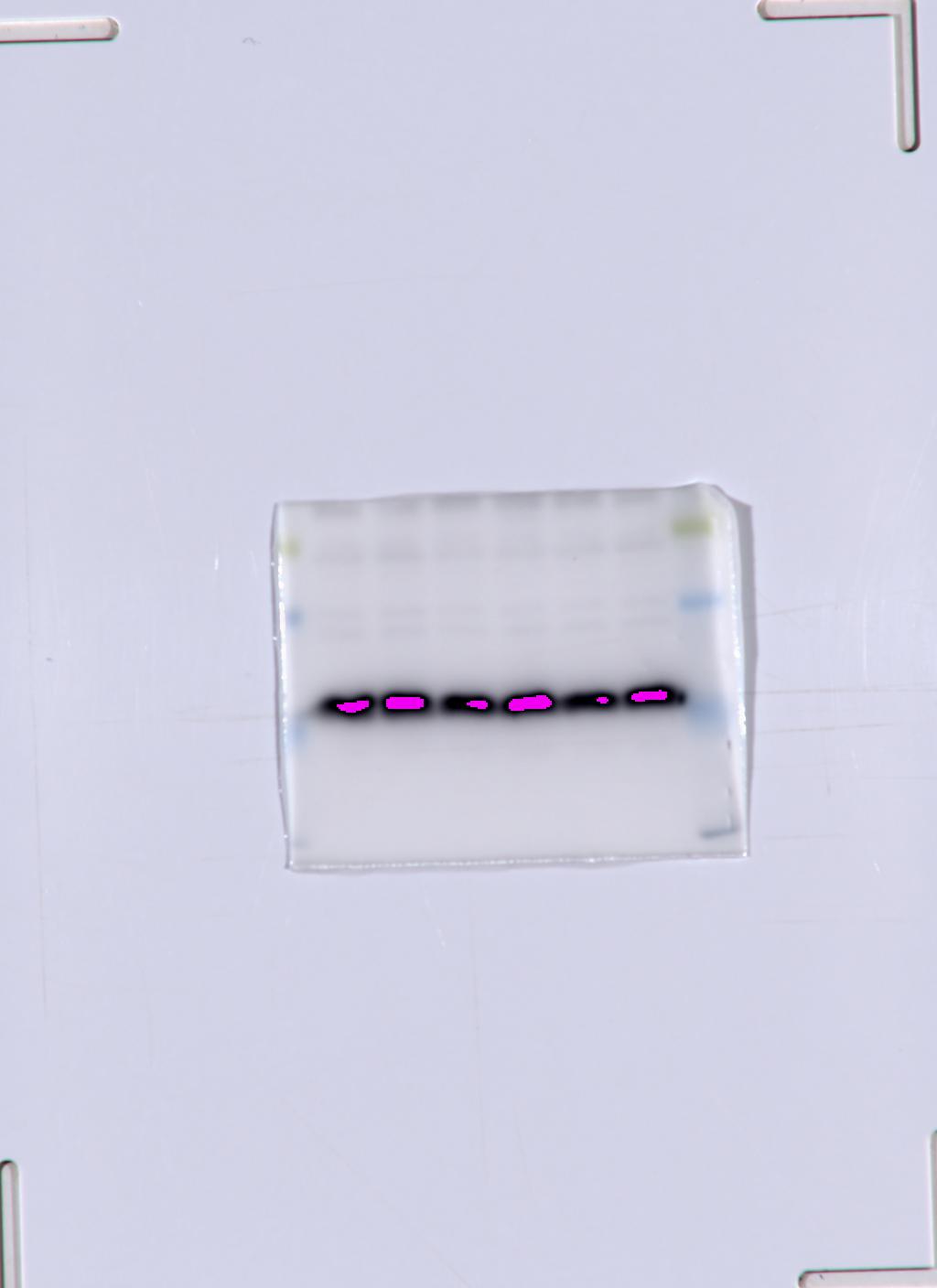

Supplement: Supplementary file 3 — WB Raw data [file 41420_2025_2583_MOESM3_ESM.zip › Figure S3 Panel B/h3 2022.04.14_12.03.13_Ch/h3 2022.04.14_12.03.13_Ch+Marker.jpg]

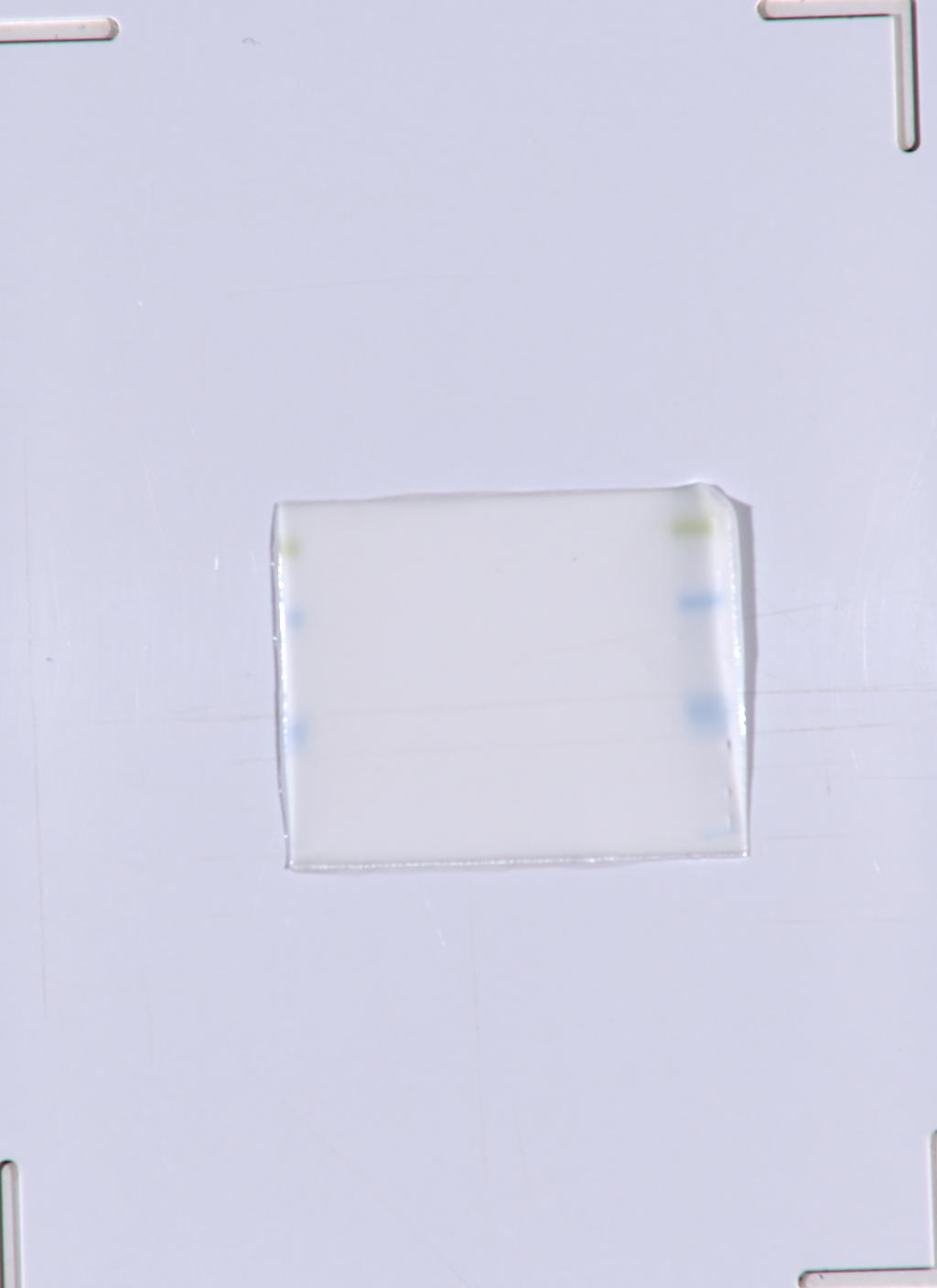

Supplement: Supplementary file 3 — WB Raw data [file 41420_2025_2583_MOESM3_ESM.zip › Figure S3 Panel B/h3 2022.04.14_12.03.13_Ch/h3 2022.04.14_12.03.13_Ch-Marker.jpg]

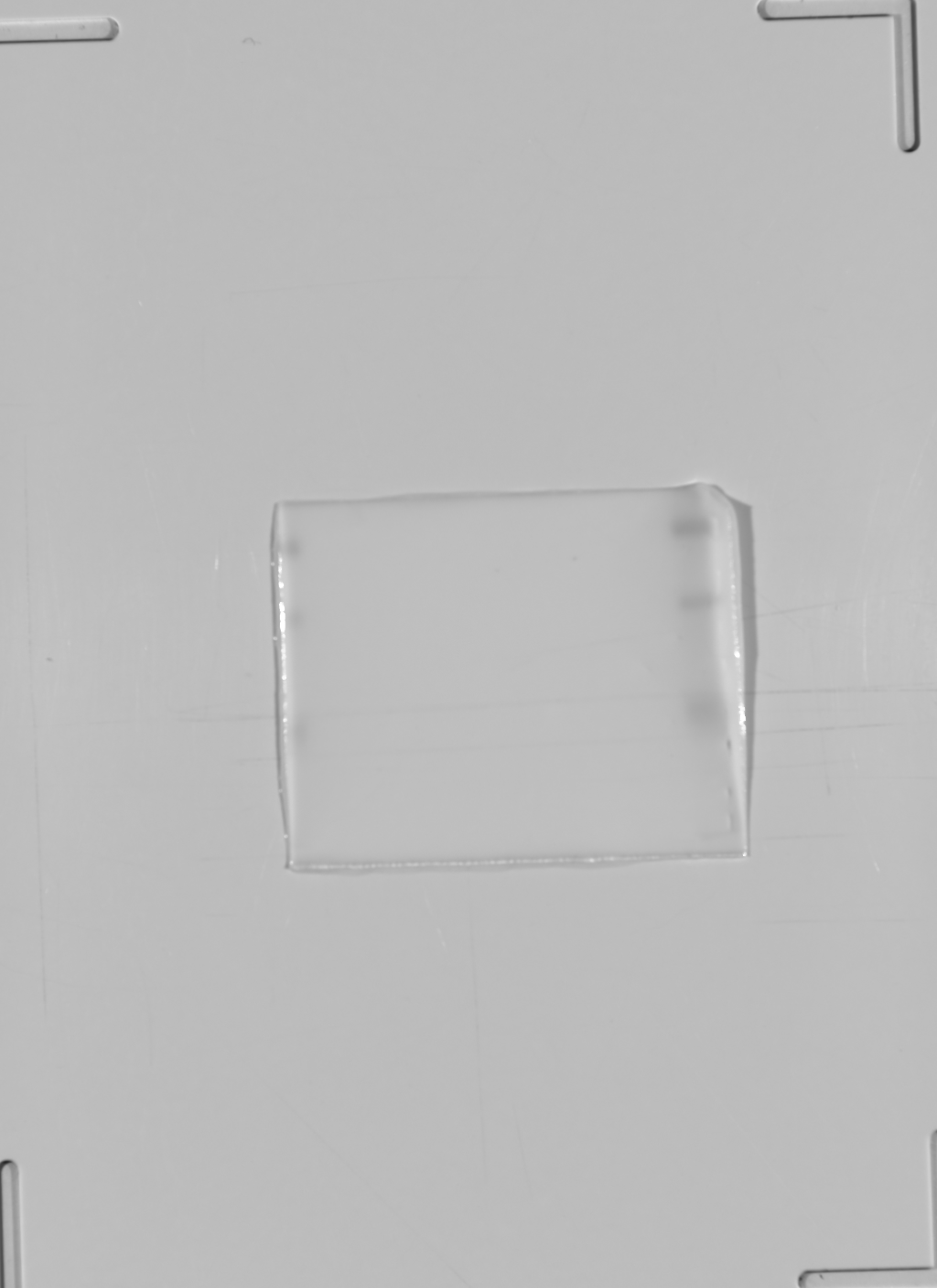

Supplement: Supplementary file 3 — WB Raw data [file 41420_2025_2583_MOESM3_ESM.zip › Figure S3 Panel B/h3 2022.04.14_12.03.13_Ch/h3 2022.04.14_12.03.13_Ch-Marker.tif]

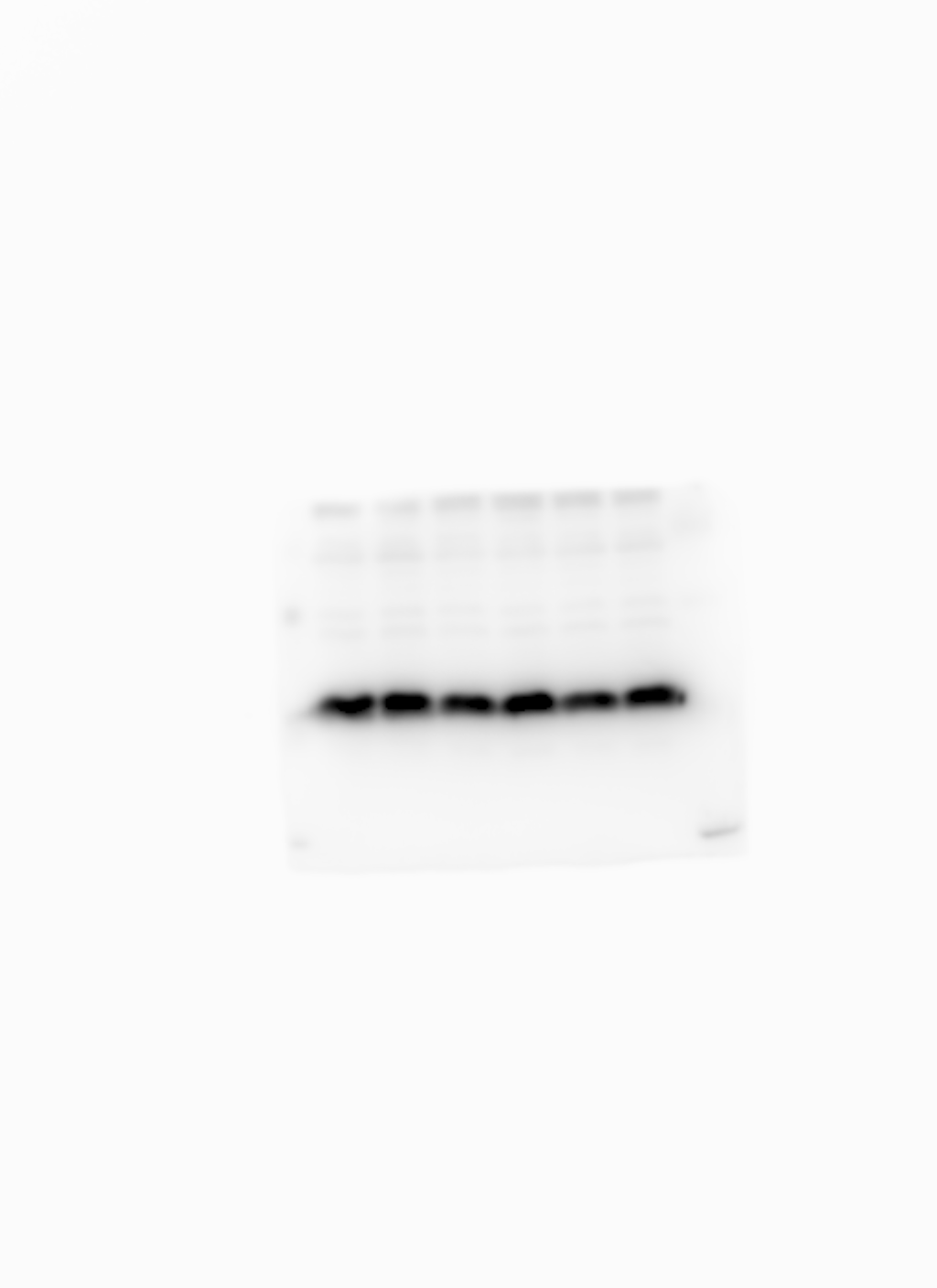

Supplement: Supplementary file 3 — WB Raw data [file 41420_2025_2583_MOESM3_ESM.zip › Figure S3 Panel B/h3 2022.04.14_12.03.13_Ch/h3 2022.04.14_12.03.13_Ch.tif]

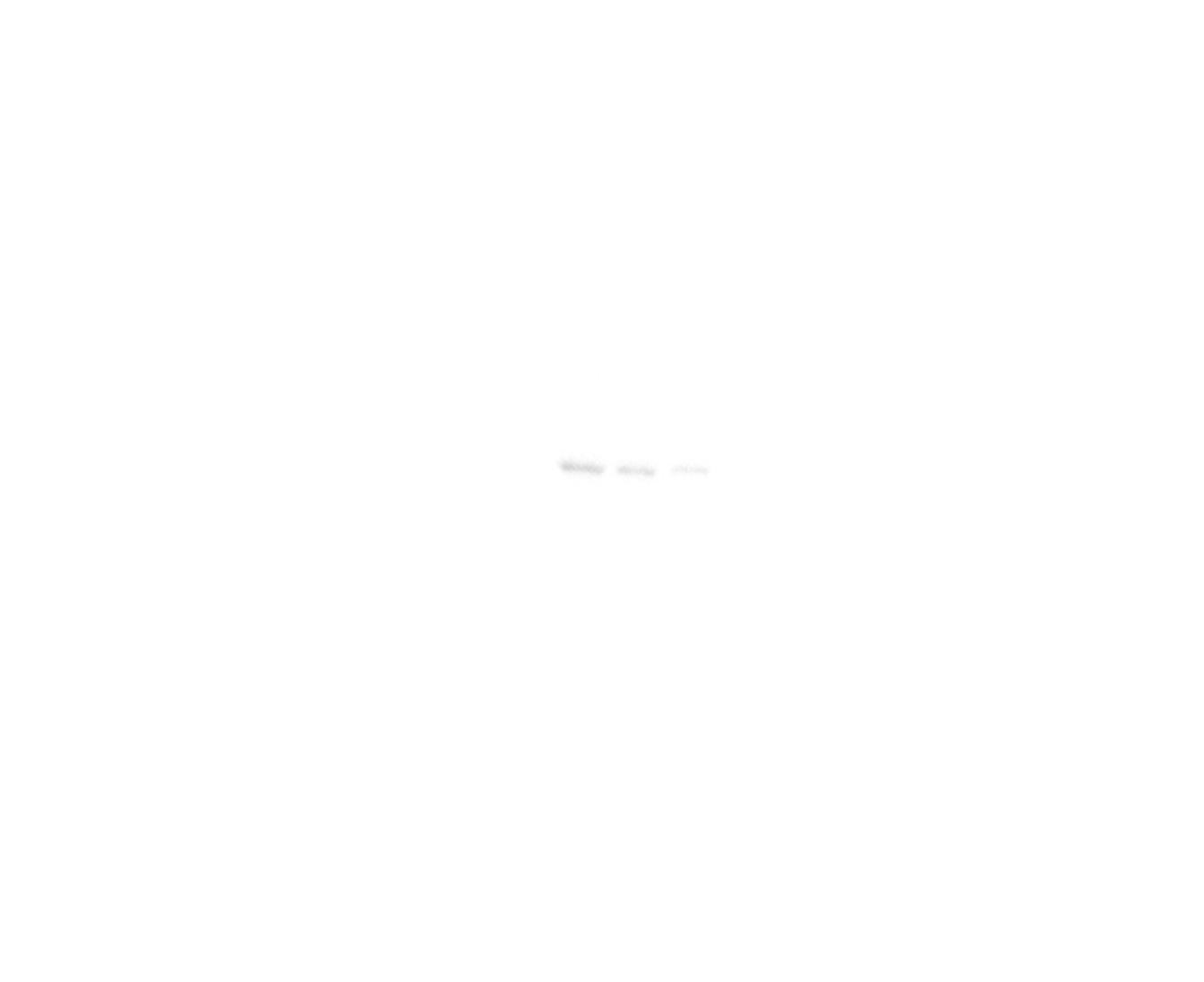

Supplement: Supplementary file 3 — WB Raw data [file 41420_2025_2583_MOESM3_ESM.zip › Figure 6 Panel B/DCAF13/dcaf131.Tif]

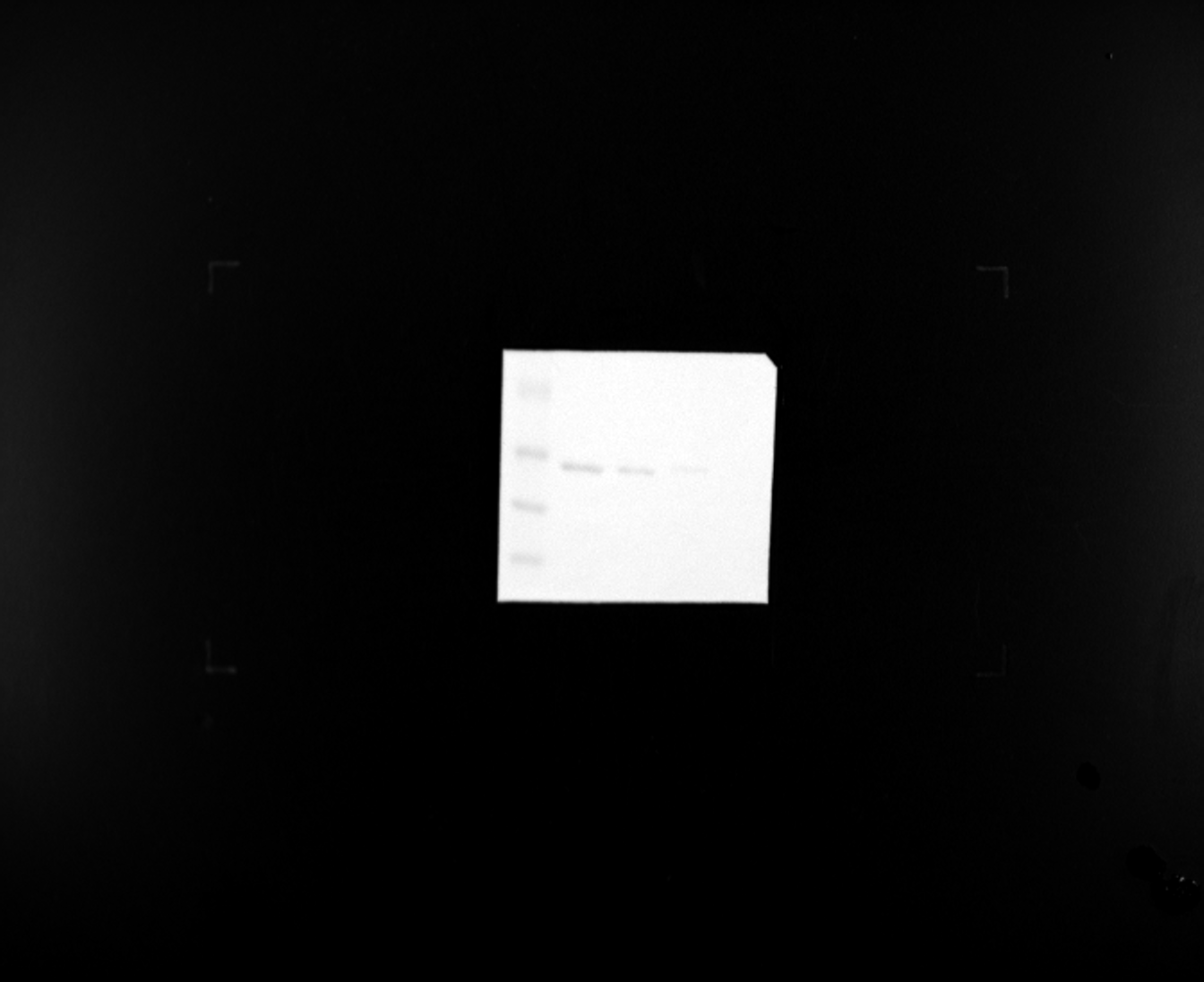

Supplement: Supplementary file 3 — WB Raw data [file 41420_2025_2583_MOESM3_ESM.zip › Figure 6 Panel B/DCAF13/dcaf131m.Tif]

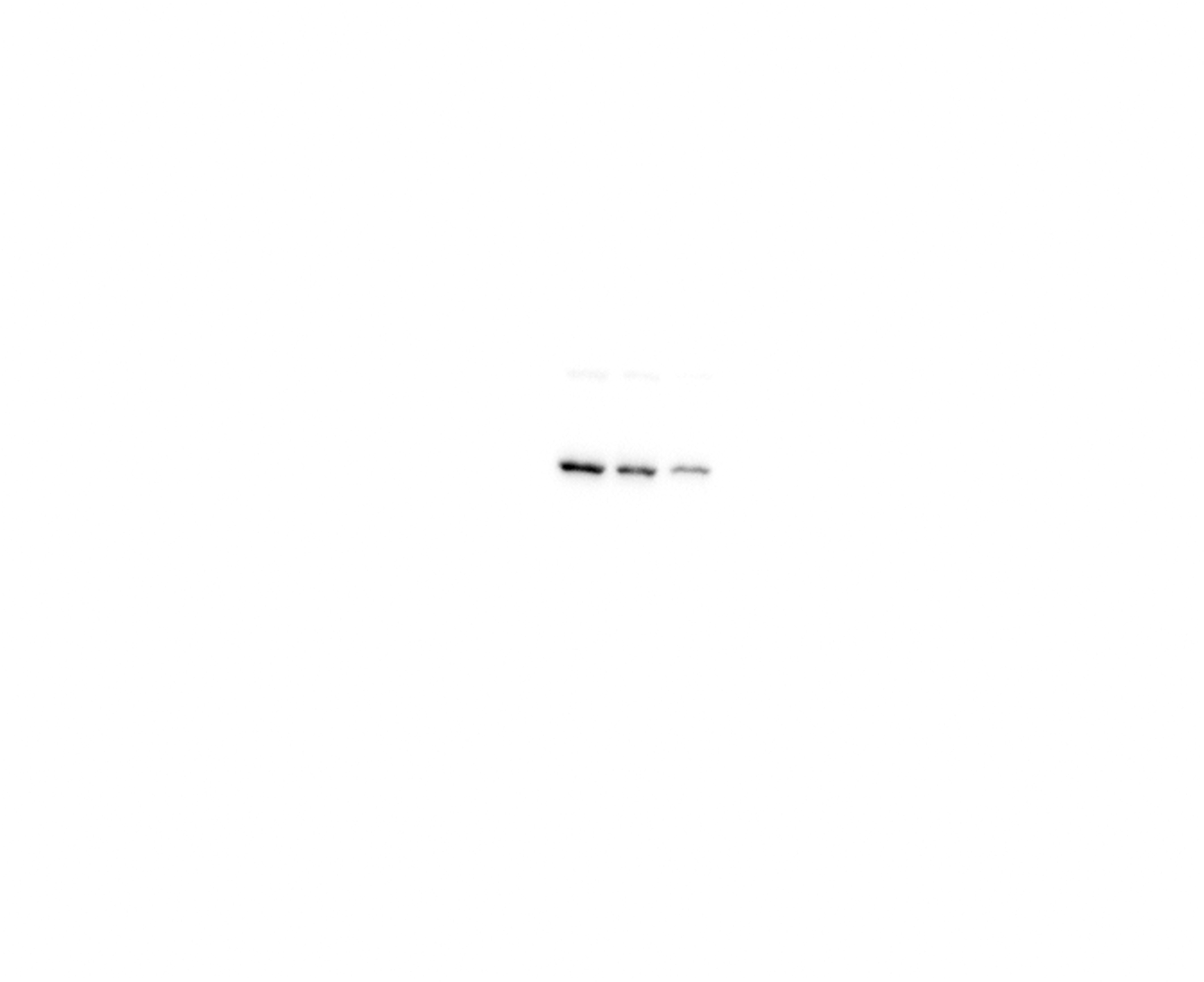

Supplement: Supplementary file 3 — WB Raw data [file 41420_2025_2583_MOESM3_ESM.zip › Figure 6 Panel B/DCAF13/dcaf132.Tif]

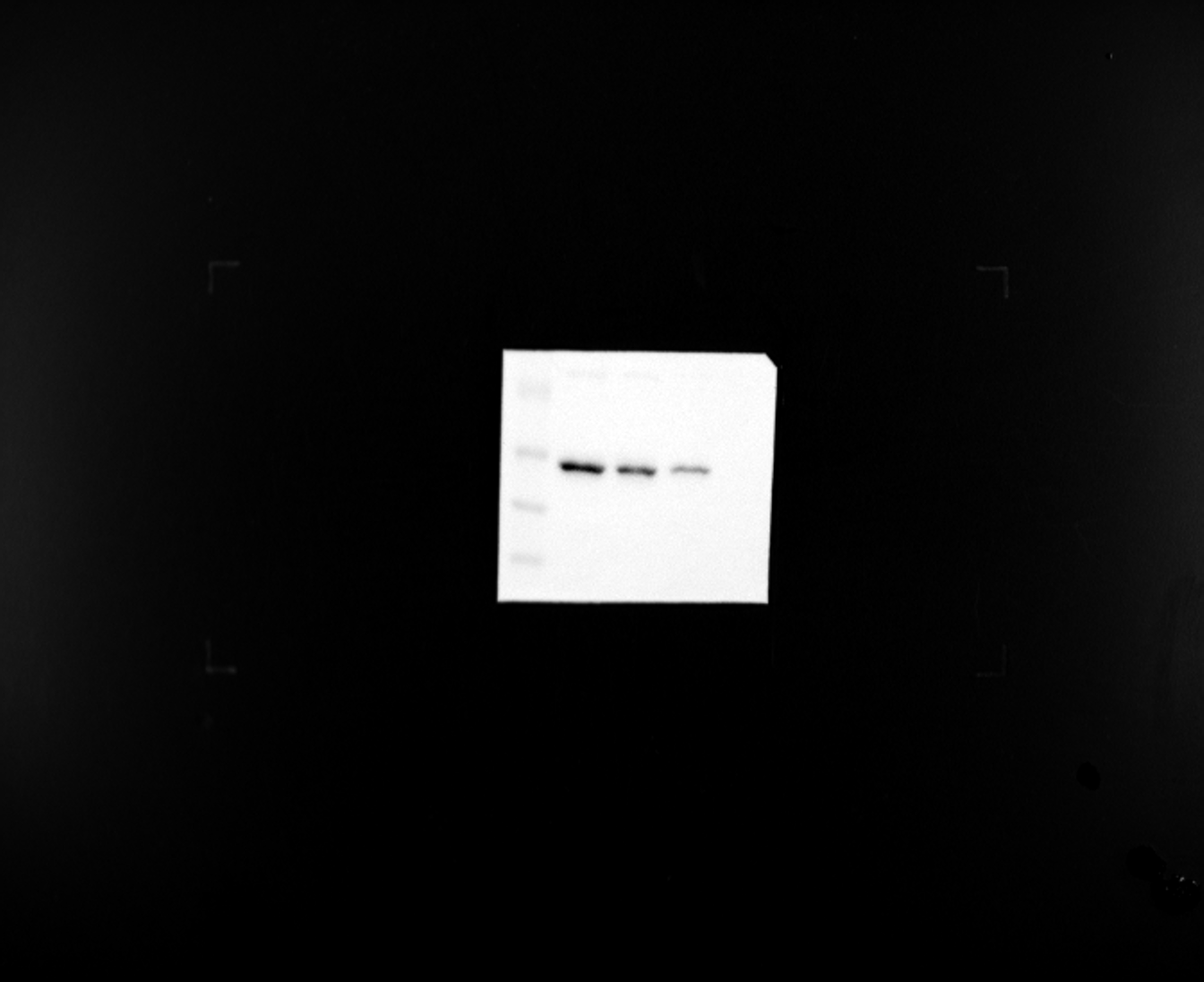

Supplement: Supplementary file 3 — WB Raw data [file 41420_2025_2583_MOESM3_ESM.zip › Figure 6 Panel B/DCAF13/dcaf132m.Tif]

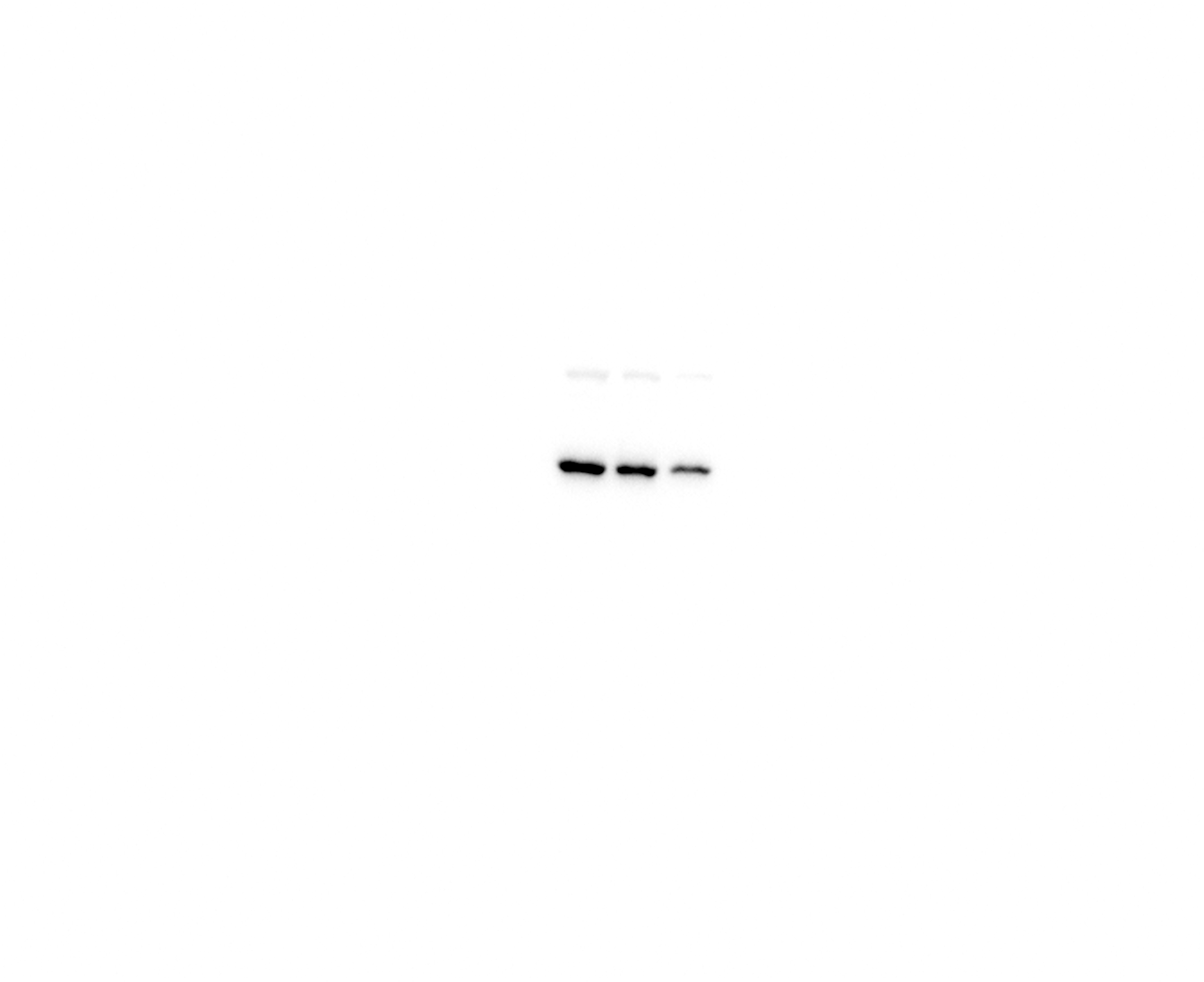

Supplement: Supplementary file 3 — WB Raw data [file 41420_2025_2583_MOESM3_ESM.zip › Figure 6 Panel B/DCAF13/dcaf133.Tif]

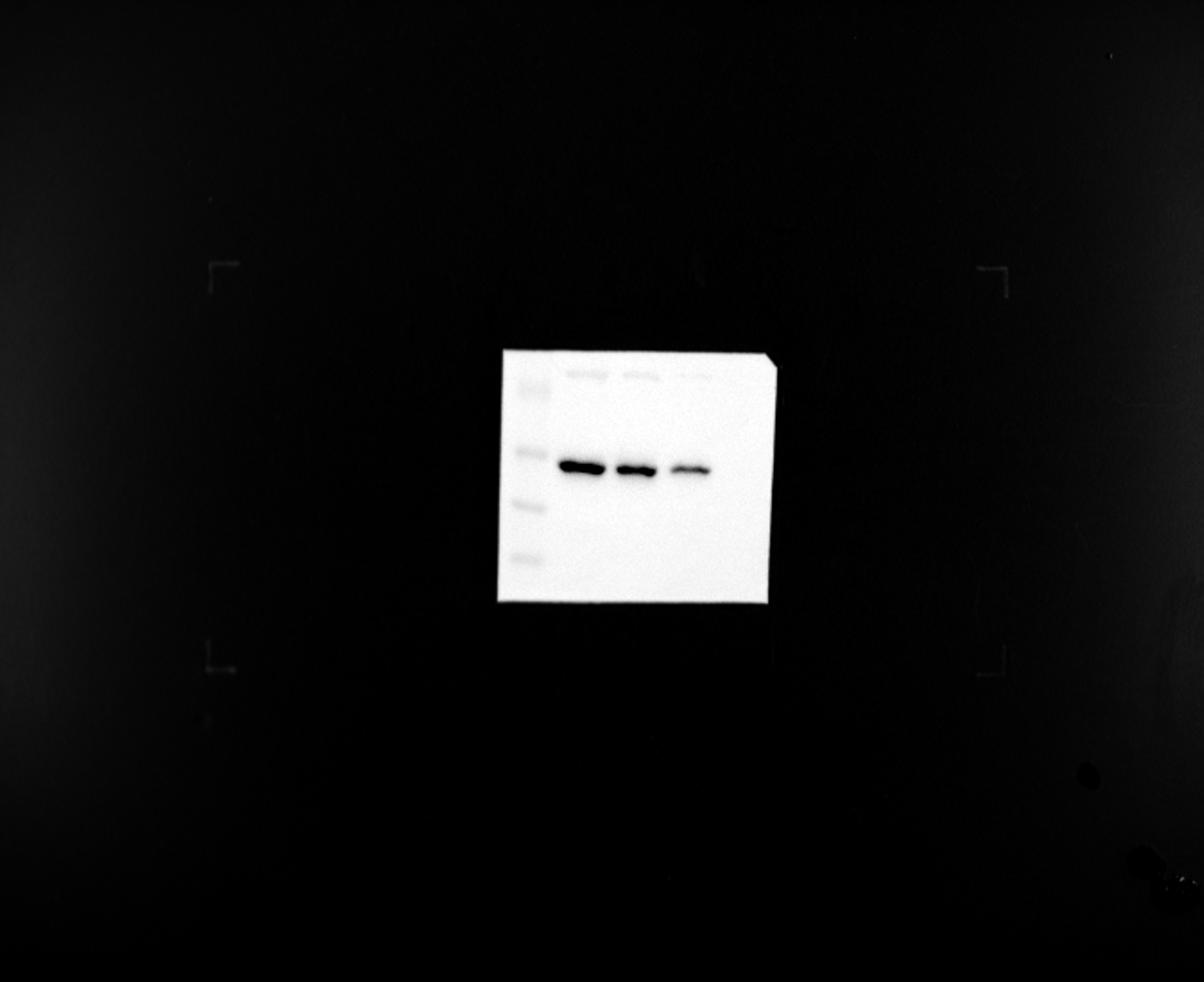

Supplement: Supplementary file 3 — WB Raw data [file 41420_2025_2583_MOESM3_ESM.zip › Figure 6 Panel B/DCAF13/dcaf133m.Tif]

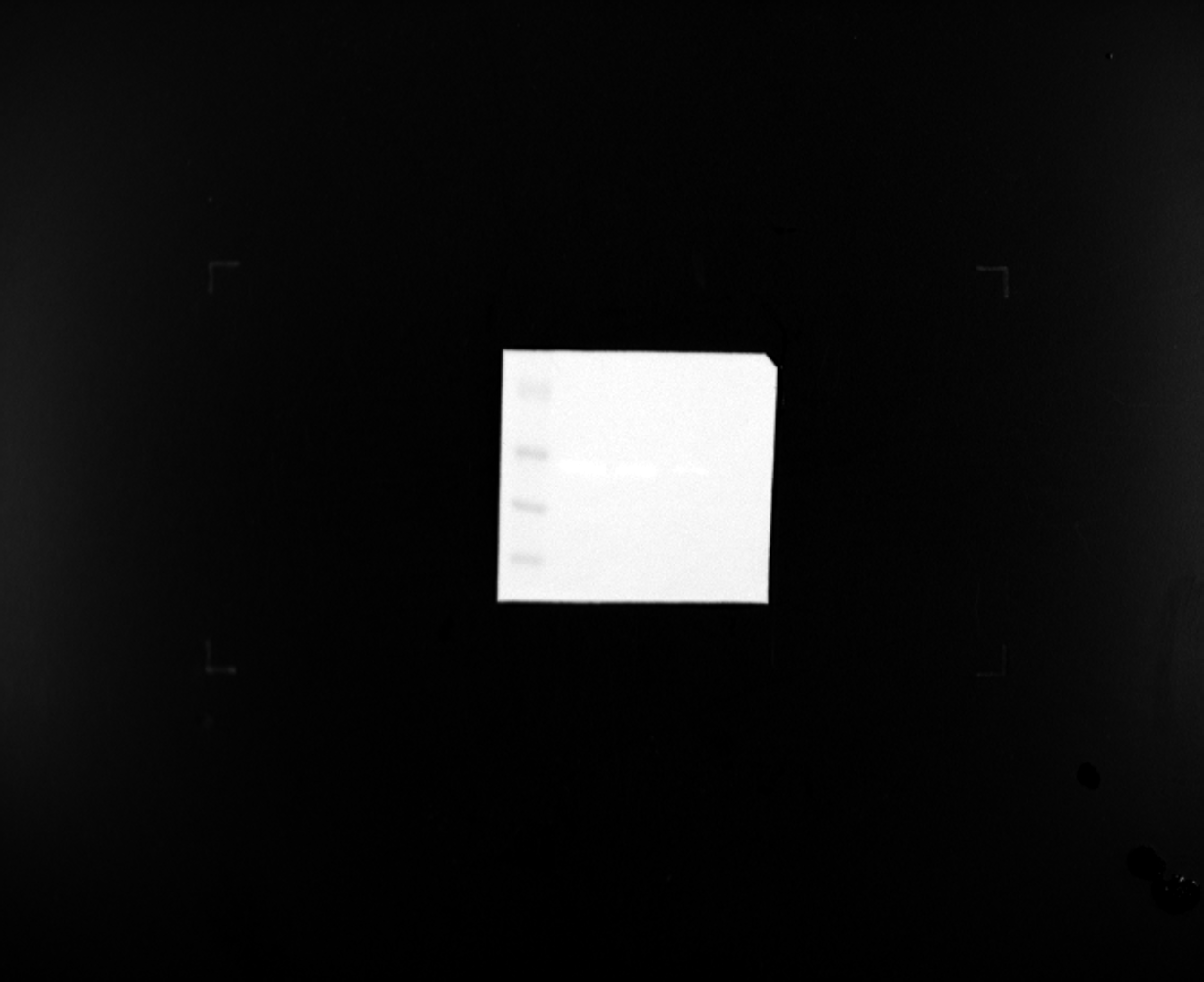

Supplement: Supplementary file 3 — WB Raw data [file 41420_2025_2583_MOESM3_ESM.zip › Figure 6 Panel B/DCAF13/dcaf13m.Tif]

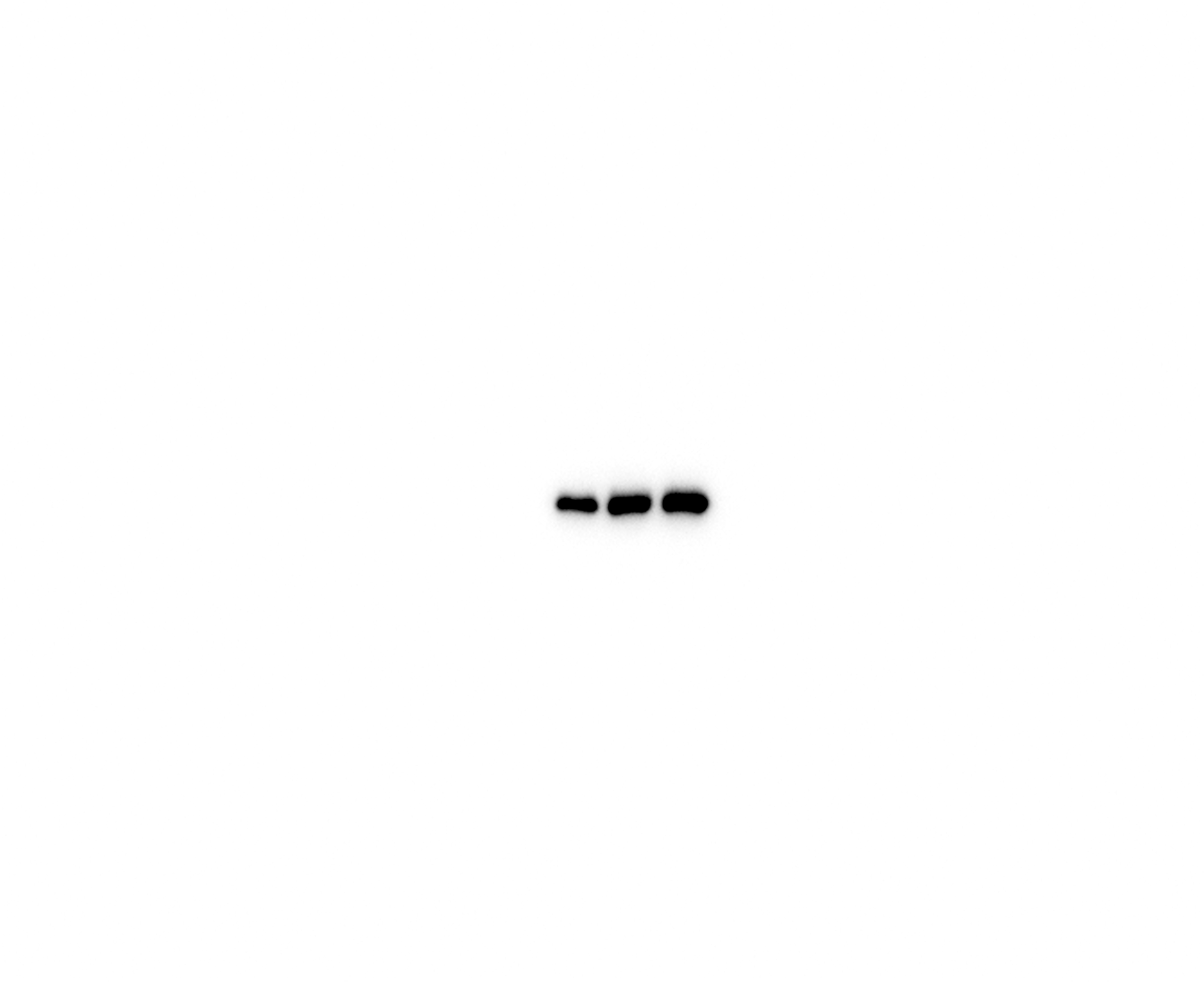

Supplement: Supplementary file 3 — WB Raw data [file 41420_2025_2583_MOESM3_ESM.zip › Figure 6 Panel B/SUV39H2/SUV39H2_1.Tif]

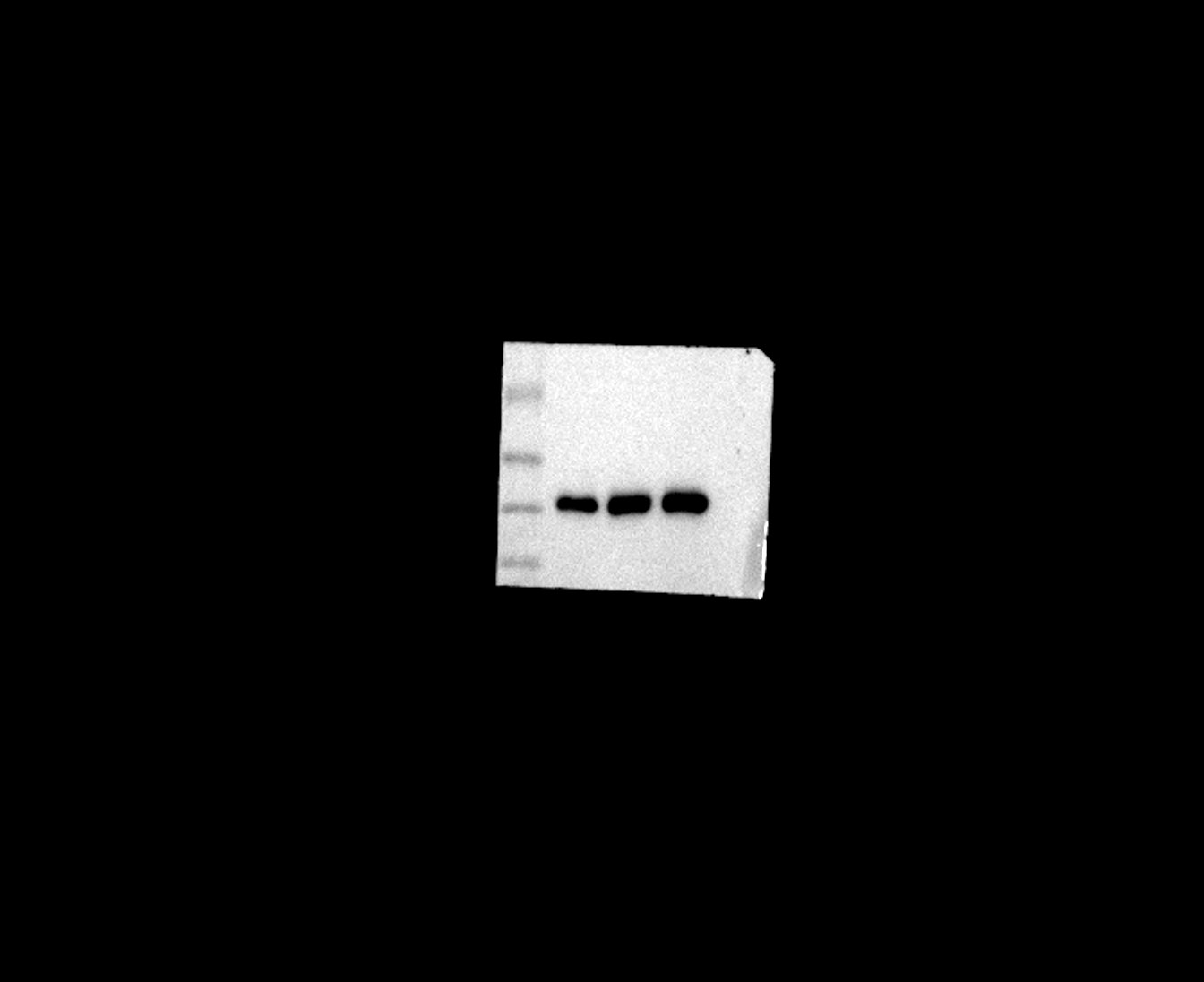

Supplement: Supplementary file 3 — WB Raw data [file 41420_2025_2583_MOESM3_ESM.zip › Figure 6 Panel B/SUV39H2/SUV39H2_1m.Tif]

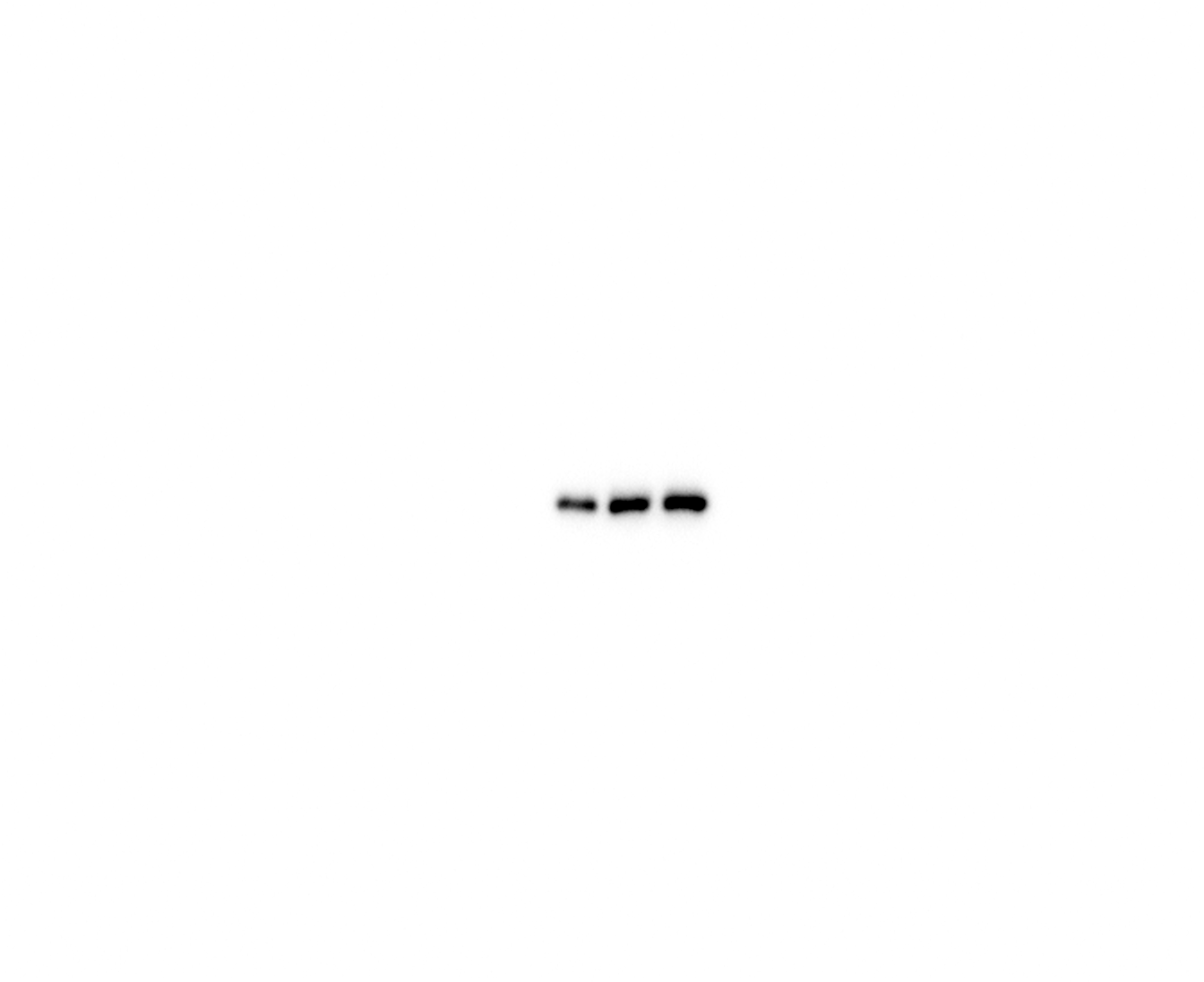

Supplement: Supplementary file 3 — WB Raw data [file 41420_2025_2583_MOESM3_ESM.zip › Figure 6 Panel B/SUV39H2/SUV39H2_2.Tif]

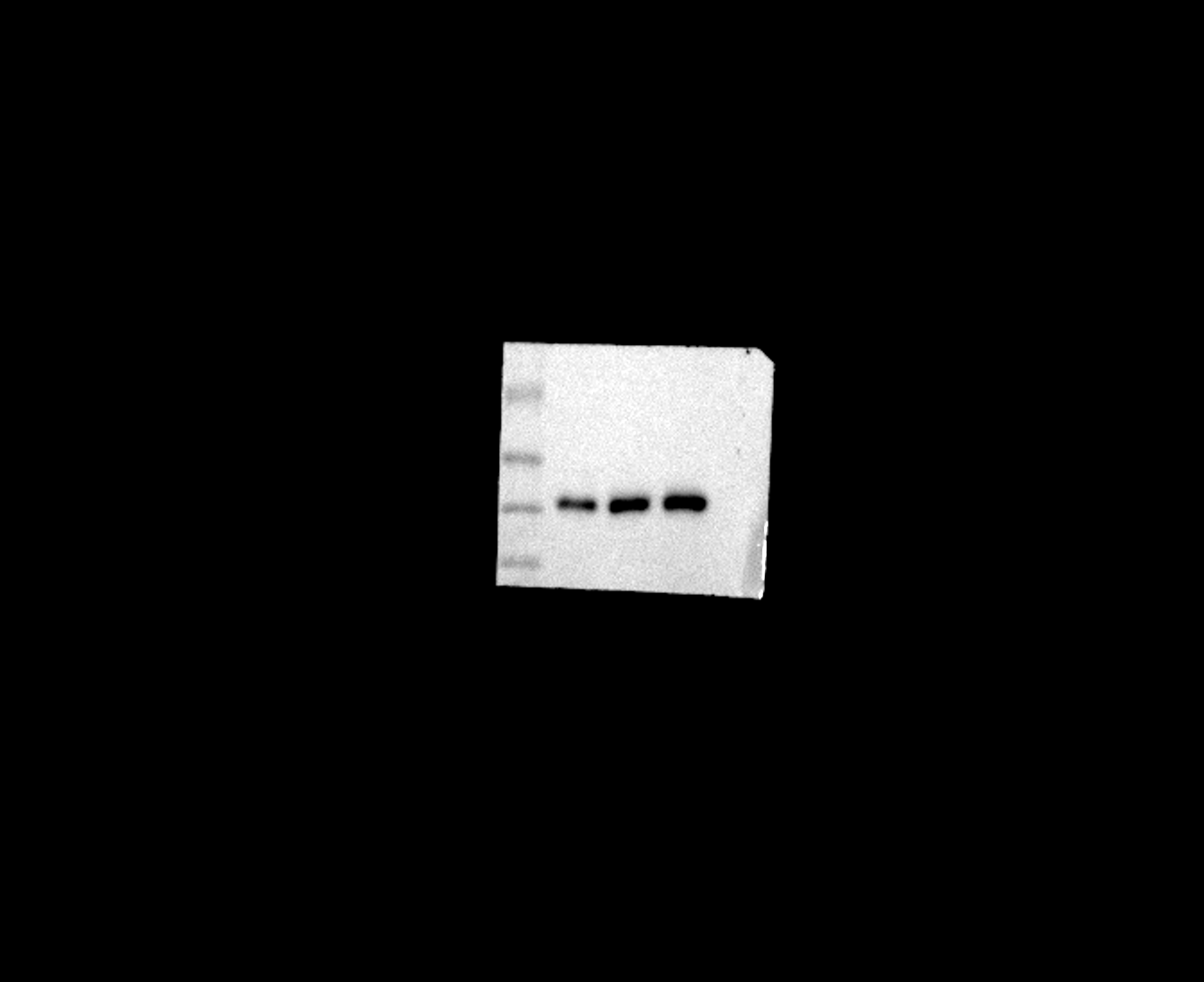

Supplement: Supplementary file 3 — WB Raw data [file 41420_2025_2583_MOESM3_ESM.zip › Figure 6 Panel B/SUV39H2/SUV39H2_2m.Tif]

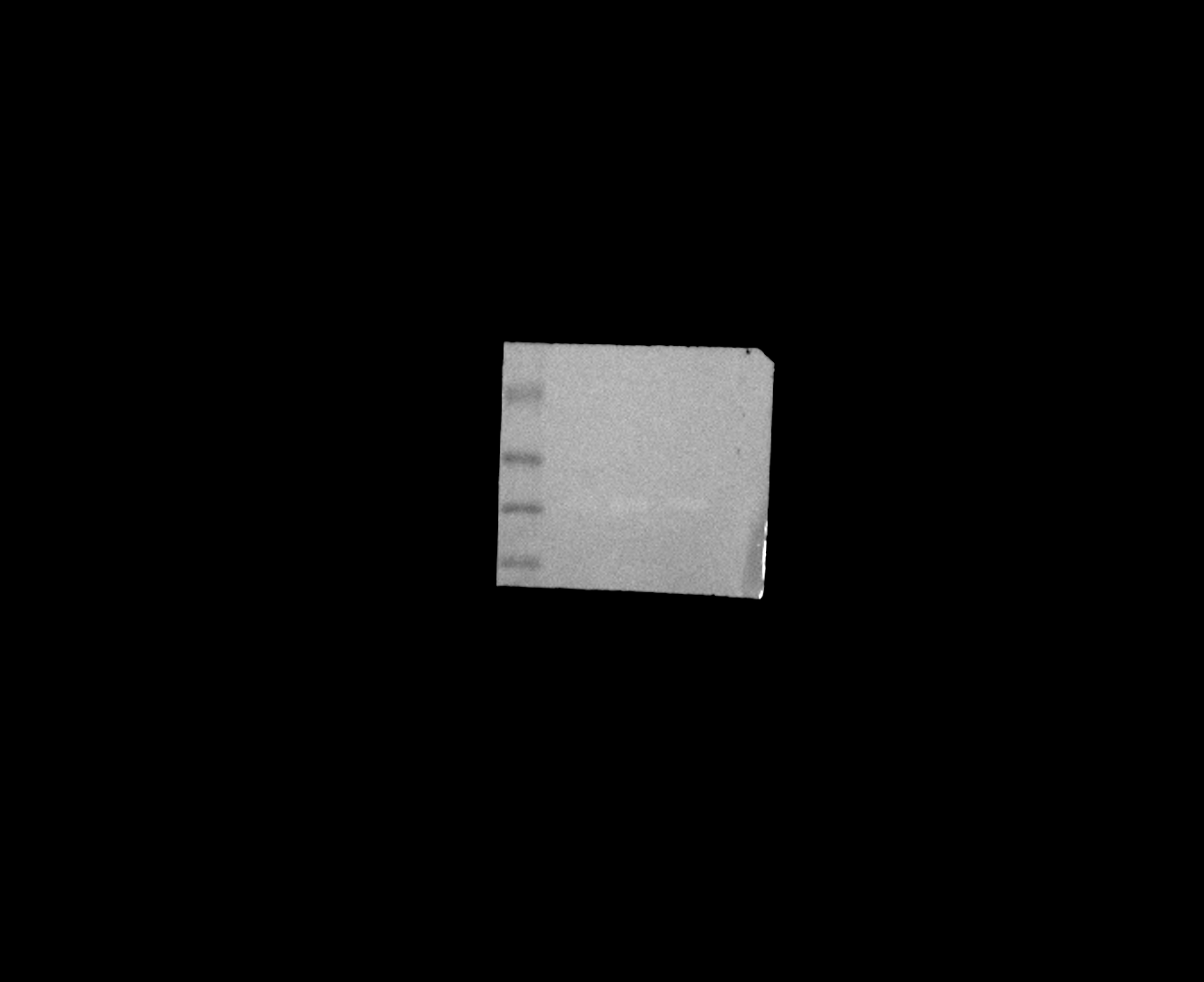

Supplement: Supplementary file 3 — WB Raw data [file 41420_2025_2583_MOESM3_ESM.zip › Figure 6 Panel B/SUV39H2/SUV39H2_m.Tif]

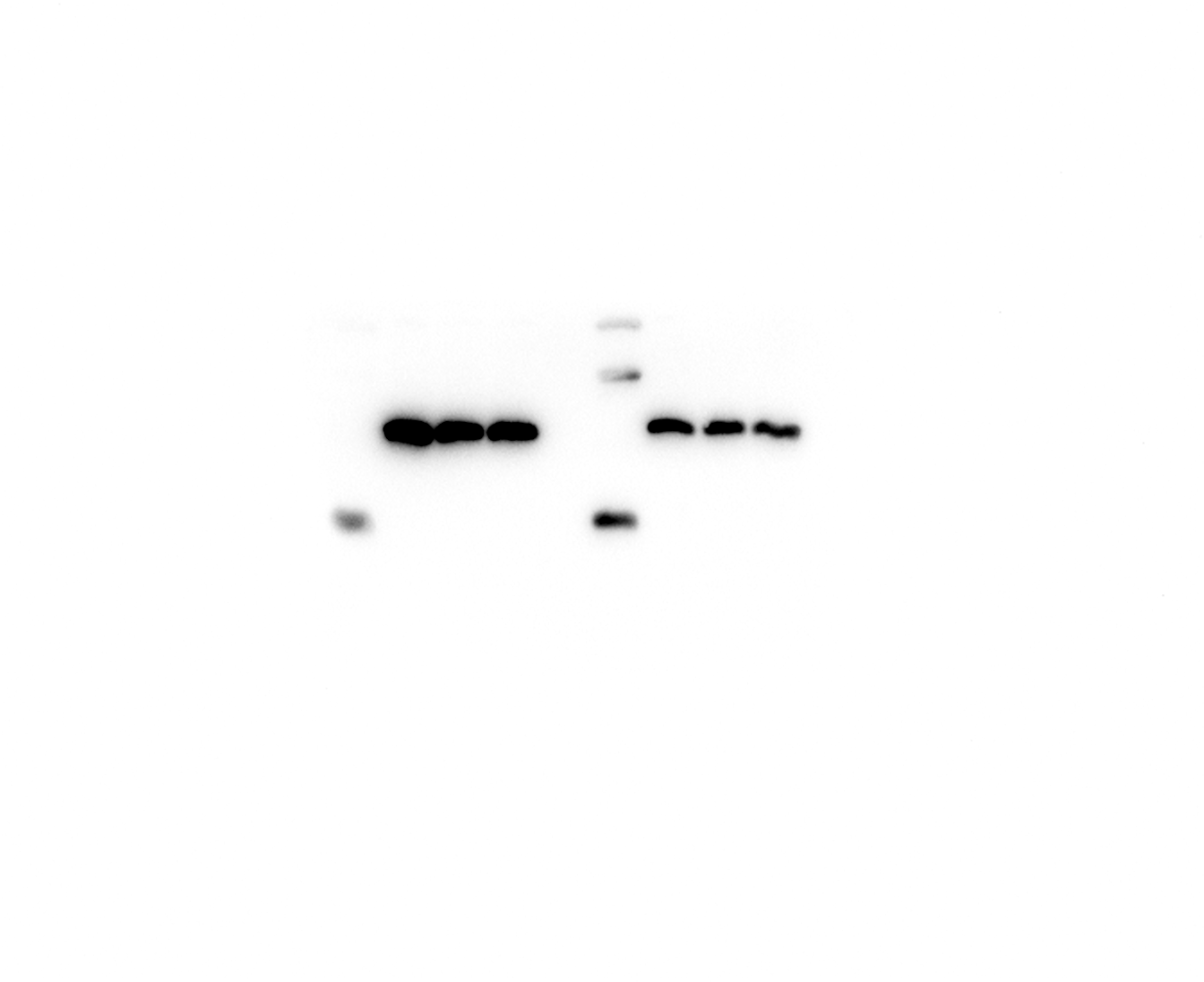

Supplement: Supplementary file 3 — WB Raw data [file 41420_2025_2583_MOESM3_ESM.zip › Figure 6 Panel B/Left is Panel B H3/h3_1.Tif]

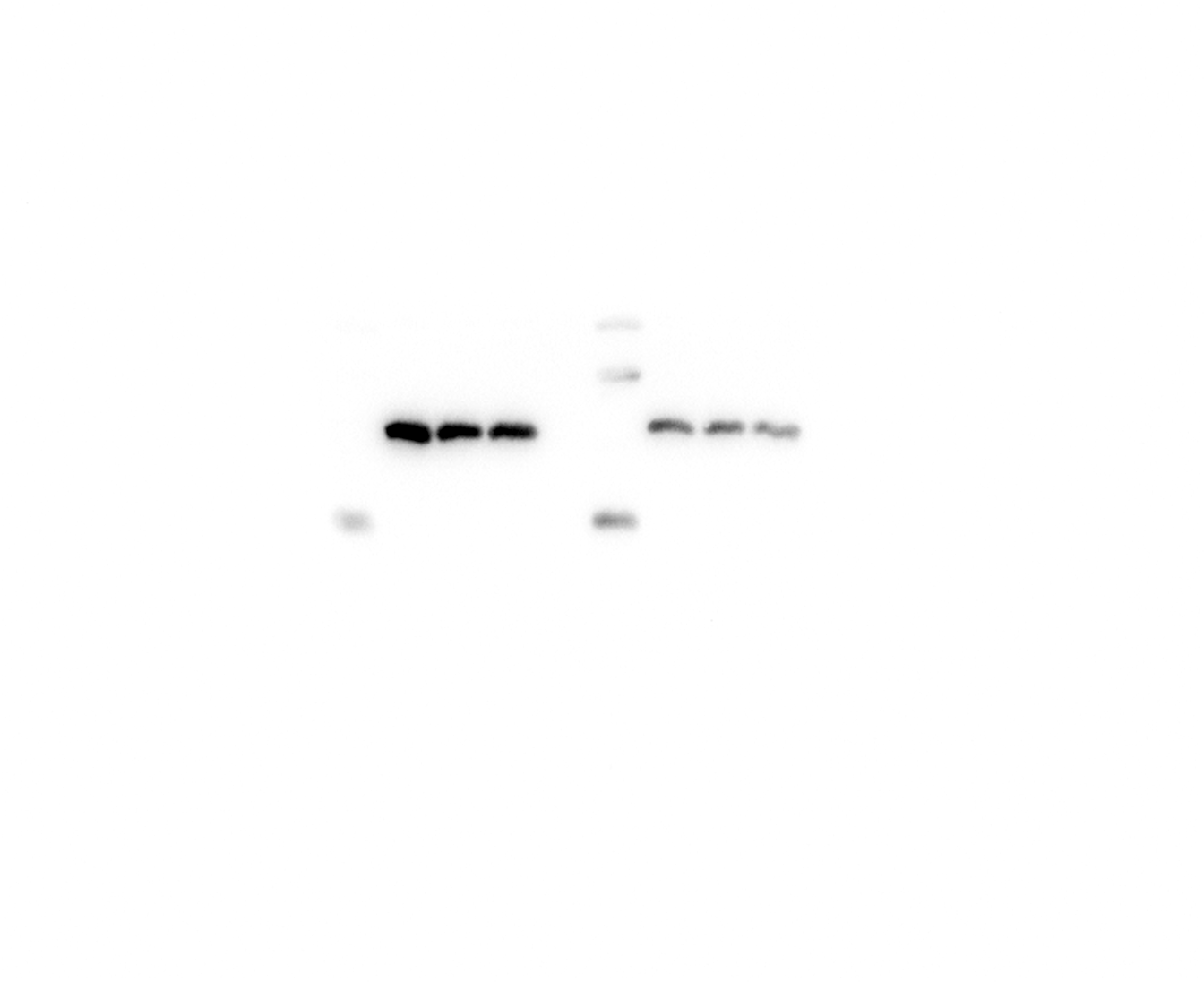

Supplement: Supplementary file 3 — WB Raw data [file 41420_2025_2583_MOESM3_ESM.zip › Figure 6 Panel B/Left is Panel B H3/h3_2.Tif]

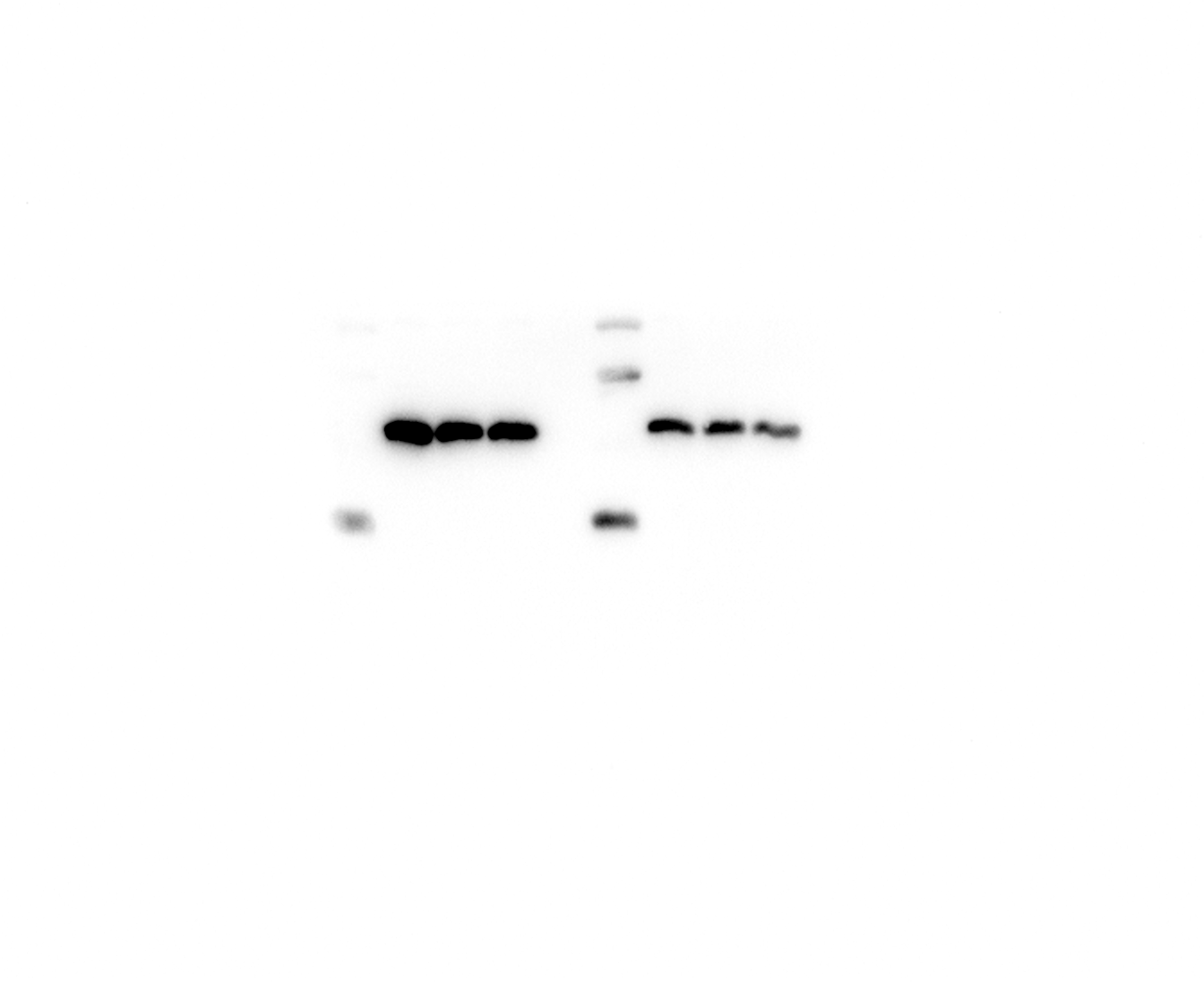

Supplement: Supplementary file 3 — WB Raw data [file 41420_2025_2583_MOESM3_ESM.zip › Figure 6 Panel B/Left is Panel B H3/h3_3.Tif]

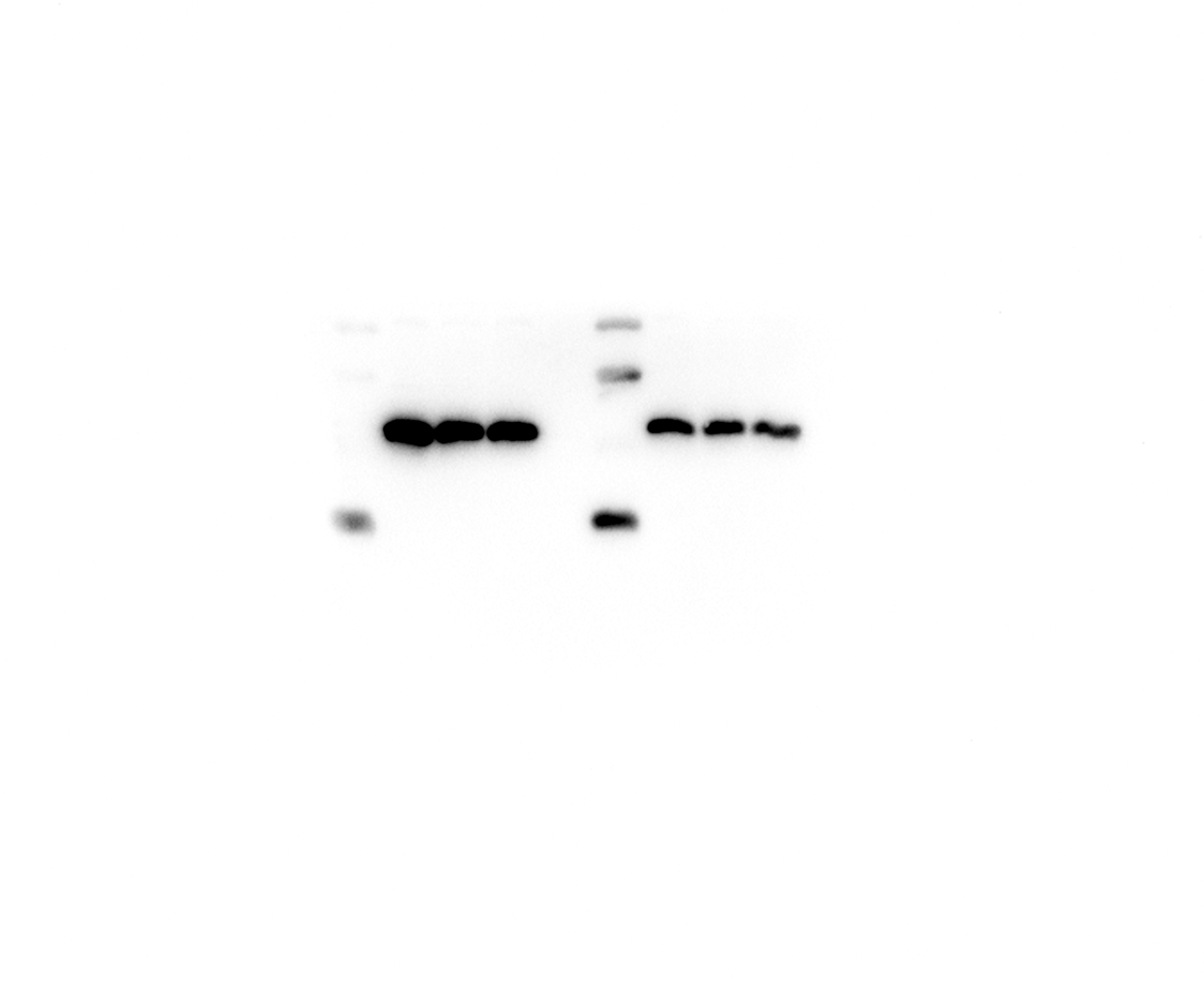

Supplement: Supplementary file 3 — WB Raw data [file 41420_2025_2583_MOESM3_ESM.zip › Figure 6 Panel B/Left is Panel B H3/h3_4.Tif]

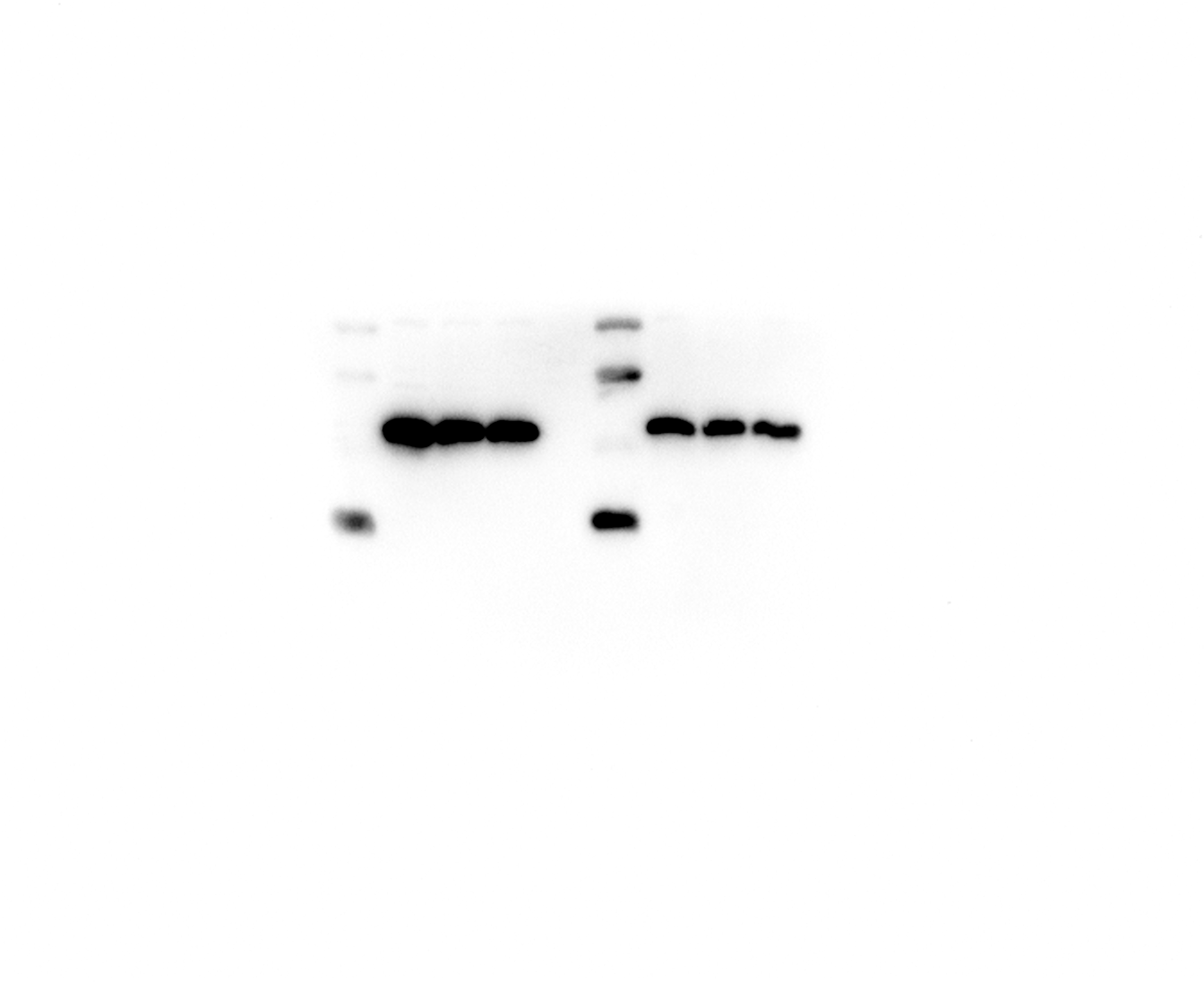

Supplement: Supplementary file 3 — WB Raw data [file 41420_2025_2583_MOESM3_ESM.zip › Figure 6 Panel B/Left is Panel B H3/h3_5.Tif]

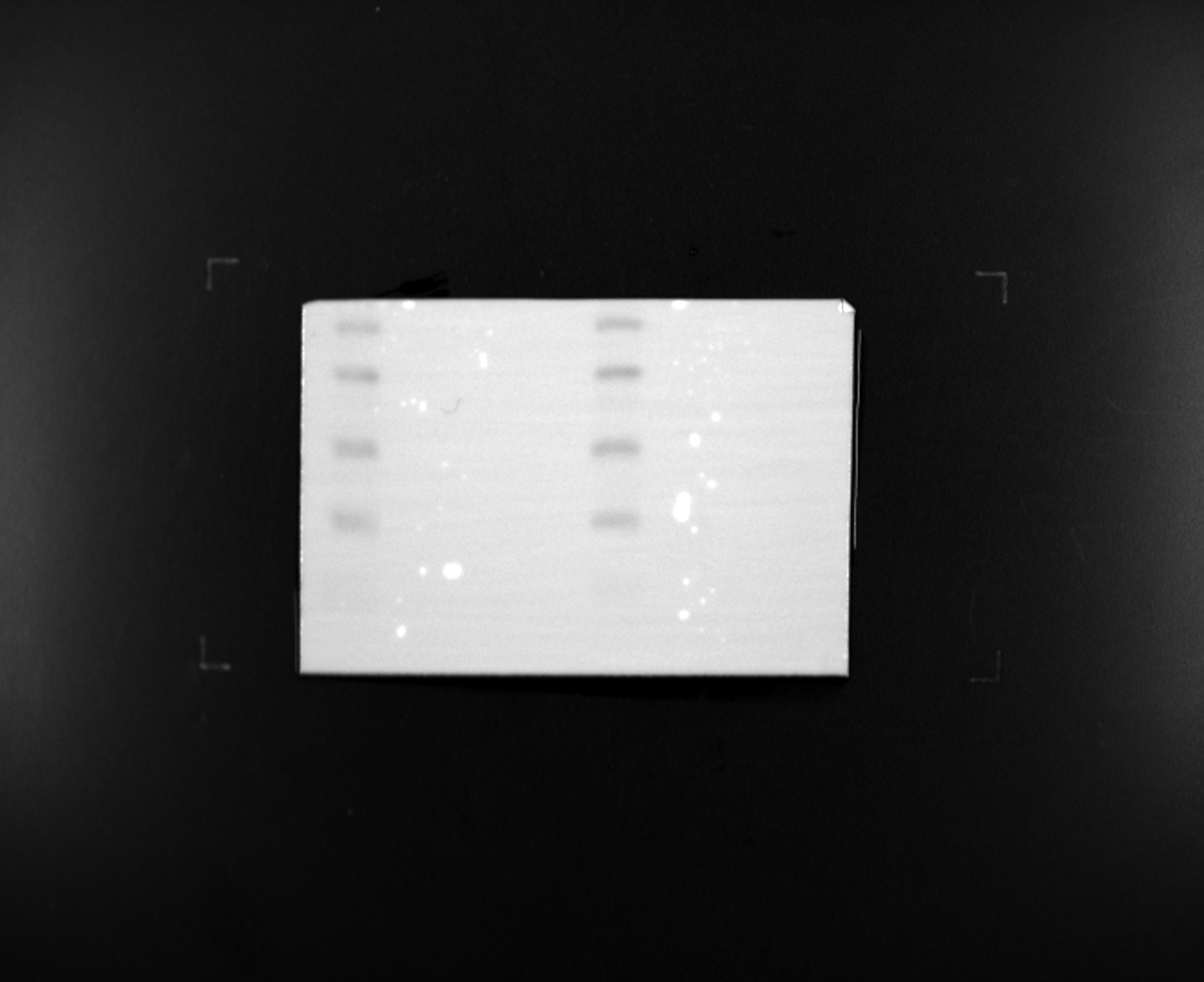

Supplement: Supplementary file 3 — WB Raw data [file 41420_2025_2583_MOESM3_ESM.zip › Figure 6 Panel B/Left is Panel B H3/h3_marker.Tif]

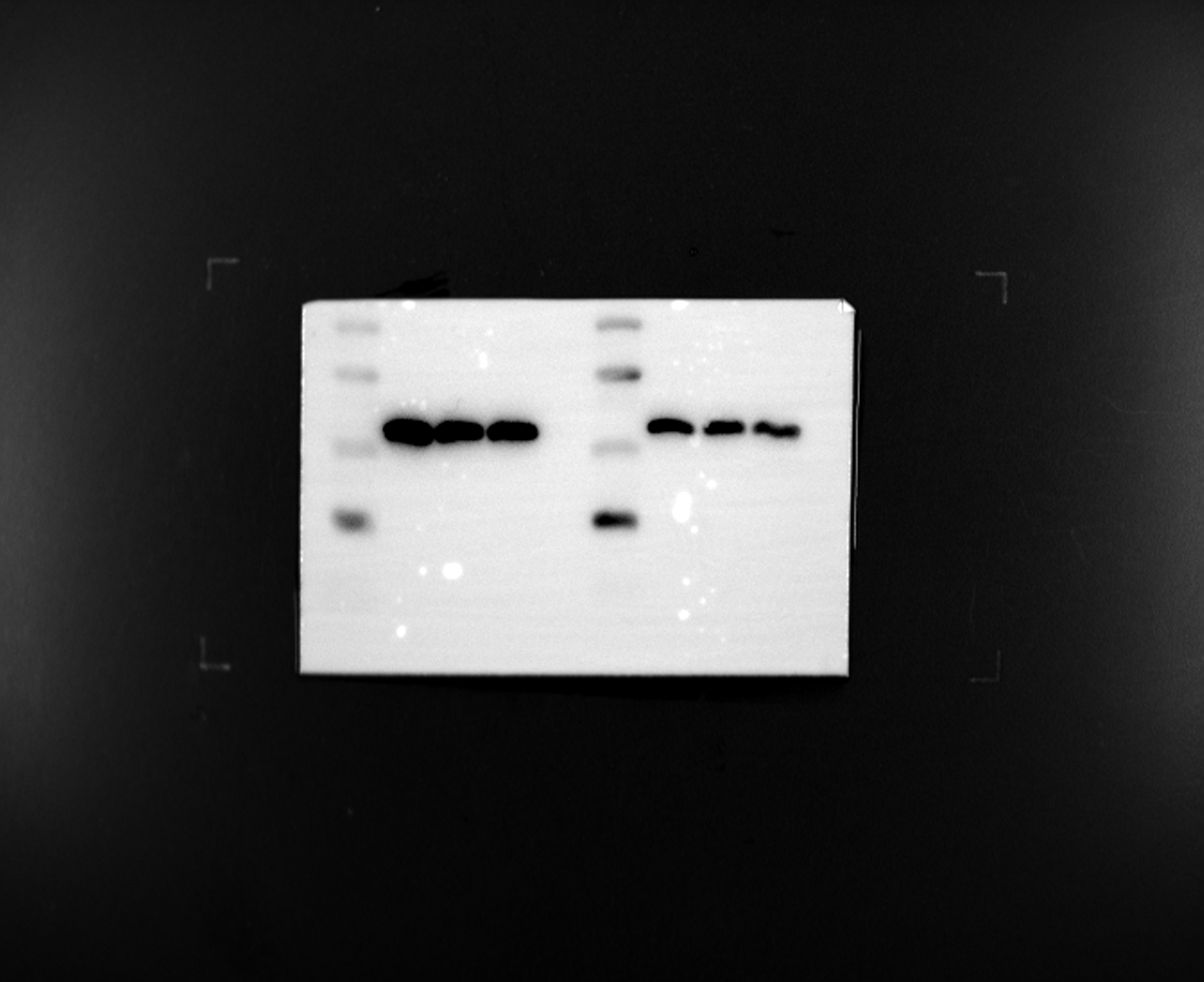

Supplement: Supplementary file 3 — WB Raw data [file 41420_2025_2583_MOESM3_ESM.zip › Figure 6 Panel B/Left is Panel B H3/h3_marker1.Tif]

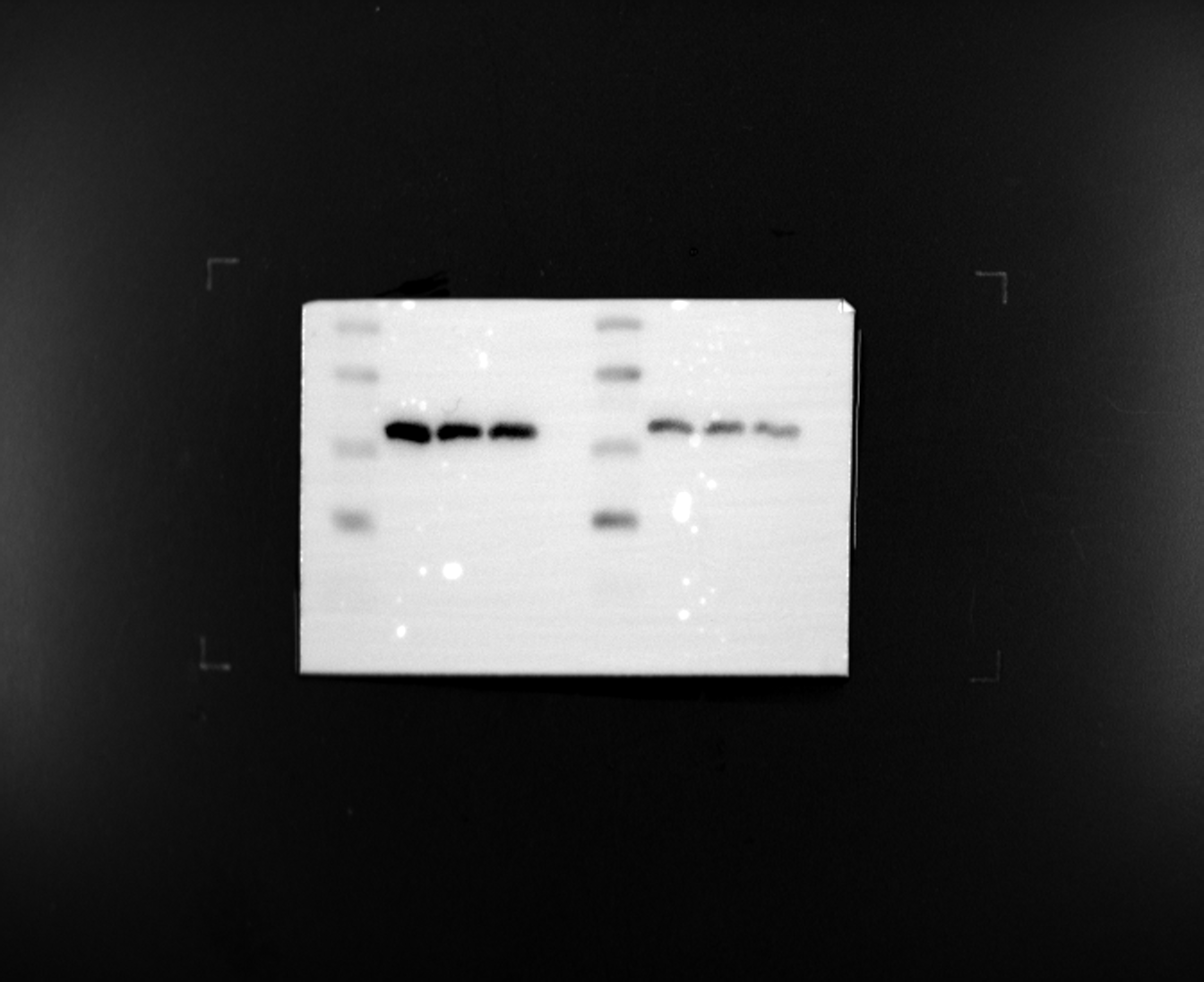

Supplement: Supplementary file 3 — WB Raw data [file 41420_2025_2583_MOESM3_ESM.zip › Figure 6 Panel B/Left is Panel B H3/h3_marker2.Tif]

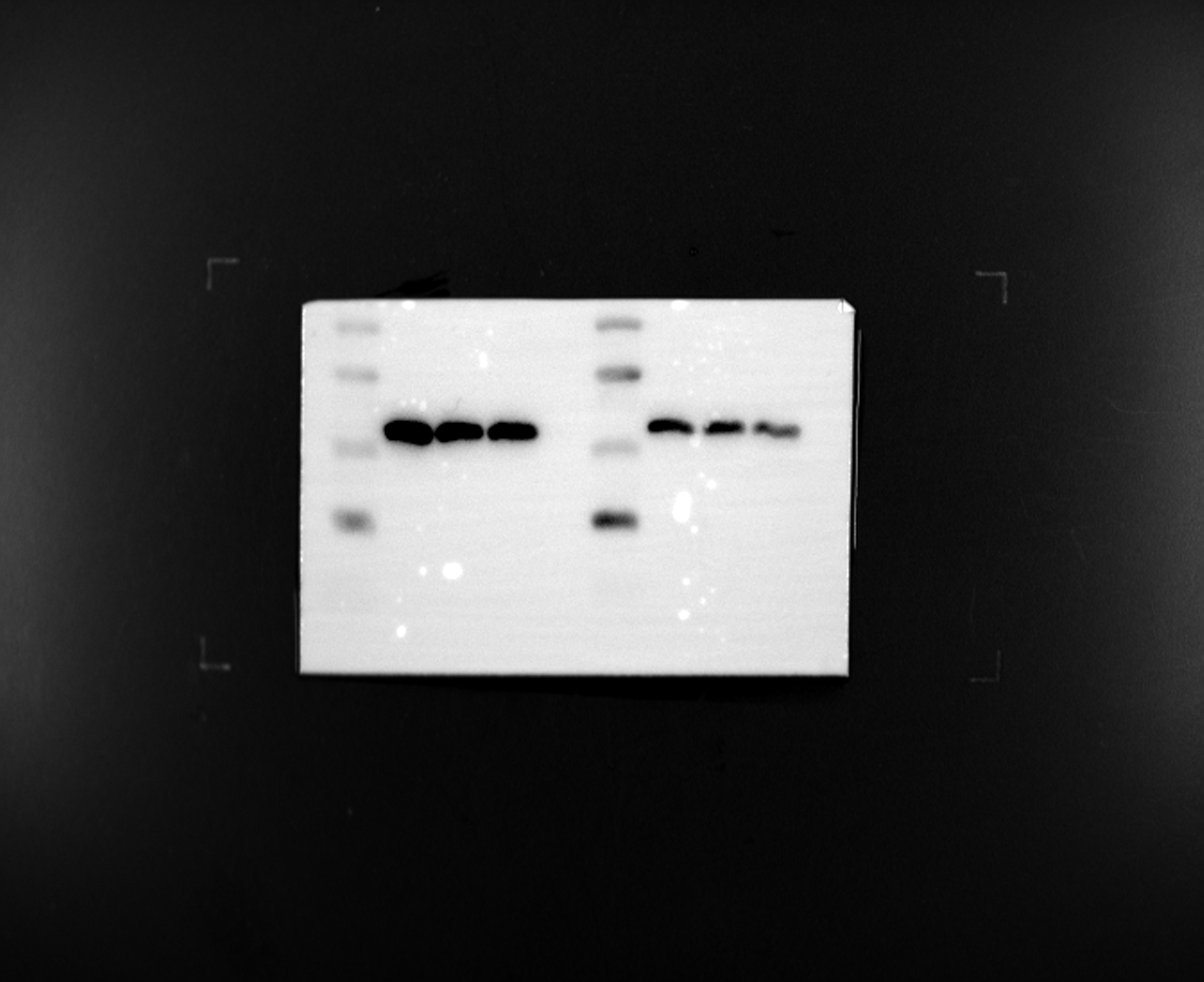

Supplement: Supplementary file 3 — WB Raw data [file 41420_2025_2583_MOESM3_ESM.zip › Figure 6 Panel B/Left is Panel B H3/h3_marker3.Tif]

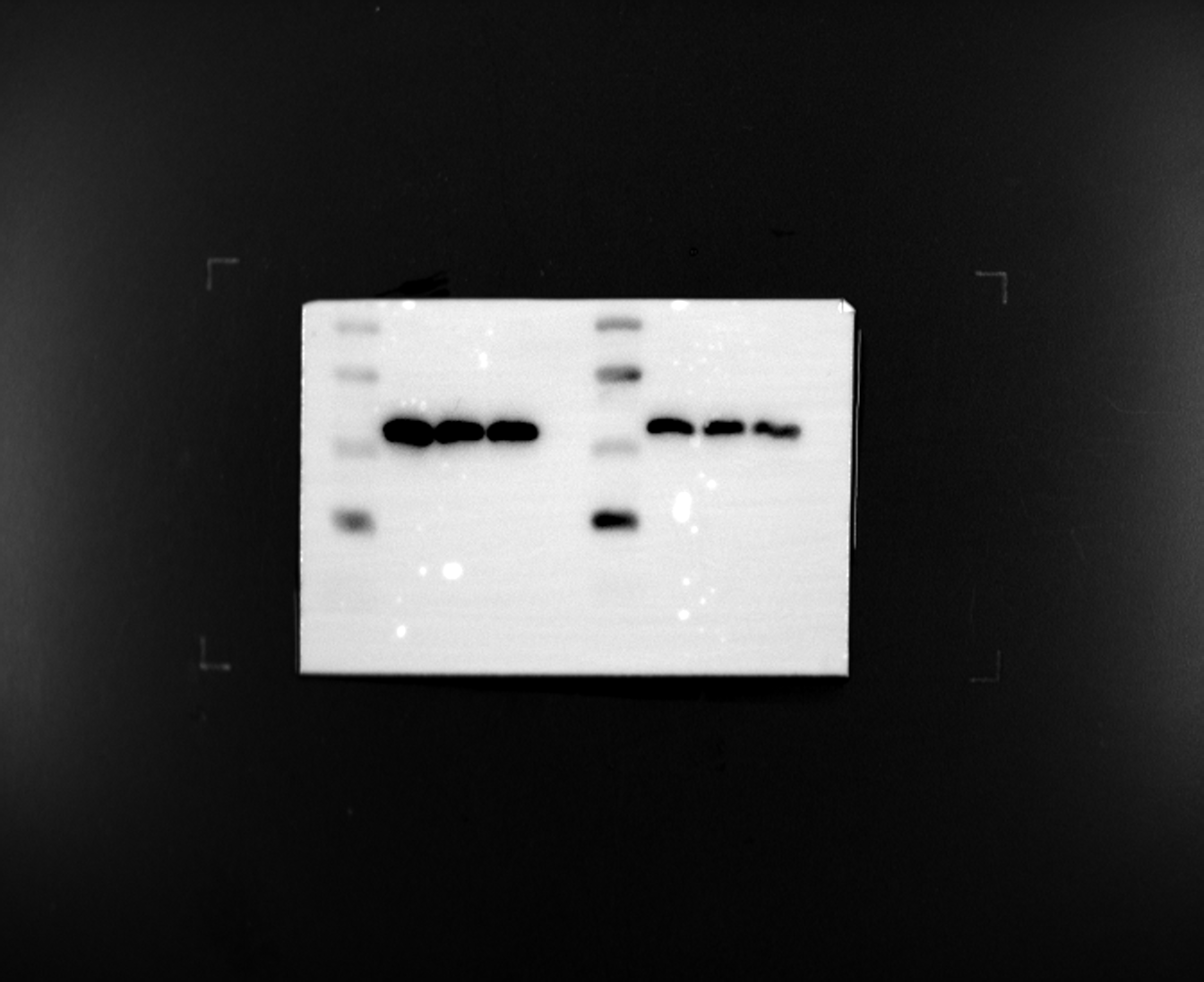

Supplement: Supplementary file 3 — WB Raw data [file 41420_2025_2583_MOESM3_ESM.zip › Figure 6 Panel B/Left is Panel B H3/h3_marker4.Tif]

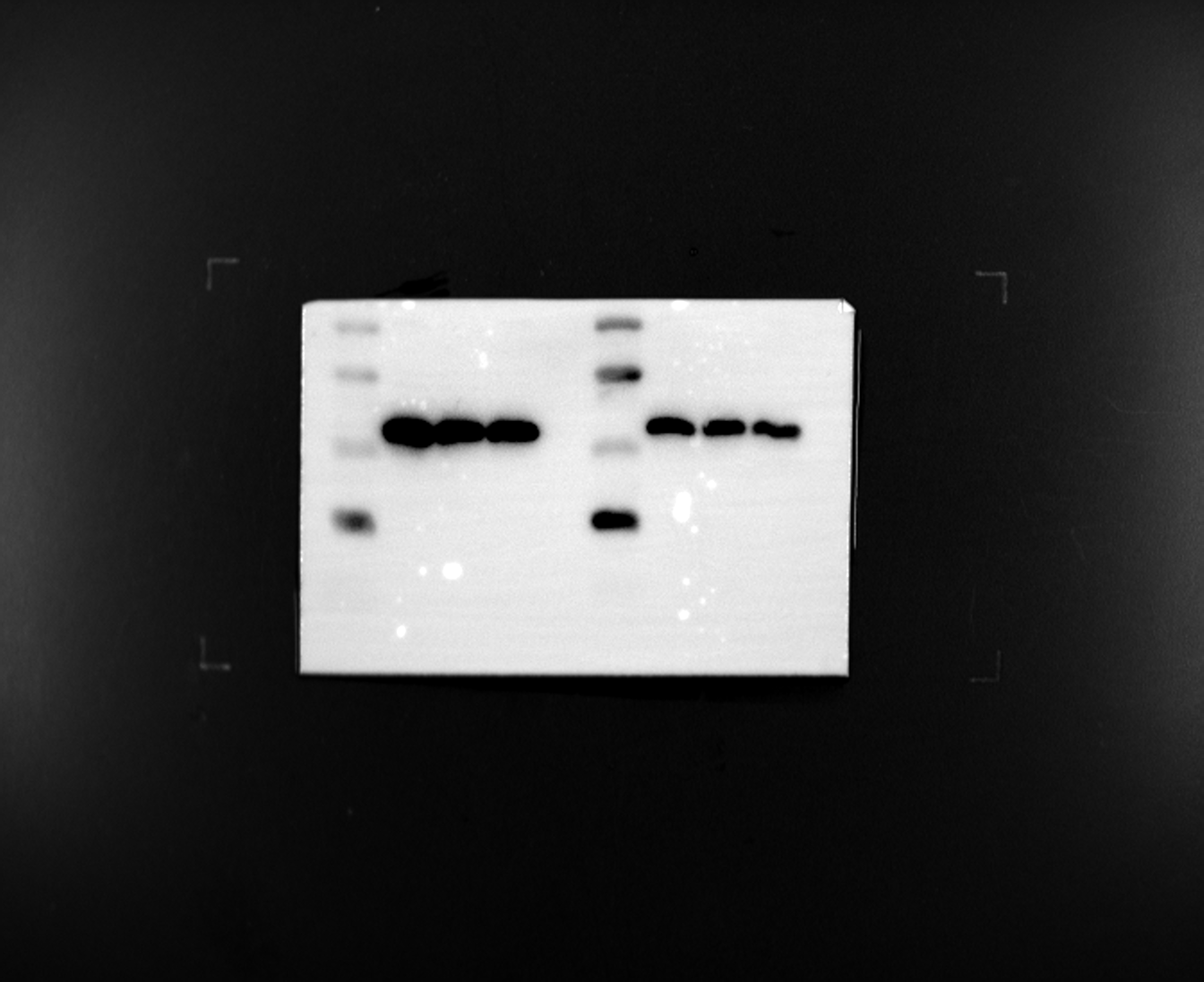

Supplement: Supplementary file 3 — WB Raw data [file 41420_2025_2583_MOESM3_ESM.zip › Figure 6 Panel B/Left is Panel B H3/h3_marker5.Tif]

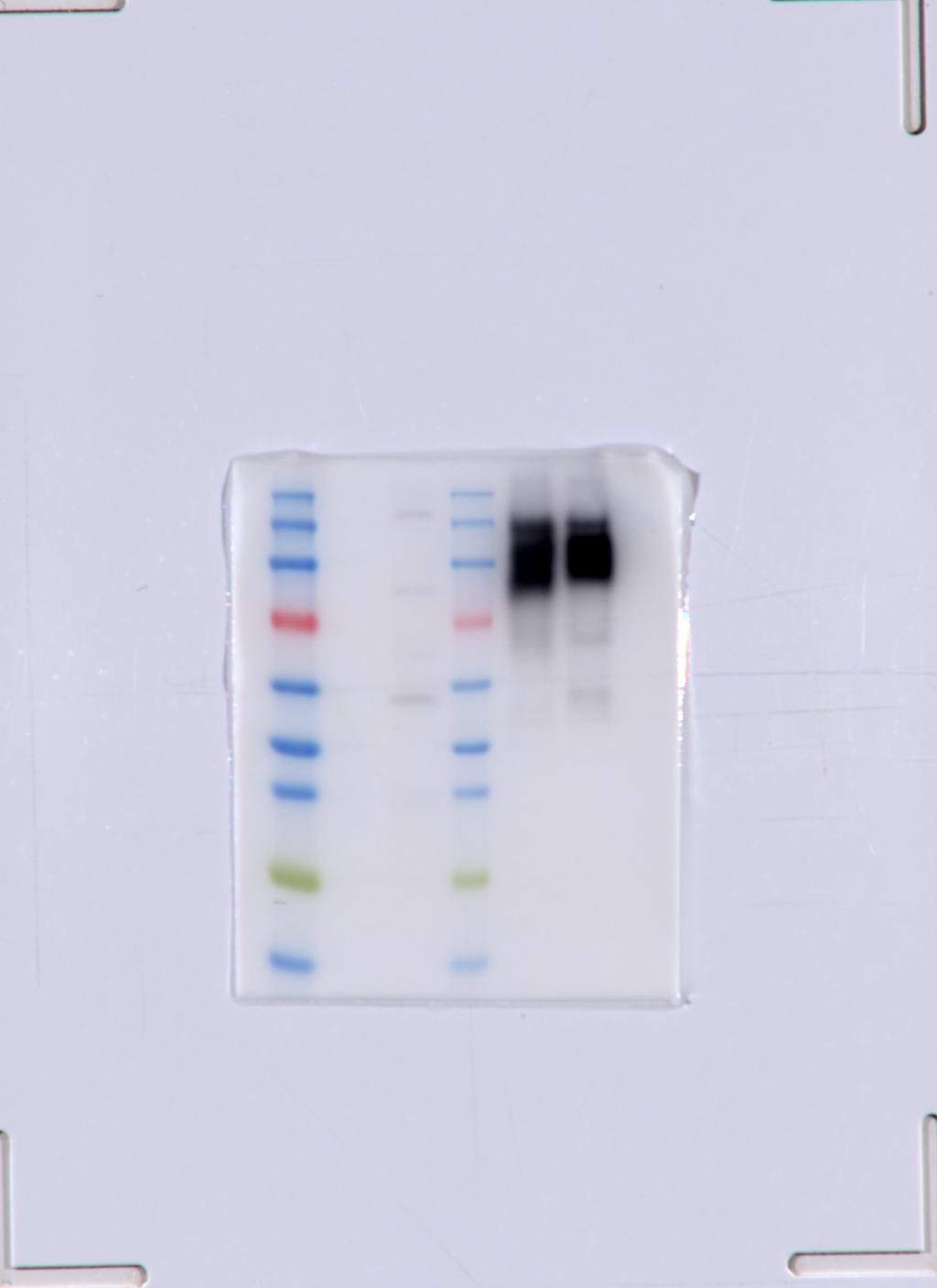

Supplement: Supplementary file 3 — WB Raw data [file 41420_2025_2583_MOESM3_ESM.zip › Figure 6 Panel D/Dcaf13 he 2022.04.03_11.05.33_Ch/Dcaf13 he 2022.04.03_11.05.33_Ch+Marker.jpg]

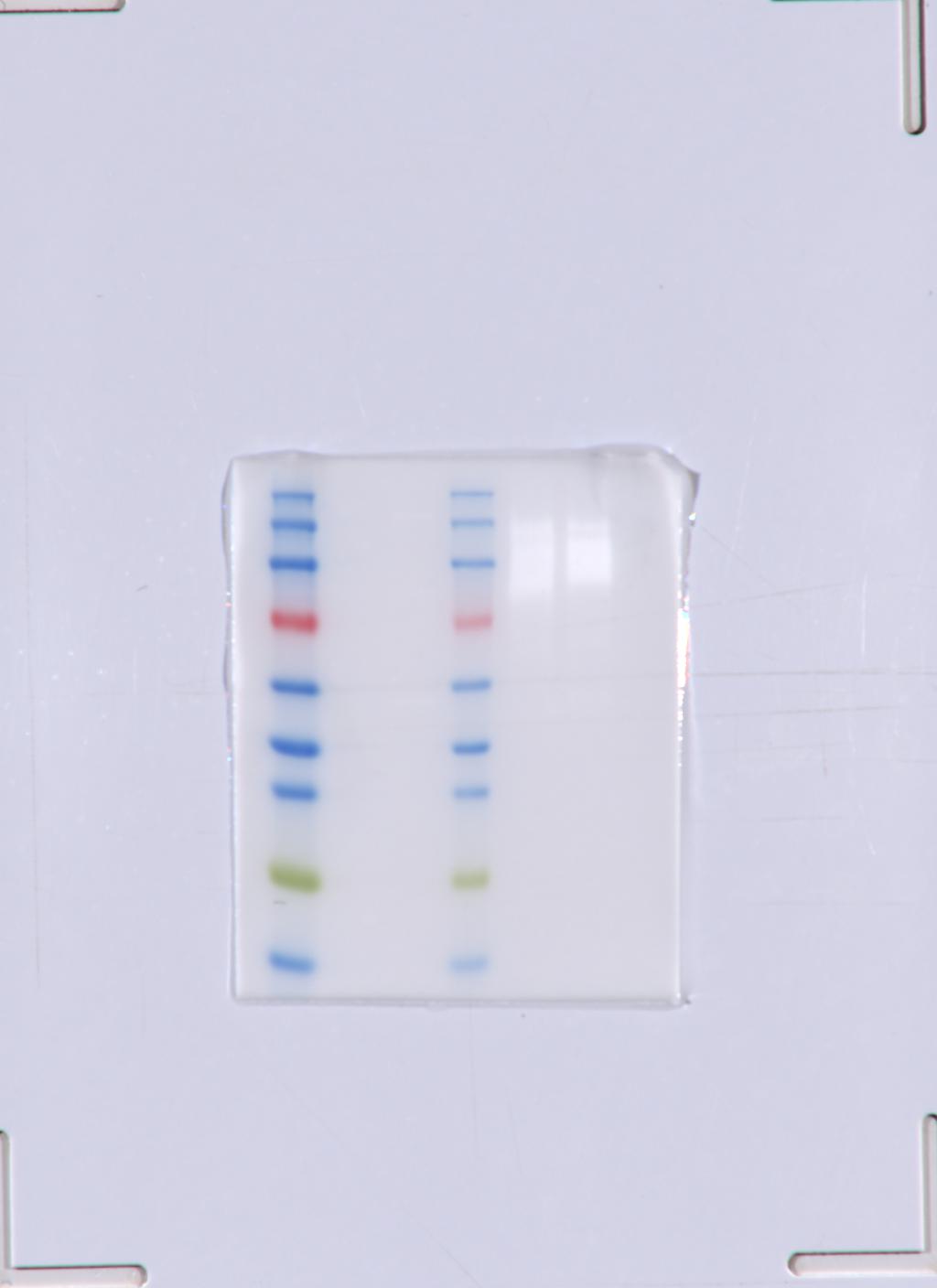

Supplement: Supplementary file 3 — WB Raw data [file 41420_2025_2583_MOESM3_ESM.zip › Figure 6 Panel D/Dcaf13 he 2022.04.03_11.05.33_Ch/Dcaf13 he 2022.04.03_11.05.33_Ch-Marker.jpg]

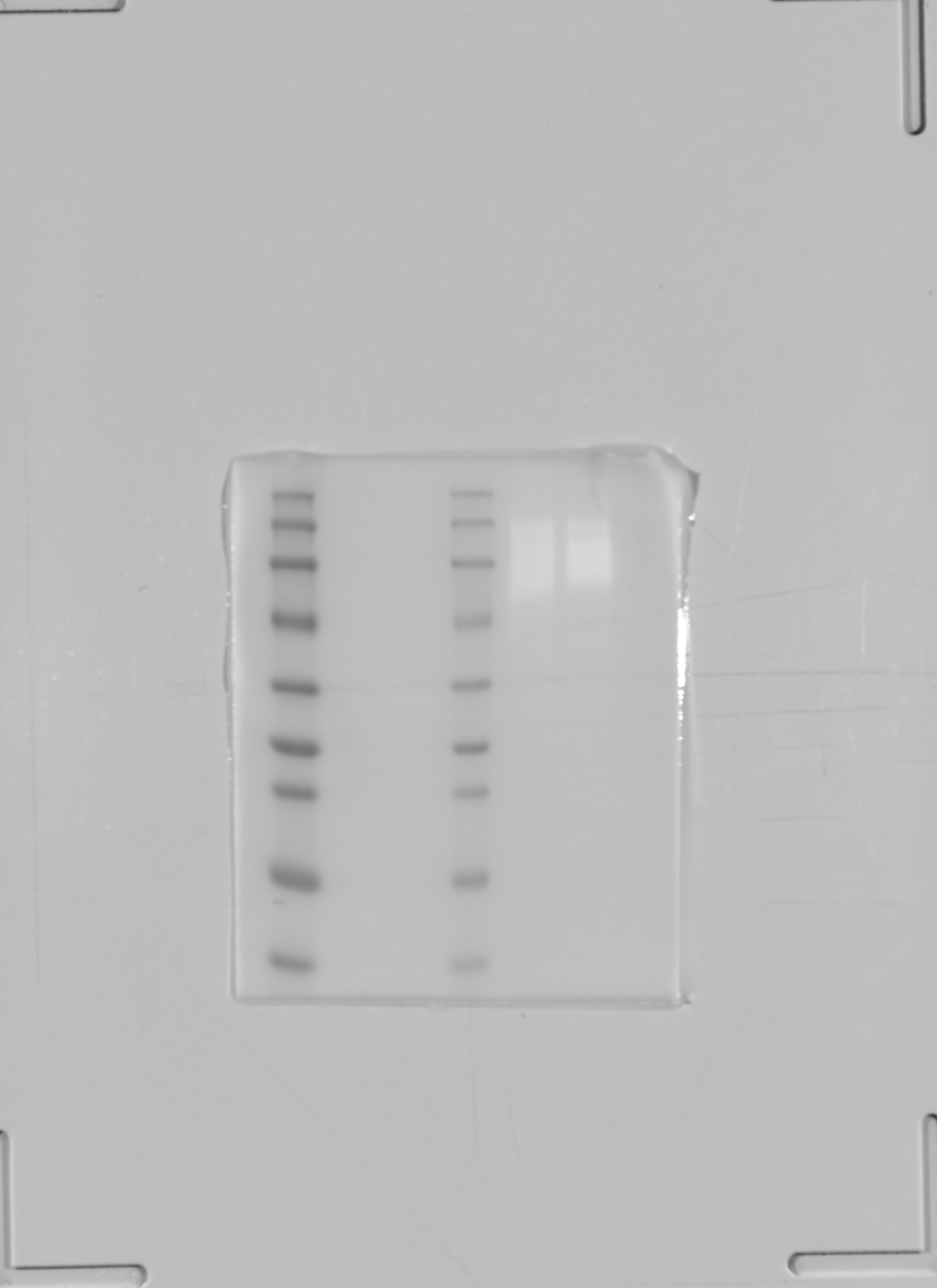

Supplement: Supplementary file 3 — WB Raw data [file 41420_2025_2583_MOESM3_ESM.zip › Figure 6 Panel D/Dcaf13 he 2022.04.03_11.05.33_Ch/Dcaf13 he 2022.04.03_11.05.33_Ch-Marker.tif]

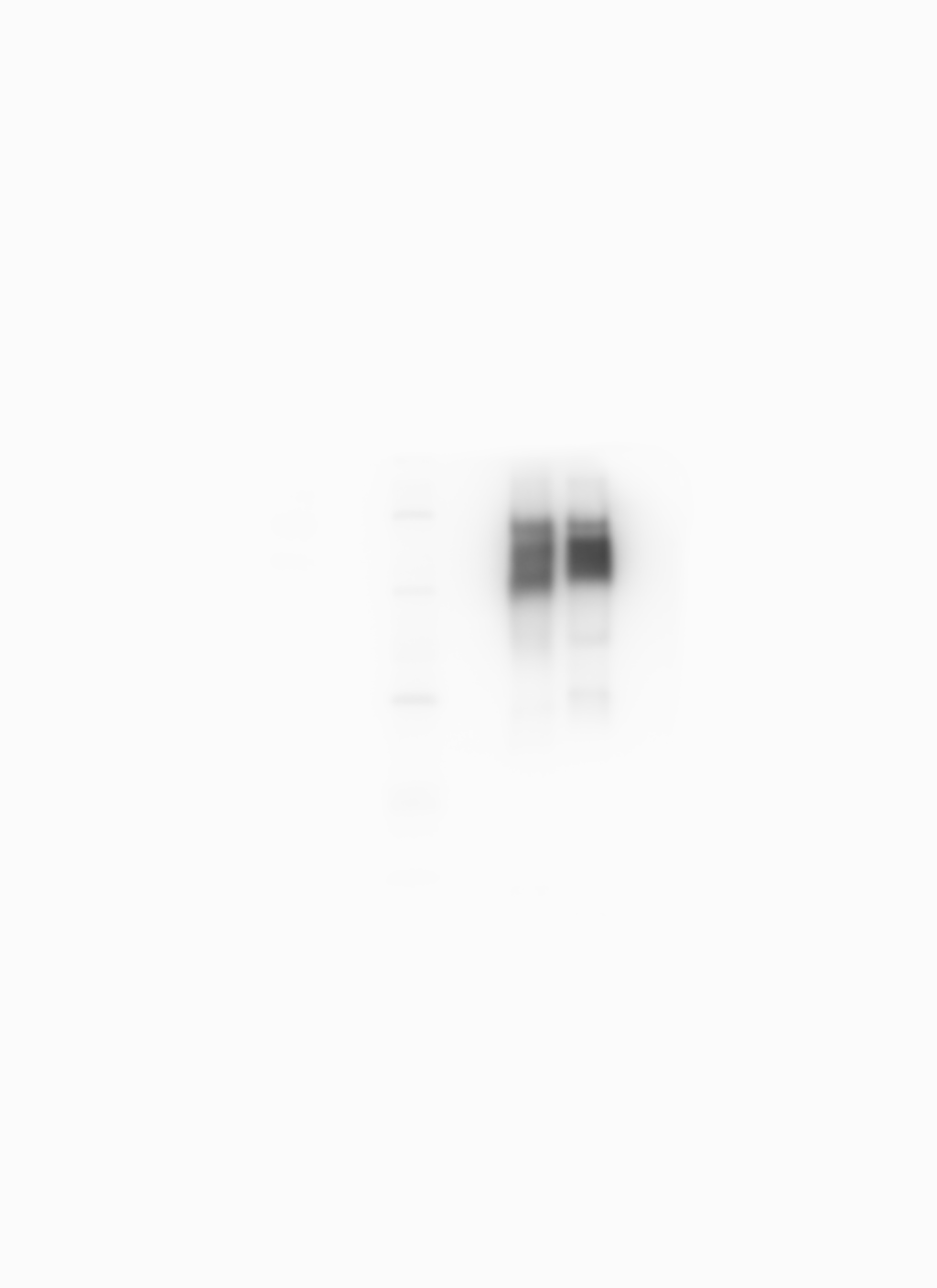

Supplement: Supplementary file 3 — WB Raw data [file 41420_2025_2583_MOESM3_ESM.zip › Figure 6 Panel D/Dcaf13 he 2022.04.03_11.05.33_Ch/Dcaf13 he 2022.04.03_11.05.33_Ch.tif]

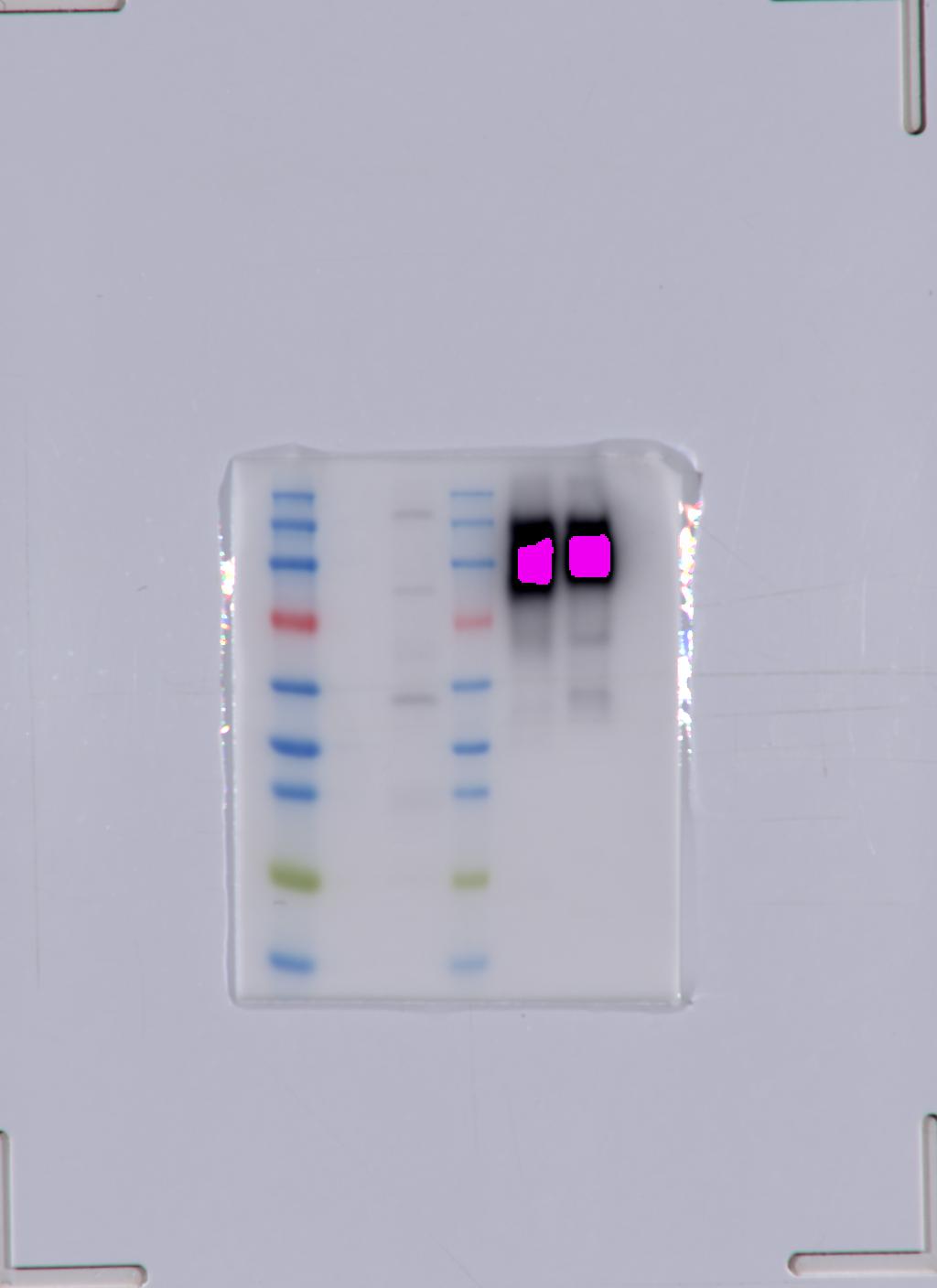

Supplement: Supplementary file 3 — WB Raw data [file 41420_2025_2583_MOESM3_ESM.zip › Figure 6 Panel D/Dcaf13 he 2022.04.03_11.07.39_Ch/Dcaf13 he 2022.04.03_11.07.39_Ch+Marker.jpg]

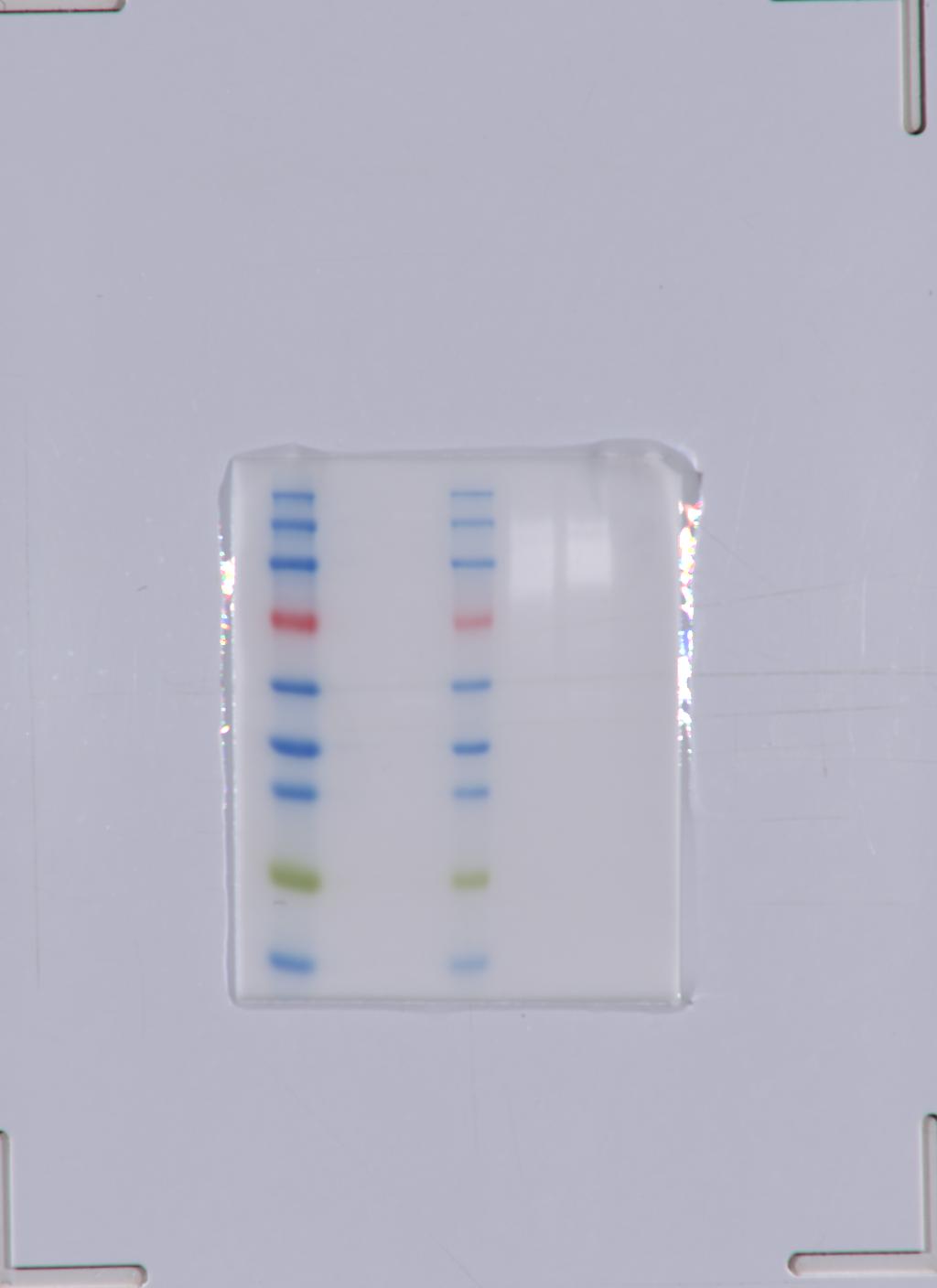

Supplement: Supplementary file 3 — WB Raw data [file 41420_2025_2583_MOESM3_ESM.zip › Figure 6 Panel D/Dcaf13 he 2022.04.03_11.07.39_Ch/Dcaf13 he 2022.04.03_11.07.39_Ch-Marker.jpg]

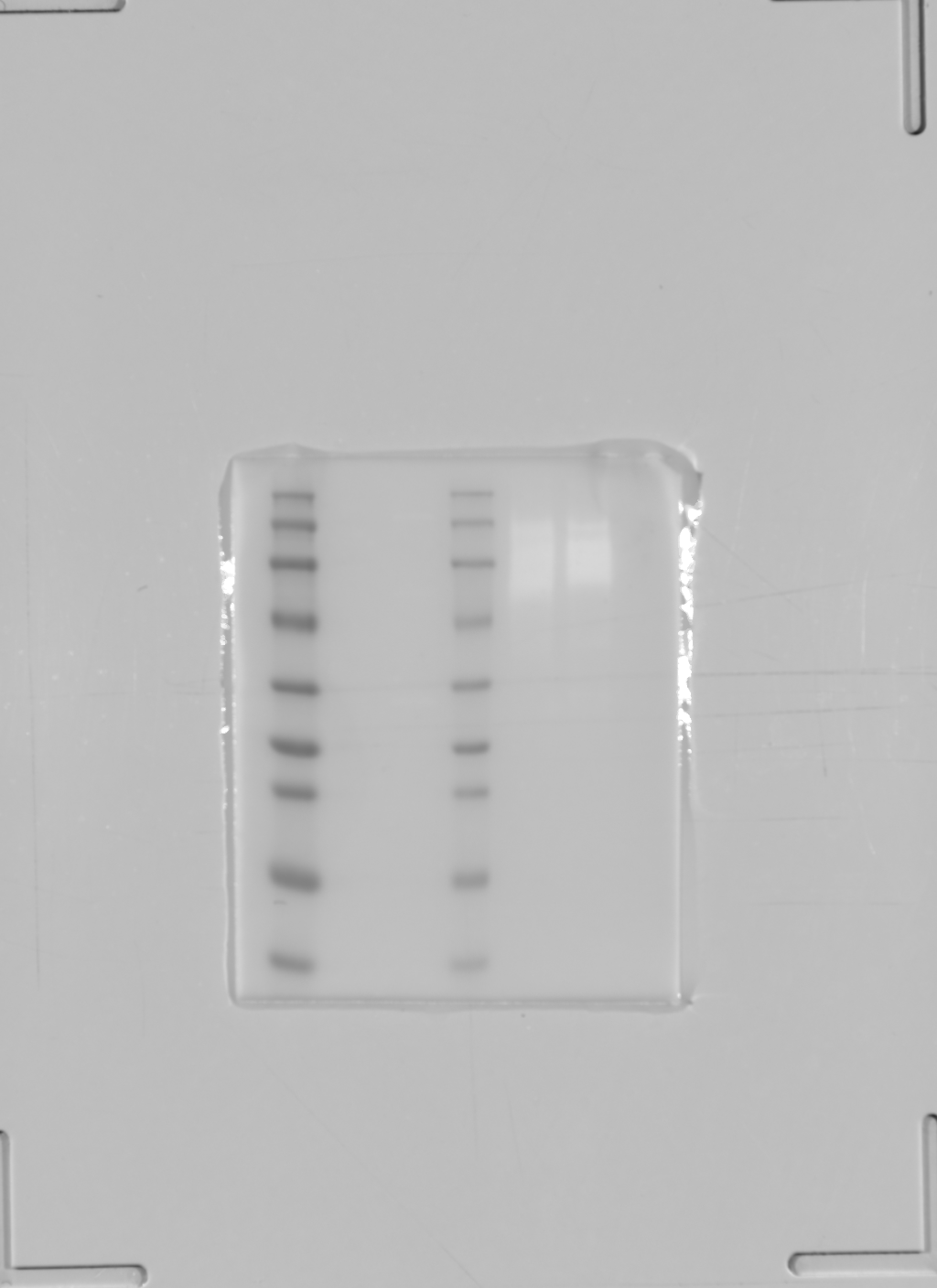

Supplement: Supplementary file 3 — WB Raw data [file 41420_2025_2583_MOESM3_ESM.zip › Figure 6 Panel D/Dcaf13 he 2022.04.03_11.07.39_Ch/Dcaf13 he 2022.04.03_11.07.39_Ch-Marker.tif]

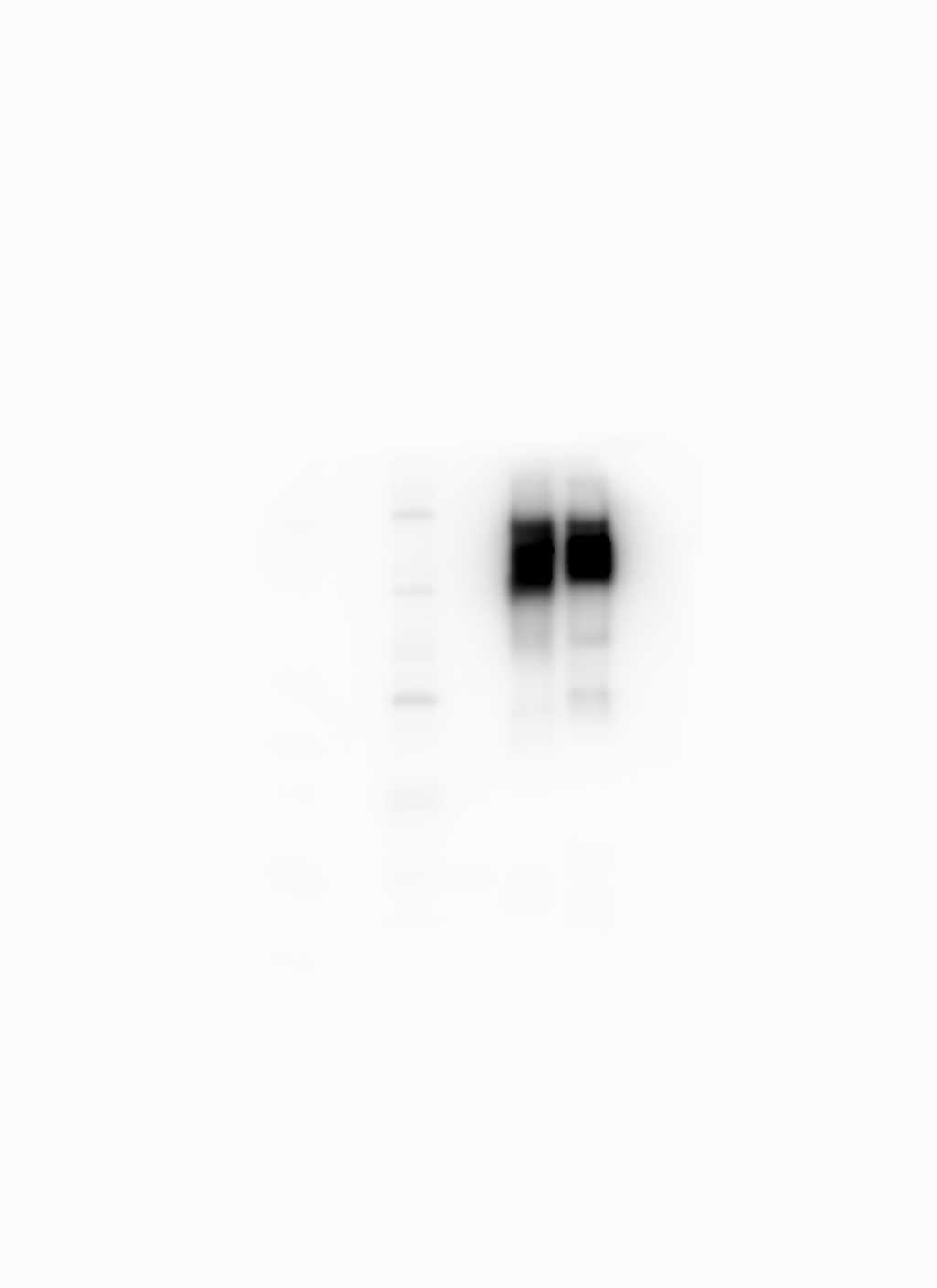

Supplement: Supplementary file 3 — WB Raw data [file 41420_2025_2583_MOESM3_ESM.zip › Figure 6 Panel D/Dcaf13 he 2022.04.03_11.07.39_Ch/Dcaf13 he 2022.04.03_11.07.39_Ch.tif]

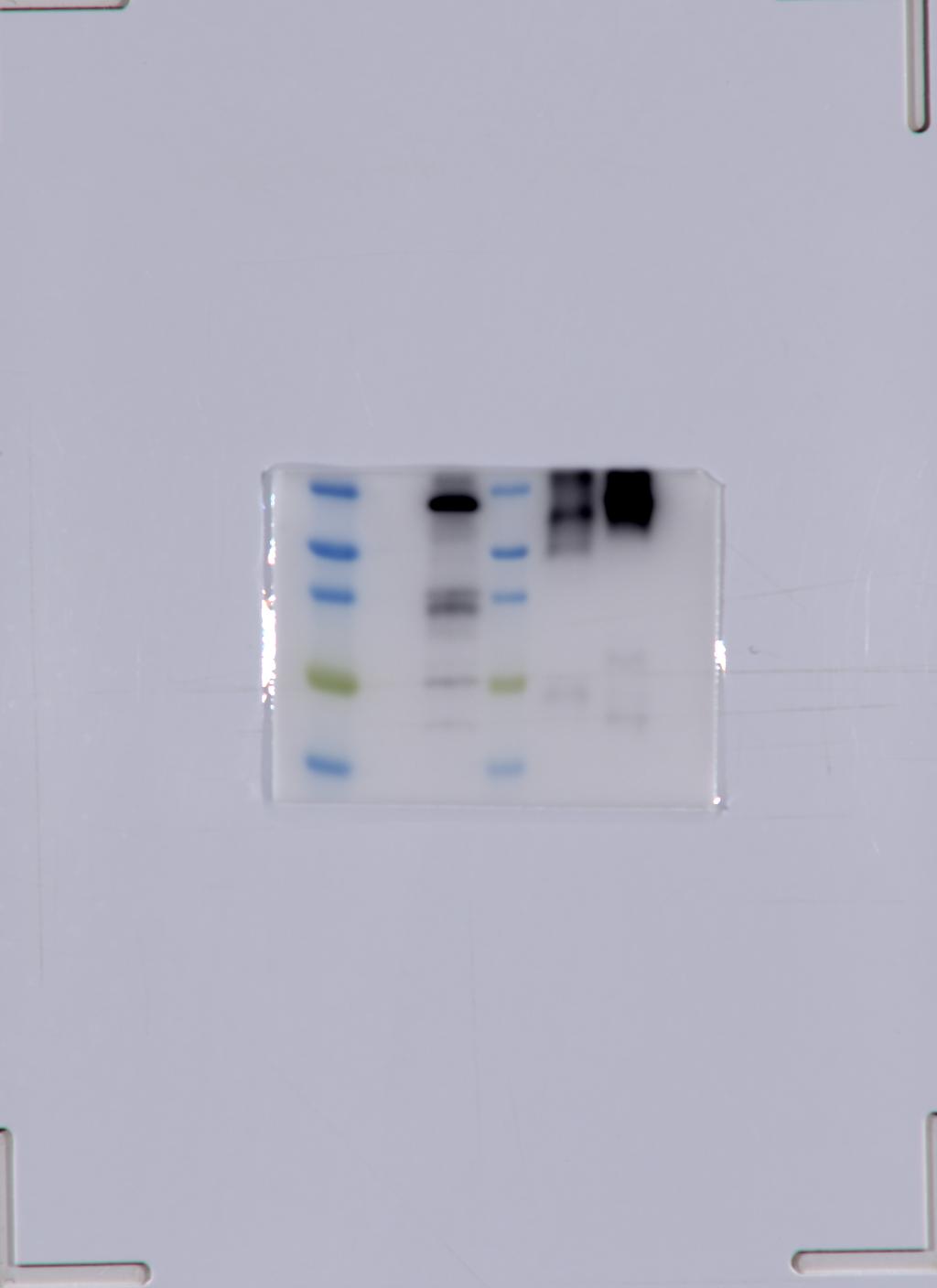

Supplement: Supplementary file 3 — WB Raw data [file 41420_2025_2583_MOESM3_ESM.zip › Figure 6 Panel D/Dcaf13 he 2022.04.03_11.10.44_Ch/Dcaf13 he 2022.04.03_11.10.44_Ch+Marker.jpg]

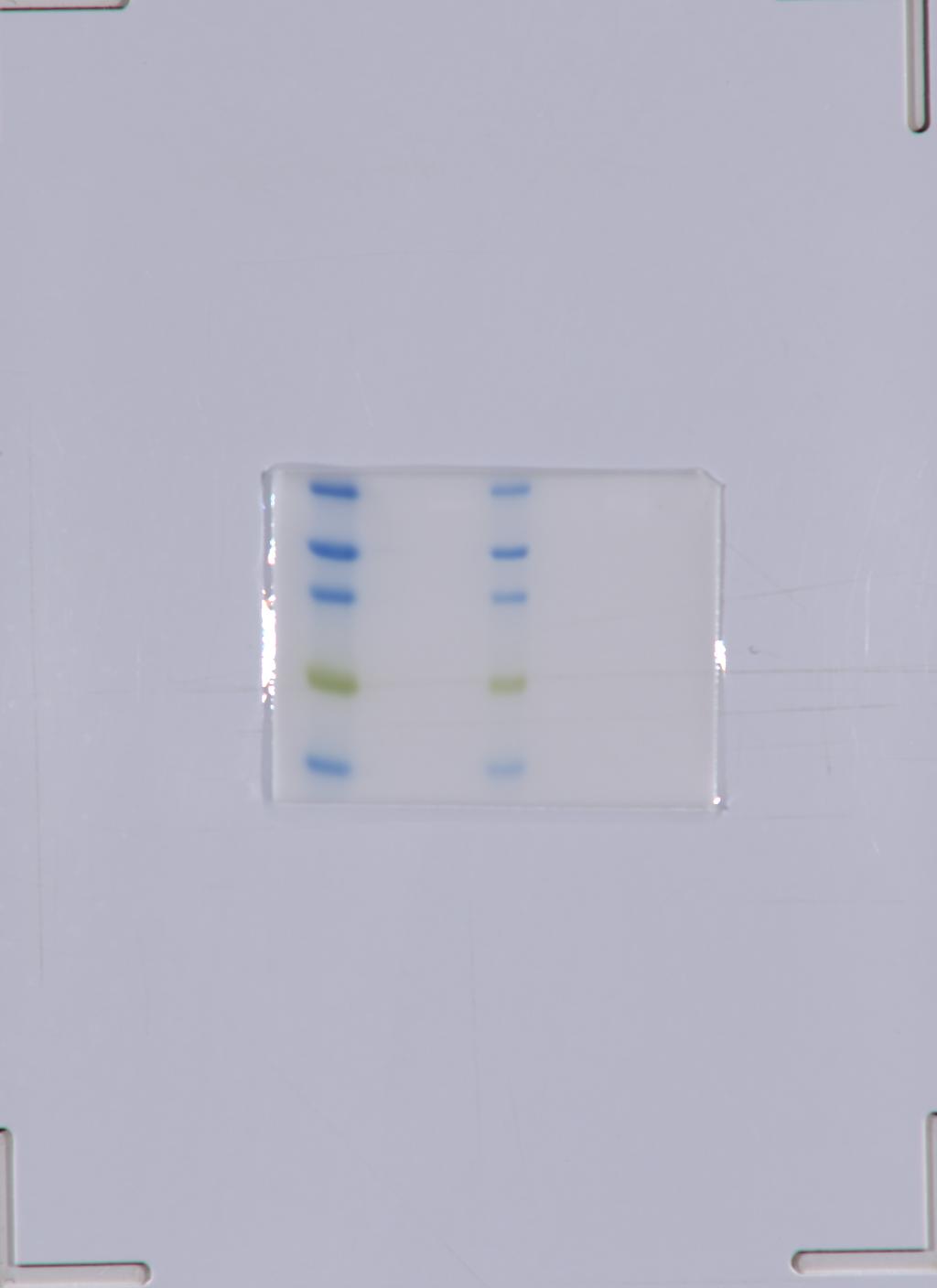

Supplement: Supplementary file 3 — WB Raw data [file 41420_2025_2583_MOESM3_ESM.zip › Figure 6 Panel D/Dcaf13 he 2022.04.03_11.10.44_Ch/Dcaf13 he 2022.04.03_11.10.44_Ch-Marker.jpg]

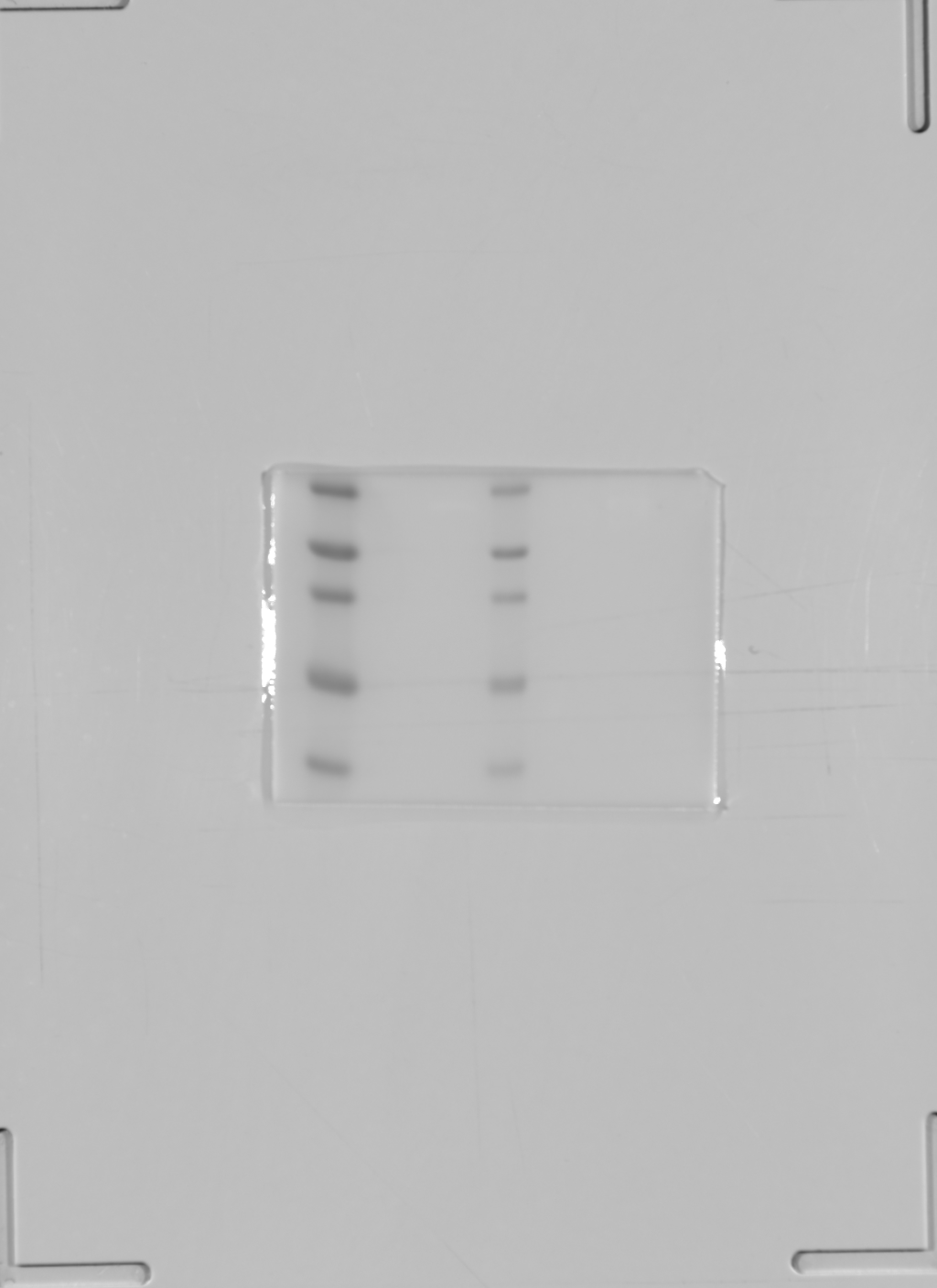

Supplement: Supplementary file 3 — WB Raw data [file 41420_2025_2583_MOESM3_ESM.zip › Figure 6 Panel D/Dcaf13 he 2022.04.03_11.10.44_Ch/Dcaf13 he 2022.04.03_11.10.44_Ch-Marker.tif]

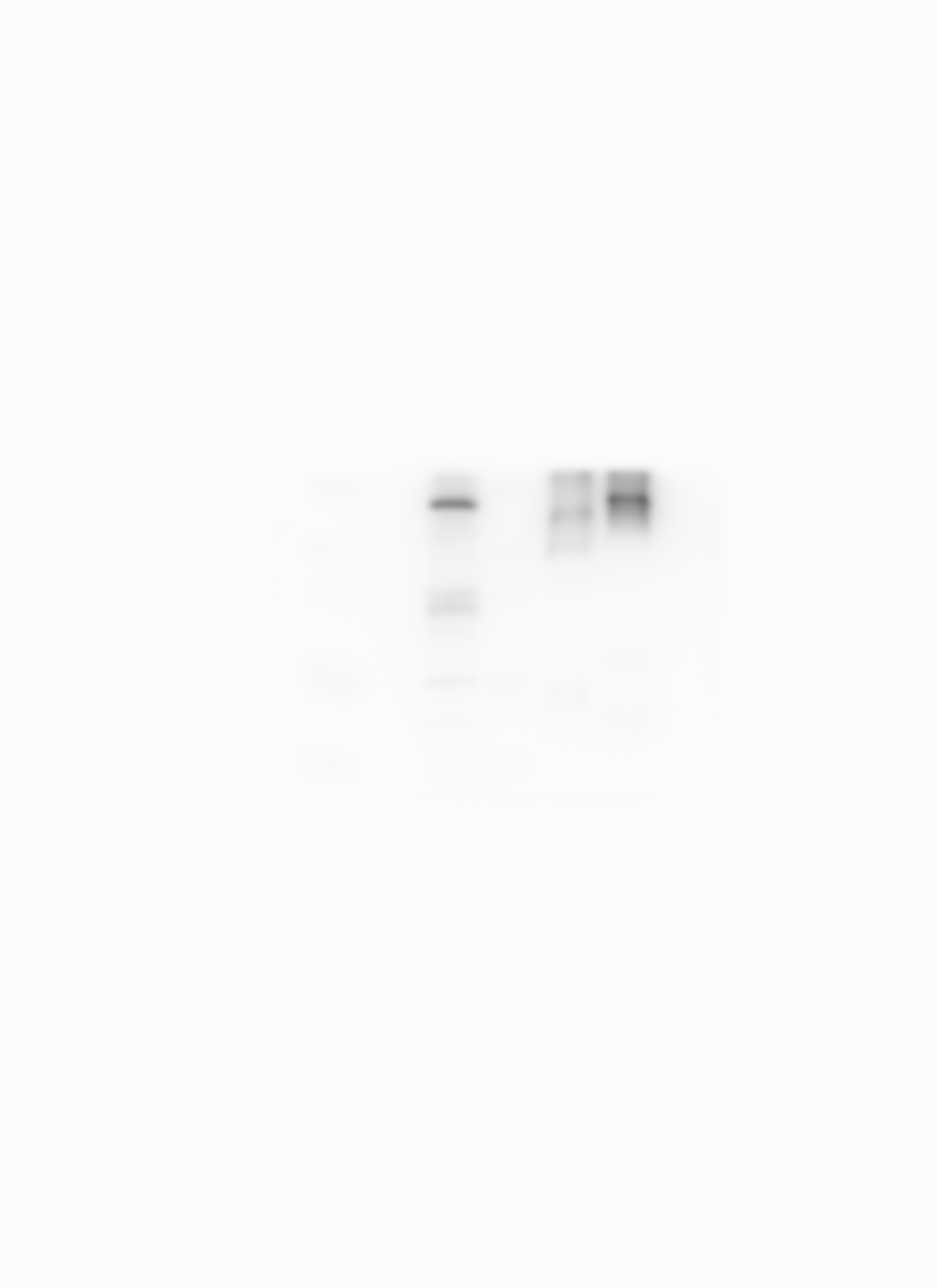

Supplement: Supplementary file 3 — WB Raw data [file 41420_2025_2583_MOESM3_ESM.zip › Figure 6 Panel D/Dcaf13 he 2022.04.03_11.10.44_Ch/Dcaf13 he 2022.04.03_11.10.44_Ch.tif]

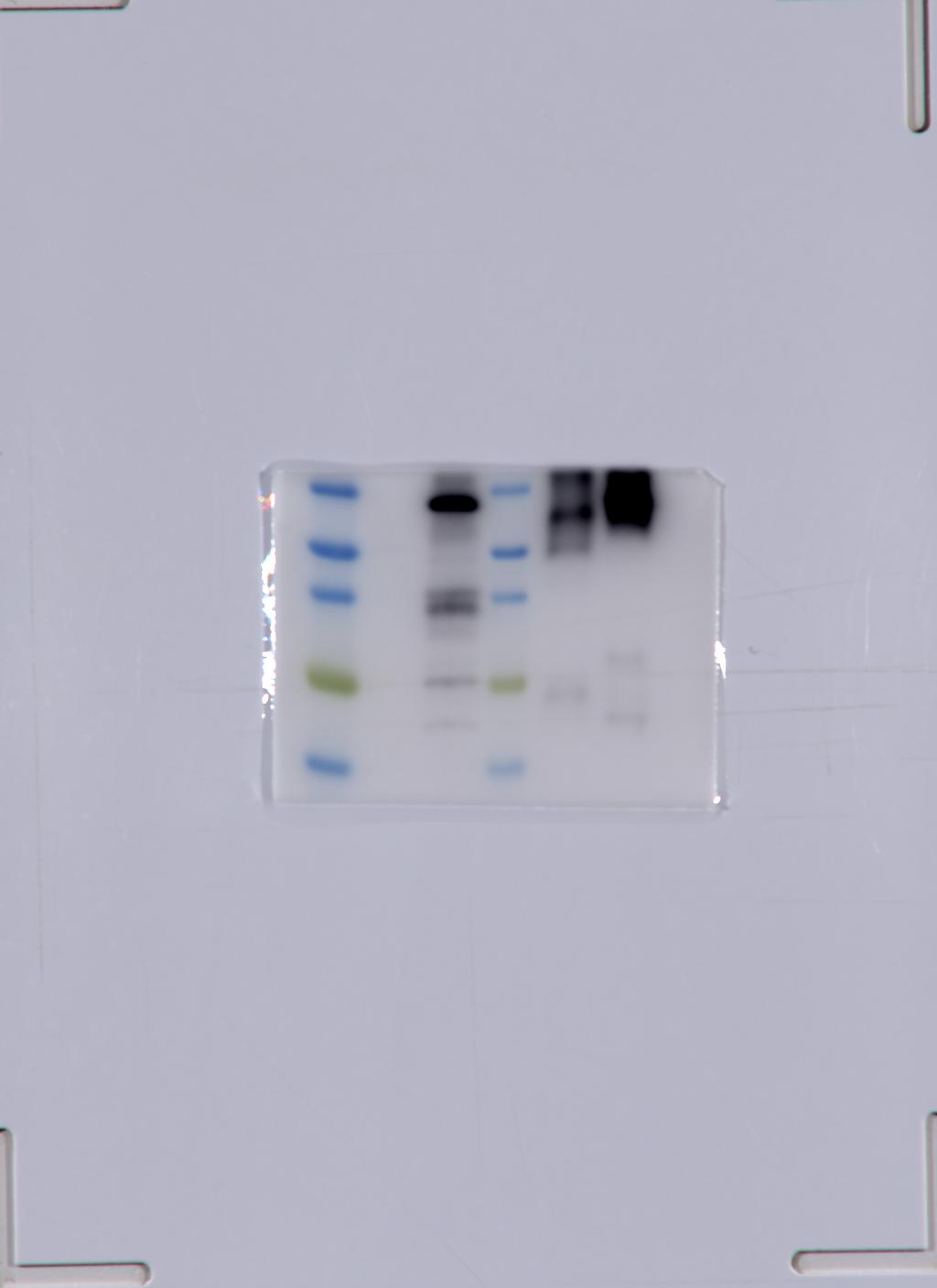

Supplement: Supplementary file 3 — WB Raw data [file 41420_2025_2583_MOESM3_ESM.zip › Figure 6 Panel D/Dcaf13 he 2022.04.03_11.11.52_Ch/Dcaf13 he 2022.04.03_11.11.52_Ch+Marker.jpg]

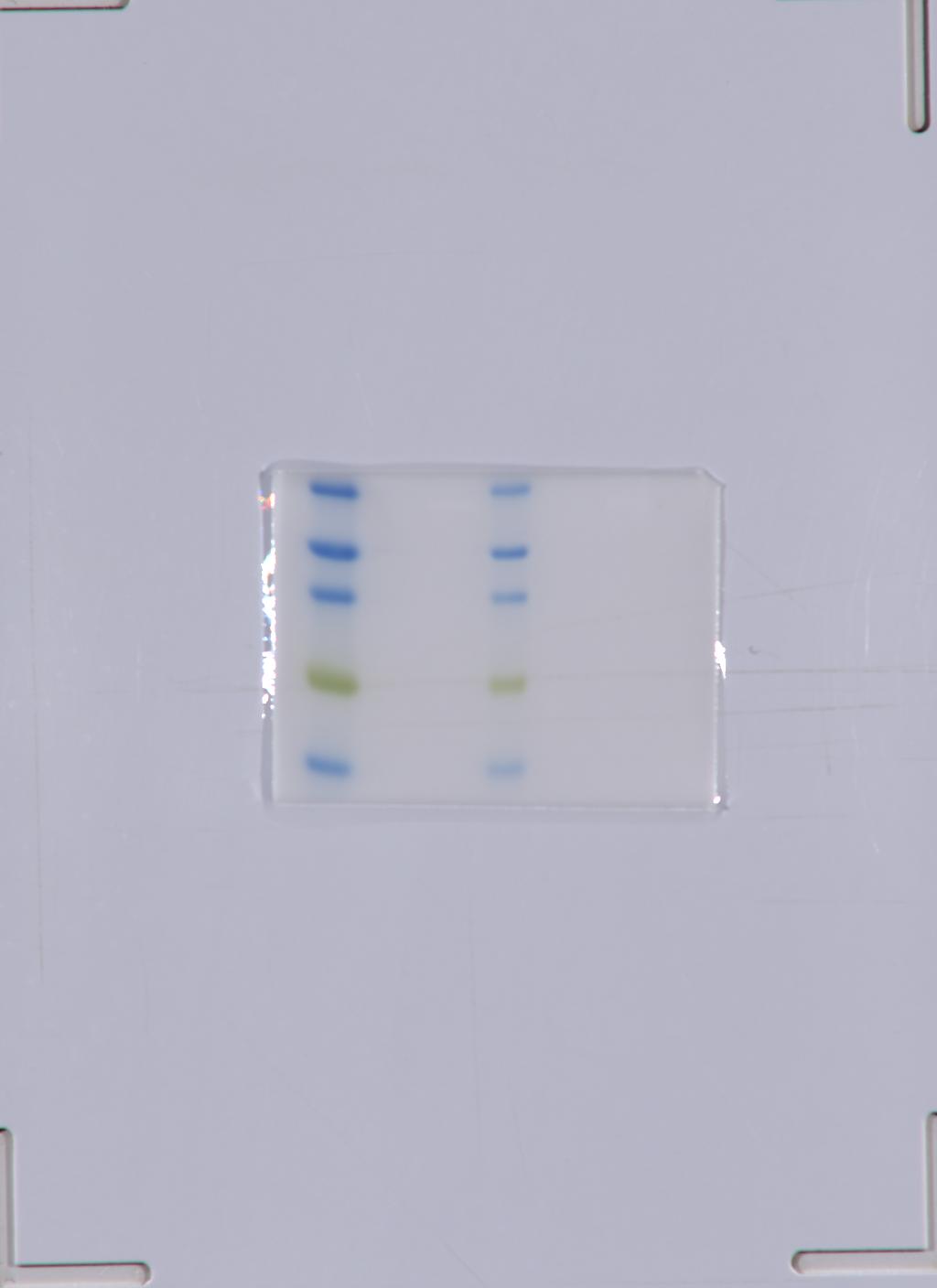

Supplement: Supplementary file 3 — WB Raw data [file 41420_2025_2583_MOESM3_ESM.zip › Figure 6 Panel D/Dcaf13 he 2022.04.03_11.11.52_Ch/Dcaf13 he 2022.04.03_11.11.52_Ch-Marker.jpg]

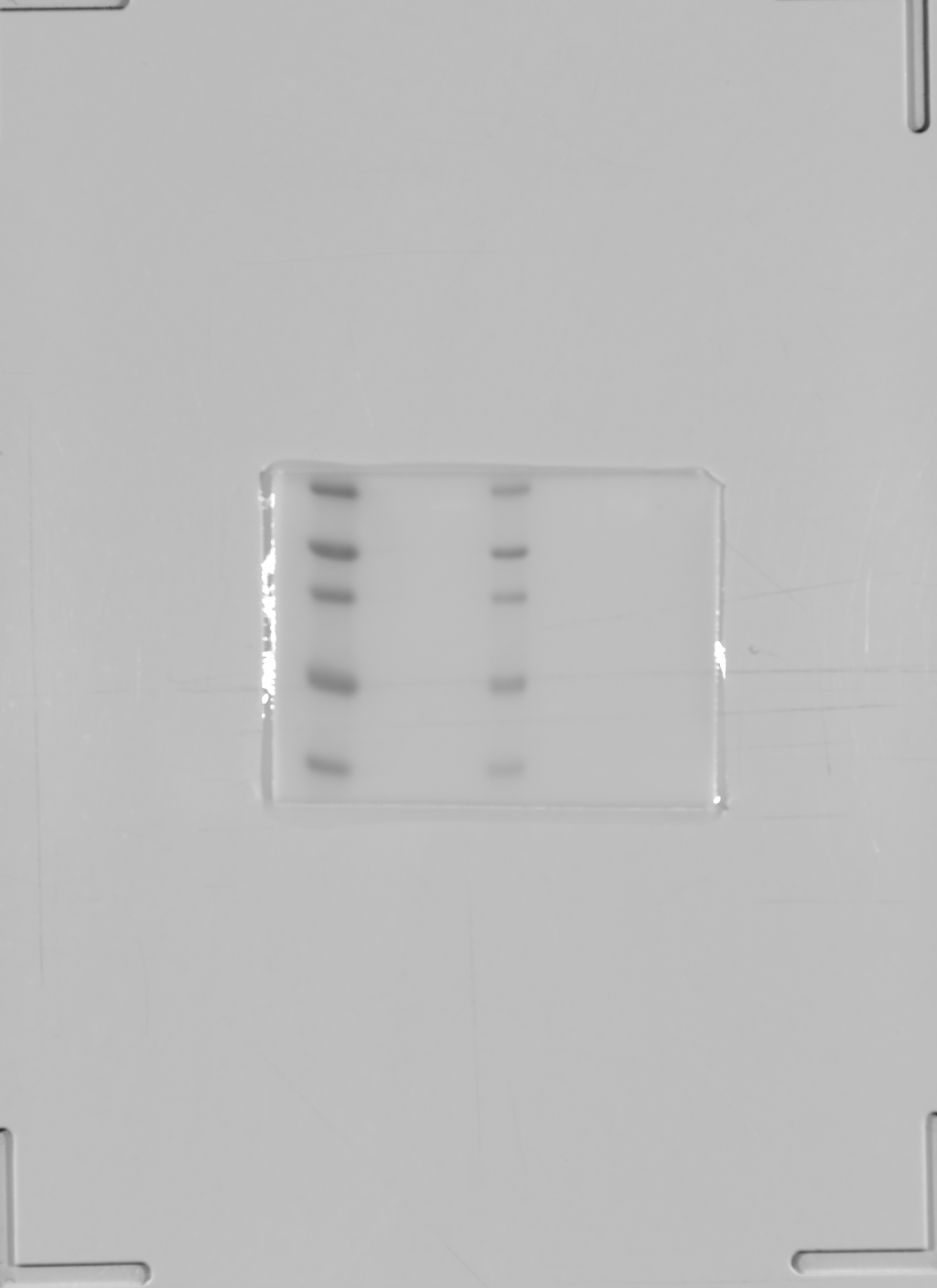

Supplement: Supplementary file 3 — WB Raw data [file 41420_2025_2583_MOESM3_ESM.zip › Figure 6 Panel D/Dcaf13 he 2022.04.03_11.11.52_Ch/Dcaf13 he 2022.04.03_11.11.52_Ch-Marker.tif]

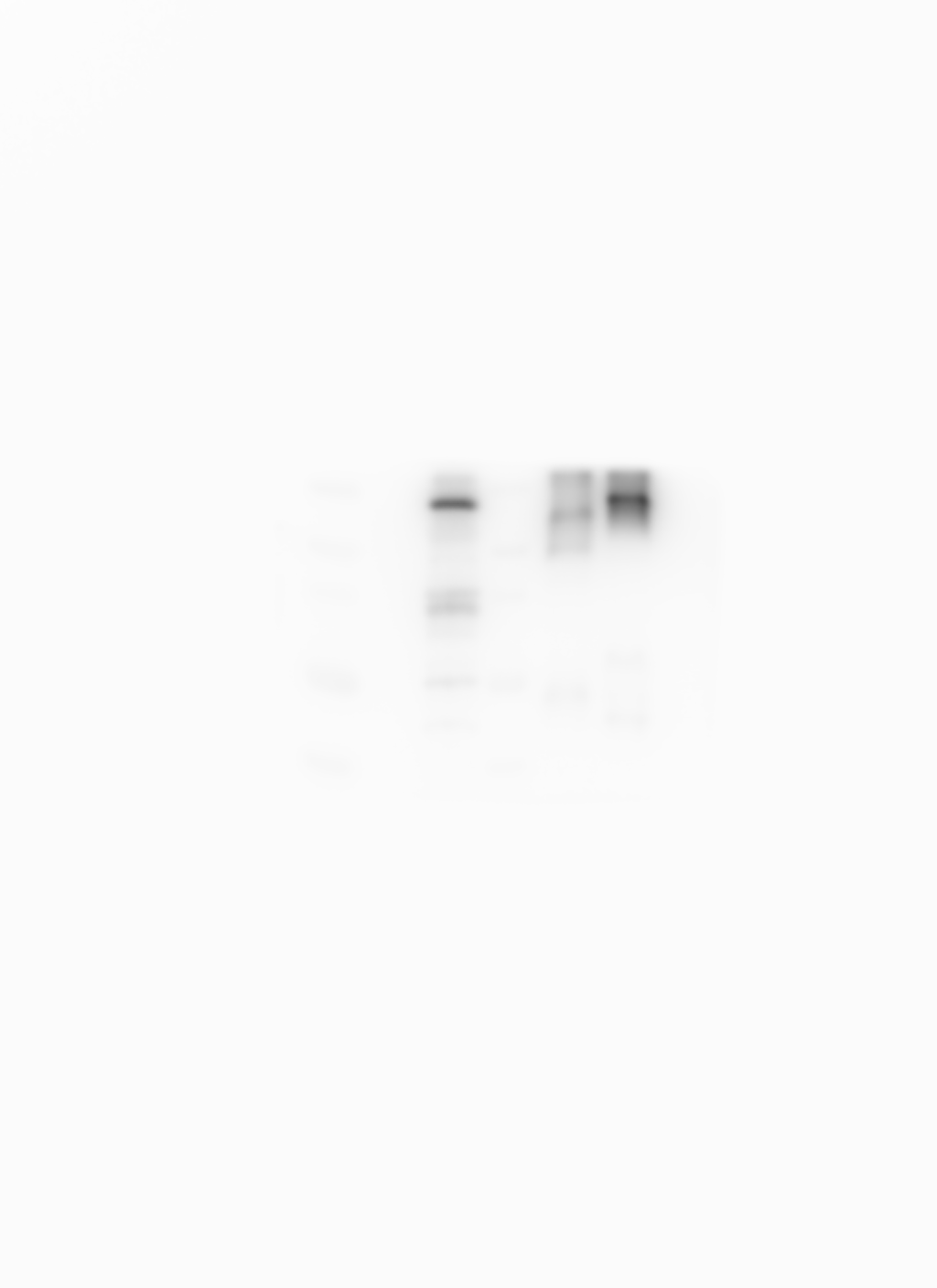

Supplement: Supplementary file 3 — WB Raw data [file 41420_2025_2583_MOESM3_ESM.zip › Figure 6 Panel D/Dcaf13 he 2022.04.03_11.11.52_Ch/Dcaf13 he 2022.04.03_11.11.52_Ch.tif]

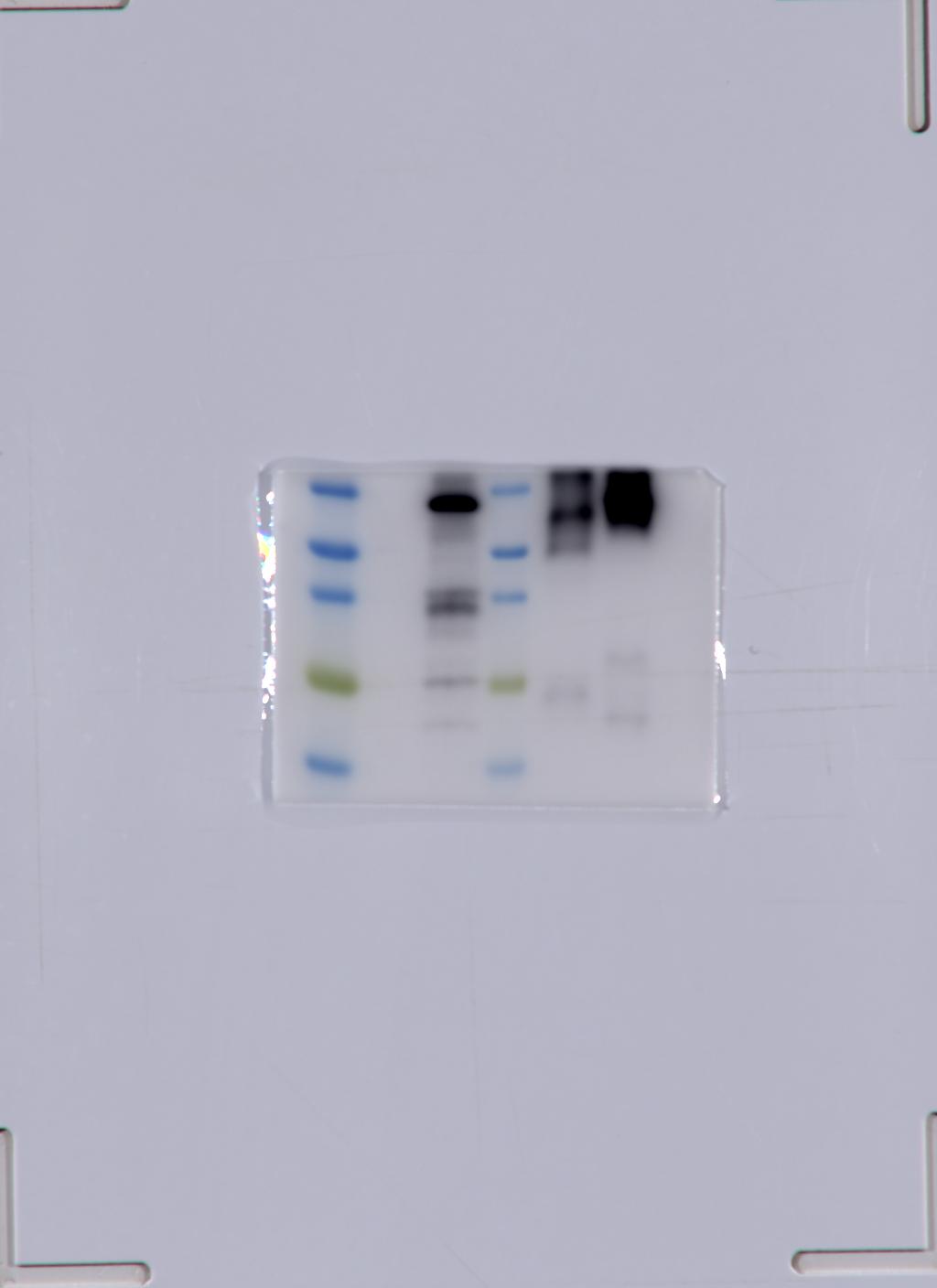

Supplement: Supplementary file 3 — WB Raw data [file 41420_2025_2583_MOESM3_ESM.zip › Figure 6 Panel D/Dcaf13 he 2022.04.03_11.12.49_Ch/Dcaf13 he 2022.04.03_11.12.49_Ch+Marker.jpg]

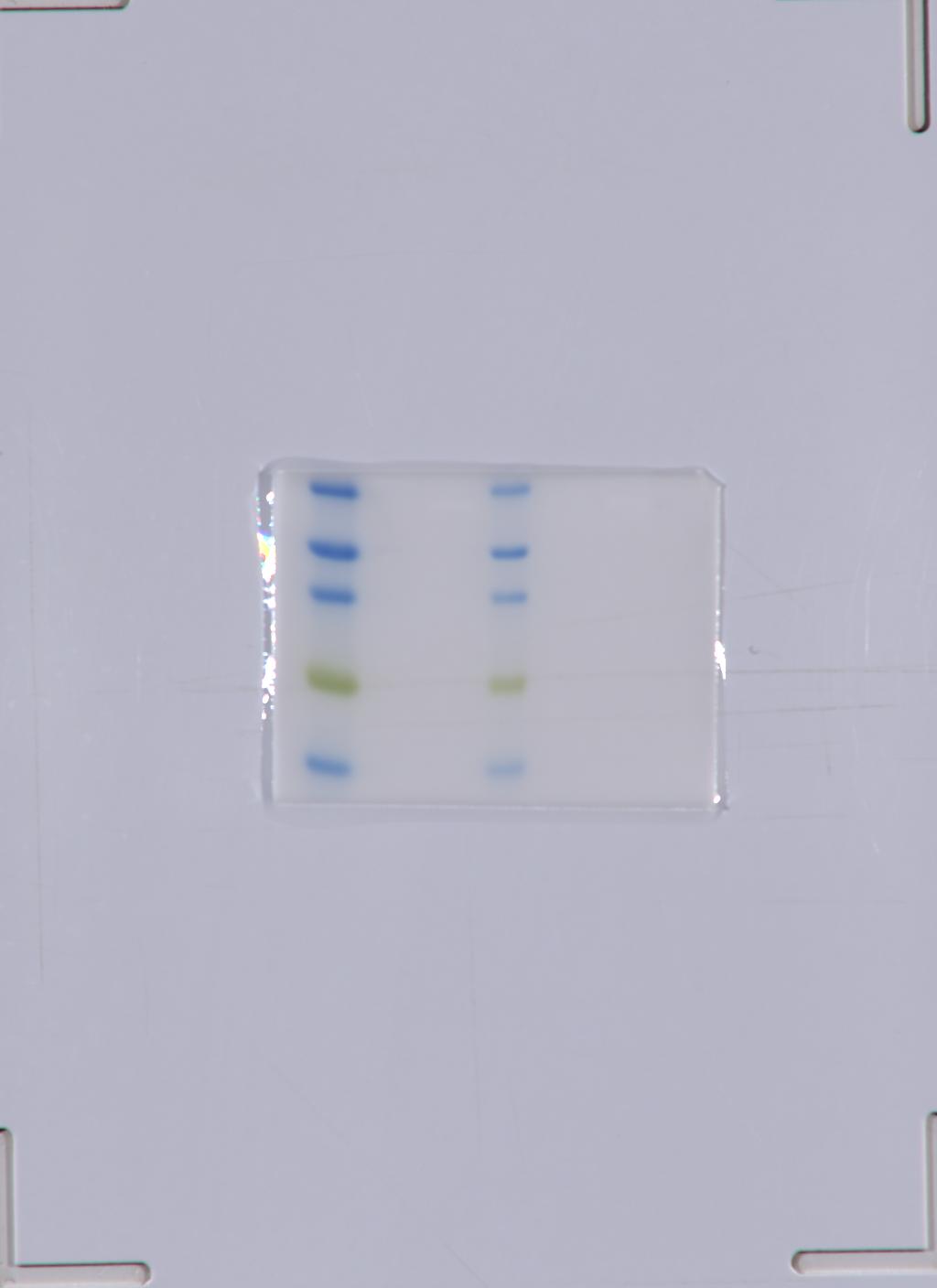

Supplement: Supplementary file 3 — WB Raw data [file 41420_2025_2583_MOESM3_ESM.zip › Figure 6 Panel D/Dcaf13 he 2022.04.03_11.12.49_Ch/Dcaf13 he 2022.04.03_11.12.49_Ch-Marker.jpg]

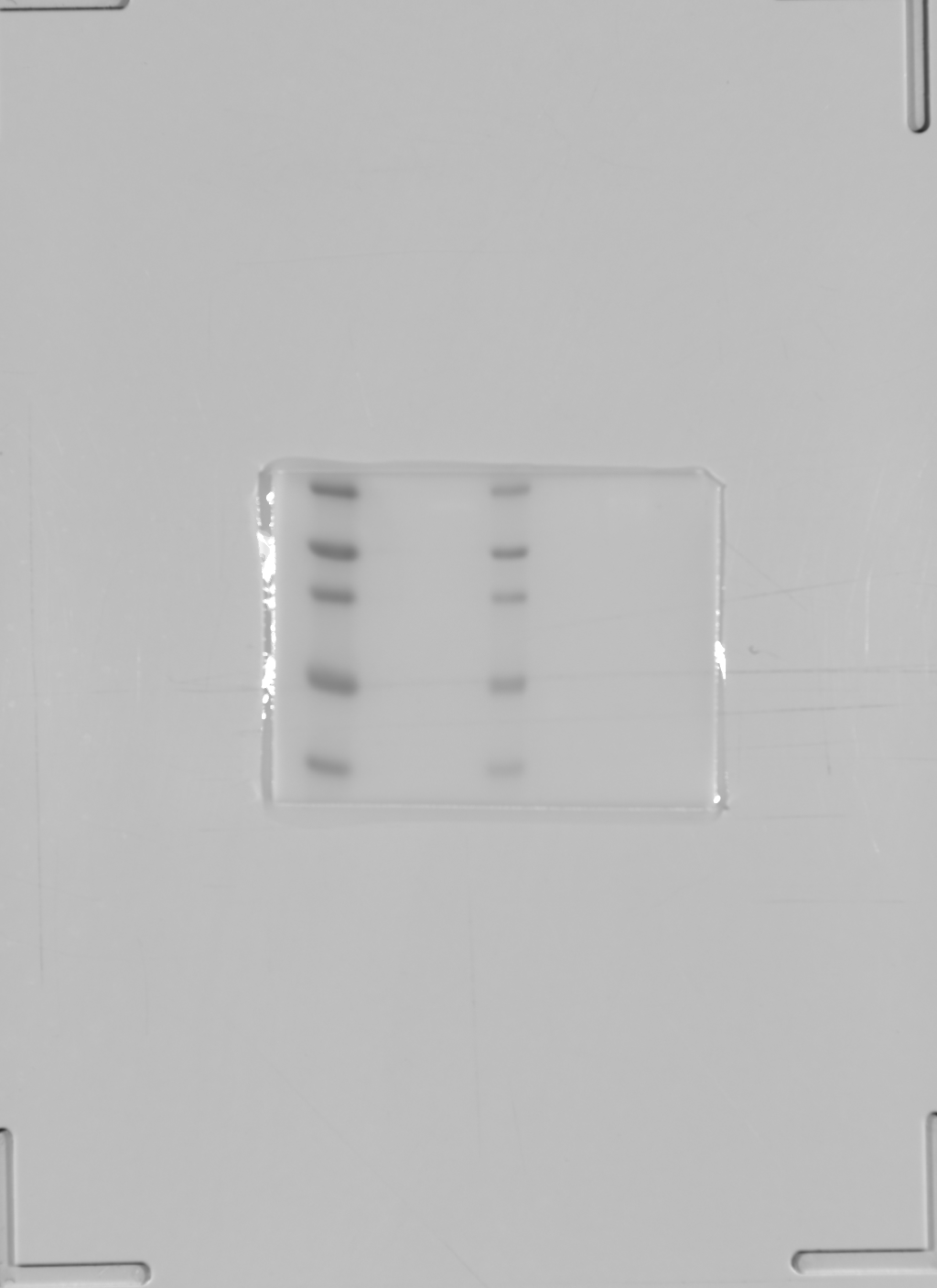

Supplement: Supplementary file 3 — WB Raw data [file 41420_2025_2583_MOESM3_ESM.zip › Figure 6 Panel D/Dcaf13 he 2022.04.03_11.12.49_Ch/Dcaf13 he 2022.04.03_11.12.49_Ch-Marker.tif]

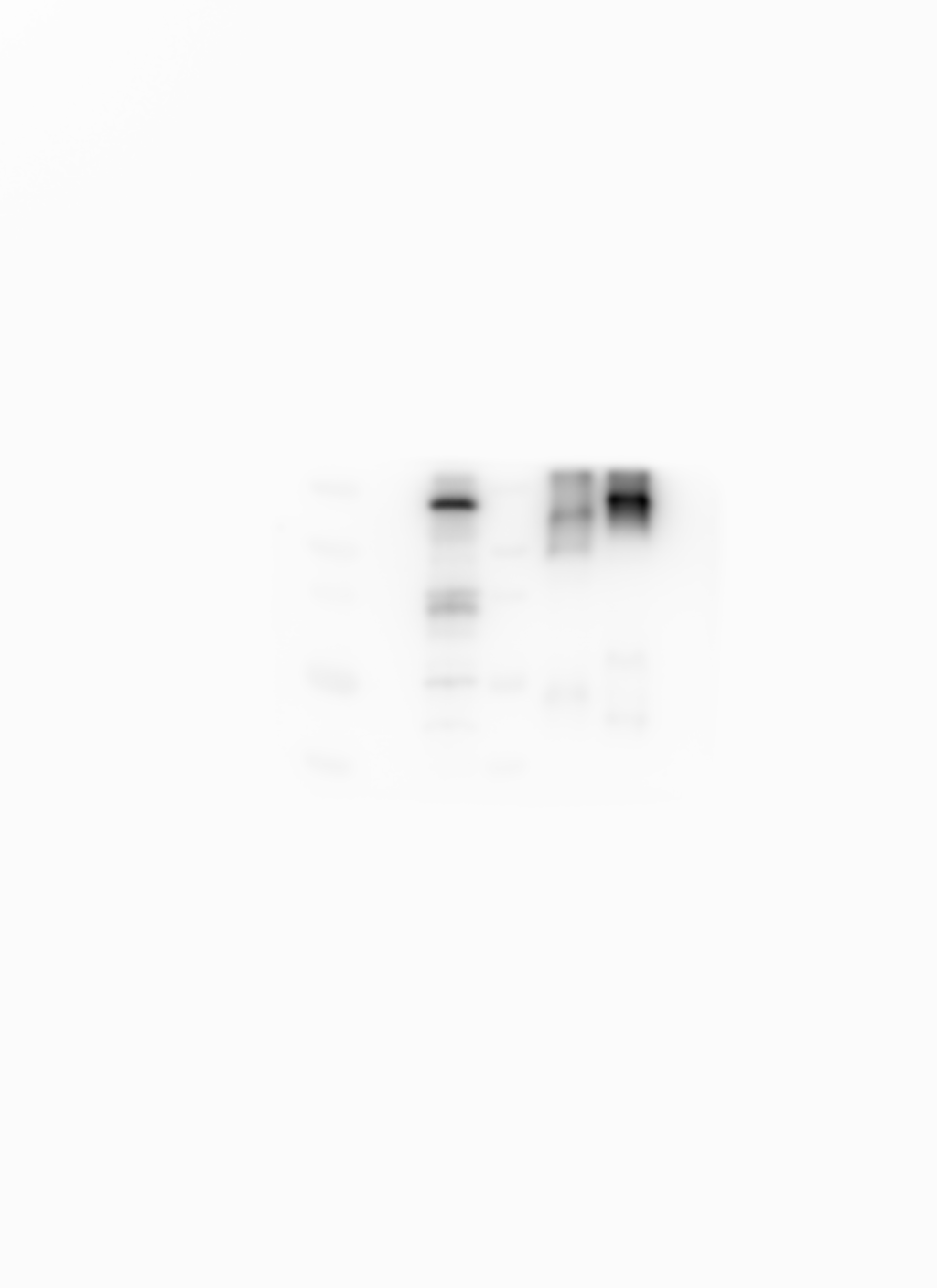

Supplement: Supplementary file 3 — WB Raw data [file 41420_2025_2583_MOESM3_ESM.zip › Figure 6 Panel D/Dcaf13 he 2022.04.03_11.12.49_Ch/Dcaf13 he 2022.04.03_11.12.49_Ch.tif]

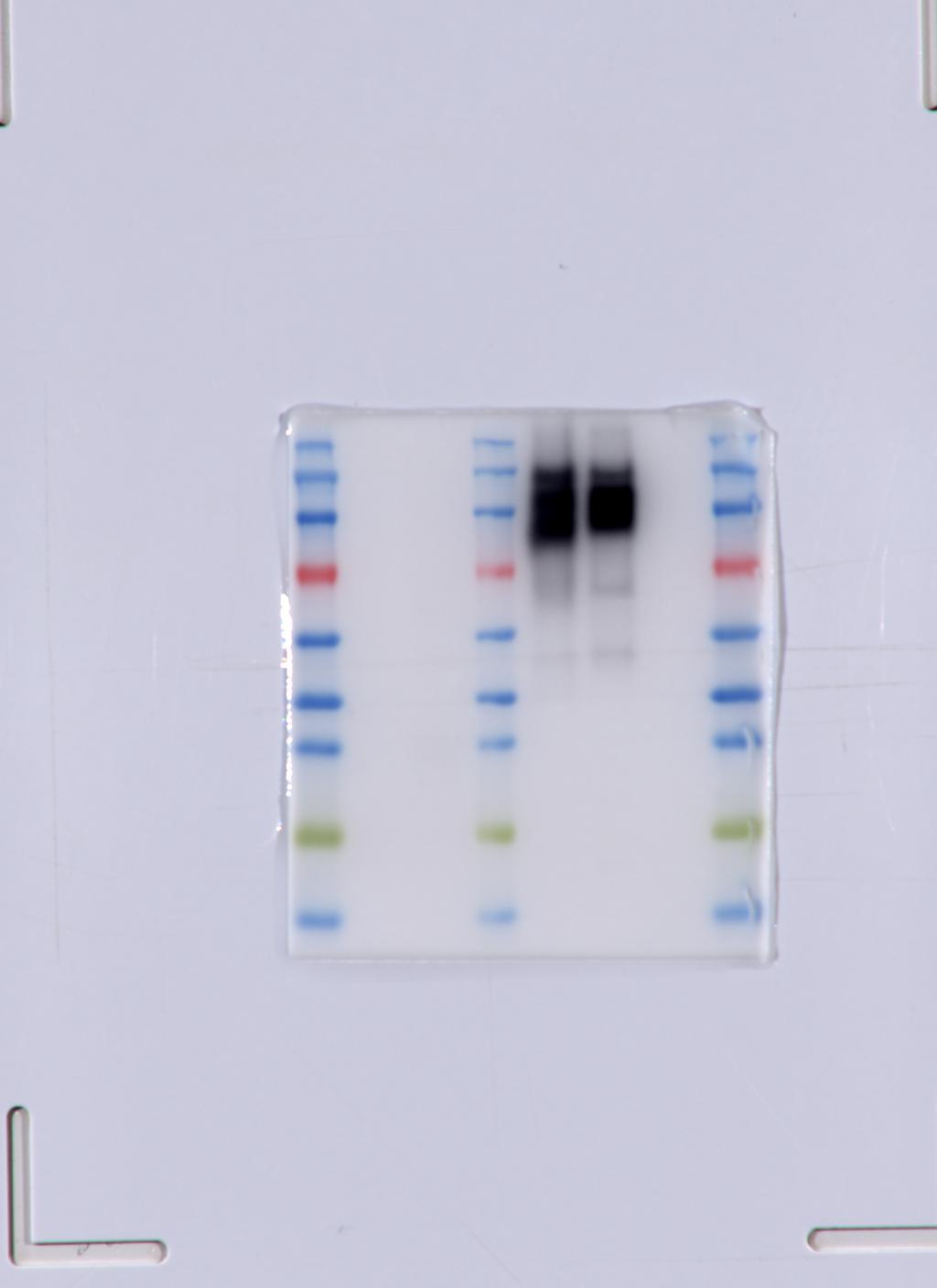

Supplement: Supplementary file 3 — WB Raw data [file 41420_2025_2583_MOESM3_ESM.zip › Figure 6 Panel D/suv39h2 he 2022.04.03_11.14.50_Ch/suv39h2 he 2022.04.03_11.14.50_Ch+Marker.jpg]

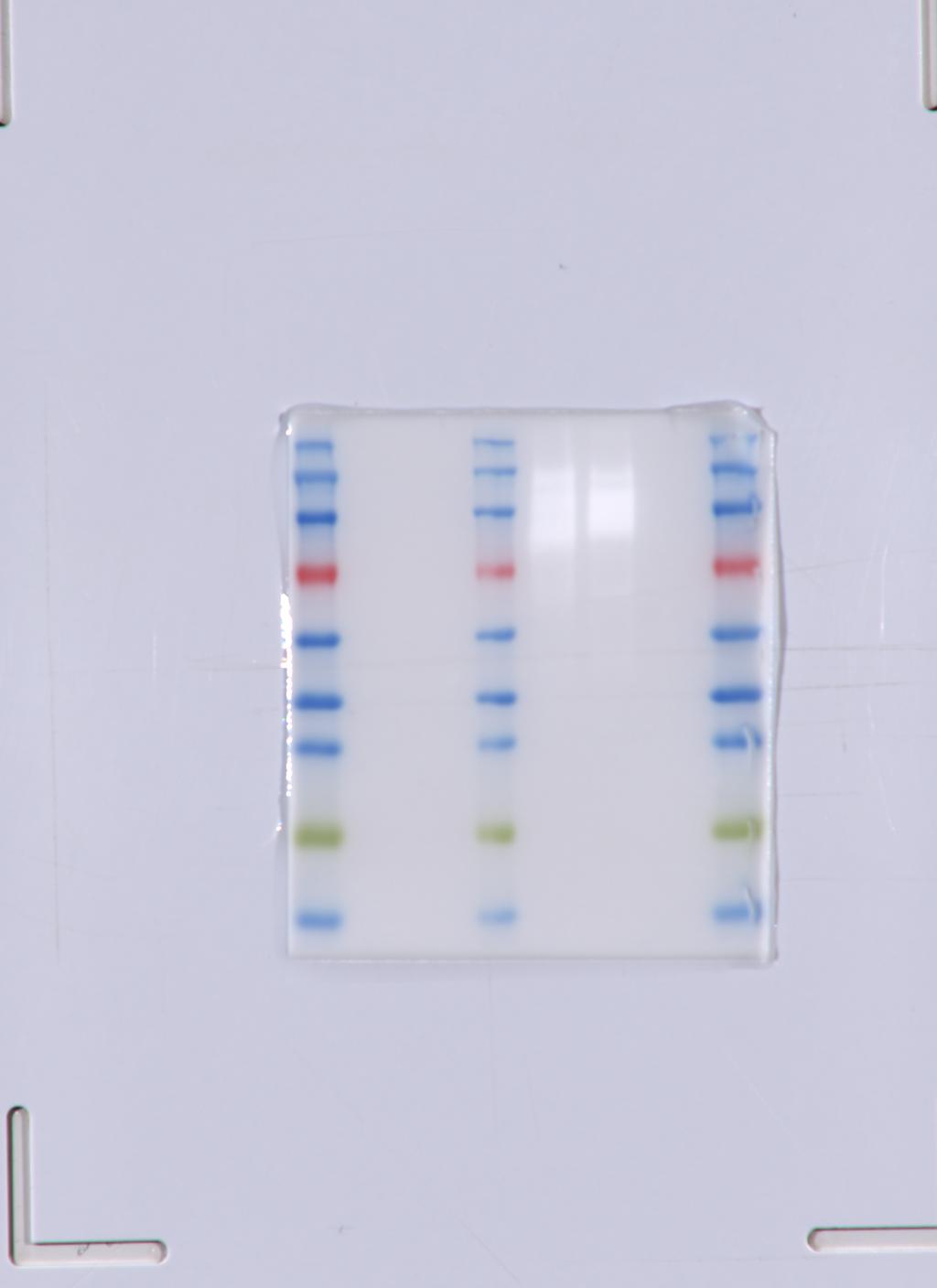

Supplement: Supplementary file 3 — WB Raw data [file 41420_2025_2583_MOESM3_ESM.zip › Figure 6 Panel D/suv39h2 he 2022.04.03_11.14.50_Ch/suv39h2 he 2022.04.03_11.14.50_Ch-Marker.jpg]

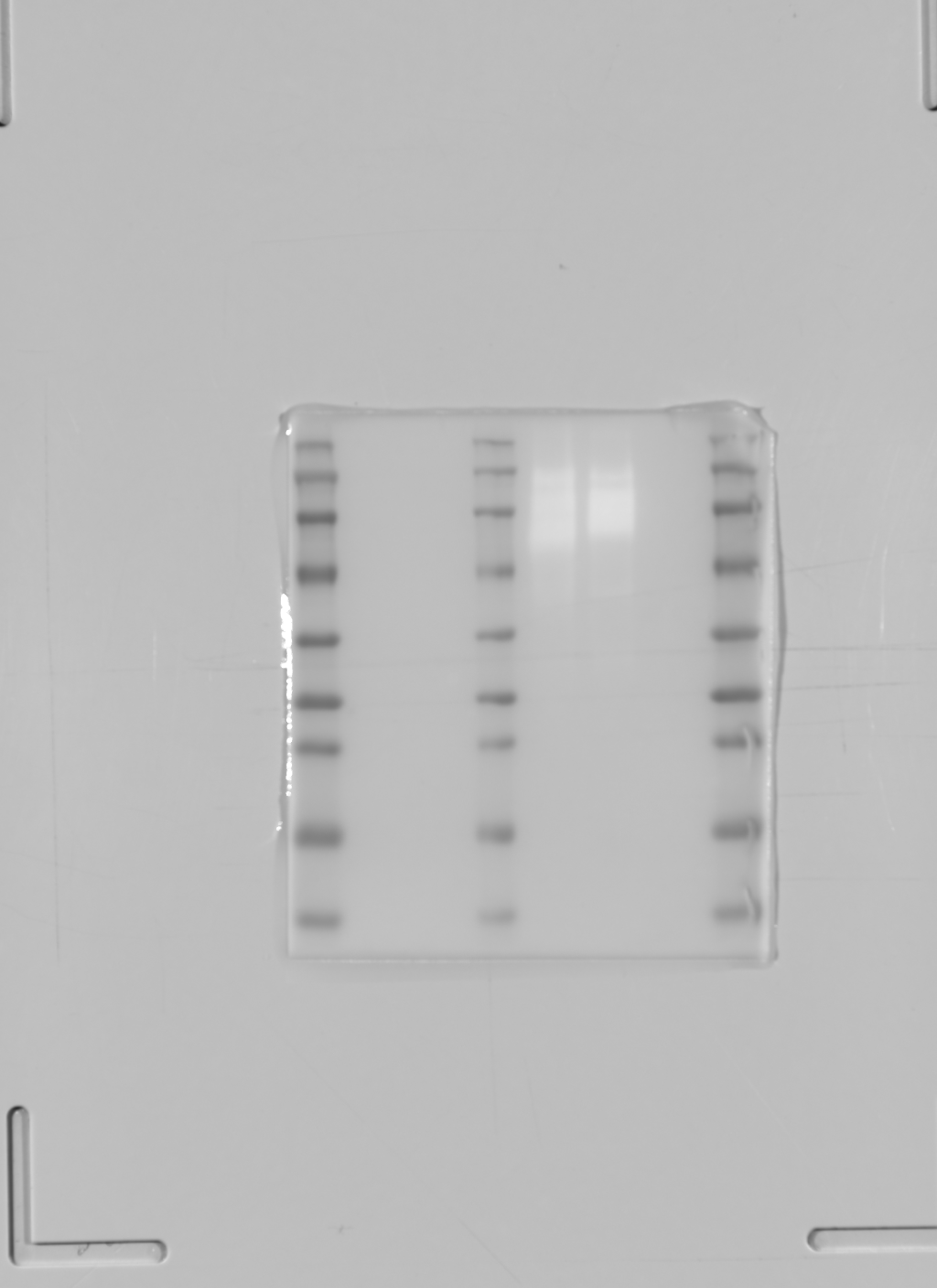

Supplement: Supplementary file 3 — WB Raw data [file 41420_2025_2583_MOESM3_ESM.zip › Figure 6 Panel D/suv39h2 he 2022.04.03_11.14.50_Ch/suv39h2 he 2022.04.03_11.14.50_Ch-Marker.tif]

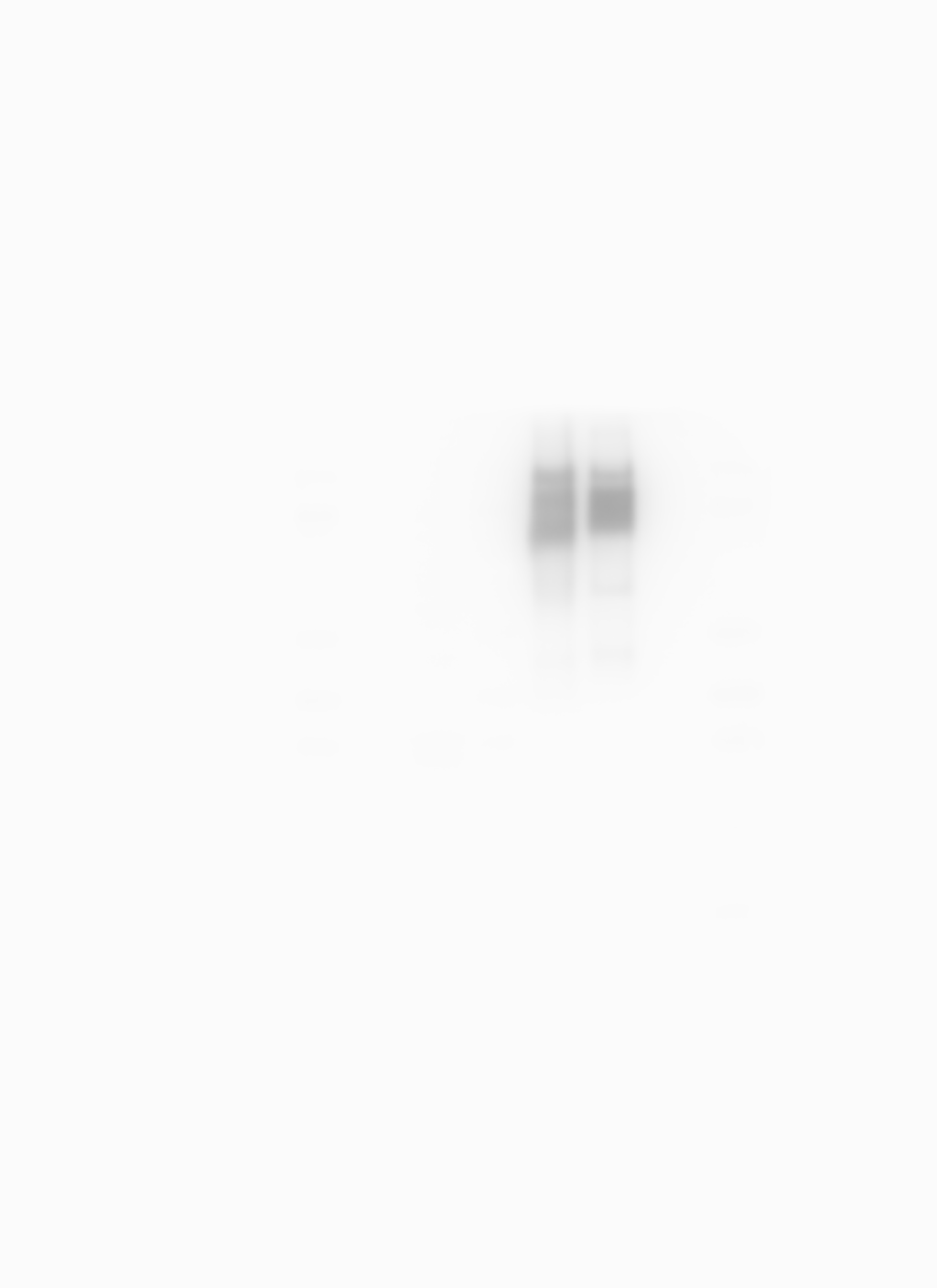

Supplement: Supplementary file 3 — WB Raw data [file 41420_2025_2583_MOESM3_ESM.zip › Figure 6 Panel D/suv39h2 he 2022.04.03_11.14.50_Ch/suv39h2 he 2022.04.03_11.14.50_Ch.tif]

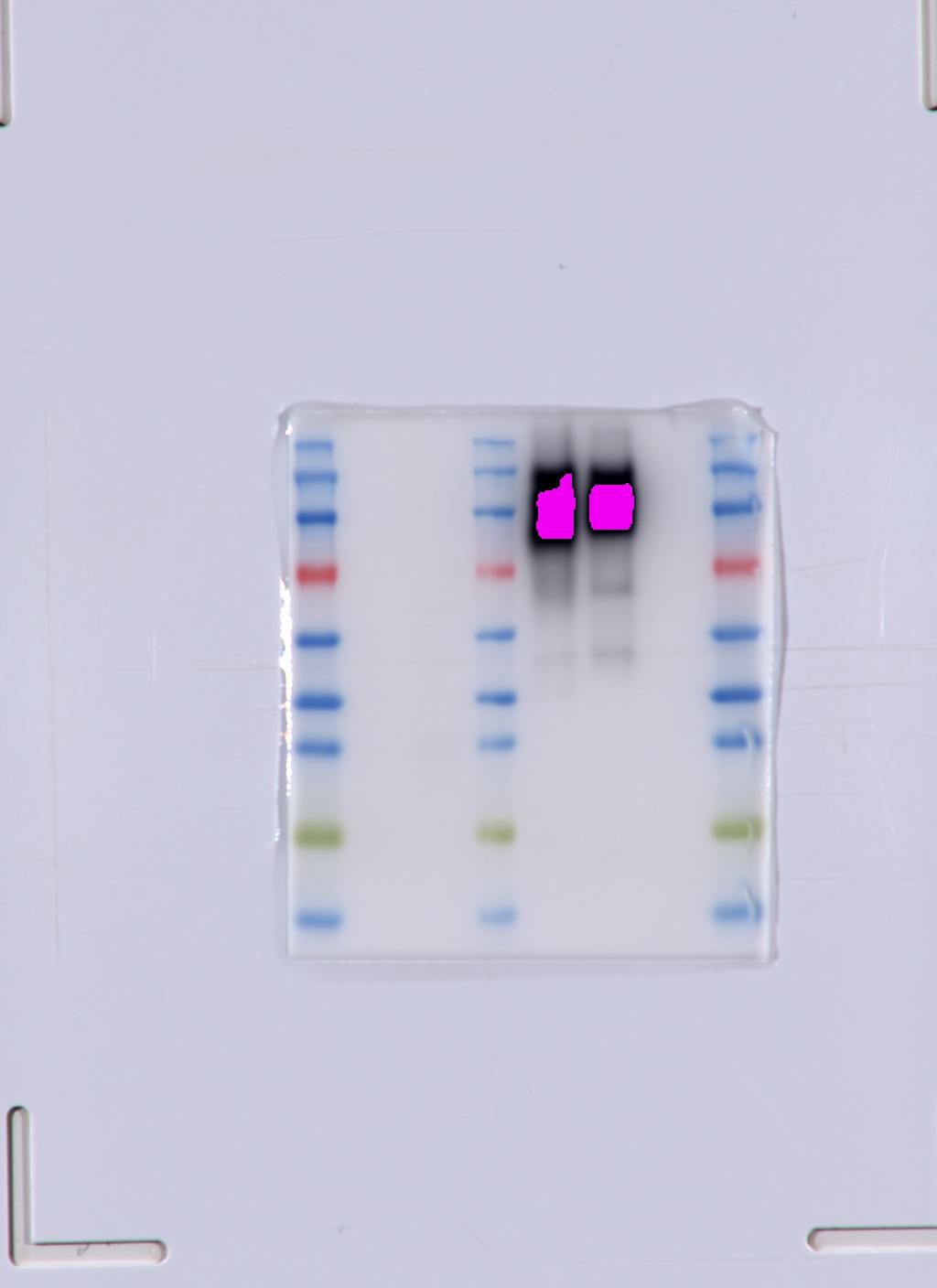

Supplement: Supplementary file 3 — WB Raw data [file 41420_2025_2583_MOESM3_ESM.zip › Figure 6 Panel D/suv39h2 he 2022.04.03_11.16.01_Ch/suv39h2 he 2022.04.03_11.16.01_Ch+Marker.jpg]

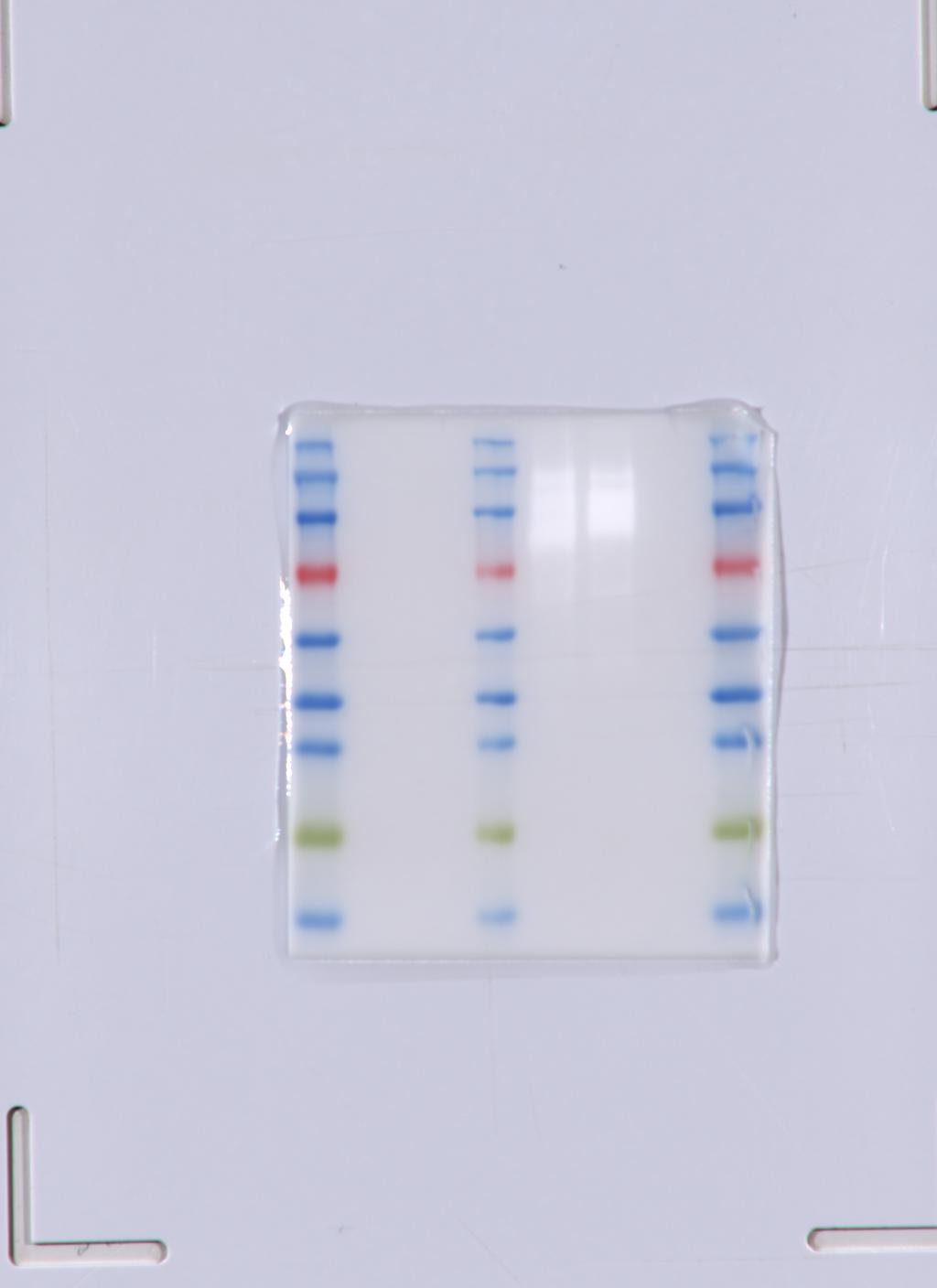

Supplement: Supplementary file 3 — WB Raw data [file 41420_2025_2583_MOESM3_ESM.zip › Figure 6 Panel D/suv39h2 he 2022.04.03_11.16.01_Ch/suv39h2 he 2022.04.03_11.16.01_Ch-Marker.jpg]

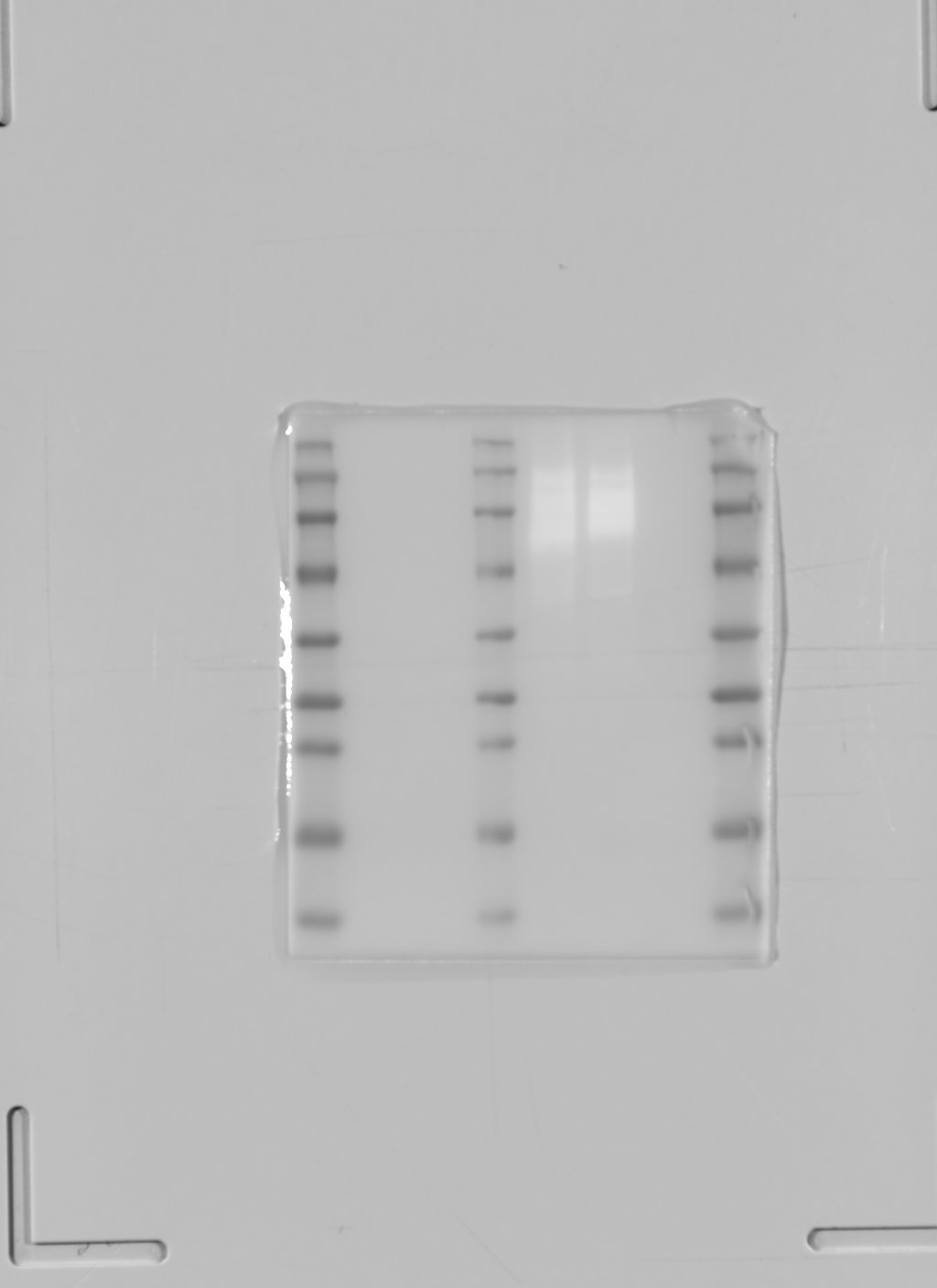

Supplement: Supplementary file 3 — WB Raw data [file 41420_2025_2583_MOESM3_ESM.zip › Figure 6 Panel D/suv39h2 he 2022.04.03_11.16.01_Ch/suv39h2 he 2022.04.03_11.16.01_Ch-Marker.tif]

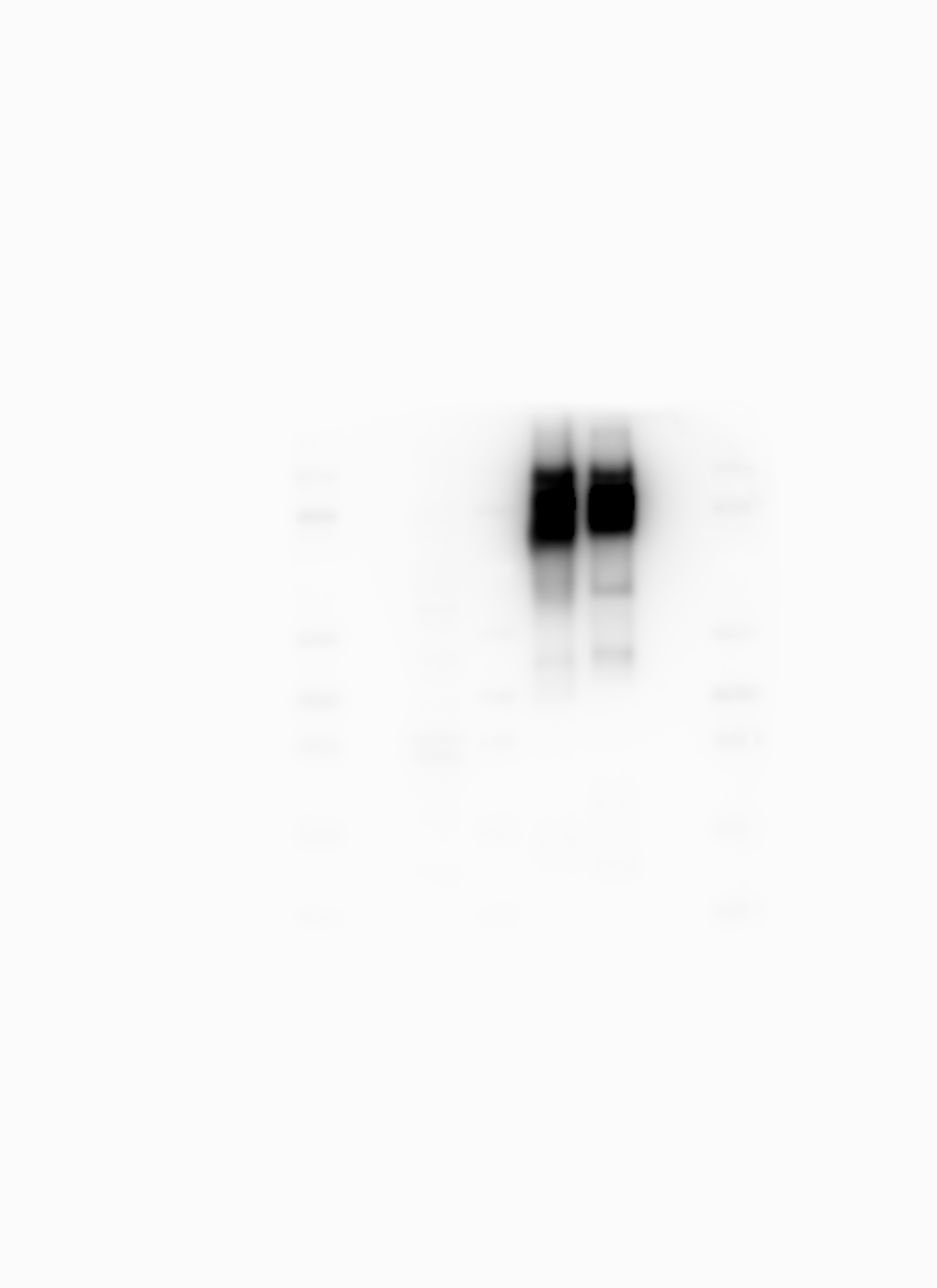

Supplement: Supplementary file 3 — WB Raw data [file 41420_2025_2583_MOESM3_ESM.zip › Figure 6 Panel D/suv39h2 he 2022.04.03_11.16.01_Ch/suv39h2 he 2022.04.03_11.16.01_Ch.tif]

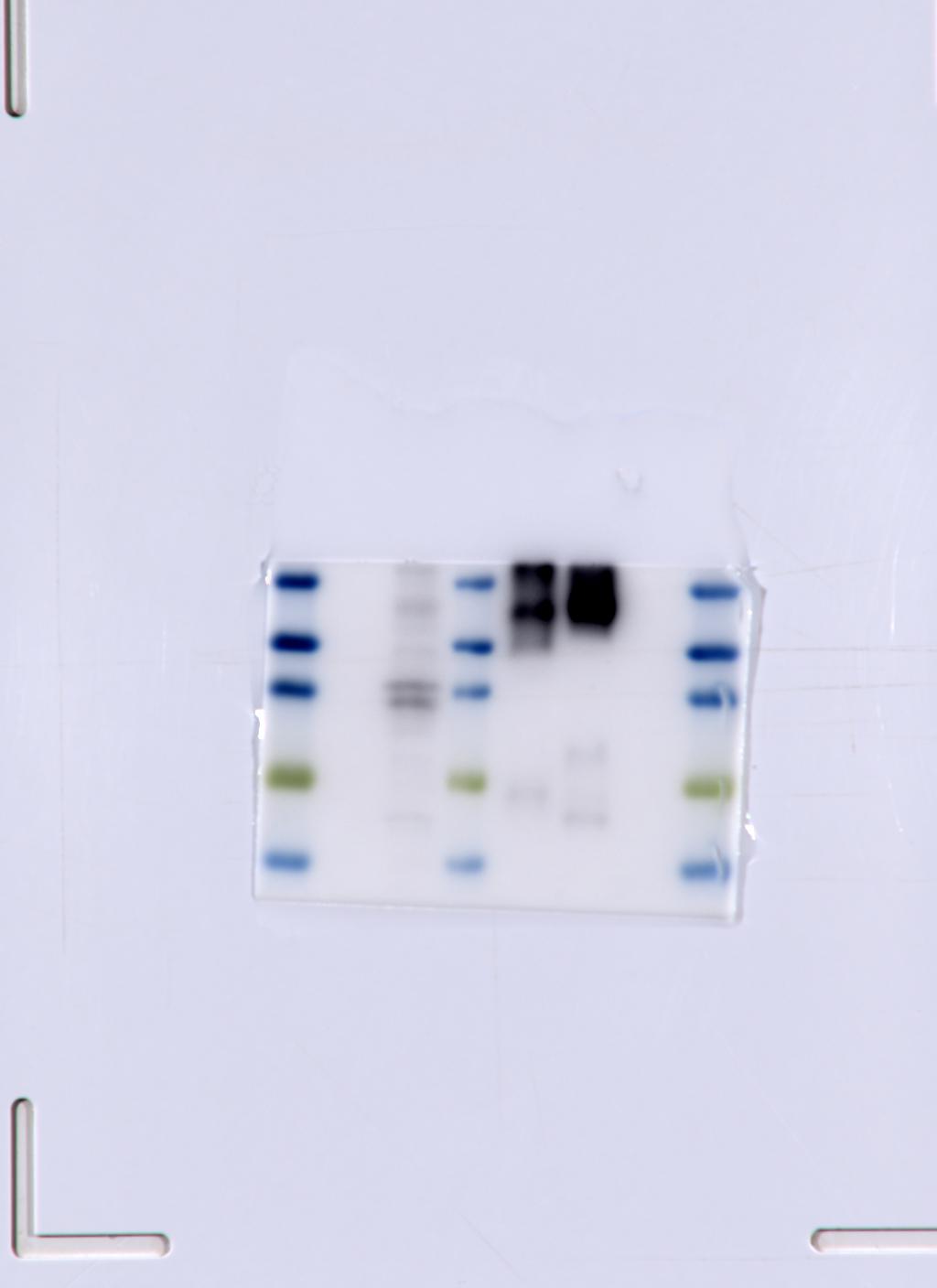

Supplement: Supplementary file 3 — WB Raw data [file 41420_2025_2583_MOESM3_ESM.zip › Figure 6 Panel D/suv39h2 he 2022.04.03_11.19.31_Ch/suv39h2 he 2022.04.03_11.19.31_Ch+Marker.jpg]

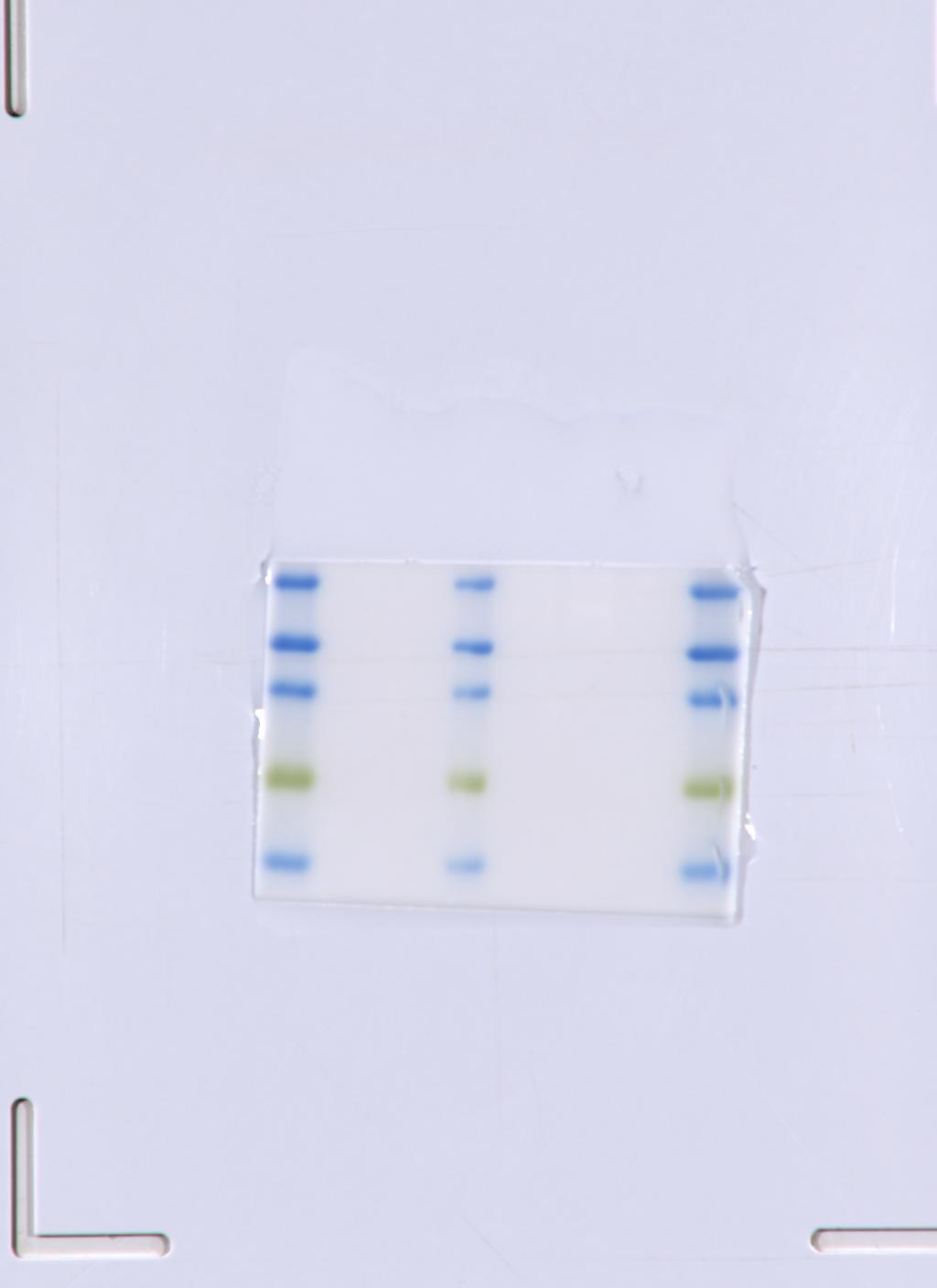

Supplement: Supplementary file 3 — WB Raw data [file 41420_2025_2583_MOESM3_ESM.zip › Figure 6 Panel D/suv39h2 he 2022.04.03_11.19.31_Ch/suv39h2 he 2022.04.03_11.19.31_Ch-Marker.jpg]

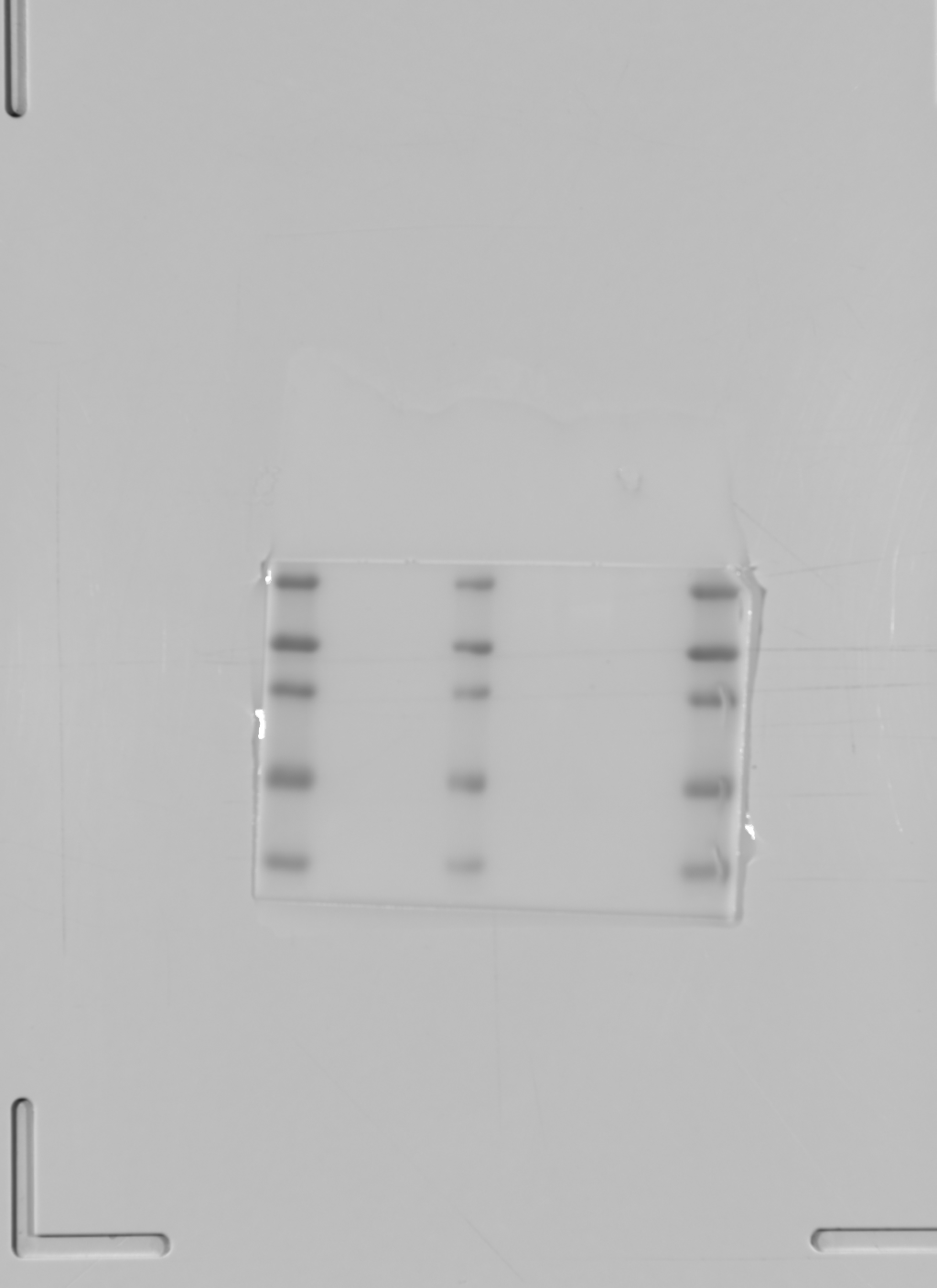

Supplement: Supplementary file 3 — WB Raw data [file 41420_2025_2583_MOESM3_ESM.zip › Figure 6 Panel D/suv39h2 he 2022.04.03_11.19.31_Ch/suv39h2 he 2022.04.03_11.19.31_Ch-Marker.tif]

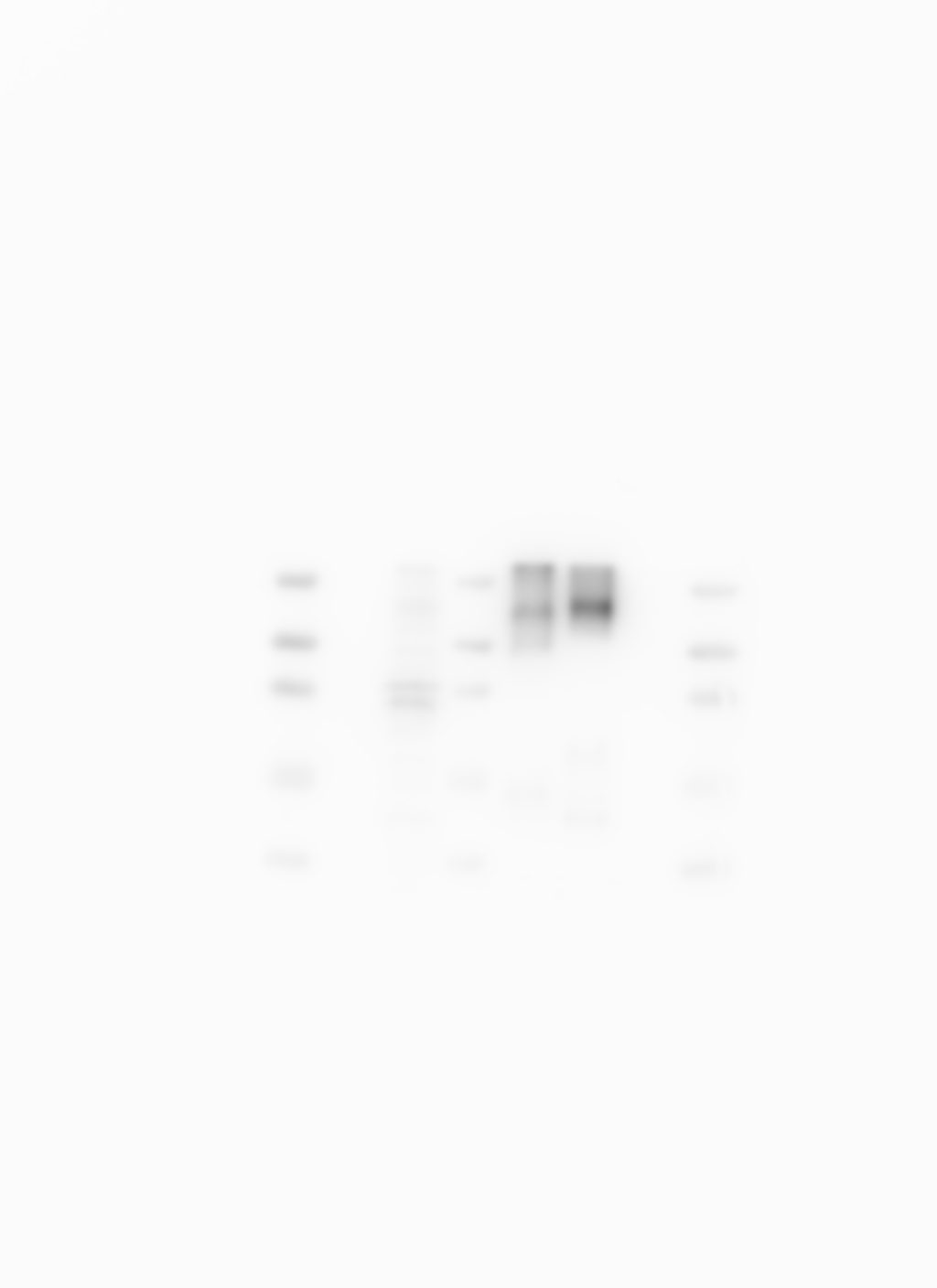

Supplement: Supplementary file 3 — WB Raw data [file 41420_2025_2583_MOESM3_ESM.zip › Figure 6 Panel D/suv39h2 he 2022.04.03_11.19.31_Ch/suv39h2 he 2022.04.03_11.19.31_Ch.tif]

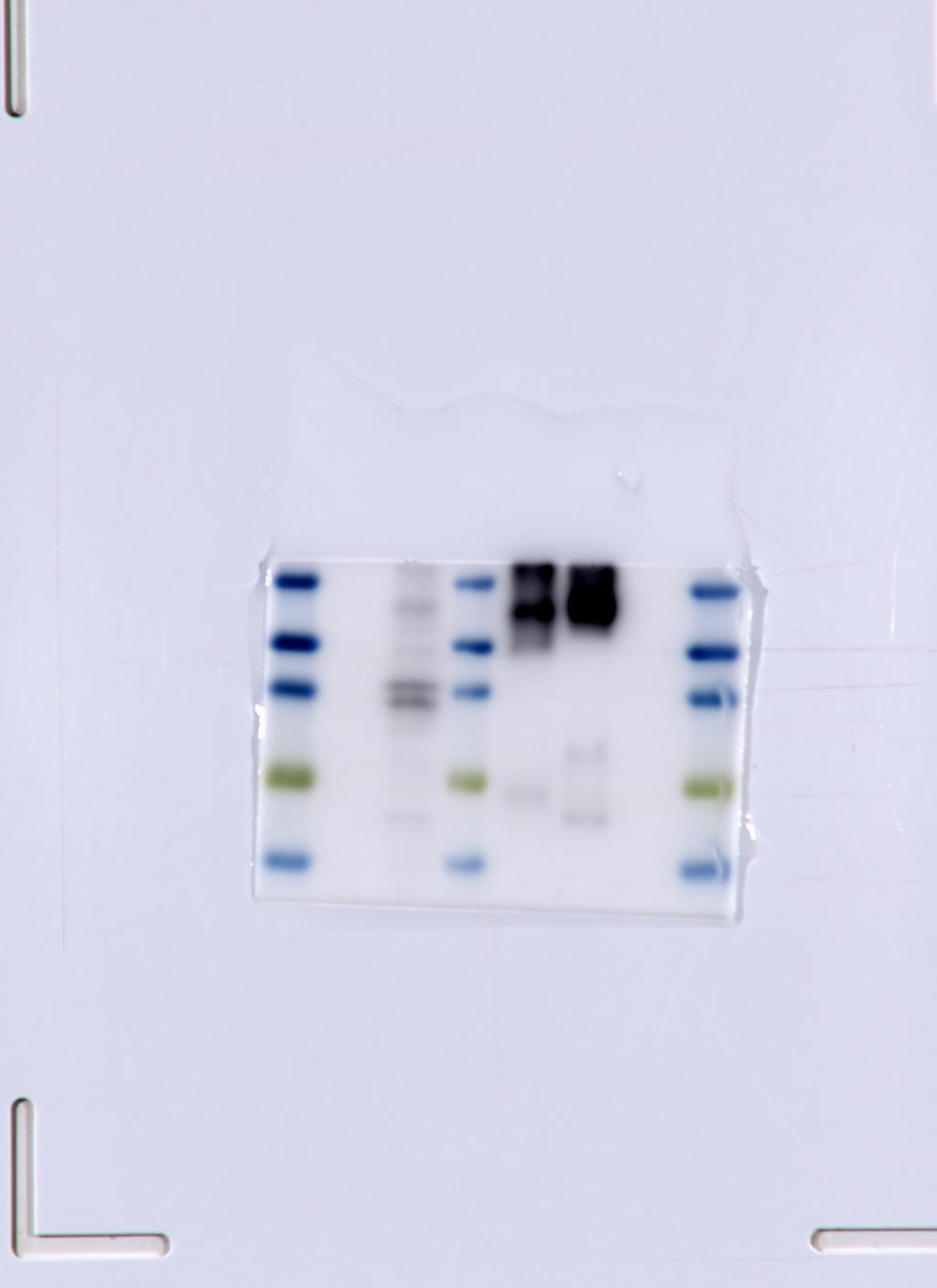

Supplement: Supplementary file 3 — WB Raw data [file 41420_2025_2583_MOESM3_ESM.zip › Figure 6 Panel D/suv39h2 he 2022.04.03_11.20.52_Ch/suv39h2 he 2022.04.03_11.20.52_Ch+Marker.jpg]

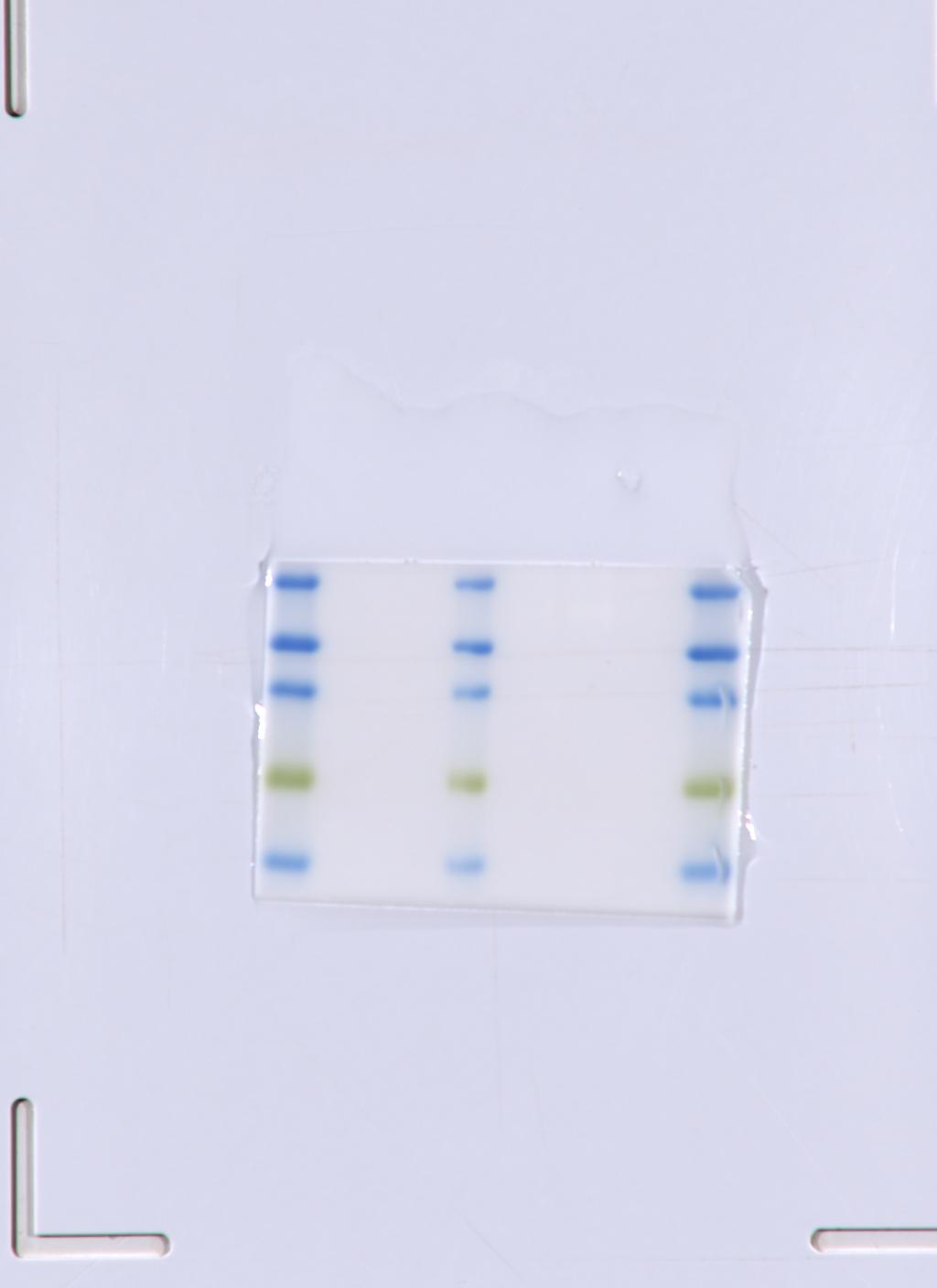

Supplement: Supplementary file 3 — WB Raw data [file 41420_2025_2583_MOESM3_ESM.zip › Figure 6 Panel D/suv39h2 he 2022.04.03_11.20.52_Ch/suv39h2 he 2022.04.03_11.20.52_Ch-Marker.jpg]

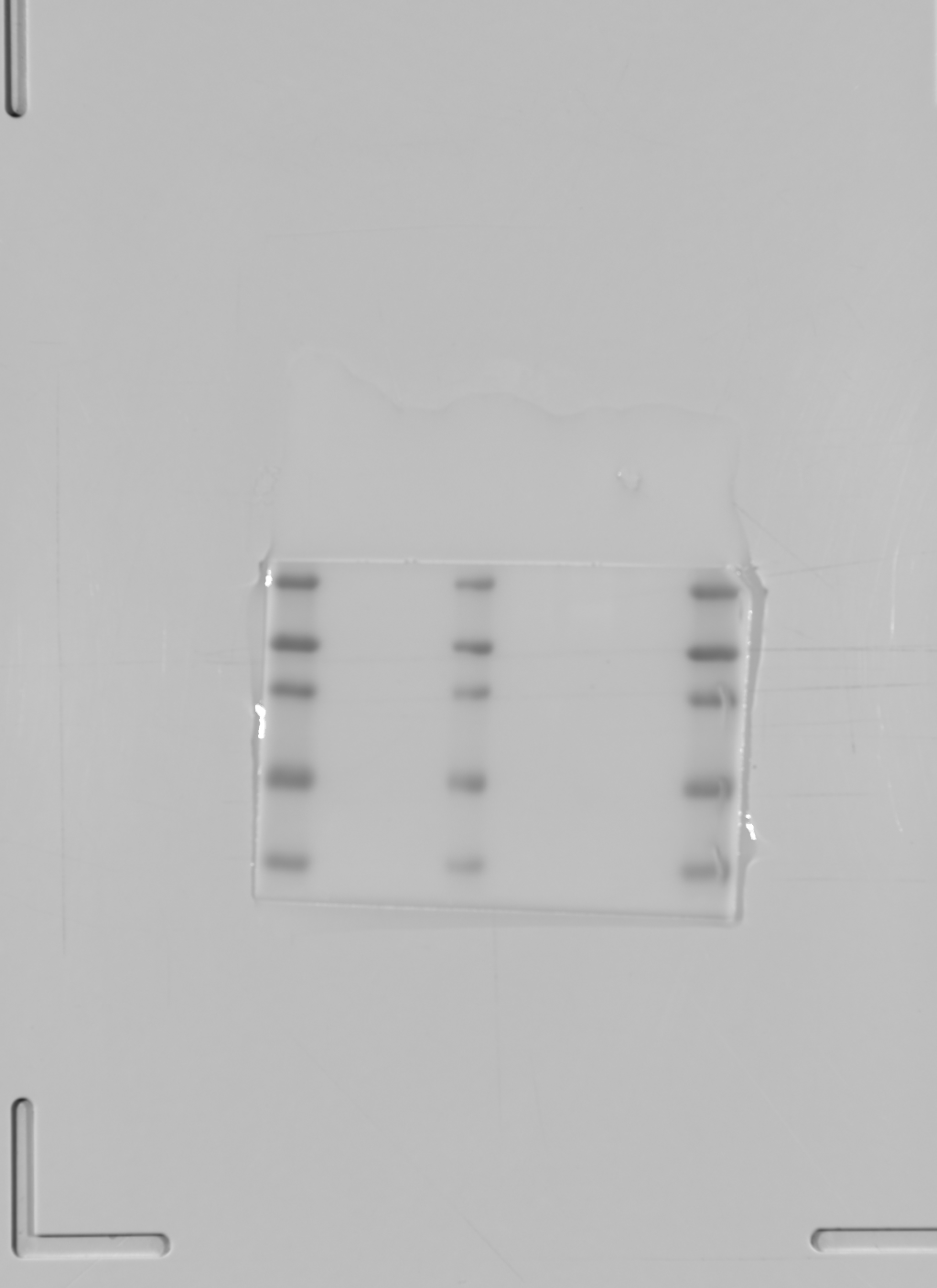

Supplement: Supplementary file 3 — WB Raw data [file 41420_2025_2583_MOESM3_ESM.zip › Figure 6 Panel D/suv39h2 he 2022.04.03_11.20.52_Ch/suv39h2 he 2022.04.03_11.20.52_Ch-Marker.tif]

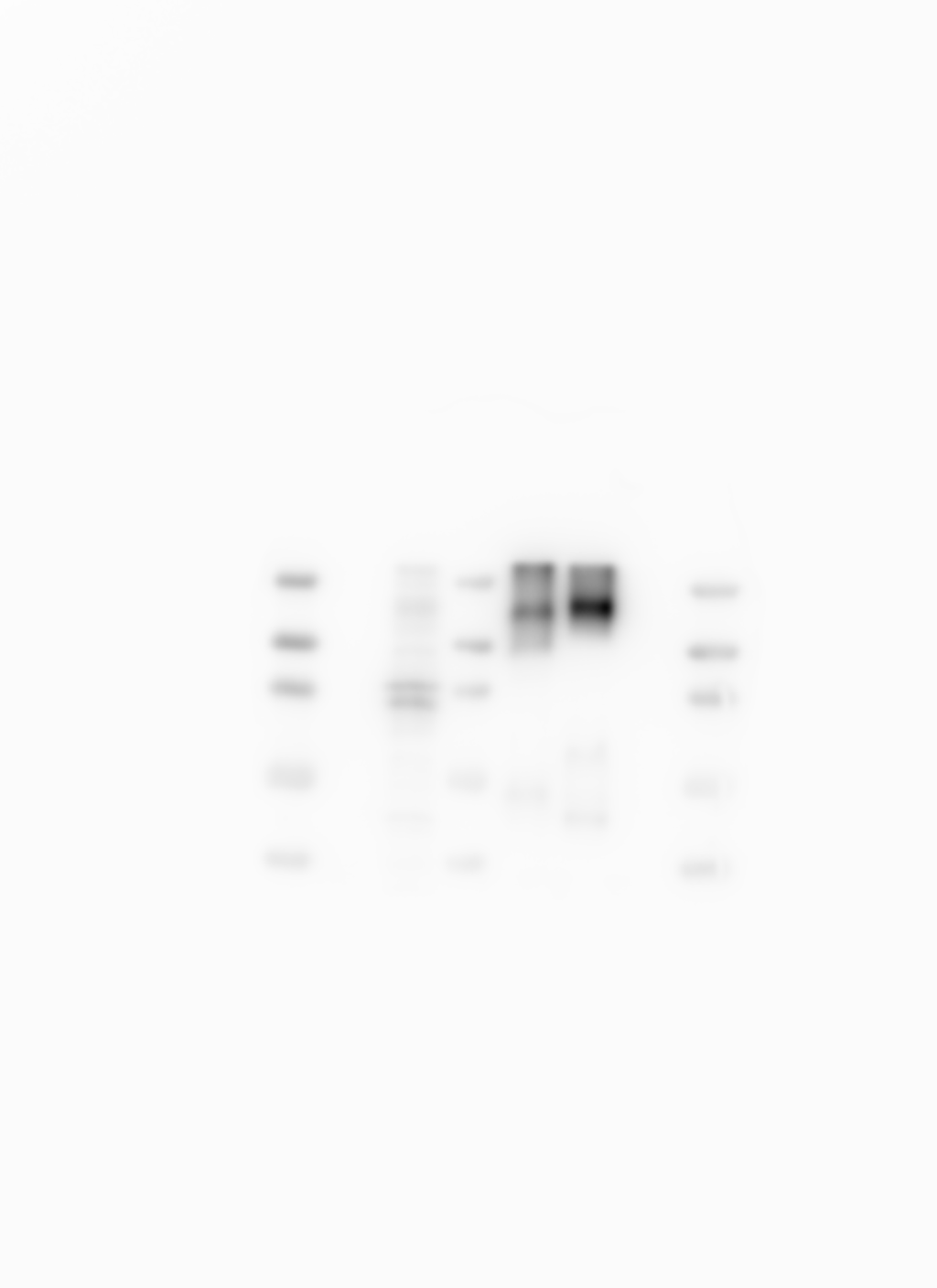

Supplement: Supplementary file 3 — WB Raw data [file 41420_2025_2583_MOESM3_ESM.zip › Figure 6 Panel D/suv39h2 he 2022.04.03_11.20.52_Ch/suv39h2 he 2022.04.03_11.20.52_Ch.tif]

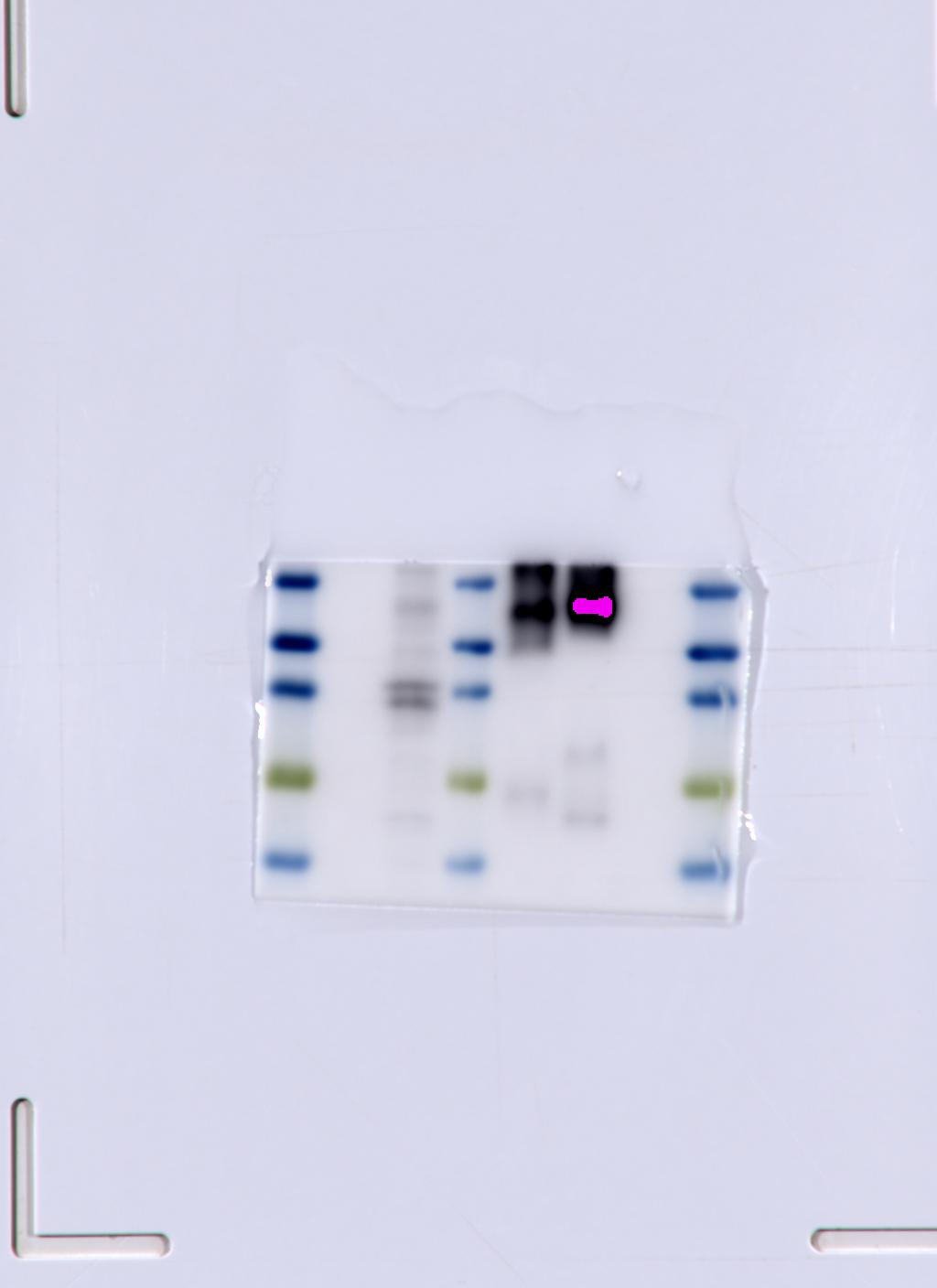

Supplement: Supplementary file 3 — WB Raw data [file 41420_2025_2583_MOESM3_ESM.zip › Figure 6 Panel D/suv39h2 he 2022.04.03_11.21.55_Ch/suv39h2 he 2022.04.03_11.21.55_Ch+Marker.jpg]

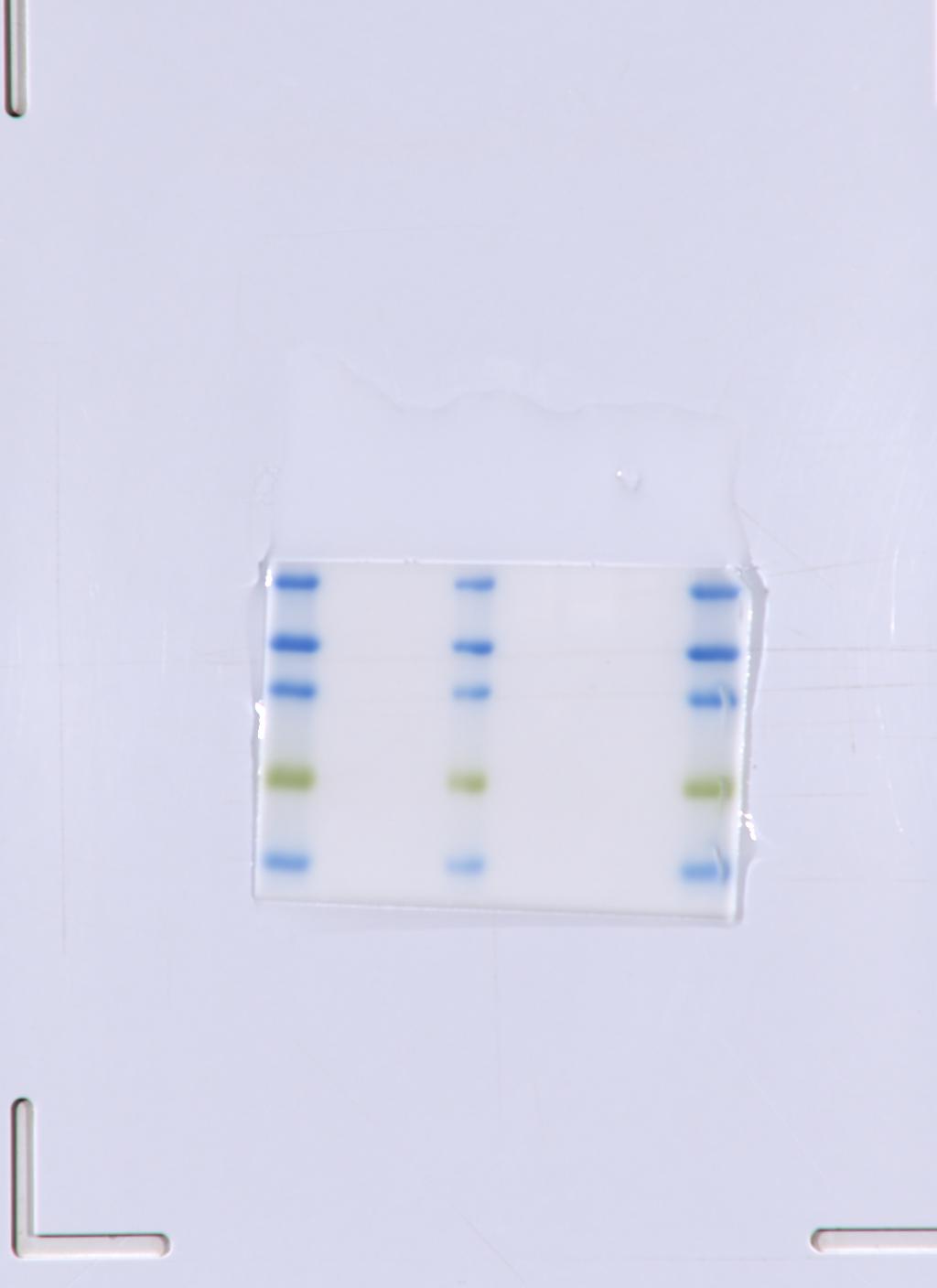

Supplement: Supplementary file 3 — WB Raw data [file 41420_2025_2583_MOESM3_ESM.zip › Figure 6 Panel D/suv39h2 he 2022.04.03_11.21.55_Ch/suv39h2 he 2022.04.03_11.21.55_Ch-Marker.jpg]

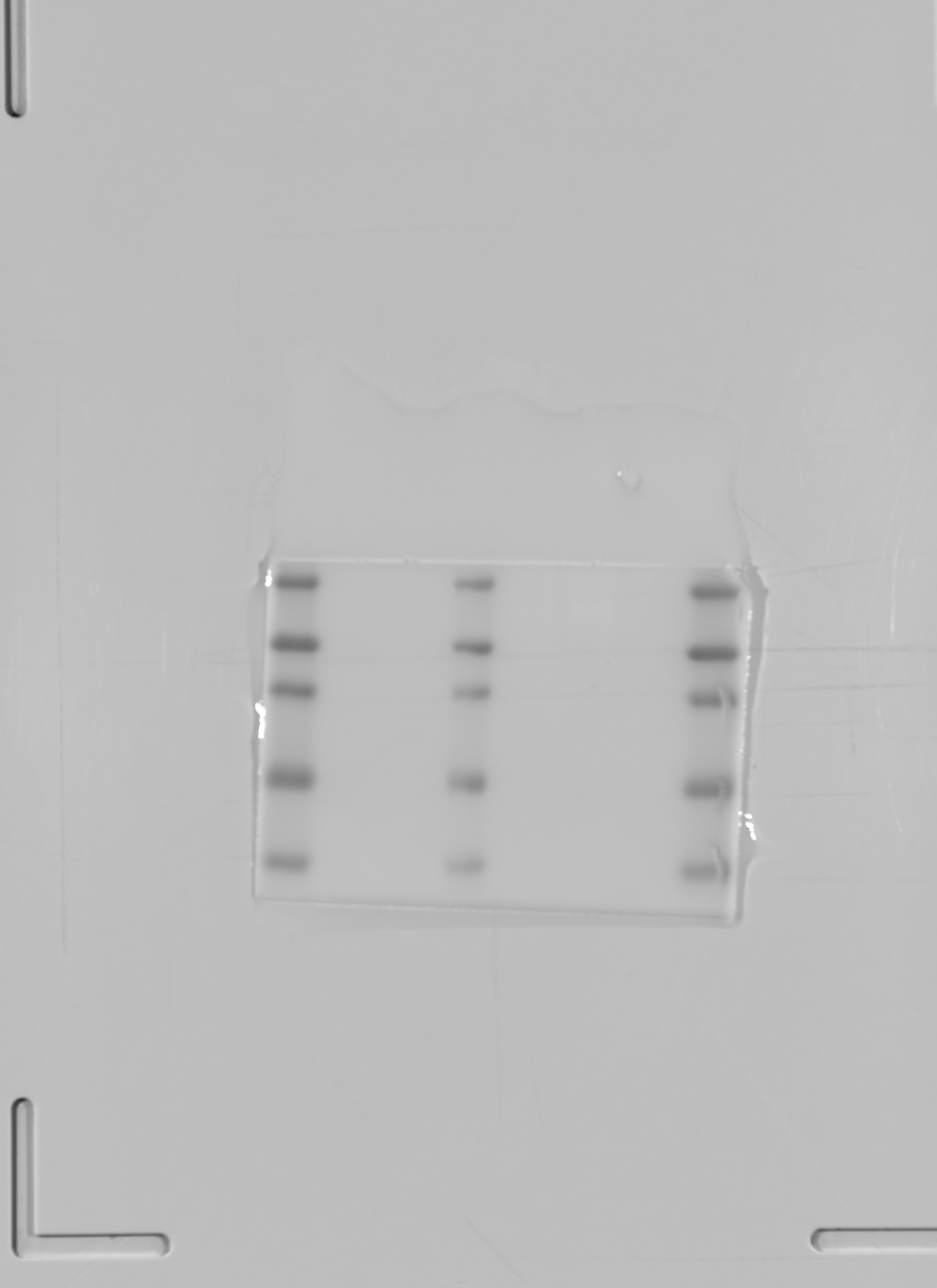

Supplement: Supplementary file 3 — WB Raw data [file 41420_2025_2583_MOESM3_ESM.zip › Figure 6 Panel D/suv39h2 he 2022.04.03_11.21.55_Ch/suv39h2 he 2022.04.03_11.21.55_Ch-Marker.tif]

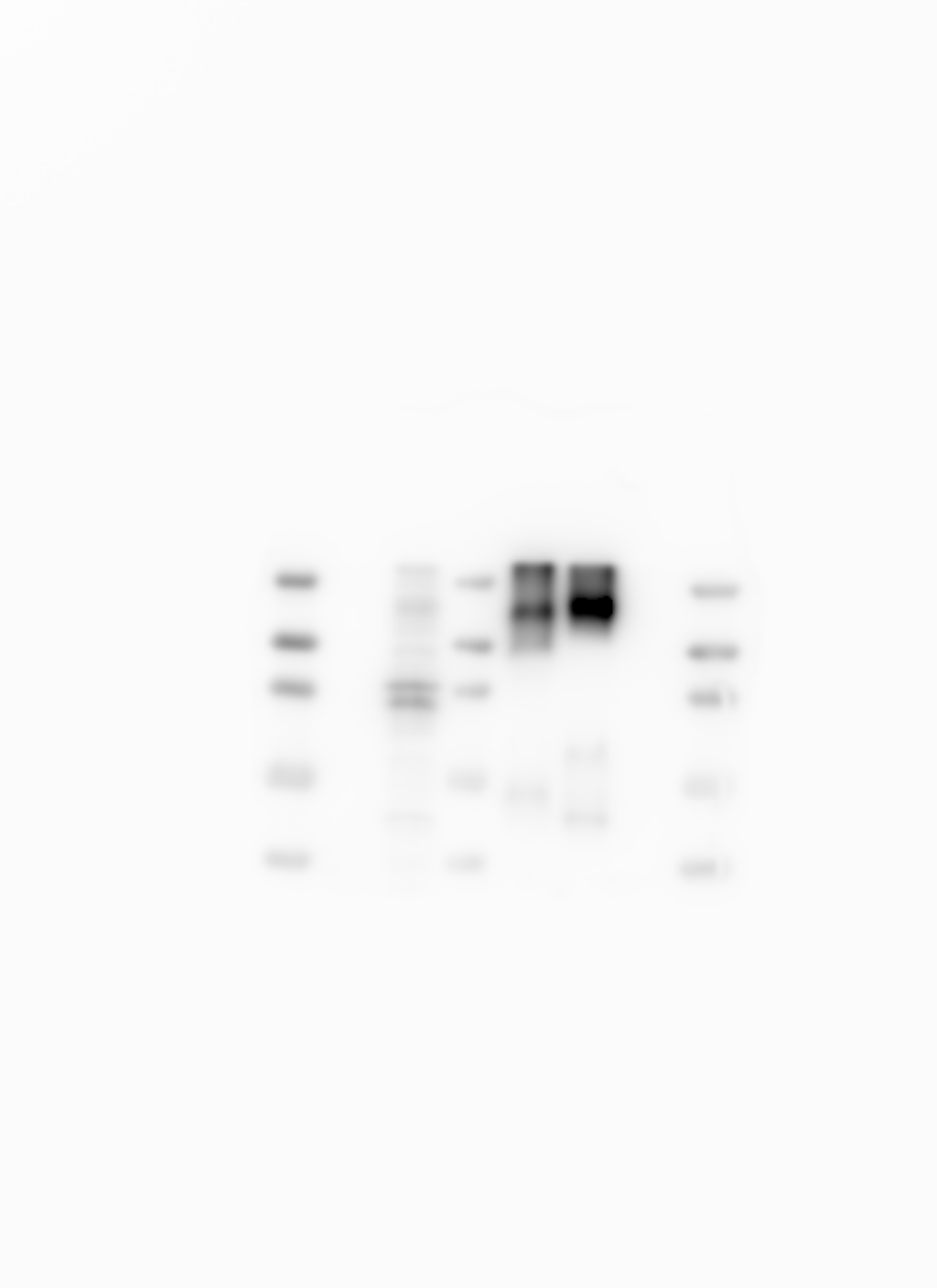

Supplement: Supplementary file 3 — WB Raw data [file 41420_2025_2583_MOESM3_ESM.zip › Figure 6 Panel D/suv39h2 he 2022.04.03_11.21.55_Ch/suv39h2 he 2022.04.03_11.21.55_Ch.tif]

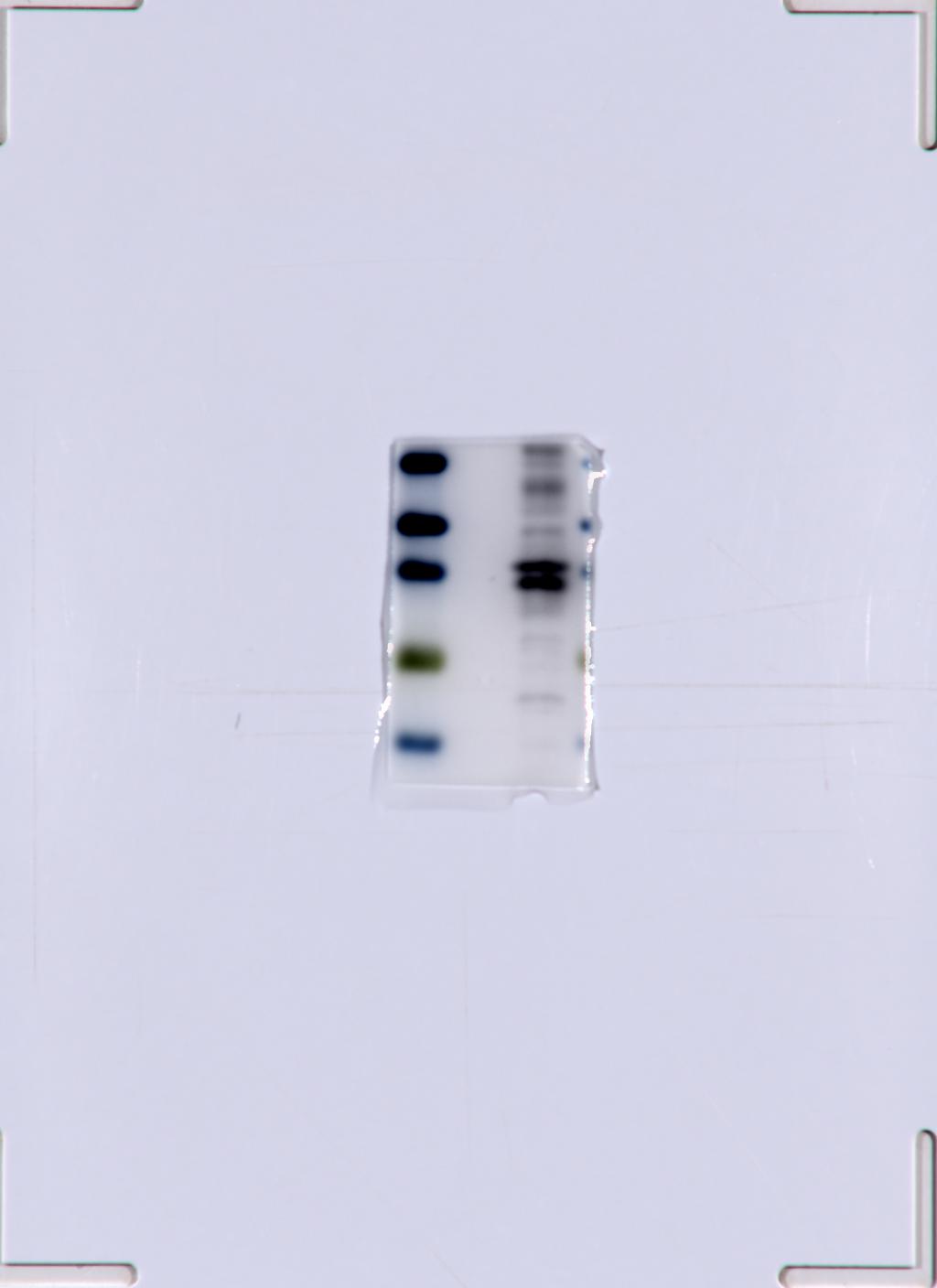

Supplement: Supplementary file 3 — WB Raw data [file 41420_2025_2583_MOESM3_ESM.zip › Figure 6 Panel D/suv39h2 ip 2022.04.03_11.24.01_Ch/suv39h2 ip 2022.04.03_11.24.01_Ch+Marker.jpg]

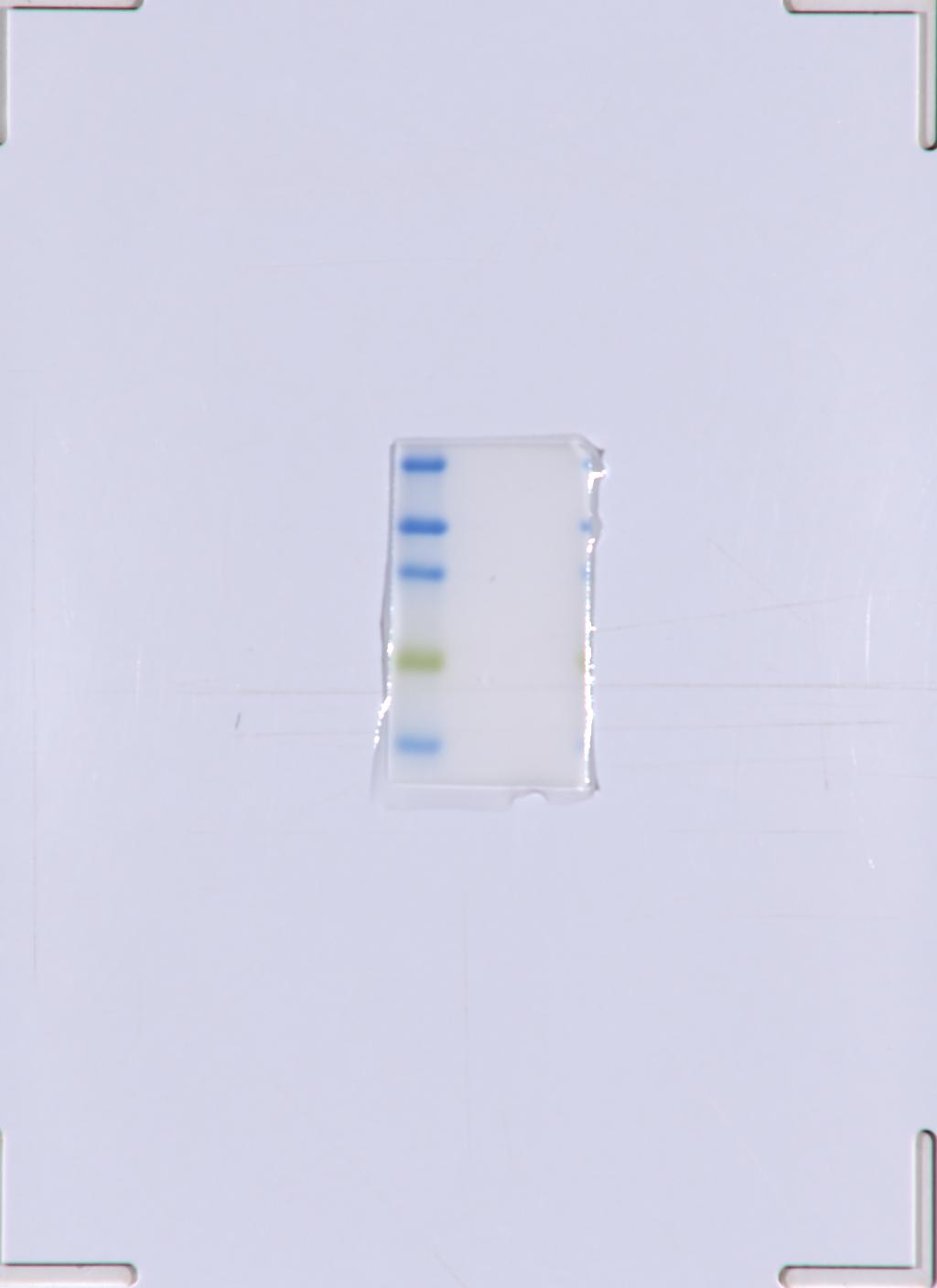

Supplement: Supplementary file 3 — WB Raw data [file 41420_2025_2583_MOESM3_ESM.zip › Figure 6 Panel D/suv39h2 ip 2022.04.03_11.24.01_Ch/suv39h2 ip 2022.04.03_11.24.01_Ch-Marker.jpg]

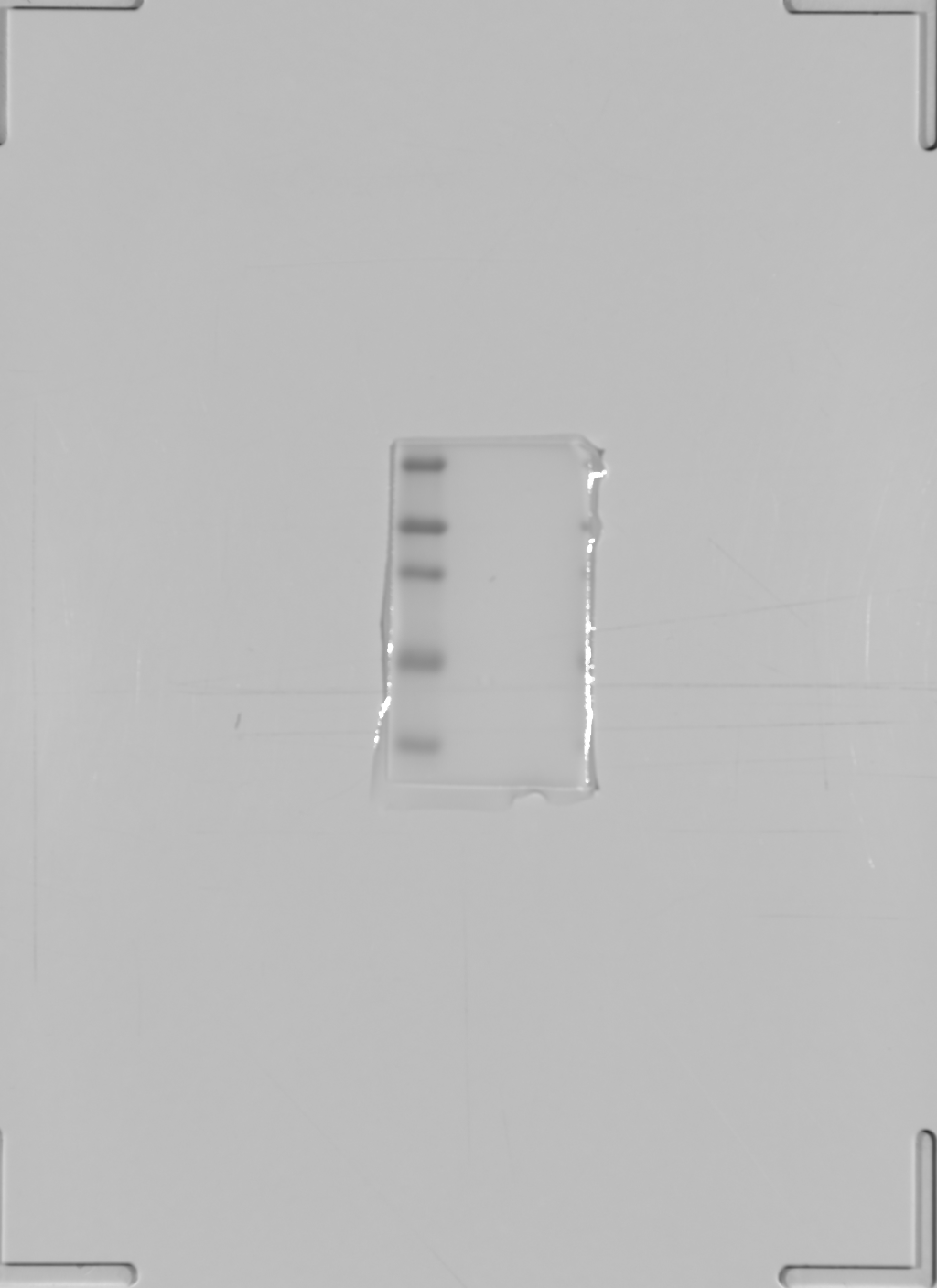

Supplement: Supplementary file 3 — WB Raw data [file 41420_2025_2583_MOESM3_ESM.zip › Figure 6 Panel D/suv39h2 ip 2022.04.03_11.24.01_Ch/suv39h2 ip 2022.04.03_11.24.01_Ch-Marker.tif]

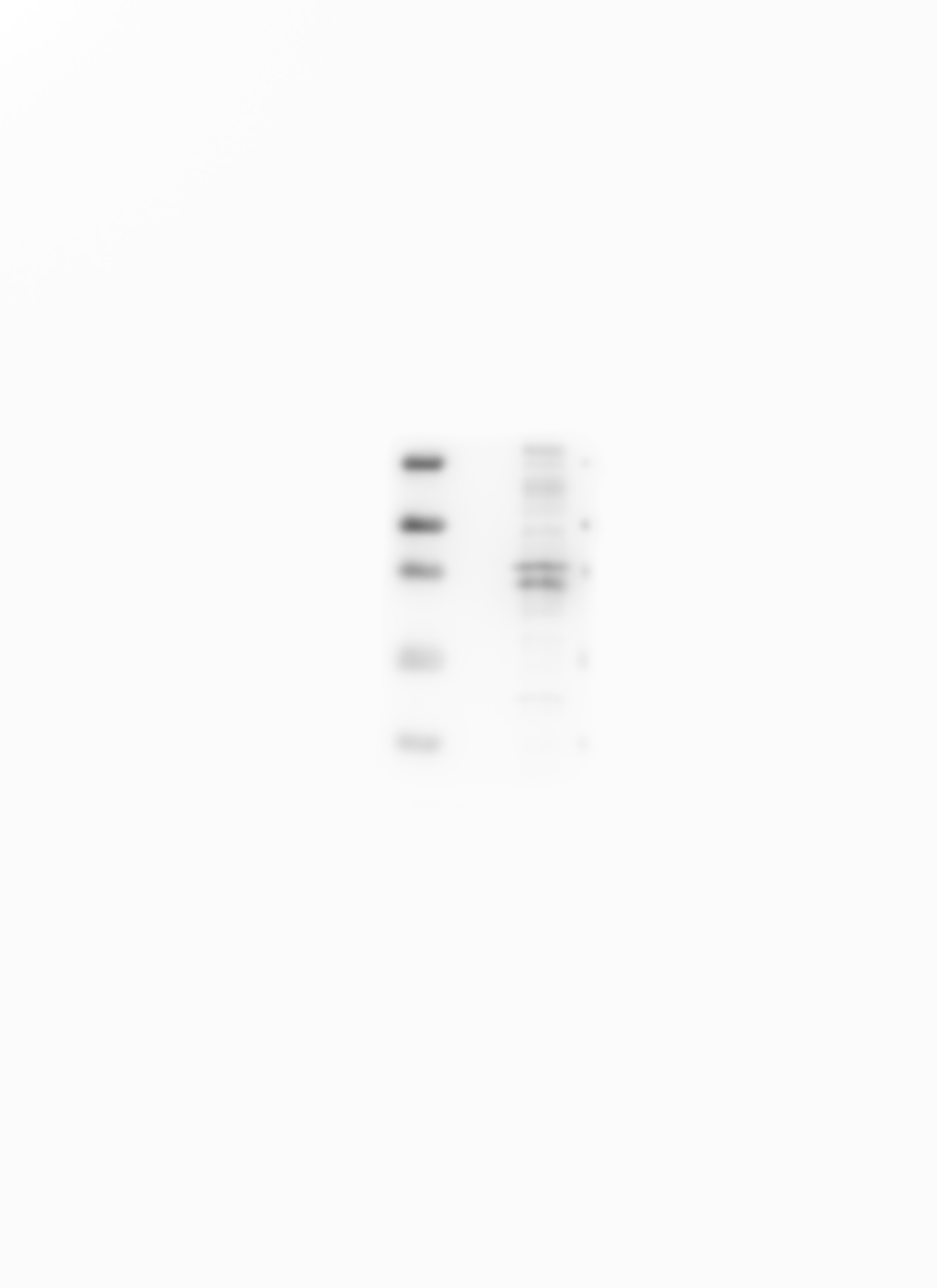

Supplement: Supplementary file 3 — WB Raw data [file 41420_2025_2583_MOESM3_ESM.zip › Figure 6 Panel D/suv39h2 ip 2022.04.03_11.24.01_Ch/suv39h2 ip 2022.04.03_11.24.01_Ch.tif]

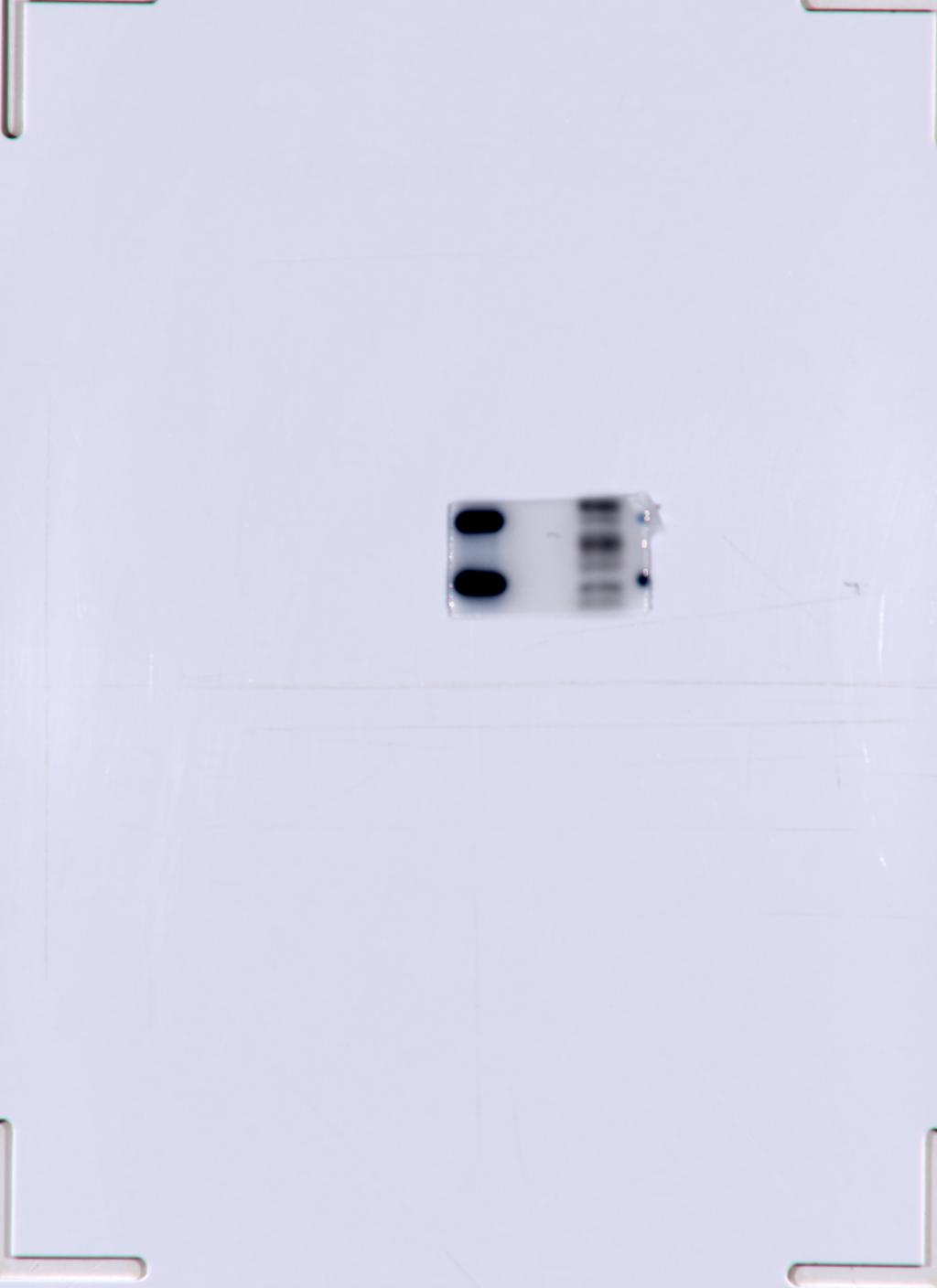

Supplement: Supplementary file 3 — WB Raw data [file 41420_2025_2583_MOESM3_ESM.zip › Figure 6 Panel D/suv39h2 ip 2022.04.03_11.26.14_Ch/suv39h2 ip 2022.04.03_11.26.14_Ch+Marker.jpg]
